# Evolutionary Relationship Between *Platycerus* Stag Beetles and Their Mycangium-Associated Yeast Symbionts

**DOI:** 10.3389/fmicb.2020.01436

**Published:** 2020-06-30

**Authors:** Kôhei Kubota, Kana Watanabe, Xue-Jiao Zhu, Kako Kawakami, Masahiko Tanahashi, Takema Fukatsu

**Affiliations:** ^1^Laboratory of Forest Zoology, Department of Forest Science, Graduate School of Agricultural and Life Sciences, The University of Tokyo, Tokyo, Japan; ^2^Laboratory of Forest Zoology, Course of Applied Life Sciences, Faculty of Agriculture, The University of Tokyo, Tokyo, Japan; ^3^Bioproduction Research Institute, National Institute of Advanced Industrial Science and Technology, Tsukuba, Japan; ^4^Department of Applied Chemistry, National Chiao Tung University, Hsinchu, Taiwan; ^5^Department of Biological Sciences, Graduate School of Science, The University of Tokyo, Tokyo, Japan; ^6^Graduate School of Life and Environmental Sciences, University of Tsukuba, Tsukuba, Japan

**Keywords:** co-evolutionary association, *Scheffersomyces*, mycangium, ITS, IGS, Japan

## Abstract

Adult females of stag beetles (Coleoptera: Lucanidae) possess an ovipositor-associated mycangium for conveying symbiotic microorganisms. In most lucanid species, their mycangium contains yeast symbionts of the genus *Scheffersomyces* Kurtzman and Suzuki that are known for their xylose-fermenting capability. The lucanid genus *Platycerus* Geoffroy, 1762 is a group of small blue stag beetles, in which ten Japanese species constitute a monophyletic clade. Here we examined the evolutionary relationships of these Japanese *Platycerus* species and their yeast symbionts, together with a Korean *Platycerus* species and other lucanid species as outgroup taxa. Based on the internal transcribed spacer (ITS) and the intergenic spacer (IGS) sequences, the yeast symbionts of all *Platycerus* species were closely related to each other and formed a monophyletic clade. There is no variation in ITS sequences of the yeast symbionts of the Japanese *Platycerus* species. Based on IGS sequences, the yeast symbionts formed clusters that largely reflected the geographic distribution of the host insects, being shared by sympatric *Platycerus* species except for *P. delicatulus* Lewis, 1883 and *P. viridicuprus* Kubota & Otobe, The symbiont phylogeny was globally not congruent with the host COI-based phylogeny, although some local congruences were observed. Statistically significant correlations were detected between the genetic distances of COI sequences of the host insects and those of IGS sequences of the yeast symbionts in Japan. These results suggest that, at least to some extent, the host insects and the yeast symbionts may have experienced co-evolutionary associations. While the Japanese *Platycerus* species formed a monophyletic clade in the COI phylogeny, the yeast symbionts of Japanese *P. viridicuprus* were very closely related to those of Korean *P. hongwonpyoi* Imura & Choe, 1989, suggesting the possibility that a recent secondary contact of the two beetle species during a marine withdrawal, e.g., in the last glacial period, might have resulted in an inter-specific horizontal transmission of the yeast symbiont.

## Introduction

Symbiotic relationships with fungi are known in diverse insect taxa (Vega and Blackwell, 2005; Biedermann and Vega, 2020). Particularly those between wood-inhabiting insects and fungi have attracted much attention, because such associations often enable utilization of woody materials that are abundant in the environment but difficult to digest as food. Since wood mainly consists of polymers such as cellulose, hemicellulose and lignin that are indigestible for most animals, microbial assistance is pivotally important for wood-feeding insects (Parkin, 1940; Haack and Slansky, 1987; Stokland, 2012). Although bacterial or protozoan symbionts of termites and wood-feeding cockroaches are anaerobic gut symbionts (Cleveland, 1924; Slaytor, 1992; Breznak and Brune, 1994), most fungal symbionts of xylophagous insects are aerobic and either gut or external symbionts (Batra, 1963; Suh et al., 2003, 2006; Nguyen et al., 2007; Grünwald et al., 2010). In ambrosia beetles, wood wasps and many other insects, fungal symbionts are carried and dispersed by external pouch-like organs called mycangia or mycetangia (Grebennikov and Leschen, 2010; Biedermann and Vega, 2020).

Stag beetles of the family Lucanidae, which embrace over 1,000 species in the world, mainly feed on decaying wood at their larval stages (Tanahashi et al., 2010; Kim and Farrell, 2015). Adult females of the Lucanidae possess a mycangium, constituted by an invaginated pouch of intersegmental membrane on the dorsal side of the abdominal tip, for conveying symbiotic microorganisms, which have been found in all the lucanid species representing 13 genera so far examined (*Aegus* Macleay, 1819; *Dorcus* Macleay, 1819; *Prosopocoilus* Hope & Westwood, 1845; *Lucanus* Scopoli, 1763; *Neolucanus* Thomson, 1862; *Prismognathus* Motschulsky, 1860; *Figulus* MacLeay, 1819; *Nigidius* MacLeay, 1819; *Platycerus* Geoffroy, 1762; *Aesalus* Fabricius, 1801; *Nicagus* LeConte, 1862; *Ceruchus* Macleay, 1819; *Sinodendron* Hellwig, 1792) (Tanahashi et al., 2010, 2017; Tanahashi and Hawes, 2016). They commonly harbor yeast symbionts in their mycangia (Tanahashi et al., 2010, 2017; Hawes, 2013) most of which belong to the genus *Scheffersomyces* Kurtzman & M. Suzuki known as a xylose-fermenting yeast group (Du Preez and Prior, 1985; Jeffries and Kurtzman, 1994; Olsson and Hahn-Hägerdal, 1996). Phylogenetically, the yeast symbionts are closely related (or belong) to *S*. *stipitis* (Pignal) Kurtzman & M. Suzuki or *S*. *segobiensis* (Santa María & C. García) Kurtzman & M. Suzuki (Tanahashi et al., 2010, 2017). Xylose is a main component of hemicelluloses in broad-leaved trees (Sjöström, 1993). Considering that hemicelluloses are difficult to utilize for most animals, the yeast symbionts of the stag beetles have been suspected to help lucanid larvae digest hemicelluloses (Tanahashi et al., 2010). The presence of mycangium in all lucanid species suggests an intimate relationship between the stag beetles and their yeast symbionts over evolutionary time. The yeast symbionts seem likely to be mutualistic to the host stag beetles, but so far, no proof for this has been found.

The genus *Platycerus* is a group of small blue stag beetles, widely distributed in the northern hemisphere and comprising more than 50 species, of which over a half are found in East Asia (29 species in China; 1 species in Korea; and 10 species in Japan based on Huang et al., 2010; Imura, 2010a,b, 2011, 2012; Huang and Chen, 2017; Kubota et al., 2008). Kubota et al. (2011) and Zhu et al. (2019), Zhu et al. (unpublished) inferred that all *Platycerus* species distributed in Japan are monophyletic and have been speciated since several hundred thousand years to some six million years ago. At most, three *Platycerus* species may be sympatrically found in Japan (Imura, 2010b; Kubota et al., 2011). Since many closely related species are distributed in the Japanese archipelago, the *Platycerus* stag beetles represent an interesting research target to investigate the evolutionary relationship between the host insects and the yeast symbionts in their speciation process.

Previously, it was shown that the yeast symbionts of each one female of *Platycerus delicatulus* Lewis, 1883 and *P*. *acuticollis* K. Kurosawa, 1969 from Japan and seven females of *P. hongwonpyoi* Imura & Choe 1989 from South Korea exhibited no genetic variation based on the sequences of the internal transcribed spacer (ITS) region and 26S ribosomal RNA gene (*26S rRNA*), which have been often used for fungal identification (Tanahashi et al., 2017). These sequences were the most similar to homologous sequences from the fungal symbionts of *Prismognathus angularis* Waterhouse, 1874 from Japan. In an attempt to detect more genetic variation among the yeast symbionts of the stag beetles, Tanahashi et al. (2017) adopted another molecular marker, intergeneic spacer (IGS) region including *26S rRNA*, IGS1, 5S ribosomal RNA gene (*5S rRNA*), IGS2, and 18S ribosomal RNA gene (*18S rRNA*) (>2,000 bp). The yeast symbionts showed some IGS sequence differences among collection sites and host species (Tanahashi et al., 2017).

In this study, we analyzed the evolutionary relationship between the stag beetles and their yeast symbionts. By focusing on the *Platycerus* species in Japan, (1) we investigated the genetic variation of the *Platycerus*-associated yeast symbionts based on ITS and IGS regions, and (2) we analyzed the presence or absence of co-evolutionary and geographic relationships between the host insects and the yeast symbionts.

## Materials and Methods

### Insects

**FIGURE 1:**
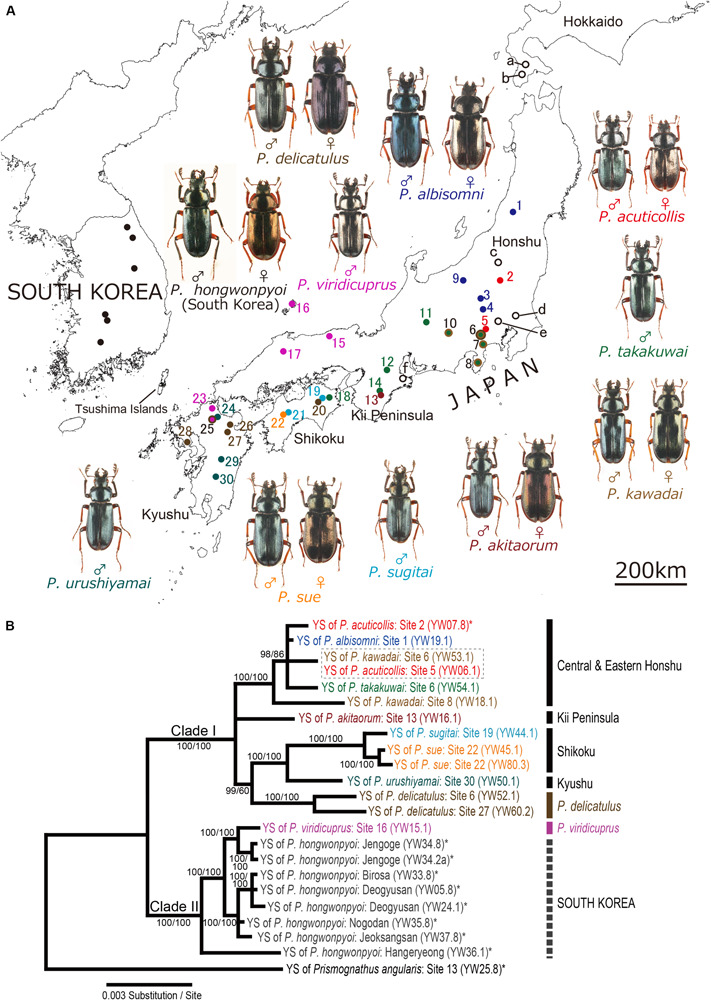
**(A)** Sample collection sites. Colored circles 1–30, collection sites of Japanese *Platycerus* species; open circles a-f, collection sites of outgroup lucanid species; filled circles in South Korea, collection sites of *P. hongwonpyoi* (Tanahashi et al., 2017). Note that sites a–c include several collection points. Also see Supplementary Table 1. **(B)** Bayesian inference (BI) phylogeny of the yeast symbionts of *Platycerus* stag beetles based on combined data of ITS and IGS sequences. Numbers at the nodes indicate posterior probability for BI phylogeny (>50%)/that recoding the gaps (50%). YS means yeast symbionts. Numbers following YW indicate the female code number and the strain number (see Supplementary Table 2). Dotted boxes indicate the combined haplotypes shared by more than one species of the host stag beetles. Asterisks indicate the sequences reported in previous studies. F81 model (ITS) and HKY+I model (IGS) were selected as the best-fit substitution model by jModelTest ver. 2.1.7. The BI phylogenies are identical regardless of the gap treatment.

In total, ten *Platycerus* species and five subspecies were collected at the sampling sites listed in Table 1. Most samples were obtained as adults in the field, and some were collected as larvae from decaying wood and reared individually to adulthood with the same wood pieces in which they were sampled in the field. Finally, we obtained in total 61 adult females representing all the *Platycerus* species and subspecies collected at 30 sites in Japan. We also collected 11 females of eight other lucanid species in Japan as outgroup taxa (Supplementary Tables 1, 2 and Figure 1A).

**TABLE 1:** ITS and IGS primers used in this study.

**Name**	**Sequence (5′-3′)**	**Strand direction**	Usage*	**References**
NS7	GAGGCAATAACAGGTCTGTGATGC	Forward	P, S	White et al., 1990
ITS5	GGAAGTAAAAGTCGTAACAAGG	Forward	S	White et al., 1990
NL4	GGTCCGTGTTTCAAGACGG	Reverse	P	White et al., 1990
IGS1	GCCTTGTTGTTACGATCTGC	Forward	P, S	Tanahashi et al., 2017
IGS2	ACCGTTTCCCGTCCGATCAAC	Reverse	S	Tanahashi et al., 2017
IGS3	TCCCACTACACTACTCGGTC	Forward	S	Tanahashi et al., 2017
IGS4	GAGACAAGCATATGACTAC	Reverse	P, S	Tanahashi et al., 2017
IGS7i	GAAGAGAGTTTAATGGTGAAC	Forward	S	Tanahashi et al., 2017
IGS8i	GTTCACCATTAAACTCTCTCC	Reverse	S	Tanahashi et al., 2017

*: P, PCR; S, sequencing analysis.

### Yeast Isolation From Adult Females

**FIGURE 2:**
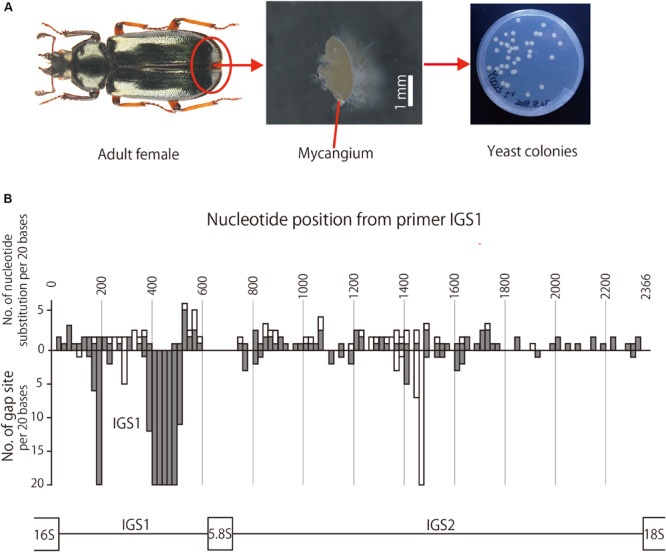
**(A)** Adult female, dissected mycangium, and isolated yeast colonies of *P. kawadai*. **(B)** Site-dependent sequence polymorphisms in IGS region among the yeast symbionts of Japanese *Platycerus* species (*n* = 60), *P. hongwonpyoi* from South Korea (*n* = 6), and *Prismognathus angularis* (*n* = 1). Bars above the horizontal line represent the numbers of nucleotide substitution, and those bellow the horizontal line represent the numbers of gap sites for every 20 bases of sequences. White and gray bars indicate the numbers within all data sets and those within *Platycerus* species, respectively.

A mycangium, which is located under the eighth abdominal tergite, was dissected from each adult female in sterilized phosphate-buffered saline (PBS) as described in Tanahashi et al. (2017) (Figure 2A). After removing surrounding exoskeletal membranes, the dissected mycangium was homogenized in PBS using a pellet pestle in a plastic tube, and each of 5-fold dilution series of the homogenate (equivalent to 1/5^1^, 1/5^2^, …, 1/5^5^ of the dissected mycangium) was spread onto a potato dextrose agar (PDA: Sigma Aldrich, St. Louis, MO, United States) plate containing 50 μg/ml rifampicin. The plates were incubated at 20°C for 4 days, which had been established as suitable temperature and duration for growth of the *Platycerus* yeast symbionts (Tanahashi et al., 2017). The number of colony-forming units (CFU) per organ was calculated based on a plate on which an adequate number (usually 30∼300) of colonies appeared (Figure 2A).

We obtained yeast symbiont colonies from mycangial homogenates prepared from 60 of 61 *Platycerus* females (98.4 %) collected in Japan. We also obtained yeast symbiont colonies from the other lucanid species (Supplementary Table 2). The colonies on PDA plates were uniform, white, circular, and protuberant, having a smooth edge and matte surface. The yeast CFU values ranged from 9.2 × 10^2^ to 5.3 × 10^4^ (*n* = 59) for the *Platycerus* species, and from 2.1 × 10^4^ to 4.2 × 10^5^ (*n* = 11) for the other lucanid species.

### DNA Sequences of Yeast Symbionts

Since no morphological differences were observed among the yeast colonies that appeared on the plate, we randomly selected 2–8 colonies for each host individual and transferred them to new PDA plates for subsequent DNA analyses. A small pellet of each yeast colony was suspended in 50 μL of lyticase solution (0.4 U/μL lyticase, 50 mM ethylenediaminetetraacetic acid [EDTA]) and incubated at 37°C for 2 h. The cell suspension was centrifuged at 9,000 rpm for 5 min, and the supernatant containing cell wall saccharides was discarded. The yeast pellet was then re-suspended in 80 μl of the cell lysis solution (1% SDS, 4 M urea, 1 mM EDTA, 150 mM NaCl and 50 mM Tris-HCl; pH 8.0) and incubated at 55°C for 1 h. After cooling, 27 μl (one-third volume of the lysate) of 8 M ammonium acetate was added, mixed vigorously and incubated on ice for 10 min. The solution was centrifuged at 9,000 rpm for 10 min. Crude nucleic acids were obtained from the supernatant (approximately, 80 μl) by isopropanol precipitation followed by 70% ethanol wash. The yeast DNA was dissolved in TE buffer (10 mM Tris-HCl, 1 mM EDTA; pH 8.0) as described (Tanahashi et al., 2017).

The entire nucleotide sequences of ITS and IGS regions were amplified by polymerase chain reaction (PCR) at 94°C for 5 min followed by 35 cycles of 95°C for 45 s, 54°C for 45 s, and 72°C for 1 min 30 s (for ITS) or 2 min 15 s (for IGS), by using the primer sets NS7-NL4 (for ITS) and IGS1-IGS4 (for IGS) (Table 1). The PCR products were purified using the Illustra ExoStar clean-up kit (GE Healthcare, Buckinghamshire, United Kingdom). Dye terminator cycle sequencing reactions were performed using the ABI Big Dye Terminator Cycle Sequencing Ready Reaction Kit (Applied Biosystems, Foster, CA, United States), and the reaction products were analyzed by an ABI 3130xl genetic analyzer (Applied Biosystems). The primers used for the sequencing analyses were listed in Table 1.

To detect fine genetic variation of the symbiotic yeasts, IGS PCR products from all isolates (i.e., 2–8 colonies per host female) were first sequenced with the primer IGS1. This short sequence (∼700 bp) covered the entire IGS1 region, which was subsequently cut out from the raw sequence data. We used this entire IGS1 sequence (namely, IGS1 haplotype) for a discrimination marker of *Scheffersomyces* symbiotic yeast strains on the ground that IGS1 is known for the largest genetic variety among IGS regions (Tanahashi et al., 2017). When more than one IGS1 haplotypes were detected from a single host female, we chose one representative isolate for each different IGS1 haplotype for further phylogenetic analyses. This process is the analytical strategy practically adopted by Tanahashi et al. (2017). After that, the whole IGS sequence (approximately 2.2 kb) was determined for each representative isolate for each host female (in total 60 sequences) (Supplementary Table 2) using the sequencing primers IGS2, IGS3, IGS4, IGS7i and IGS8i.

Although IGS regions are suitable for detecting strain-level genetic differences, they are not commonly used for species identification or phylogenetic analysis at higher taxonomic levels, because of too much genetic variation as well as insufficient taxonomic coverage in the databases. Therefore, for the purposes of species identification and phylogenetic analysis of the symbiotic yeasts, we chose 23 symbiotic yeast isolates from 12 females representing all the Japanese *Platycerus* species (Supplementary Table 2). The ITS PCR products of the representative yeast isolates were sequenced with the primer NS7 and ITS5 (Table 1), which were around 0.7 kb in size and usually contained whole ITS regions (ITS1, *5.8S rRNA*, and ITS2) of the *Scheffersomyces* yeasts. We also determined ITS sequences of 11 symbiotic yeast strains that were isolated from eight outgroup lucanid species (Supplementary Table 2).

### DNA Sequences of Insect Hosts

For DNA sequencing analysis of host *Platycerus* beetles, Kubota et al. (2011) reported that cytochrome oxidase subunit I (COI) gene exhibited suitable levels of variation for detecting genetic divergence among species and populations, whereas some nuclear genes did not. Introgressive hybridization has been recognized in some Japanese *Platycerus* species (e.g., *P. takakuwai* Fujita, 1987) based on the 28S ribosomal RNA (*28S rRNA*) and mitochondrial cytochrome oxidase subunit I (COI) genes. It should be noted, however, that both the mitochondria and the symbiotic yeasts are essentially transmitted from mother to offspring (Tanahashi and Hawes, 2016). Since it seems to be reasonable to compare the evolutionary patterns between COI gene of the host beetles and *IGS* gene of the yeast symbionts, we sequenced COI gene of the host beetles. It should be noted that we were not always able to use the same beetle samples for the yeast isolation and the host DNA analysis. In such cases, we used alternative beetle samples collected at the same field sites. A considerable part of those COI sequences have already been published in previous studies (Kubota and Kubota, 2011; Tanahashi et al., 2017). Therefore, we newly analyzed 18 Japanese *Platycerus* samples that were needed in this study.

Genomic DNA was extracted from the testes or muscle tissues of adult *Platycerus* beetles preserved in ethanol using the Wizard Genomic DNA Purification Kit (Promega, Madison, WI, United States). The COI gene (primers C1-J-2183 and L2-N-3014, Simon et al., 1994) was amplified by PCR at 94°C for 3 min followed by 30 cycles of 94°C for 1 min, 48°C for 1 min, and 72°C for 1 min, with a final 7 min extension at 72°C. The PCR products were purified using the Illustra ExoStar clean-up kit (GE Healthcare, Buckinghamshire, United Kingdom). The Dye terminator cycle sequencing reactions were performed using the ABI Big Dye Terminator Cycle Sequencing Ready Reaction Kit (Applied Biosystems, Foster, CA, United States), and electrophoresed using an ABI 3130xl genetic analyzer (Applied Biosystems). For the sequencing analysis, the same primer sets as PCR were used.

### Phylogenetic Analyses

Besides the 23 ITS sequences, 60 IGS sequences, and 23 COI sequences determined in this study, we also used the following sequences reported in previous studies: 18 yeast ITS sequences, of which 13 were symbionts of lucanid beetles (two from Japanese *Platycerus* species, eight from *Platycerus hongwonpyoi* of South Korea, and three from other lucanid species); 11 yeast IGS sequences, of which two were from Japanese *Platycerus* species, eight were from *Platycerus hongwonpyoi* of South Korea, and one was from Japanese *Prismognathus angularis*; 27 Japanese *Platycerus* COI sequences (Supplementary Table 2).

We constructed a haplotype matrix for each of the ITS and IGS regions of yeasts, and the COI gene of beetles using the DnaSP ver. 5.10.01 software package (Librado and Rozas, 2009). To align the sequences, we used ClustalW implemented in BioEdit ver. 7.2.5 (Hall, 1999). Gaps were treated as missing data. Aligned haplotype sequences were used to construct phylogenetic trees based on maximum likelihood (ML) and Bayesian inference (BI) methods. In addition, 23 combined sequences of ITS and IGS were used to construct phylogenetic trees based on BI methods. The sequence alignments used for the phylogenetic analyses are available as Supplementary Appendices 1–4.

ML trees were constructed using PhyML ver. 3.0 (Guindon and Gascuel, 2003) under the best-fit substitution model selected using jModelTest ver. 2.1.7 (Darriba et al., 2012) based on the Bayesian information criterion (BIC). Confidence at each node was assessed by 100 bootstrap replications.

BI trees were constructed using three runs in MrBayes ver. 3.2.6 (Ronquist and Huelsenbeck, 2003) under the best-fit substitution model selected by jModelTest ver. 2.1.7, for 200 million generations (samplefreq = 20,000) and 2,500 samples burn-in, using Tracer ver. 1.5.0 (Rambaut and Drummond, 2016) to examine convergence toward high effective sample sizes.

For the ITS, IGS, and their combined sequences, we also constructed BI trees based on gap-recoded sequences using FastGap 1.2 (Borchsenius, 2009) to minimize the effect of gaps.

### Relationships Among Geographic Distance of Sites, Genetic Distance of Yeasts, and Genetic Distance of Host Beetles

For analyzing co-evolutionary relationships based on genetic sequence data, several methods, e.g., SH-test, AU-test, and likelihood-ratio test (Kawakita, 2008) are available. In these methods, the null hypothesis is that the phylogenies of the hosts and parasites are the same. In this study, since the host insect phylogeny and the yeast symbiont phylogeny seemed to be not at all the same, we adopted the Parafit test (Legendre et al., 2002) using *p*-distances between IGS sequences of the yeast symbionts and those between COI sequences of the host insects, where null hypothesis is that their phylogenies are not correlated. In addition, *Platycerus* species and populations are genetically differentiated in Japan, and their genetic divergence seems to be related to their geographic distribution (Kubota et al., 2011). Therefore, we also used the Mantel test to remove the effects of geographical distance.

We randomly chose a symbiont IGS sequence and a host COI sequence for each host beetle population to avoid duplication of beetle populations. Geographic distances between the collection sites of Japanese *Platycerus* species were estimated using a distance calculation tool available at https://vldb.gsi.go.jp/sokuchi/surveycalc/surveycalc/bl2stf.html (cited 3 May 2018). Genetic *p*-distances (pairwise nucleotide diversity) of IGS sequences and COI sequences between the insect populations were calculated using Arlequin ver. 3.1 (Excoffier et al., 2005).

We examined the relationship between the geographic distance of the collection sites and the IGS *p*-distance of the yeast symbionts, the relationship between the geographical distance of the collection sites and the COI *p*-distance of the host beetles, and the relationship between the IGS *p*-distance of the yeast symbionts and the COI *p*-distance of the host beetles by the Mantel test. The Parafit test and the Mantel test were conducted based on 10,000 pseudoreplications of randomization.

## Results

### Taxonomic Position of Yeast Symbionts Inferred From ITS Phylogeny

All the ITS sequences of the yeast symbionts of the *Platycerus* species collected in Japan exhibited no sequence variation (containing partial *18S rRNA*, ITS1, *5.8S rRNA*, ITS2, and partial *26S rRNA*; 652 bp; *n* = 23 from 12 females). These sequences were completely identical to the sequences previously reported from two Japanese *Platycerus* species and seven Korean individuals of *P. hongwonpyoi* (Tanahashi et al., 2017) contained only two nucleotide substitutions compared with the sequence of the yeast symbiont from *Prosmognathus angularis* (Tanahashi et al., 2017) and were the most similar to the sequence of *Scheffersomyces segobiensis* (Tanahashi et al., 2017) among formally described yeast species. Note that *S. segobiensis* was isolated from a jewel beetle *Chalcophora mariana massiliensis* (Villers, 1789) (Coleoptera: Buprestidae) (Santa-María and García-Aser, 1977). Phylogenetic analysis based on the ITS sequences revealed that the yeast symbionts of the *Platycerus* species constitute a distinct lineage in the clade of the yeast symbionts of the other lucanid species including environmental *Scheffersomyces* isolates (Supplementary Figure 1).

### Phylogeny of Yeast Symbionts and Host Beetles, and Comparison With Symbiont Phylogeny

We first tested genetic variation of the yeast symbionts based on 2–8 colonies within a host female by using the primers IGS1 and IGS2 (covering partial *26S rRNA*, IGS1, *5S rRNA*, and partial IGS2; >700 bp in size). The IGS1 sequences of multiple yeast colonies isolated from a host female were identical for all the *Platycerus* females examined, suggesting that there is little genetic variation among the yeast symbionts in the mycangium of a single female. Therefore, a representative isolate was randomly selected for each of the all female samples, and the entire sequences of IGS regions (partial *26S rRNA*, IGS1, *5S rRNA*, IGS2, and partial *18S rRNA*; 2177–2311 bp; *n* = 59) were sequenced and subjected to the phylogenetic analysis.

For the IGS region, the nucleotide substitution rate was higher in the order of IGS1, the former half of IGS2, and the latter half of IGS2 (Figure 2B). Gap sites were concentrated much in IGS1, and a little in the second quarter of IGS2 (Figure 2B).

In the IGS phylogeny, two major clades were recognized among the yeast symbionts of *Platycerus* stag beetles: Clade I contained most of the Japanese *Platycerus* populations, whereas Clade II embraced most populations of *P. viridicuprus* Kubota et al. (2008) in Japan and all populations of *P. hongwonpyoi* in South Korea. Most of the Japanese *Platycerus* species (except for *P. delicatulus* and most of *P. viridicuprus* populations) distributed in the same areas tended to share the yeast symbionts belonging to the same subclade: Clade Ia, central and eastern Honshu; Clade Ib, the Kii Peninsula; and Clade Ic, Shikoku and Kyushu (Supplementary Figure 2; also see Figure 1A). The yeast symbionts of *P. delicatulus* populations formed a monophyletic group of Clade Id. Clade II consisted of the yeast symbionts of most *P. viridicuprus* populations (western Honshu and northern Kyushu) and all *P. hongwonpyoi* populations in South Korea. Only one female of *P. viridicuprus* (Site 25; northern Kyushu) possessed the yeast symbiont that represented the same IGS sequence to *P. urushiyamai* Imura, 2007 (Supplementary Figure 2). The ITS + IGS combined phylogeny based on 23 samples exhibited the same topology as the IGS phylogeny (Figure 1B).

**FIGURE 3:**
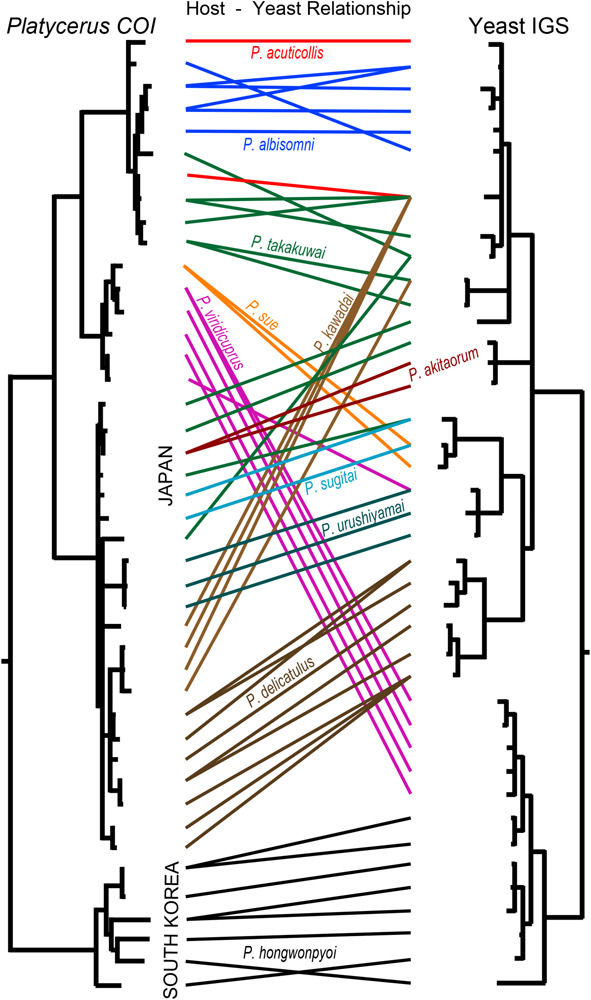
Comparison between IGS phylogeny of the yeast symbionts and COI phylogeny of their host *Platycerus* stag beetles. The phylogenies are taken and modified from Supplementary Figures 2, 3. The colored lines show the correspondence of the yeast symbionts and their insect hosts.

We constructed COI phylogeny of the host insects (Supplementary Figure 3), and compared it with IGS phylogeny of the yeast symbionts (Figure 3). Globally, the host phylogeny and the symbiont phylogeny were not congruent, indicating that no strict host-symbiont co-speciation has been established in the evolutionary course of the *Platycerus* stag beetles. On the other hand, it was evident that the same *Platycerus* species tended to be associated with closely related yeast symbionts (Figure 3) suggesting that stable host-symbiont associations may be locally observed, at least to some extent, in the *Platycerus* stag beetles.

In each phylogenetic analysis, number of OTUs, length of aligned sequence, numbers of informative sites, gap sites, and recoded gap sites using FastGap 1.2 are shown in Table 2.

**TABLE 2:** Numbers of OTUs and haplotypes, length of aligned sequence, numbers of informative sites, gap sites, and recoded gap sites in each phylogenetic analysis.

**Organism**	**Analized region**	**Number of OTUs**	**Number of haplotypes**	**Number of sites (bp)**			
				**Length of aligned sequence**	**Informative sites**	**Gap sites**	**Recoded gap sites^∗^**
Yeast	ITS	41	15	672	73	51	29
	IGS	71	41	2,366	157	206	32
	ITS + IGS	23	22	2,892	140	71	27
Beetle	COI	45	44	753	225	0	–

*, recoded using FastGap 1.2.

### Relationships Among Geographic Distances Between Collection Sites, Genetic Distances Between Yeast Symbionts, and Genetic Distances Between Host Insects

**FIGURE 4:**
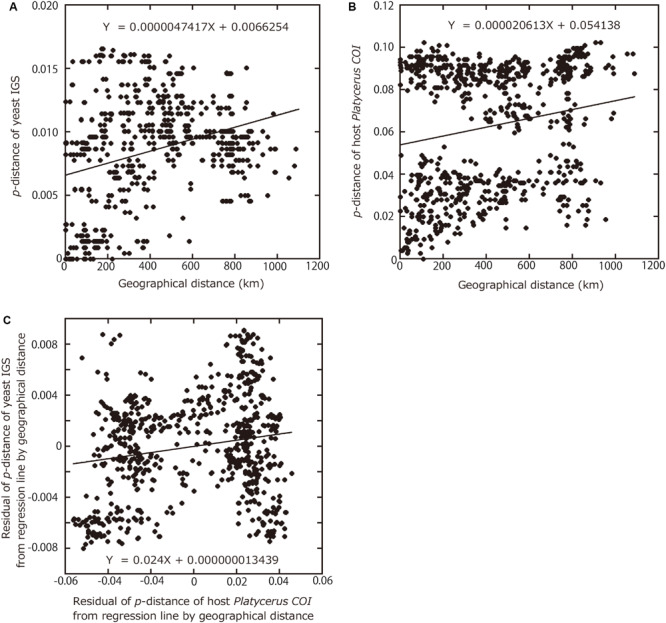
Relationships among geographic distances between collection sites, genetic distances between yeast symbionts, and genetic distances between host *Platycerus* stag beetles. **(A)** Relationship of geographical distances between collection sites versus *p*-distances between the symbiont IGS sequences. **(B)** Relationship of geographical distances between collection sites versus *p*-distances of the host COI sequences. **(C)** Relationship of residual *p*-distances between the host COI sequences regressed to geographical distances versus residual *p*-distances between the symbiont IGS sequences regressed to geographical distances.

Finally, in an attempt to quantitatively evaluate the phylogenetic and geographic aspects of the host-symbiont association in the *Platycerus* stag beetles, correlations between the IGS *p*-distances among the yeast symbionts, the COI *p*-distances among the host insects, and the geographic distances between the collection sites were analyzed statistically. The IGS *p*-distances between the yeast symbionts were significantly correlated with the COI *p*-distances between the host insects (*P* < 0.0001, Parafit test with 10,000 replicates). The IGS *p*-distances between the yeast symbionts were significantly correlated with the geographical distances between the collection sites (*P* < 0.0001, Mantel test with 10,000 replicates) (Figure 4A). Similarly, the COI *p*-distances between the host insects were significantly correlated with the geographical distances between the collection sites (*P* < 0.0001, Mantel test with 10,000 replicates) (Figure 4B). Reflecting that the Japanese *Platycerus* species and populations were phylogenetically divided into two large clades (Supplementary Figure 3), their genetic distances form two clusters in Figure 4B.

For both IGS and COI, regression lines were obtained with geographical distances as the explanatory variables and *p*-distances as the dependent variables. To remove the effects of the geographic distances, the residuals of *p*-distance from regression lines were calculated. Even after removing the effects of the geographic distances, the residuals of IGS *p-*distances between the yeast symbionts were significantly correlated with the residuals of COI *p*-distances between the host insects (*P* = 0.0004, Mantel test with 10,000 replicates) (Figure 4C).

## Discussion

In this study, we demonstrated that almost all the adult females of Japanese *Platycerus* stag beetles are associated with the specific *Scheffersomyces* yeast symbiont within their mycangium (Figure 2A). Based on ITS sequences, all the yeast symbionts isolated from Japanese and Korean *Platycerus* species and populations exhibit no sequence variation, constituting a distinct lineage within the *Scheffersomyces* yeast symbionts of diverse stag beetles (Supplementary Figure 1). These patterns suggest the possibility that the Japanese and Korean *Platycerus* species are associated with a clade of yeast symbionts that shares the common fungal ancestor with *S*. *segobiensis* isolated from a jewel beetle (Supplementary Figure 1).

Based on IGS sequences, we uncovered that the yeast symbionts of Japanese *Platycerus* stag beetles exhibit considerable genetic and geographic diversity, in which sympatric *Platycerus* species tend to be associated with the same or closely related yeast symbionts (Supplementary Figure 2). Notable examples are *P. kawadai* Fujita & Ichikawa, 1982 and *P. takakuwai* at sites 6, 7 and 8 in Central Honshu; *P. takakuwai* and *P. sugitai* Okuda & Fujita, 1987 at sites 18 and 19 in Shikoku; *P. sue* Imura, 2007 and *P. sugitai* at sites 21 and 22 in Shikoku; *P. urushiyamai* and *P. viridicuprus* at sites 24 and 25 in Kyushu (Supplementary Figure 2). These patterns strongly suggest that the yeast symbionts must have been occasionally transferred between the sympatric host species, plausibly via sharing of the same decaying wood as the larval habitat.

By contrast, we found that, across *P. delicatulus* populations covering a wide distribution range, their yeast symbionts form a distinct monophyletic clade (Supplementary Figure 2). Considering that *P. delicatulus* is sympatrically found with many other *Platycerus* species (Figure 1A) the host-symbiont relationship in *P. delicatulus* must have been robust without lateral transfer events. It is currently obscure why the host-symbiont connection is stable in *P. delicatulus*, but we suggest the possibility that ecological aspects of *P. delicatulus* might be relevant. While larvae of most *Platycerus* species feed on thin, wet and soft decaying wood on the ground, larvae of *P. delicatulus* live on large and dry decaying wood, typically standing dead trees (Imura, 2010b). It is conceivable, although speculative, that the distinct ecological niche of *P. delicatulus* prevents lateral symbiont transfers from other *Platycerus* species, thereby underpinning the host-symbiont fidelity.

Previous molecular phylogenetic studies reported that Japanese *Platycerus* species form a monophyletic clade and are genetically distinct from Korean *P. hongwonpyoi* (Imura and Nagahata, 2009; Kubota et al., 2011) (see Supplementary Figure 3). Notwithstanding this, based on IGS sequences, the yeast symbionts of *P. viridicuprus* form a well-supported monophyletic clade with the yeast symbionts of *P. hongwonpyoi* (Figure 1B and Supplementary Figure 2). How can this host-symbiont discrepancy be interpreted? A possible evolutionary scenario is that *P. viridicuprus* has been maintaining an ancestral type of the yeast symbiont since the ancestral *Platycerus* species dispersed from the Korean Peninsula to Japan. However, although the Japanese *Platycerus* clade including *P*. *viridicuprus* and the Asian continental *Platycerus* clade including *P*. *hongwonpyoi* diverged about 10 million years ago based on COI sequences (Zhu et al., unpublished) the yeast symbionts of *P. viridicuprus* are genetically very close to the yeast symbionts of *P. hongwonpyoi* (Figure 1B and Supplementary Figure 2). These facts do not favor the above idea. An alternative, and more likely evolutionary scenario is that a recent secondary contact of *P. viridicuprus* and *P. hongwonpyoi* upon a marine withdrawal around the Tsushima Islands (see Figure 1A) resulted in the lateral symbiont transfer between the two species. Note that one female of *P. viridicuprus* collected at the western distribution edge (Figure 1A, site 25 in northern Kyushu) exceptionally possessed the yeast symbiont belonging to the Kyushu clade of Clade Ic, which can be explained by a lateral symbiont transfer from *P. urushiyamai* to *P. viridicuprus*.

The results of Parafit and Mantel tests showed that the genetic divergence of the *Platycerus* stag beetles and the genetic divergence of the yeast symbionts are significantly correlated to each other, with or without considering the effects of the geographic distance (Figure 4). These results suggest that the divergence of the host insects has constrained the divergence of the yeast symbionts, and/or *vice versa*. In other words, the *Platycerus* stag beetles and the yeast symbionts have co-evolved to some extent but only incompletely, which reflects the following phylogenetic patterns. Globally, the symbiont phylogeny is not at all congruent with the host phylogeny (Figure 3) which strongly suggests that losses, gains, lateral transfers and/or replacements of the *Scheffersomyces* yeast symbionts must have occurred repeatedly in the evolutionary course of the *Platycerus* stag beetles. Plausibly, these dynamic evolutionary aspects are relevant to the following factors: (i) the *Scheffersomyces* yeast symbionts are cultivable and thus capable of surviving outside the symbiotic organ of the insect hosts (Figure 2A), (ii) allied *Scheffersomyces* yeasts are commonly found in and isolated from the galleries of decaying woods where lucanid larvae inhabit (Kubota et al., 2010) (iii) the *Scheffersomyces* yeasts are capable of xylose fermentation and thus likely to adapt to proliferation and survival on woody materials (Jeffries et al., 2007; Tanahashi et al., 2010). On the other hand, focusing on local relationships, the same host species tend to be associated with closely related yeast symbionts (Figure 3), suggesting that stable host-symbiont associations may be observed in the *Platycerus* stag beetles to some extent. In addition, *Platycerus* species and *Prismognathus angularis* often coexist in the same wood pieces, each of them is associated with its own yeast symbiont lineage, and these two genera never share the same yeasts (Figure 1B and Supplementary Figure 2; Zhu et al., unpublished). These local host-symbiont affinities are likely to be underpinned by the mycangium-mediated vertical transmission of the yeast symbionts generally found in stag beetles (Tanahashi et al., 2010) and also by presumable low dispersal ability of the insects. Overall, these complex phylogenetic and genetic patterns highlight complex evolutionary trajectories of the host-symbiont relationships in the *Platycerus* stag beetles.

Diverse taxa of yeasts are known to be associated with a variety of coleopteran families such as Platypodidae, Scolytidae, Rhynchophoridae, Buprestidae, Bostrychidae, Chrysomelidae, Nitidulidae, Erotylidae, Mycetophagidae, Ciidae, and Lucanidae (Kurtzman et al., 2011; Endoh, 2012). They are isolated from various parts of insect bodies (e.g., whole body, gut, ovary, etc.), larval galleries, and feces. However, only a small fraction of the yeast associates are known to provide mutual benefits to the host beetles. For example, the yeast symbionts of Scolytidae improve the nutritional and environmental conditions of the host beetles (Hunt and Borden, 1990; Rivera et al., 2009). Also the yeast symbionts of Platypodidae are thought to nutritionally contribute to the host beetles (Endoh, 2012). In the beetle bodies, especially within their gut, specific fungal floras including diverse yeasts have been commonly recognized, which often exhibit inter- and intra-specific variations (Shifrine and Phaff, 1956; Leufvén and Nehls, 1986; Suh et al., 2004, 2006; Suh and Blackwell, 2005; Endoh et al., 2011).

*Scheffersomyces* yeasts are not only found from *Platycerus* and other groups of the Lucanidae (Van der Walt et al., 1971; Tanahashi et al., 2010, 2017; Hawes, 2013; Tanahashi and Hawes, 2016) but also detected from the larval gut of wood-feeding beetles belonging to the Passalidae, the Cerambycidae and the Scarabaeidae (Suh et al., 2003, 2006; Kubota et al., 2011) although no mycangium-like symbiotic organs have been identified in these non-lucanid beetles (Tanahashi et al., 2010). Considering that *Scheffersomyces* is the almost only fungus found in the female-specific mycangium of *Platycerus*, *Dorcus*, *Lucanus* and other lucanid beetles (though some fungal flora may exist in their larval gut), it seems likely that the *Scheffersomyces* yeast symbionts play some important roles for the host stag beetles. Thus far, however, the exact symbiont function has been elusive. In *Platycerus* species, the yeast symbionts are constantly found in the larval gut and galleries but not in the adult gut (Kubota et al., 2010). On account of the xylose-fermenting capability of *Scheffersomyces* yeasts (Du Preez and Prior, 1985; Jeffries and Kurtzman, 1994; Olsson and Hahn-Hägerdal, 1996) it is conceivable, although speculative, that the yeast symbionts may contribute to the survival or development of the host insect larvae by helping hemicellulose digestion in the decaying wood (Tanahashi et al., 2010, 2017). In fact, we confirmed that most yeast symbionts of stag beetles examined in this study can utilize xylose, while *Saccharomyces cerevisiae* cannot (Watanabe et al., unpublished data). How and why specific *Scheffersomyces* yeast symbionts are selected and colonize the mycangium is of great interest and deserves future studies. To address these questions, we should investigate the horizontal and vertical transmission routes and mechanisms of the yeast symbionts between the host beetles, natural ex-host habitats of the yeast symbionts in the field, the effects of the yeast symbionts on host growth and survival, and other biological aspects of the lucanid-yeast associations.

## Data Availability Statement

The datasets generated for this study can be found in the DDBJ: LC438646–LC438745.

## Author Contributions

KW, KaK, and MT contributed to the data generation of this study. KôK and X-JZ performed genetic analyses. KôK contributed to the study design with the help of MT. KôK and TF wrote the manuscript. All authors approved the final version of the manuscript.

## Conflict of Interest

The authors declare that the research was conducted in the absence of any commercial or financial relationships that could be construed as a potential conflict of interest.
