# Supplementary material for: Evolutionary Relationship Between Platycerus Stag Beetles and Their Mycangium-Associated Yeast Symbionts
Source: Front Microbiol. 2020 Jun 30;11:1436. doi: 10.3389/fmicb.2020.01436 (PMC7338584; doi:10.3389/fmicb.2020.01436)
Supplement: Supplementary file 1 [file Data_Sheet_1.PDF]

SI Table 1. Sample collection Sites.

Site	Locality	Altitude	Latitude	Longitude
1	Yumiharidaira, Nishikawa-machi, Yamagata Pref.	640	38.48	140.01
2	Mt. Takayama, Nikko-shi, Tochigi Pref.	1280	36.75	139.44
3	Mt.Akagunayama, Fujioka-shi, Gunma Pref.	1100	36.15	138.90
4	Irikawa, Chichibu-shi, Saitama Pref.	1300	35.94	138.80
5	Kubo, Dôshi-mura, Yamanashi Pref.	460	35.54	139.09
6	Mt. Kanyudoyama, Yamakita-chô, Kanagawa Pref.	1400	35.51	139.05
7	Mt. kamiyama, Hakone-chô, Kanagawa Pref.	1420	35.23	139.02
8	Haccho-ike, Izu-shi, Shizuoka Pref.	1200	34.85	138.96
9	Mt. Kenashiyama, Nozawaonsen-mura, Nagano Pref.	1330	36.91	138.48
10	Mt. Torikurayama, Ôshika-mura, Nagano Pref.	1640	35.55	138.09
11	Mt. Funayama, Takayama-shi, Gifu Pref.	1470	36.02	137.24
12	Mt. Ôborayama, Tsu-shi, Mie Pref.	770	34.53	136.22
13	Mt. Nagoyadake, Ôdaigaharayama, Ôdai-chô, Mie Pref.	1520	34.19	136.10
14	Mt. Obagamine, Kamikitayama-mura, Nara Pref.	1100	34.23	136.02
15	Mt. Ôginosen, Shin-onsen-chô, Hyôgo Pref.	1000	35.44	134.46
16	Mt. Washigamine, Okinoshima-chô, Shimane Pref.	450	36.26	133.33
17	Mt. Tachieboshiyama, Shôbara-shi, Hiroshima Pref.	1180	35.05	133.07
18	Dosu-tôge Pass, Kamiyama-chô, Tokushima Pref.	1030	33.92	134.29
19	Mt. Marusasayama, Mima-shi, Tokushima Pref.	1370	33.87	134.09
20	Mt. Tsurugisan, Miyoshi-shi, Tokushima Pref.	1320	33.87	134.09
21	Mt. Ishizuchisan, Saijo-shi, Ehime Pref.	1430	33.75	133.15
22	Mt. Ishizuchisan, Saijo-shi, Ehime Pref.	1680	33.77	133.12
23	Mt. Fukuchiyama, Nôgata-shi, Fukuoka Pref.	680	33.75	130.80
24	Mt. Hikosan, Hikosan, Soeda-machi, Fukuoka Pref.	960	33.48	130.93
25	Mt. Gakumekisan, Soeda-machi, Fukuoka Pref.	740	33.46	130.91
26	Mt. Yufudake, Beppu-shi, Ôita Pref.	1100	33.28	131.40
27	Mt. Kurodake, Yufu-shi, Ôita Pref.	880	33.12	131.29
28	Mt. Gokaharadake, Ômura-shi, Nagasaki Pref.	900	32.96	130.08
29	Mt. Shiratoriyama, Yatsushiro-shi, Kumamoto Pref.	1480	32.48	131.00
30	Mt. Shiragadake, Asagiri-machi, Kumamoto Pref.	1370	32.16	130.94
a	Assabu-chô, Hiyama-gun, Hokkaido Pref.			
b	Kaminokuni-chô, Hiyama-gun, Hokkaido Pref.			
c	Minamiaizu-chô, Aizu-gun, Fukushima Pref.			
d	Shôrenji, Kashiwa-shi, Chiba Pref.			
e	Midori-machi, Nishitokyo-shi, Tokyo Met.			
f	Nakajima, Ise-shi, Mie Pref.			

Sites 1–30, for *Platycerus* species; Sites a–f, for outgroup; Sites a–c, including several collection points.
