# Supplementary material for: Evolutionary Relationship Between Platycerus Stag Beetles and Their Mycangium-Associated Yeast Symbionts
Source: Front Microbiol. 2020 Jun 30;11:1436. doi: 10.3389/fmicb.2020.01436 (PMC7338584; doi:10.3389/fmicb.2020.01436)
Supplement: Supplementary file 2 [file Data_Sheet_2.PDF]

SI Table 2. Yeast and insect samples examined.

Host insect species	Site	Host female no.	Yeast: Strain (number of clones examined) ITS	Yeast: Strain (number of clones examined) IGS	Yeast: GenBank accession no. ITS	Yeast: GenBank accession no. IGS	Insect sequence: GenBank accession no. *COI*
Platycerus delicatulus Lewis, 1883	2	W65		YW65.2(2)		LC438669	LC438728
	4	W10		YW10.1(2)		LC438670	(AB426945)**
	6	W52	YW52.1(1)	YW52.1(2)	(LC133297)*	(LC133308)*	(LC133287)*
	6	W55		YW55.1(2)		LC438671	
	20	W46		YW46.1(2)		LC438672	(AB426954)**
	25	W68		YW68.3(4)		LC438673	LC438729
	25	W72		YW72.2(4)		LC438674	
	26	W61		YW61.2(2)		LC438675	(AB609396)**
	27	W60	YW60.2(2)	YW60.2(2)	LC438646	LC438676	(AB426955)**
ssp. *unzendakensis* Fujita et Ichikawa, 1982	28	W47		YW47.2(3)		LC438677	LC438730
	28	W51		YW51.1(2)		same as LC438677	
P. kawadai Fujita et Ichikawa, 1982	6	W53	YW53.1(2)	YW53.1(2)	LC438647	LC438678	LC438731
	6	W56		YW56.1(2)		LC438679	
	7	W03		YW03.1(3)		LC438680	LC438732
	8	W12		YW12.1(3)		LC438681	LC438733
	8	W18	YW18.1(2)	YW18.1(2)	LC438648	LC438682	
	10	W70		YW70.3(4)		LC438683	LC438734
	10	W74		YW74.3(4)		LC438684	
P. acuticollis K. Kurosawa, 1969	2	W07	YW07.8(1)	YW07.8(6)	(LC133296)*	(LC133307)*	(LC133286)*
	5	W06	YW06.1(2)	YW06.1(4)	LC438649	LC438685	LC438735
P. albisomni Kubota, Kubota et Otobe, 2008	1	W19	YW19.1(2)	YW19.1(3)	LC438650	LC438686	(AB426970)**
	1	W21		YW21.1(3)		LC438687	
	9	W22		YW22.1(3)		LC438688	LC438736
	9	W23		YW23.1(2)		LC438689	
ssp. *chichibuensis* Kubota, Kubota et Otobe, 2008	3	W08		YW08.1(2)		LC438690	(AB573678)**
	4	W09		YW09.1(3)		LC438691	(AB426987)**
	4	W11		YW11.1(3)		LC438692	
P. takakuwai Fujita, 1987	6	W54	YW54.1(2)	YW54.1(2)	LC438651	LC438693	LC438737
	6	W57		YW57.1(2)		LC438694	
	7	W86		YW86.3(3)		LC438695	LC438738
	8	W13		YW13.1(3)		LC438696	LC438739
	8	W17		YW17.1(2)		LC438697	
	10	K01		YK01.1(8)		LC438698	
	10	K02		YK02.1(8)		LC438699	LC438740
	10	K03		YK03.1(8)		LC438700	
ssp. *akitai* Fujita, 1987	11	W69	No colony	No colony			(AB609474)**
	11	W73		YW73.3(4)		LC438701	
	12	W38		YW38.1(2)		LC438702	LC438741
	12	W64		YW64.1(2)		LC438703	
	14	W88		YW88.1(8)		LC438704	LC438742
	14	W89		YW89.1(8)		LC438705	
ssp. *namedai* Fujita, 1987	18	W43		YW43.2(2)		LC438706	(AB427025)**
	18	W82		YW82.3(4)		LC438707	
P. viridicuprus Kubota, Kubota et Otobe, 2008	15	W75		YW75.3(4)		LC438708	(AB427017)**
	15	W76		YW76.3(4)		LC438709	
	16	W04		YW04.1(3)		LC438710	LC438743
	16	W15	YW15.1(2)	YW15.1(2)	LC438652	LC438711	
	17	W78		YW78.3(4)		LC438712	(AB609531)**
	17	W79		YW79.3(4)		LC438713	
ssp. *kanadai* Kubota, Kubota et Otobe, 2008	23	W58		YW58.1(2)		LC438714	(AB427032)**
	23	W59		YW59.1(2)		LC438715	
	25	W87		YW87.1(8)		LC438716	LC438744
P. akitaorum Imura, 2007	13	W14		YW14.9(3)		LC438717	(AB427035)**
	13	W16	YW16.1(2)	YW16.1(2)	LC438653	LC438718	
P. sugitai Okuda et Fujita, 1987	19	W44	YW44.1(2)	YW44.1(2)	LC438654	LC438719	(AB588794)**
	19	W83		YW83.3(4)		LC438720	
	21	W81		YW81.3(4)		LC438721	(AB588806)**
	21	W84		YW84.3(4)		LC438722	
P. urushiyamai Imura, 2007	24	W48		YW48.1(2)		LC438723	(AB427045)**
	29	W49		YW49.2(2)		LC438724	LC438745
	30	W50	YW50.1(1)	YW50.1(2)	LC438655	LC438725	(AB609567)**
P. sue Imura, 2007	22	W45	YW45.1(2)	YW45.1(2)	LC438656	LC438726	(AB588778)**
	22	W80	YW80.3(2)	YW80.3(4)	LC438657	LC438727	
P. hongwonpyoi Imura et Choe, 1989		W36	YW36.1(1)	YW36.1(7)	(LC133288)*	(LC133299)*	(LC133280)*
(from South Korea)		W34	YW34.8(1)	YW34.8(5)	(LC133289)*	(LC133300)*	(LC133281)*
			YW34.2a(1)	YW34.2a(3)	(LC133290)*	(LC133301)*	
		W33	YW33.8(3)	YW33.8(8)	(LC133291)*	(LC133302)*	(LC133282)*
		W05	YW05.8(1)	YW05.8(8)	(LC133292)*	(LC133303)*	(LC133283)*
		W24	YW24.1(1)	YW24.1(4)	(LC133293)*	(LC133304)*	
		W37	YW37.8(1)	YW37.8(8)	(LC133294)*	(LC133305)*	(LC133284)*
		W35	YW35.8(1)	YW35.8(8)	(LC133295)*	(LC133306)*	(LC133285)*
P. oregonensis Westwood, 1844							(AB609586)**
Prismognathus angularis Waterhouse, 1874	13	W25	YW25.8(3)	YW25.8(8)	(LC133298)*	(LC133309)*	
Lucanus cervus (Linnaeus, 1758)					(LC120355)		

L. maculifemoratus Motsulsky, 1861	b	W28	YW28.1(2)	LC438658
	c	W31	YW31.1(2)	LC438659
Dorcus parallelipipedus (Linnaeus, 1758)				(LC120356)
D. striatipennis (Motchulsky, 1861)	b	W29	YW29.2(2)	LC438660
	c	W40	YW40.1(2)	LC438661
D. rubrofemoratus (Vollenhoven, 1865)	a	W27	YW27.1(2)	LC438662
	c	W41	YW41.1(2)	LC438663
D. rectus (Motchulsky, 1857)	d	W01	YW01.2(2)	LC438664
D. montivagus (Lewis, 1883)	b	W26	YW26.1(2)	LC438665
D. hopei binodulosus Waterhouse, 1874	c	W42	YW42.1(2)	LC438666
D. titanus pilifer Vollenhoven, 1861	e	W63	YW63.1(2)	LC438667
Figulus binodulus Waterhouse, 1873	f	W71	YW71.1(2)	LC438668
(Yeast Species)				
Sheffersomyces segobiensis (Santa Maria & C. Garcia)			JCM10740	(LC120358)
S. stipitis (Pignal)			CBS6054	(CP000497)
S. coipomensis (Ramirez & Gonzalez)			JCM8916	(LC120359)
Candida quercitrusa S. A. Meyer & Phaff				(AB013579)
Debaryomyces hansenii var. *hansenii* (Zopf) Lodder & Kreger-van Rij			MTCC_234	(AHBE01000021)

Accession numbers in parentheses have already been published in previous studies. *, from Tanahashi et al. (2017). **, from Kubota et al. (2010); Kubota & Kubota (2011); Kubota et al. (2011).
