# Supplementary material for: Evolutionary Relationship Between Platycerus Stag Beetles and Their Mycangium-Associated Yeast Symbionts
Source: Front Microbiol. 2020 Jun 30;11:1436. doi: 10.3389/fmicb.2020.01436 (PMC7338584; doi:10.3389/fmicb.2020.01436)
Supplement: Supplementary file 3 [file Data_Sheet_3.PDF]

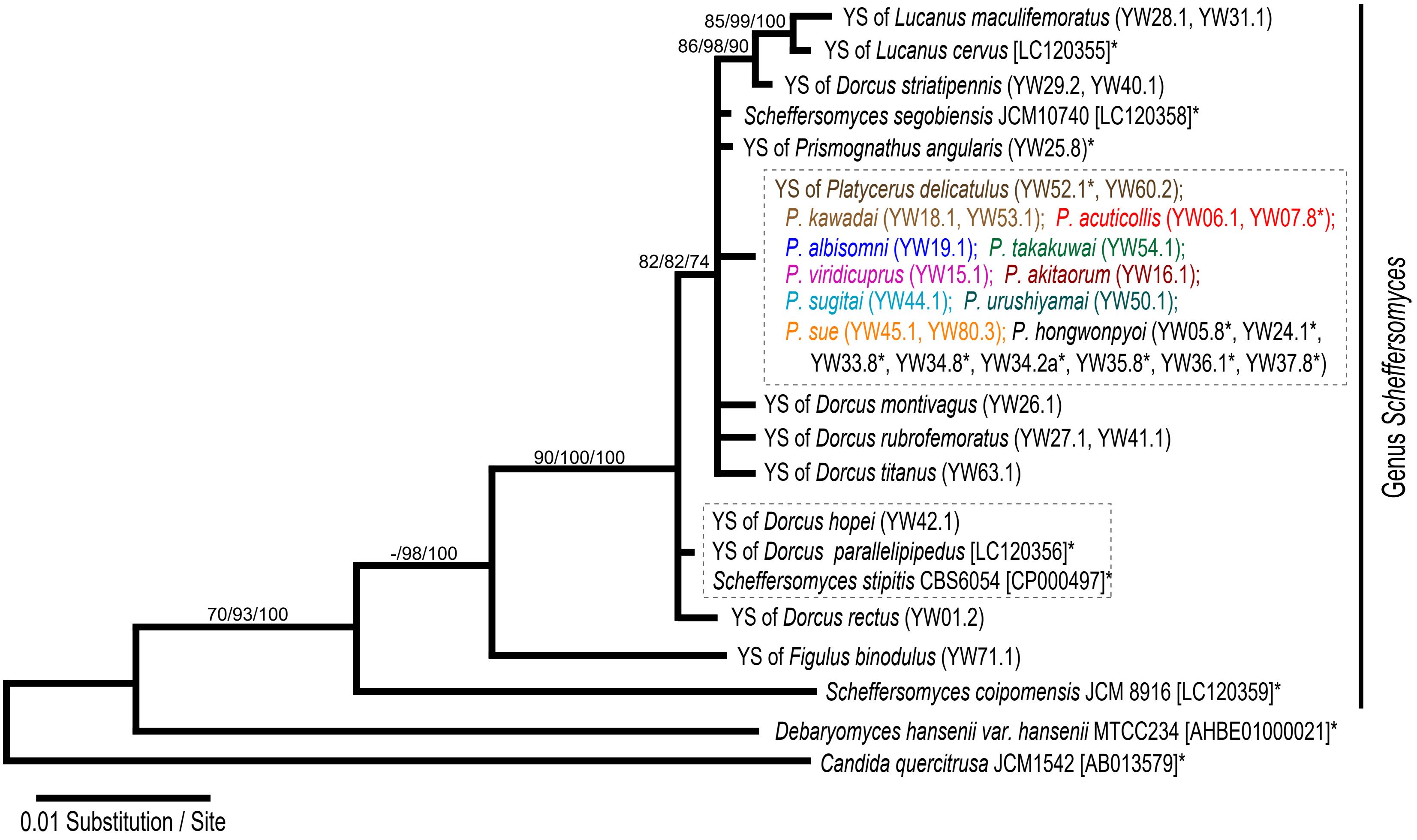

SI Figure 1. Bayesian inference (BI) phylogeny of the yeast symbionts of *Platycerus* stag beetles based on ITS sequences. Numbers near the branches indicate bootstrap probability for ML phylogeny (>50%)/posterior probability for BI phylogeny (>50%)/that recoding gaps (>50%). YS means yeast symbionts. Numbers following YW indicate the female code number and the strain number (see SI Table 2). Dotted boxes indicate ITS haplotypes shared by more than one species of the host beetles. Asterisks indicate the sequences from previous studies. HKY85 + I model (ML) and HKY + I model (BI) were selected as the best-fit substitution models by jModelTest ver. 2.1.7. ML and BI phylogenies are nearly identical to each other, although the branching order of the yeast symbiont of *Figulus binodulus* and *Scheffersomyces coipomensis* exhibited inconsistency. The BI phylogenies are identical regardless of the gap treatment.
