# Supplementary material for: Evolutionary Relationship Between Platycerus Stag Beetles and Their Mycangium-Associated Yeast Symbionts
Source: Front Microbiol. 2020 Jun 30;11:1436. doi: 10.3389/fmicb.2020.01436 (PMC7338584; doi:10.3389/fmicb.2020.01436)
Supplement: Supplementary file 4 [file Data_Sheet_4.PDF]

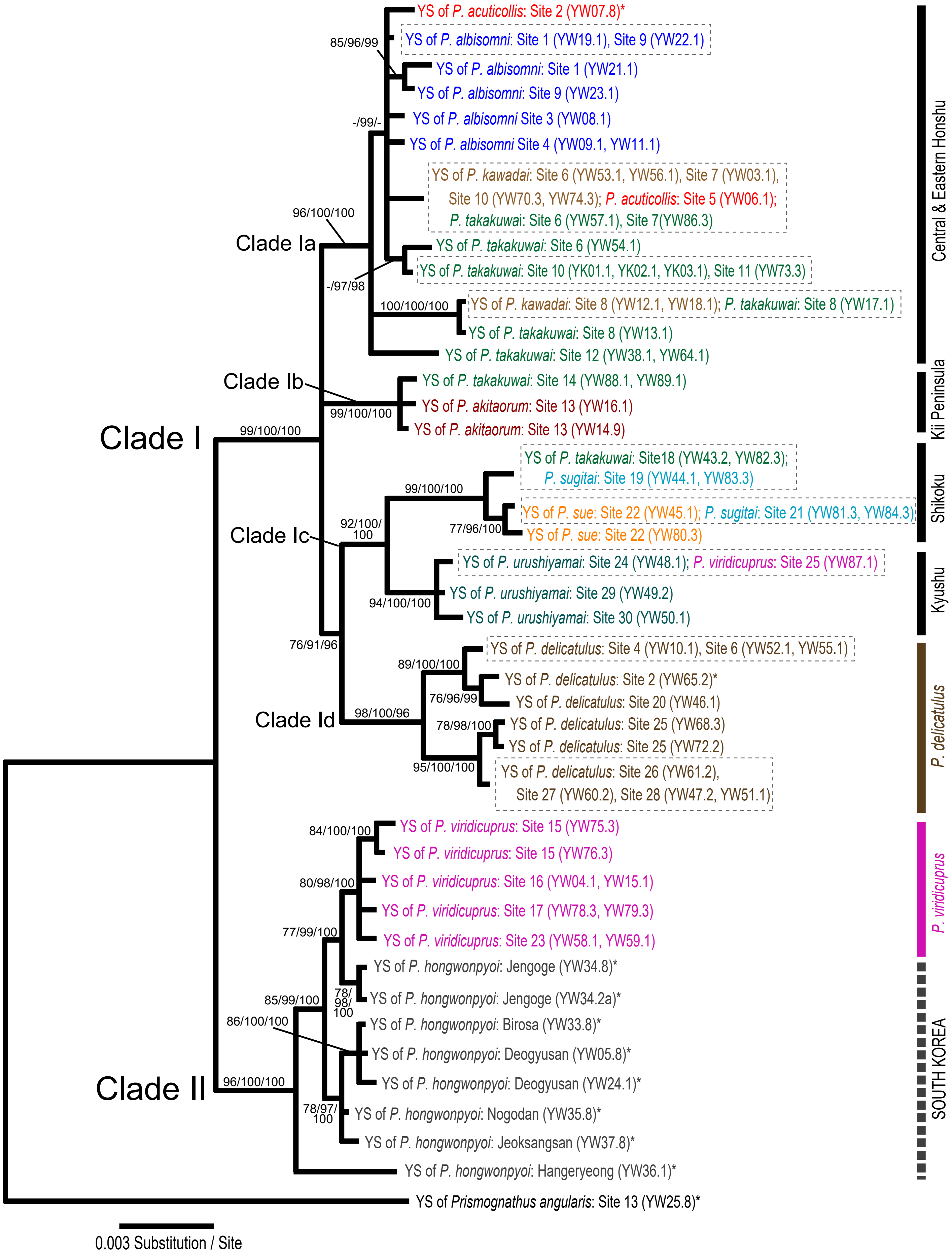

SI Figure 2. Bayesian inference (BI) phylogeny of the yeast symbionts of *Platycerus* stag beetles based on IGS sequences. Numbers at the nodes indicate bootstrap probability for ML phylogeny (>50%)/posterior probability for BI phylogeny (>50%)/that recoding the gaps (>50%). YS means yeast symbionts. Numbers following YW or YK indicate the female code number andthe strain number (see SI Table 2). Dotted boxes indicate IGS haplotypes shared by more than one species of the hoststag beetles. Asterisks indicate the sequences reported in previous studies. HKY85 + G model (ML) and HKY + G model (BI) were selected as the best-fit substitution models by jModelTest ver. 2.1.7. The ML and BI phylogenies are identical regardless of the gap treatment except for peripheral branches.
