# Supplementary material for: Evolutionary Relationship Between Platycerus Stag Beetles and Their Mycangium-Associated Yeast Symbionts
Source: Front Microbiol. 2020 Jun 30;11:1436. doi: 10.3389/fmicb.2020.01436 (PMC7338584; doi:10.3389/fmicb.2020.01436)
Supplement: Supplementary file 5 [file Data_Sheet_5.PDF]

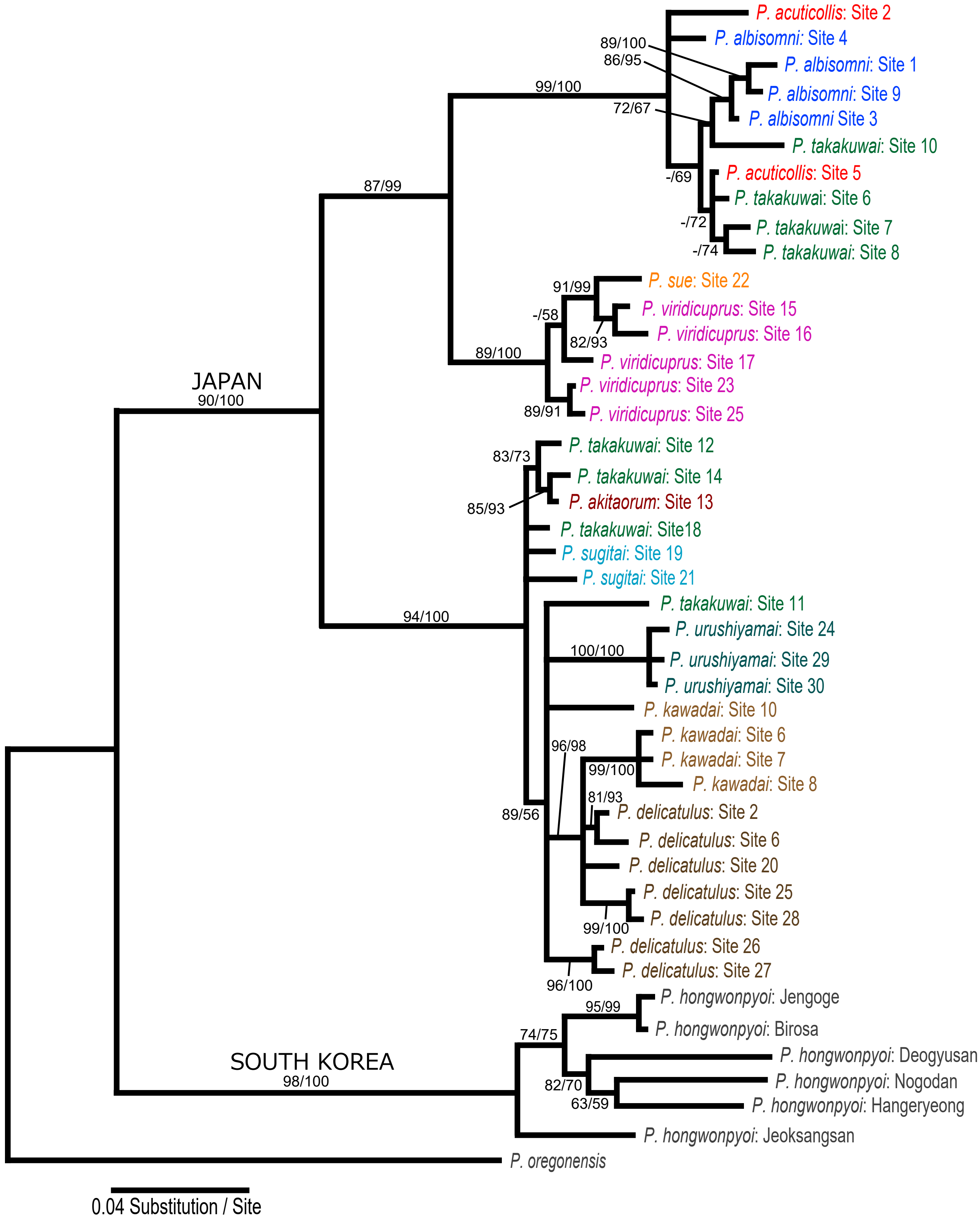

SI Figure 3. Bayesian inference (BI) phylogeny of *Platycerus* stag beetles based on *COI* gene sequences. *P. oregonensis* was treated as the outgroup (SI Table 2). Numbers at the nodes indicate bootstrap probability for maximum likelihood (ML) phylogeny (>50%)/posterior probability for Bayesian inference (BI) phylogeny (>50%). Asterisks indicate the sequences reported in previous studies. TN93 + G + I model (ML) and TrN + I + G model (BI) were selected as the best-fit substitution model by jModelTest ver. 2.1.7.
