# Supplementary material for: Evolutionary Relationship Between Platycerus Stag Beetles and Their Mycangium-Associated Yeast Symbionts
Source: Front Microbiol. 2020 Jun 30;11:1436. doi: 10.3389/fmicb.2020.01436 (PMC7338584; doi:10.3389/fmicb.2020.01436)
Supplement: Supplementary file 6 [file Data_Sheet_6.PDF]

SI Appendix 1. The sequence alignment used for the ITS phylogenetic analysis using ClustalW.

```
YS_D._rectus_YW01_2___________       GGAAGTAAAAGTCGTAACAAGGTTTCCGTAGGTGAACCTGCGGAAGGATC 50
YS_D._hopei_YW42_1____________       GGAAGTAAAAGTCGTAACAAGGTTTCCGTAGGTGAACCTGCGGAAGGATC 50
YS_L._maculifemoiratus_YW28_1_       GGAAGTAAAAGTCGTAACAAGGTTTCCGTAGGTGAACCTGCGGAAGGATC 50
YS_L.cervus___________________       GGAAGTAAAAGTCGTAACAAGGTTTCCGTAGGTGAACCTGCGGAAGGATC 50
YS_D._striatipennis_YW29_2           GGAAGTAAAAGTCGTAACAAGGTTTCCGTAGGTGAACCTGCGGAAGGATC 50
YS_P._angularis_YW25_1________       GGAAGTAAAAGTCGTAACAAGGTTTCCGTAGGTGAACCTGCGGAAGGATC 50
S._segobiensis________________       GGAAGTAAAAGTCGTAACAAGGTTTCCGTAGGTGAACCTGCGGAAGGATC 50
YS_P._sugitai_YW44_1__________       GGAAGTAAAAGTCGTAACAAGGTTTCCGTAGGTGAACCTGCGGAAGGATC 50
YS_D._titanus_YW63_1__________       GGAAGTAAAAGTCGTAACAAGGTTTCCGTAGGTGAACCTGCGGAAGGATC 50
YS_D._montivagus_YW26_1_______       GGAAGTAAAAGTCGTAACAAGGTTTCCGTAGGTGAACCTGCGGAAGGATC 50
YS_D._rubrofemoratus_YW27_1___       GGAAGTAAAAGTCGTAACAAGGTTTCCGTAGGTGAACCTGCGGAAGGATC 50
YS_F._binodulus_YW71_1________       GGAAGTAAAAGTCGTAACAAGGTTTCCGTAGGTGAACCTGCGGAAGGATC 50
S._coipomensis_______________        GGAAGTAAAAGTCGTAACAAGGTTTCCGTAGGTGAACCTGCGGAAGGATC 50
C._quercitrusa________________       GGAAGTAAAAGTCGTAACAAGGTTTCCGTAGGTGAACCTGCGGAAGGATC 50
D._hansenni_____________             GGAAGTAAAAGTCGTAACAAGGTTTCCGTAGGTGAACCTGCGGAAGGATC 50
                                     **************************************************

YS_D._rectus_YW01_2___________       ATTACAGTATTCTTTTTGCCAGCGCTTAACTGCGCGGCGAAAAAACCTTA 100
YS_D._hopei_YW42_1____________       ATTACAGTATTCTTTTTGCCAGCGCTTAACTGCGCGGCGAAAAAACCTTA 100
YS_L._maculifemoiratus_YW28_1_       ATTACAGTATTCTTTTTGCCAGCGCTTAACTGCGCGGCGAAAAAACCTTA 100
YS_L.cervus___________________       ATTACAGTATTCTTTTTGCCAGCGCTTAACTGCGCGGCGAAAAAACCTTA 100
YS_D._striatipennis_YW29_2           ATTACAGTATTCTTTTTGCCAGCGCTTAACTGCGCGGCGAAAAAACCTTA 100
YS_P._angularis_YW25_1________       ATTACAGTATTCTTTTTGCCAGCGCTTAACTGCGCGGCGAAAAAACCTTA 100
S._segobiensis________________       ATTACAGTATTCTTTTTGCCAGCGCTTAACTGCGCGGCGAAAAAACCTTA 100
YS_P._sugitai_YW44_1__________       ATTACAGTATTCTTTTTGCCAGCGCTTAACTGCGCGGCGAAAAAACCTTA 100
YS_D._titanus_YW63_1__________       ATTACAGTATTCTTTTTGCCAGCGCTTAACTGCGCGGCGAAAAAACCTTA 100
YS_D._montivagus_YW26_1_______       ATTACAGTATTCTTTTTGCCAGCGCTTAACTGCGCGGCGAAAAAACCTTA 100
YS_D._rubrofemoratus_YW27_1___       ATTACAGTATTCTTTTTGCCAGCGCTTAACTGCGCGGCGAAAAAACCTTA 100
YS_F._binodulus_YW71_1________       ATTACAGTATTCTTTTTGCCAGCGCTTAACTGCGCGGCGAAAAAACCTTA 100
S._coipomensis_______________        ATTACAGTATTAGTTTTACCTGCGCTTAATTGCGCGGTAAACAAAC-TTA 99
C._quercitrusa________________       ATTACAGTATTCTTTT-GCCAGCGCTTA-TTGCGCGGCGAAAAA-CCTTA 97
D._hansenni_____________             ATTACAGTATTCTTTTTGCCAGCGCTTAATTGCGCGGCGAAAAAACCTTA 100
                                     ***********  ***  ** *******  *******  ** ** * ***
```

```
YS_D._rectus_YW01_2___________          CACACAGTGTTTT-CTTTATTAGAA--ACTATTGCTTTGGTCTGGCTCAG 147
YS_D._hopei_YW42_1____________          CACACAGTGTTTT-CTTTATTAGAA--ACTATTGCTTTGGTCTGGCTCAG 147
YS_L._maculifemoiratus_YW28_1_          CACACAGTGTTTT-CTTTATTAGAA--ACTATTGCTTTGGTCTGGCTCAG 147
YS_L.cervus___________________          CACACAGTGTTTT-CTTTATTAGAA--ACTATTGCTTTGGTTTGGCTCAG 147
YS_D._striatipennis_YW29_2              CACACAGTGTTTT-CTTTATTAGAA--ACTATTGCTTTGGTTTGGCTCAG 147
YS_P._angularis_YW25_1________          CACACAGTGTTTT-CTTTATTAGAA--ACTATTGCTTTGGTTTGGCTCAG 147
S._segobiensis________________          CACACAGTGTTTT-CTTTATTAGAA--ACTATTGCTTTGGTTTGGCTCAG 147
YS_P._sugitai_YW44_1__________          CACACAGTGTTTT-CTTTATTAGAA--ACTATTGCTTTGGTTTGGCTCAG 147
YS_D._titanus_YW63_1__________          CACACAGTGTTTT-CTTTATTAGAA--ACTATTGCTTTGGTTTGGCTCAG 147
YS_D._montivagus_YW26_1_______          CACACAGTGTTTT-CTTTATTAGAA--ACTATTGCTTTGGTTTGGCTCAG 147
YS_D._rubrofemoratus_YW27_1___          CACACAGTGTTTT-CTTTATTAGAA--ACTATTGCTTTGGTTTGGCTCAG 147
YS_F._binodulus_YW71_1________          CACACAGTGTTTT-CTTTATTAGAA--ACTATTGCTTTGGTCTGGCCTAG 147
S._coipomensis_______________           CACACAATGTTTTTCTTTATTAGAA--ACTTTTGCTTTGGTCTGGCTTAG 147
C._quercitrusa________________          CACACAGTGATTTCTTTCTTTGAAAACA---TTGCTTTGGTCTGGCGCAA 144
D._hansenni_____________                CACACAGTGTTTTTTGTTATTACAAGAACTTTTGCTTTGGTCTGGACTAG 150
                                        ****** ** ***   *  **  **  *   ********** ***   *

YS_D._rectus_YW01_2___________          AAATGAGTTGGGCCAGAGGTTTACC-----AAACTTCAAT-TTT--ATTG 189
YS_D._hopei_YW42_1____________          AAATGAGTTGGGCCAGAGGTTTACC-----AAACTTCAAT-TTT--ATTG 189
YS_L._maculifemoiratus_YW28_1_          AAATGAGTTGGGCCAGAGGTTTACC-----AAACTTCAAT-TTT--ATTG 189
YS_L.cervus___________________          AAATGAGTTGGGCCAGAGGTTTACC-----AAACTTCAAT-TTT--ATTG 189
YS_D._striatipennis_YW29_2              AAATGAGTTGGGCCAGAGGTTTACC-----AAACTTCAAT-TTT--ATTG 189
YS_P._angularis_YW25_1________          AAATGAGTTGGGCCAGAGGTTTACC-----AAACTTCAAT-TTT--ATTG 189
S._segobiensis________________          AAATGAGTTGGGCCAGAGGTTTACC-----AAACTTCAAT-TTT--ATTG 189
YS_P._sugitai_YW44_1__________          AAATGAGTTGGGCCAGAGGTTTACC-----AAACTTCAAT-TTT--ATTG 189
YS_D._titanus_YW63_1__________          AAATGAGTTGGGCCAGAGGTTTACC-----AAACTTCAAT-TTT--ATTG 189
YS_D._montivagus_YW26_1_______          AAATGAGTTGGGCCAGAGGTTTACC-----AAACTTCAAT-TTT--ATTG 189
YS_D._rubrofemoratus_YW27_1___          AAATGAGTTGGGCCAGAGGTTTACT-----AAACTTCAAT-TTT--ATTG 189
YS_F._binodulus_YW71_1________          AAATAGGTTGGGCCAGAGGTTTACT-----AAACTTCAAT-TTT--ATTG 189
S._coipomensis_______________           AAATAAGTTGGGCCAGAGGTTTA-------AAACTTCAAT-TTTTAATTG 189
C._quercitrusa________________          G------TTGGGCCAAAGGTTTATT-----AAACTTCAAT-TTTATATTG 182
D._hansenni_____________                AAATAGTTTGGGCCAGAGGTTTACTGAACTAAACTTCAATATTTATATTG 200
                                               ******** *******       ********** ***  ****

YS_D._rectus_YW01_2___________          AATTGTTATTTTATTAATTTGTCAATTTGTTGATTAAATTCAAAAA-TCT 238
YS_D._hopei_YW42_1____________          AATTGTTATTTTATTAATTTGTCAATTTGTTGATTAAATTCAAAAA-TCT 238
```

```
YS_L._maculifemoiratus_YW28_1_      AATTGTTATTTTATTATTTTGTCAATTTGTTGATTAAATTCAAAAAATCT 239
YS_L.cervus___________________      AATTGTTATTTTATTATTTTGTCAATTTGTTGATTAAATTCAAAAAATCT 239
YS_D._striatipennis_YW29_2          AATTGTTATTTTATTAATTTGTCAATTTGTTGATTAAATTCAAAAA-TCT 238
YS_P._angularis_YW25_1________      AATTGTTATTTTATTAATTTGTCAATTTGTTGATTAAATTCAAAAA-TCT 238
S._segobiensis________________      AATTGTTATTTTATTAATTTGTCAATTTGTTGATTAAATTCAAAAA-TCT 238
YS_P._sugitai_YW44_1__________      AATTGTTATTTTATTAATTTGTCAATTTGTTGATTAAATTCAAAAA-TCT 238
YS_D._titanus_YW63_1__________      AATTGTTATTTTATTAATTTGTCAATTTGTTGATTAAATTCAAAAA-TCT 238
YS_D._montivagus_YW26_1_______      AATTGTTATTTTATTAATTTGTCAATTTGTTGATTAAATTCAAAAA-TCT 238
YS_D._rubrofemoratus_YW27_1___      AATTGTTATTTTATTAATTTGTCAATTTGTTGATTAAATTCAAAAA-TCT 238
YS_F._binodulus_YW71_1________      AATTGTTATTTTATATTATTGTCAATTTGTTGATTAAATTCAAAAA-TCT 238
S._coipomensis_______________       AATTGTTTATTTATTAT-TTGTCAATTTGTTGATTAAATTCAAAAA-TCT 237
C._quercitrusa________________      AACTGTTATTTTAACTAAA-GTCAATTTGTTGATTAAATTCAAAAA-TCT 230
D._hansenni_____________            AATTGTTATTT-ATTTAATTGTCAATTTGTTGATTAAATTCAAAAAATCT 249
                                    ** ****  ** *       ************************** ***

YS_D._rectus_YW01_2___________      TCAAAACTTTCAACAACGGATCTCTTGGTTCTCGCATCGATGAAGAACGC 288
YS_D._hopei_YW42_1____________      TCAAAACTTTCAACAACGGATCTCTTGGTTCTCGCATCGATGAAGAACGC 288
YS_L._maculifemoiratus_YW28_1_      TCAAAACTTTCAACAACGGATCTCTTGGTTCTCGCATCGATGAAGAACGC 289
YS_L.cervus___________________      TCAAAACTTTCAACAACGGATCTCTTGGTTCTCGCATCGATGAAGAACGC 289
YS_D._striatipennis_YW29_2          TCAAAACTTTCAACAACGGATCTCTTGGTTCTCGCATCGATGAAGAACGC 288
YS_P._angularis_YW25_1________      TCAAAACTTTCAACAACGGATCTCTTGGTTCTCGCATCGATGAAGAACGC 288
S._segobiensis________________      TCAAAACTTTCAACAACGGATCTCTTGGTTCTCGCATCGATGAAGAACGC 288
YS_P._sugitai_YW44_1__________      TCAAAACTTTCAACAACGGATCTCTTGGTTCTCGCATCGATGAAGAACGC 288
YS_D._titanus_YW63_1__________      TCAAAACTTTCAACAACGGATCTCTTGGTTCTCGCATCGATGAAGAACGC 288
YS_D._montivagus_YW26_1_______      TCAAAACTTTCAACAACGGATCTCTTGGTTCTCGCATCGATGAAGAACGC 288
YS_D._rubrofemoratus_YW27_1___      TCAAAACTTTCAACAACGGATCTCTTGGTTCTCGCATCGATGAAGAACGC 288
YS_F._binodulus_YW71_1________      TCAAAACTTTCAACAACGGATCTCTTGGTTCTCGCATCGATGAAGAACGC 288
S._coipomensis_______________       TCAAAACTTTCAACAACGGATCTCTTGGTTCTCGCATCGATGAAGAACGC 287
C._quercitrusa________________      TCAAAACTTTCAACAACGGATCTCTTGGTTCTCGCATCGATGAAGAACGC 280
D._hansenni_____________            TCAAAACTTTCAACAACGGATCTCTTGGTTCTCGCATCGATGAAGAACGC 299
                                    **************************************************

YS_D._rectus_YW01_2___________      AGCGAAATGCGATAAGTAATATGAATTGCAGATTTTCGTGAATCATCGAA 338
YS_D._hopei_YW42_1____________      AGCGAAATGCGATAAGTAATATGAATTGCAGATTTTCGTGAATCATCGAA 338
YS_L._maculifemoiratus_YW28_1_      AGCGAAATGCGATAAGTAATATGAATTGCAGATTTTCGTGAATCATCGAA 339
YS_L.cervus___________________      AGCGAAATGCGATAAGTAATATGAATTGCAGATTTTCGTGAATCATCGAA 339
```

```
YS_D._striatipennis_YW29_2        AGCGAAATGCGATAAGTAATATGAATTGCAGATTTTCGTGAATCATCGAA 338
YS_P._angularis_YW25_1________    AGCGAAATGCGATAAGTAATATGAATTGCAGATTTTCGTGAATCATCGAA 338
S._segobiensis________________    AGCGAAATGCGATAAGTAATATGAATTGCAGATTTTCGTGAATCATCGAA 338
YS_P._sugitai_YW44_1__________    AGCGAAATGCGATAAGTAATATGAATTGCAGATTTTCGTGAATCATCGAA 338
YS_D._titanus_YW63_1__________    AGCGAAATGCGATAAGTAATATGAATTGCAGATTTTCGTGAATCATCGAA 338
YS_D._montivagus_YW26_1_______    AGCGAAATGCGATAAGTAATATGAATTGCAGATTTTCGTGAATCATCGAA 338
YS_D._rubrofemoratus_YW27_1___    AGCGAAATGCGATAAGTAATATGAATTGCAGATTTTCGTGAATCATCGAA 338
YS_F._binodulus_YW71_1________    AGCGAAATGCGATAAGTAATATGAATTGCAGATTTTCGTGAATCATCGAA 338
S._coipomensis_______________     AGCGAAATGCGATAAGTAATATGAATTGCAGATTTTCGTGAATCATCGAA 337
C._quercitrusa________________    AGCGAATTGCGATAAGTAATATGAATTGCAGATTTTCGTGAATCATCGAA 330
D._hansenni_____________          AGCGAAATGCGATAAGTAATATGAATTGCAGATTTTCGTGAATCATCGAA 349
                                  ****** *******************************************

YS_D._rectus_YW01_2___________    TCTTTGAACGCACATTGCGCCCTTTGGTATTCCAAAGGGCATGCCTGTTT 388
YS_D._hopei_YW42_1____________    TCTTTGAACGCACATTGCGCCCTTTGGTATTCCAAAGGGCATGCCTGTTT 388
YS_L._maculifemoiratus_YW28_1_    TCTTTGAACGCACATTGCGCCCTTTGGTATTCCAAAGGGCATGCCTGTTT 389
YS_L.cervus___________________    TCTTTGAACGCACATTGCGCCCTTTGGTATTCCAAAGGGCATGCCTGTTT 389
YS_D._striatipennis_YW29_2        TCTTTGAACGCACATTGCGCCCTTTGGTATTCCAAAGGGCATGCCTGTTT 388
YS_P._angularis_YW25_1________    TCTTTGAACGCACATTGCGCCCTTTGGTATTCCAAAGGGCATGCCTGTTT 388
S._segobiensis________________    TCTTTGAACGCACATTGCGCCCTTTGGTATTCCAAAGGGCATGCCTGTTT 388
YS_P._sugitai_YW44_1__________    TCTTTGAACGCACATTGCGCCCTTTGGTATTCCAAAGGGCATGCCTGTTT 388
YS_D._titanus_YW63_1__________    TCTTTGAACGCACATTGCGCCCTTTGGTATTCCAAAGGGCATGCCTGTTT 388
YS_D._montivagus_YW26_1_______    TCTTTGAACGCACATTGCGCCCTTTGGTATTCCAAAGGGCATGCCTGTTT 388
YS_D._rubrofemoratus_YW27_1___    TCTTTGAACGCACATTGCGCCCTTTGGTATTCCAAAGGGCATGCCTGTTT 388
YS_F._binodulus_YW71_1________    TCTTTGAACGCACATTGCGCCCTTTGGCATTCCAAAGGGCATGCCTGTTT 388
S._coipomensis_______________     TCTTTGAACGCACATTGCGCCCTTTGGTATTCCAAAGGGCATGCCTGTTT 387
C._quercitrusa________________    TCTTTGAACGCACATTGCGCCCTTTGGTATTCCAAAGGGCATGCCTGTTT 380
D._hansenni_____________          TCTTTGAACGCACATTGCGCCCTCTGGTATTCCAGAGGGCATGCCTGTTT 399
                                  *********************** *** ****** ***************

YS_D._rectus_YW01_2___________    GAGCGTCATTTCTCTCTCAAACCCTCGGGTTTGGTATTGAGTGATACTCT 438
YS_D._hopei_YW42_1____________    GAGCGTCATTTCTCTCTCAAACCCTCGGGTTTGGTATTGAGTGATACTCT 438
YS_L._maculifemoiratus_YW28_1_    GAGCGTCATTTCTCTCTCAAACCCTCGGGTTTGGTATTGAGTGATACTCT 439
YS_L.cervus___________________    GAGCGTCATTTCTCTCTCAAACCCTCGGGTTTGGTATTGAGTGATACTCT 439
YS_D._striatipennis_YW29_2        GAGCGTCATTTCTCTCTCAAACCCTCGGGTTTGGTATTGAGTGATACTCT 438
YS_P._angularis_YW25_1________    GAGCGTCATTTCTCTCTCAAACCCTCGGGTTTGGTATTGAGTGATACTCT 438
```

```
S._segobiensis_______________     GAGCGTCATTTCTCTCTCAAACCCTCGGGTTTGGTATTGAGTGATACTCT 438
YS_P._sugitai_YW44_1_________     GAGCGTCATTTCTCTCTCAAACCCTCGGGTTTGGTATTGAGTGATACTCT 438
YS_D._titanus_YW63_1_________     GAGCGTCATTTCTCTCTCAAACCCTCGGGTTTGGTATTGAGTGATACTCT 438
YS_D._montivagus_YW26_1_______    GAGCGTCATTTCTCTCTCAAACCCTCGGGTTTGGTATTGAGTGATACTCT 438
YS_D._rubrofemoratus_YW27_1___    GAGCGTCATTTCTCTCTCAAACCCTCGGGTTTGGTATTGAGTGATACTCT 438
YS_F._binodulus_YW71_1_______     GAGCGTCATTTCTCTCTCAAACCCTCGGGTTTGGTATTGAGTGATACTCT 438
S._coipomensis______________      GAGCGTCATTTCTCTCTCAAACCTTCGGGTTTGGTATTGAGTGATACTCT 437
C._quercitrusa_______________     GAGCGTCATTTCTCTCTCAAATCTTCGGATTTGGTTTTGAGTGATACTCT 430
D._hansenni____________           GAGCGTCATTTCTCTCTCAAACCTTCGGGTTTGGTATTGAGTGATACTCT 449
                                  ********************* * **** ****** **************

YS_D._rectus_YW01_2__________     TAGTCGAACTAGGCGTTTGCTTGAAAAGTATTGGCACGAGTGGTACTAAA 488
YS_D._hopei_YW42_1___________     TAGTCGAACTAGGCGTTTGCTTGAAAAGTATTGGCACGAGTGGTACTAAA 488
YS_L._maculifemoiratus_YW28_1_    TAGTCGAACTAGGCGTTTGCTTGAAAAGTATTGGCACGAGTGGTACTAAA 489
YS_L.cervus__________________     TAGTCGAACTAGGCGTTTGCTTGAAAAGTATTGGCACGAGTGGTACTAAA 489
YS_D._striatipennis_YW29_2        TAGTCGAACTAGGCGTTTGCTTGAAAAGTATTGGCACGAGTGGTACTAAA 488
YS_P._angularis_YW25_1_______     TAGTC-AACTAGGCGTTTGCTTGAAAAGTATTGGCACGAGTGGTACTAAA 487
S._segobiensis_______________     TAGTCGAACTAGGCGTTTGCTTGAAAAGTATTGGCACGAGTGGTACTAAA 488
YS_P._sugitai_YW44_1_________     TAGTTGAACTAGGCGTTTGCTTGAAAAGTATTGGCACGAGTGGTACTAAA 488
YS_D._titanus_YW63_1_________     TAGTCGAACTAGGCGTTTGCTTGAAAAGTATTGGCACGAGTGGTACTAAA 488
YS_D._montivagus_YW26_1_______    TAGTCGAACTAGGCGTTTGCTTGAAAAGTATTGGCACGAGTGGTACTGAA 488
YS_D._rubrofemoratus_YW27_1___    TAGTCGAACTAGGCGTTTGCTTGAAAAGTATTGGCACGAGTGGTACTAAA 488
YS_F._binodulus_YW71_1_______     TAGTCGAACTAGGCGTTTGCTTGAAAAGTATTGGCACGAGTGGTACTAAA 488
S._coipomensis______________      TAGTCGAACTAGGCGTTTGCTTGAAAAGTATTGGCACGAGTCGTACTAAA 487
C._quercitrusa_______________     TAGTCGGACTAGGCGTTTGCTTGAAAAGTATTGGCAAGAGTGGTACTTTA 480
D._hansenni____________           TAGTCGAACTAGGCGTTTGCTTGAAATGTATTGGCATGAGTGGTACTGGA 499
                                  ****   ******************* ********* **** *****  *

YS_D._rectus_YW01_2__________     TAGTACTGAC-AGAATATTT-CAATGTATTAGGTTTATCCAACTCGTTGA 536
YS_D._hopei_YW42_1___________     TAGTACTGAC-AGAATATTT-CAATGTATTAGGTTTATCCAACTCGTTGA 536
YS_L._maculifemoiratus_YW28_1_    TAGTACTGAC-AGAATATTT-CAATGTATTAGGTTTATCCAACTCGTTGA 537
YS_L.cervus__________________     TAGTACTGAC-AGAATATTT-CAATGTATTAGGTTTATCCAACTCGTTGA 537
YS_D._striatipennis_YW29_2        TAGTACTGAC-AGAATATTT-CAATGTATTAGGTTTATCCAACTCGTTGA 536
YS_P._angularis_YW25_1_______     TAGTACTGAC-AGAATATTT-CAATGTATTAGGTTTATCCAACTCGTTGA 535
S._segobiensis_______________     TAGTACTGAC-AGAATATTT-CAATGTATTAGGTTTATCCAACTCGTTGA 536
YS_P._sugitai_YW44_1_________     TAGTACTGAC-AGAATATTT-CAATGTATTAGGTTTATCCAACTCGTTGA 536
```

```
YS_D._titanus_YW63_1_________        TAGTACTGAC-AGAATATTT-CAATGTATTAGGTTTATCCAACTCGTTGA 536
YS_D._montivagus_YW26_1_______       TAGTACTGAC-AGAATATTT-CAATGTATTAGGTTTATCCAACTCGTTGA 536
YS_D._rubrofemoratus_YW27_1___       TAGTACTGAC-AGAATATTT-CAATGTATTAGGTTTATCCAACTCGTTGA 536
YS_F._binodulus_YW71_1________       TAGTGCTGACTGGAATATTT-CAATGTATTAGGTTTATCCAACTCGTTGA 537
S._coipomensis_______________        TAGTGCTTTCAGGAACATTTTCAATGTATTAGGTTTATCCAACTCGTTGA 537
C._quercitrusa________________       -GGTGCT----AAACTG-TTTCAATGTATTAGGTTTATCCAACTCGTTGA 524
D._hansenni_____________             TAGTGCT----ATATGACTTTCAATGTATTAGGTTTATCCAACTCGTTGA 545
                                       ** **      *    ** *****************************

YS_D._rectus_YW01_2___________       T--ACTTC-TGGCGGTGAATTTTT-GGTATATT-GGCTTTGCCTTACAAA 581
YS_D._hopei_YW42_1____________       G--ACTTC-TGGCGGTGAATTTTT-GGTATATT-GGCTTTGCCTTACAAA 581
YS_L._maculifemoiratus_YW28_1_       G--ACTTC-TGGCGGTGAATTTTT-GGTATATT-TGCTTTGCCTTACAAA 582
YS_L.cervus___________________       G--ACTTC-TGGCGGTGAATTTTT-GGTATATT-TGCTTTGCCTTACAAA 582
YS_D._striatipennis_YW29_2           G--ACTTC-TGGCGGTGAATTTTT-GGTATATT-TGCTTTGCCTTACAAA 581
YS_P._angularis_YW25_1________       G--ACTTC-TGGCGGTGAATTTTT-GGTATATT-GGCTTTGCCTTACAAA 580
S._segobiensis________________       G--ACTTC-TGGCGGTGAATTTTT-GGTATATT-GGCTTTGCCTTACAAA 581
YS_P._sugitai_YW44_1__________       G--ACTTC-TGGCGGTGAATTTTT-GGTATATT-GGCTTTGCCTTACAAA 581
YS_D._titanus_YW63_1_________        G--TCTTC-TGGCGGTGAATTTTT-GGTATATT-GGCTTTGCCTTACAAA 581
YS_D._montivagus_YW26_1_______       G--ACTTC-TGGCGGTGAATTTTT-GGTATATT-GGCTTTGCCTTACAAA 581
YS_D._rubrofemoratus_YW27_1___       G--ACTTC-TGGCGGTGAATTTTT-GGTATATT-GGCTTTGCCTTACAAA 581
YS_F._binodulus_YW71_1________       G--ACTTCCTCTCGGTGAATTTTT-GGTATAGT-GGCTTTGCCTTACAAA 583
S._coipomensis_______________        G--AATTCCAGTTAGTGAATTTTT-GGTATATTTGGCTTTGCCTTACAAA 584
C._quercitrusa________________       ATGAGGTT--AGT--TACATTATT--GTGCT-TAGGCTCGGCCTTACAAC 567
D._hansenni_____________             AT-AGTTT--AATGGTATATTTCTCGGTATTCTAGGCTCGGCCTTACAAT 592
                                           *        *  ***  *  **    *  ***  *********

YS_D._rectus_YW01_2___________       ACAACAAACAA-GTTTGACCTCAAATCAGGTAGGATTACCCGCTGAACTT 630
YS_D._hopei_YW42_1____________       ACAACAAACAA-GTTTGACCTCAAATCAGGTAGGATTACCCGCTGAACTT 630
YS_L._maculifemoiratus_YW28_1_       ACAACAAACAA-GTTTGACCTCAAATCAGGTAGGATTACCCGCTGAACTT 631
YS_L.cervus___________________       ACAACAAACAA-GTTTGACCTCAAATCAGGTAGGATTACCCGCTGAACTT 631
YS_D._striatipennis_YW29_2           ACAACAAACAA-GTTTGACCTCAAATCAGGTAGGATTACCCGCTGAACTT 630
YS_P._angularis_YW25_1________       ACAACAAACAA-GTTTGACCTCAAATCAGGTAGGATTACCCGCTGAACTT 629
S._segobiensis________________       ACAACAAACAA-GTTTGACCTCAAATCAGGTAGGATTACCCGCTGAACTT 630
YS_P._sugitai_YW44_1__________       ACAACAAACAA-GTTTGACCTCAAATCAGGTAGGATTACCCGCTGAACTT 630
YS_D._titanus_YW63_1_________        ACAACAAACAA-GTTTGACCTCAAATCAGGTAGGATTACCCGCTGAACTT 630
YS_D._montivagus_YW26_1_______       ACAACAAACAA-GTTTGACCTCAAATCAGGTAGGATTACCCGCTGAACTT 630
```

```
YS_D._rubrofemoratus_YW27_1___      ACAACAAACAA-GTTTGACCTCAAATCAGGTAGGATTACCCGCTGAACTT 630
YS_F._binodulus_YW71_1________      ACAACAAACAA-GTTTGACCTCAAATCAGGTAGGATTACCCGCTGAACTT 632
S._coipomensis_______________       ACAACAAACAA-GTTTGACCTCAAATCAGGTAGGACTACCCGCTGAACTT 633
C._quercitrusa________________      A--ACAAACAAAGTTTGACCTCAAATCAGGTAGGATTACCCGCTGAACTT 615
D._hansenni_____________            ATAACAAACAA-GTTTGACCTCAAATCAGGTAGGATTACCCGCTGAACTT 641
                                    *  ******** *********************** **************

YS_D._rectus_YW01_2___________      AAGCATATCAATAAGCGGAGGA 652
YS_D._hopei_YW42_1____________      AAGCATATCAATAAGCGGAGGA 652
YS_L._maculifemoiratus_YW28_1_      AAGCATATCAATAAGCGGAGGA 653
YS_L.cervus___________________      AAGCATATCAATAAGCGGAGGA 653
YS_D._striatipennis_YW29_2          AAGCATATCAATAAGCGGAGGA 652
YS_P._angularis_YW25_1________      AAGCATATCAATAAGCGGAGGA 651
S._segobiensis________________      AAGCATATCAATAAGCGGAGGA 652
YS_P._sugitai_YW44_1__________      AAGCATATCAATAAGCGGAGGA 652
YS_D._titanus_YW63_1__________      AAGCATATCAATAAGCGGAGGA 652
YS_D._montivagus_YW26_1_______      AAGCATATCAATAAGCGGAGGA 652
YS_D._rubrofemoratus_YW27_1___      AAGCATATCAATAAGCGGAGGA 652
YS_F._binodulus_YW71_1________      AAGCATATCAATAAGCGGAGGA 654
S._coipomensis_______________       AAGCATATCAATAAGCGGAGGA 655
C._quercitrusa________________      AAGCATATCAATAAGCGGAGGA 637
D._hansenni_____________            AAGCATATCAATAAGCGGAGGA 663
                                    **********************
```
