# Supplementary material for: Evolutionary Relationship Between Platycerus Stag Beetles and Their Mycangium-Associated Yeast Symbionts
Source: Front Microbiol. 2020 Jun 30;11:1436. doi: 10.3389/fmicb.2020.01436 (PMC7338584; doi:10.3389/fmicb.2020.01436)
Supplement: Supplementary file 7 [file Data_Sheet_7.PDF]

SI Appendix 2. The sequence alignment used for the IGS phylogenetic analysis using ClustalW.

```
YS_P._viridicuprus_YW75_3___       TGAGATTAAGCTTCAGTTGTCCGATTTGTTTGTGTTACACAACACAATCT 50
YS_P._viridicuprus_YW76_3___       TGAGATTAAGCTTCAGTTGTCCGATTTGTTTGTGTTACACAACACAATCT 50
YS_P._viridicuprus_YW78_3___       TGAGATTAAGCTTCAGTTGTCCGATTTGTTTGTGTTACACAACACAATCT 50
YS_P._viridicuprus_YW58_1___       TGAGATTAAGCTTCAGTTGTCCGATTTGTTTGTGTTACACAACACAATCT 50
YS_P._viridicuprus_YW04_1___       TGAGATTAAGCTTCAGTTGTCCGATTTGTTTGTGTTACACAACACAATCT 50
YS_P._hongwonpyoi_YW34_8____       TGAGATTAAGCTTCAGTTGTCCGATTTGTTTGTGTTACACAACACAATCT 50
YS_P._hongwonpyoi_YW34_2a___       TGAGATTAAGCTTCAGTTGTCCGATTTGTTTGTGTTACACAACACAATCT 50
YS_P._hongwonpyoi_YW33_8____       TGAGATTAAGCTTCAGTTGTCCGATTTGTTTGTGTTACACAACACAATCT 50
YS_P._hongwonpyoi_YW24_1____       TGAGATTAAGCTTCAGTTGTCCGATTTGTTTGTGTTACACAACACAATCT 50
YS_P._hongwonpyoi_YW05_8____       TGAGATTAAGCTTCAGTTGTCCGATTTGTTTGTGTTACACAACACAATCT 50
YS_P._hongwonpyoi_YW35_8___        TGAGATTAAGCTTCAGTTGTCCGATTTGTTTGTGTTACACAACACAATCT 50
YS_P._hongwonpyoi_YW37_8____       TGAGATTAAGCTTCAGTTGTCCGATTTGTTTGTGTTACACAACACAATCT 50
YS_P._hongwonpyoi_YW36_1____       TGAGATTAAGCTTCAGTTGTCCGATTTGTTTGTGTTACACAACACAATCT 50
YS_P._sue_YW45_1____________       TGAGATTAAGCTTCAGTTGTCCGATTTGTTTGTGTTACACAACACAATCT 50
YS_P._sue_YW80_3____________       TGAGATTAAGCTTCAGTTGTCCGATTTGTTTGTGTTACACAACACAATCT 50
YS_P._takakuwai_YW43_2______       TGAGATTAAGCTTCAGTTGTCCGATTTGTTTGTGTTACACAACACAATCT 50
YS_P._urushiyamai_YW48_1____       TGAGATTAAGCTTCAGTTGTCCGATTTGTTTGTGTTACACAACACAATCT 50
YS_P._urushiyamai_YW50_1____       TGAGATTAAGCTTCAGTTGTCCGATTTGTTTGTGTTACACAACACAATCT 50
YS_P._urushiyamai_YW49_2____       TGAGATTAAGCTTCAGTTGTCCGATTTGTTTGTGTTACACAACACAATCT 50
YS_P._delicatulus_YW65_2____       TGAGATTAAGCTTCAGTTGTCCGATTTGTTTGTGTTACACAACACAATCT 50
YS_P._delicatulus_YW46_1____       TGAGATTAAGCTTCAGTTGTCCGATTTGTTTGTGTTACACAACACAATCT 50
YS_P._delicatulus_YW10_1____       TGAGATTAAGCTTCAGTTGTCCGATTTGTTTGTGTTACACAACACAATCT 50
YS_P._delicatulus_YW68_3____       TGAGATTAAGCTTCAGTTGTCCGATTTGTTTGTGTTACACAACACAATCT 50
YS_P._delicatulus_YW72_2____       TGAGATTAAGCTTCAGTTGTCCGATTTGTTTGTGTTACACAACACAATCT 50
YS_P._delicatulus_YW47_2____       TGAGATTAAGCTTCAGTTGTCCGATTTGTTTGTGTTACACAACACAATCT 50
YS_P._takakuwai_YW88_1______       TGAGATTAAGCTTCAGTTGTCCGATTTGTTTGTGTTACACAACACAATCT 50
YS_P._akitaorum_YW16_1______       TGAGATTAAGCTTCAGTTGTCCGATTTGTTTGTGTTACACAACACAATCT 50
YS_P._akitaorum_YW14_2______       TGAGATTAAGCTTCAGTTGTCCGATTTGTTTGTGTTACACAACACAATCT 50
YS_P._kawadai_YW12_1______         TGAGATTAAGCTTCAGTTGTCCGATTTGTTTGTGTTATACAACACAATCT 50
YS_P._takakuwai_YW13_1______       TGAGATTAAGCTTCAGTTGTCCGATTTGTTTGTGTTATACAACACAATCT 50
YS_P._albisomni_YW19_1______       TGAGATTAAGCTTCAGTTGTCCGATTTGTTTGTGTTACACAACACAATCT 50
YS_P._albisomni_YW21_1______       TGAGATTAAGCTTCAGTTGTCCGATTTGTTTGTGTTACACAACACAATCT 50
YS_P._albisomni_YW23_1______       TGAGATTAAGCTTCAGTTGTCCGATTTGTTTGTGTTACACAACACAATCT 50
YS_P._kawadai_YW03_1________       TGAGATTAAGCTTCAGTTGTCCGATTTGTTTGTGTTACACAACACAATCT 50
```

```
YS_P._takakuwai_YW54_1______        TGAGATTAAGCTTCAGTTGTCCGATTTGTTTGTGTTACACAACACAATCT 50
YS_P._takakuwai_YW73_3______        TGAGATTAAGCTTCAGTTGTCCGATTTGTTTGTGTTACACAACACAATCT 50
YS_P._acuticollis_YW07_8____        TGAGATTAAGCTTCAGTTGTCCGATTTGTTTGTGTTACACAACACAATCT 50
YS_P._albisomni_YW08_1______        TGAGATTAAGCTTCAGTTGTCCGATTTGTTTGTGTTACACAACACAATCT 50
YS_P._albisomni_YW09_1______        TGAGATTAAGCTTCAGTTGTCCGATTTGTTTGTGTTACACAACACAATCT 50
YS_P._takakuwai_YW38_1______        TGAGATTAAGCTTCAGTTGTCCGATTTGTTTGTGTTACATAACACACTCT 50
YS_P._angularis_YW25_8______        TGAGATTAAGCTTCAGTTGTCCGATTTGTTTGTGTTACACAACACAATCT 50
                                    ************************************* * ****** ***

YS_P._viridicuprus_YW75_3___        CCTCTAAGTGATAGTTGGCAGGCGCTAACTAGTATTTAGAGGAGTTTT-A 99
YS_P._viridicuprus_YW76_3___        CCTCTAAGTGATAGTTGGCAGGCGCTAACTAGTATTTAGAGGAGTTTT-A 99
YS_P._viridicuprus_YW78_3___        CCTCTAAGTGATAGTTGGCAGGCGCTAACTAGTATTTAGAGGAGTTTT-A 99
YS_P._viridicuprus_YW58_1___        CCTCTAAGTGATAGTTGGCAGGCGCTAACTAGTATTTAGAGGAGTTTT-A 99
YS_P._viridicuprus_YW04_1___        CCTCTAAGTGATAGTTGGCAGGCGCTAACTAGTATTTAGAGGAGTTTT-A 99
YS_P._hongwonpyoi_YW34_8____        CCTCTAAGTGATAGTTGGCAGGCGCTAACTAGTATTTAGAGGAGTTTT-A 99
YS_P._hongwonpyoi_YW34_2a___        CCTCTAAGTGATAGTTGGCAGGCGCTAACTAGTATTTAGAGGAGTTTT-A 99
YS_P._hongwonpyoi_YW33_8____        CCTCTAAGTGATAGTTGGCAGGCGCTAACTAGTATTTAGAGGAGTTTT-A 99
YS_P._hongwonpyoi_YW24_1____        CCTCTAAGTGATAGTTGGCAGGCGCTAACTAGTATTTAGAGGAGTTTT-A 99
YS_P._hongwonpyoi_YW05_8____        CCTCTAAGTGATAGTTGGCAGGCGCTAACTAGTATTTAGAGGAGTTTT-A 99
YS_P._hongwonpyoi_YW35_8___         CCTCTAAGTGATAGTTGGCAGGCGCTAACTAGTATTTAGAGGAGTTTT-A 99
YS_P._hongwonpyoi_YW37_8____        CCTCTAAGTGATAGTTGGCAGGCGCTAACTAGTATTTAGAGGAGTTTT-A 99
YS_P._hongwonpyoi_YW36_1____        CCTCTAAGTGATAGTTGGCAGGCGCTAACTAGTATTTAGAGGAGTTTT-A 99
YS_P._sue_YW45_1____________        CCTCTAAGTGATAGTTGGCAGGCGCTAACTAGTATTTAGAGGAGTTTT-A 99
YS_P._sue_YW80_3____________        CCTCTAAGTGATAGTTGGCAGGCGCTAACTAGTATTTAGAGGAGTTTT-A 99
YS_P._takakuwai_YW43_2______        CCTCTAAGTGATAGTTGGCAGGCGCTAACTAGTATTTAGAGGAGTTTT-A 99
YS_P._urushiyamai_YW48_1____        CCTCTAAGTGATAGTTGGCAGGTGCTAACTAGTATTTAGAGGAGTTTT-A 99
YS_P._urushiyamai_YW50_1____        CCTCTAAGTGATAGTTGGCAGGTGCTAACTAGTATTTAGAGGAGTTTT-A 99
YS_P._urushiyamai_YW49_2____        CCTCTAAGTGATAGTTGGCAGGTGCTAACTAGTATTTAGAGGAGTTTT-A 99
YS_P._delicatulus_YW65_2____        CCTCTAAGTGATAGTTGGCAGGCGCTAGCTAGTATTTAGAGGAGTTTT-A 99
YS_P._delicatulus_YW46_1____        CCTCTAAGTGATAGTTGGCAGGCGCTAGCTAGTATTTAGAGGAGTTTT-A 99
YS_P._delicatulus_YW10_1____        CCTCTAAGTGATAGTTGGCAGGCGCTAGCTAGTATTTAGAGGAGTTTT-A 99
YS_P._delicatulus_YW68_3____        CCTCTAAGTGATAGTTGGCAGGCGCTAGCTAGTATTTAGAGGAGTTTT-A 99
YS_P._delicatulus_YW72_2____        CCTCTAAGTGATAGTTGGCAGGCGCTAGCTAGTATTTAGAGGAGTTTT-A 99
YS_P._delicatulus_YW47_2____        CCTCTAAGTGATAGTTGGCAGGCGCTAGCTAGTATTTAGAGGAGTTTT-A 99
YS_P._takakuwai_YW88_1______        CCTCTAAGTGATAGTTGGCAGGCGCTAACTAGTATTTAGAGGAGTTTT-A 99
YS_P._akitaorum_YW16_1______        CCTCTAAGTGATAGTTGGCAGGCGCTAACTAGTATTTAGAGGAGTTTT-A 99
```

```
YS_P._akitaorum_YW14_2______         CCTCTAAGTGATAGTTGGCAGGCGCTAACTAGTATTTAGAGGAGTTTT-A 99
YS_P._kawadai_YW12_1______           CCTCTAAGTGATAGTTGGCAGGTGCTAACTAGTATTTAGAGGAGTTTT-A 99
YS_P._takakuwai_YW13_1______         CCTCTAAGTGATAGTTGGCAGGTGCTAACTAGTATTTAGAGGAGTTTT-A 99
YS_P._albisomni_YW19_1______         CCTCTAAGTGATAGTTGGCAGGCGCTAACTAGTATTTAGAGGAGTTTT-A 99
YS_P._albisomni_YW21_1______         CCTCTAAGTGATAGTTGGCAGGCGCTAACTAGTATTTAGAGGAGTTTT-A 99
YS_P._albisomni_YW23_1______         CCTCTAAGTGATAGTTGGCAGGCGCTAACTAGTATTTAGAGGAGTTTT-A 99
YS_P._kawadai_YW03_1________         CCTCTAAGTGATAGTTGGCAGGCGCTAACTAGTATTTAGAGGAGTTTT-A 99
YS_P._takakuwai_YW54_1______         CCTCTAAGTGATAGTTGGCAGGCGCTAACTAGTATTTAGAGGAGTTTC-A 99
YS_P._takakuwai_YW73_3______         CCTCTAAGTGATAGTTGGCAGGCGCTAACTAGTATTTAGAGGAGTTTC-A 99
YS_P._acuticollis_YW07_8____         CCTCTAAGTGATAGTTGGCAGGCGCTAACTAGTATTTAGAGGAGTTTT-A 99
YS_P._albisomni_YW08_1______         CCTCTAAGTGATAGTTGGCAGGCGCTAACTAGTATTTAGAGGAGTTTT-A 99
YS_P._albisomni_YW09_1______         CCTCTAAGTGATAGTTGGCAGGCGCTAACTAGTATTTAGAGGAGTTTT-A 99
YS_P._takakuwai_YW38_1______         CCTCTAAGTGATAGTTGGCAGACGCTAACTAGTATTTAGAGGAGTTTT-A 99
YS_P._angularis_YW25_8______         CCTCTAAGTGATAGTTGGCAGGCGCTAACTAGTATTTAGAGGAGTTTTTA 100
                                     *********************  **** *******************  *

YS_P._viridicuprus_YW75_3___         CATTTCTTGCAAACACTGATGTCCGGGTAACCCCTGTTAAAATGGTTGCG 149
YS_P._viridicuprus_YW76_3___         CATTTCTTGCAAACACTGATGTCCGGGTAACCCCTGTTAAAATGGTTGCG 149
YS_P._viridicuprus_YW78_3___         CATTTCTTGCAAACACTGATGTCCGGGTAACCCCTGATAAAATGGTTGCG 149
YS_P._viridicuprus_YW58_1___         CATTTCTTGCAAACACTGATGTCCGGGTAACCCCTGATAAAATGGTTGCG 149
YS_P._viridicuprus_YW04_1___         CATTTCTTGCAAACACTGATGTCCGGGTAACCCCTGATAAAATGGTTGCG 149
YS_P._hongwonpyoi_YW34_8____         CATTTCTTGCAAACACTGATGTCCGGGTAACCCCTGATAAAATGGTTGCG 149
YS_P._hongwonpyoi_YW34_2a___         CATTTCTTGCAAACACTGATGTCCGGGTAACCCCTGATAAAATGGTTGCG 149
YS_P._hongwonpyoi_YW33_8____         CATTTCTTGCAAACACTGATGTCCGGGTAACCCCTGATAAAATGGTTGCG 149
YS_P._hongwonpyoi_YW24_1____         CATTTCTTGCAAACACTGATGTCCGGGTAACCCCTGATAAAATGGTTGCG 149
YS_P._hongwonpyoi_YW05_8____         CATTTCTTGCAAACACTGATGTCCGGGTAACCCCTGATAAAATGGTTGCG 149
YS_P._hongwonpyoi_YW35_8___          CATTTCTTGCAAACACTGATGTCCGGGTAACCCCTGATAAAATGGTTGCG 149
YS_P._hongwonpyoi_YW37_8____         CATTTCTTGCAAACACTGATGTCCGGGTAACCCCTGATAAAATGGTTGCG 149
YS_P._hongwonpyoi_YW36_1____         CATTTCTTGCAAACATTGATGTCCGGGTAACCCCTGATAAAATGGTTGCG 149
YS_P._sue_YW45_1____________         CATTTCTTGCAAACACTGATGTCCGGGTAACCCCTGATAAAATGGTTGCG 149
YS_P._sue_YW80_3____________         CATTTCTTGCAAACATTGATGTCCGGGTAACCCCTGATAAAATGGTTGCG 149
YS_P._takakuwai_YW43_2______         CATTTCTTGCAAACACTGATGTCCGGGTAACCCCTGATAAAATGGTTGCG 149
YS_P._urushiyamai_YW48_1____         CATTTCTTGCAAACACTGATGTCCGGGTAACCCCTGATAAAATGGTTGCG 149
YS_P._urushiyamai_YW50_1____         CATTTCTTGCAAACACTGATGTCCGGGTAACCCCTGATAAAATGGTTGCG 149
YS_P._urushiyamai_YW49_2____         CATTTCTTGCAAACACTGATGTCCGGGTAACCCCTGATAAAATGGTTGCG 149
YS_P._delicatulus_YW65_2____         CATTTCTTGCAAACACTGATGTCCGGGTAACCCCTGATAAAATGGTTGCG 149
```

```
YS_P._delicatulus_YW46_1____      CATTTCTTGCAAACACTGATGTCCGGGTAACCCCTGATAAAATGGTTGCG 149
YS_P._delicatulus_YW10_1____      CATTTCTTGCAAACACTGATGTCCGGGTAACCCCTGATAAAATGGTTGCG 149
YS_P._delicatulus_YW68_3____      CATTTCTTGCAAACATTGATGTCCGGGTAACCCCTGATAAAATGGTTGCG 149
YS_P._delicatulus_YW72_2____      CATTTCTTGCAAACATTGATGTCCGGGTAACCCCTGATAAAATGGTTGCG 149
YS_P._delicatulus_YW47_2____      CATTTCTTGCAAACATTGATGTCCGGGTAACCCCTGATAAAATGGTTGCG 149
YS_P._takakuwai_YW88_1______      CATTTCTTGCAAACACTGATGTCCGGGTAACCCCTGATAAAATGGTTGCG 149
YS_P._akitaorum_YW16_1______      CATTTCTTGCAAACACTGATGTCCGGGTAACCCCTGATAAAATGGTTGCG 149
YS_P._akitaorum_YW14_2______      CATTTCTTGCAAACACTGATGTCCGGGTAACCCCTGATAAAATGGTTGCG 149
YS_P._kawadai_YW12_1______        CATTTCTTGCAAACACTGATGTCCGGGTAACCCCCGATAAAATGGATGCG 149
YS_P._takakuwai_YW13_1______      CATTTCTTGCAAACACTGATGTCCGGGTAACCCCCGATAAAATGGATGCG 149
YS_P._albisomni_YW19_1______      CATTTCTTGCAAACACTGATGTCCGGGTAACCCCCGATAAAATGGTTGCG 149
YS_P._albisomni_YW21_1______      CATTTCTTGCAAACACTGATGTCCGGGTAACCCCCGATAAAATGGTTGCG 149
YS_P._albisomni_YW23_1______      CATTTCTTGCAAACACTGATGTCCGGGTAACCCCCGATAAAATGGTTGCG 149
YS_P._kawadai_YW03_1________      CATTTCTTGCAAACACTGATGTCCGGGTAACCCCCGATAAAATGGTTGCG 149
YS_P._takakuwai_YW54_1______      CATTTCTTGCAAACACTGATGTCCGGGTAACCCCCGATAAAATGGTTGCG 149
YS_P._takakuwai_YW73_3______      CATTTCTTGCAAACACTGATGTCCGGGTAACCCCCGATAAAATGGTTGCG 149
YS_P._acuticollis_YW07_8____      CATTTCTTGCAAACACTGATGTCCGGGTAACCCCCGATAAAATGGTTGCG 149
YS_P._albisomni_YW08_1______      CATTTCTTGCAAACACTGATGTCCGGGTAACCCCCGATAAAATGGTTGCG 149
YS_P._albisomni_YW09_1______      CATTTCTTGCAAACACTGATGTCCGGGTAACCCCCGATAAAATGGTTGCG 149
YS_P._takakuwai_YW38_1______      CATTTCTTGCAAACACTGATGTCCGGGTAACCCCCGATAAAATGGTTGCG 149
YS_P._angularis_YW25_8______      CATTTCTTGCAAACACTGATGTCCGGGTAACCCCTGATAAAATGGTTGCG 150
                                  *************** ****************** * ******** ****

YS_P._viridicuprus_YW75_3___      AAAAAGTTTGAAAAAAGTCTTTACAGAATTTTTTTA-A-GAATTTTTAGA 197
YS_P._viridicuprus_YW76_3___      AAAAAGTTTGAAAAAAGTCTTTACAGAATTTTTTTA-A-GAATTTTTAGA 197
YS_P._viridicuprus_YW78_3___      AAAAAGTTTGAAAAAAGTCTTTACAGAATTTTTTTA-A-GAATTTTTAGA 197
YS_P._viridicuprus_YW58_1___      AAAAAGTTTGAAAAAAGTCT--ACAGAATTTTTTTA-A-GAATTTTTAGA 195
YS_P._viridicuprus_YW04_1___      AAAAAGTTTGAAAAAAGTCTTTACAGAATTTTTTTA-A-GAATTTTTAGA 197
YS_P._hongwonpyoi_YW34_8____      AAAAAGTTTGAAAAAAGTCTTTACAGAATTTTTTTA-AAGAATTTTTAGA 198
YS_P._hongwonpyoi_YW34_2a___      AAAAAGTTTGAAAAAAGTCTTTACAGAATTTTTTTA-AAGAATTTTTAGA 198
YS_P._hongwonpyoi_YW33_8____      AAAAAGTTTGAAAAAAGTCT--ACAGAATTTTTTTT-AAGAATTTTTAGA 196
YS_P._hongwonpyoi_YW24_1____      AAAAAGTTTGAAAAAAGTCTTTACAGAATTTTTTTT-AAGAATTTTTAGA 198
YS_P._hongwonpyoi_YW05_8____      AAAAAGTTTGAAAAAAGTCTTTACAGAATTTTTTTT-AAGAATTTTTAGA 198
YS_P._hongwonpyoi_YW35_8___       AAAAAGTTTGAAAAAAGTCT--ACAGAATTTTTTTT-AAGAATTTTTAGA 196
YS_P._hongwonpyoi_YW37_8____      AAAAAGTTTGAAAAAAGTCTTTACAGAATTTTTTTT-AAGAATTTTTAGA 198
YS_P._hongwonpyoi_YW36_1____      AAAAAGTTTGAGAAAAGTCTTTACAGAATTTTTTTT-AAGAATTTTTAGA 198
```

```
YS_P._sue_YW45_1____________      AAAAAGTT-GAGAAAAGTCTTTACAGAATTCTTTTT-AAGAATTTTTAGA 197
YS_P._sue_YW80_3____________      AAAAAGTT-GAGAAAAGTCTTTACAGAATTCTTTTT-AAGAATTTTTAGA 197
YS_P._takakuwai_YW43_2______      AAAAAGTT-GAGAAAAGTCTTTACAG--------------AATTTTTAGA 184
YS_P._urushiyamai_YW48_1____      AAAAAGTT-GAGAAAAGTCTTTACAGAATTTTTTTT-AAGAATTTTTAGA 197
YS_P._urushiyamai_YW50_1____      AAAAAGTT-GAGAAAAGTCTTTACAGAATTTTTTTT-AAGAATTTTTAGA 197
YS_P._urushiyamai_YW49_2____      AAAAAGTT-GAGAAAAGTCTTTACAGAATTTTTTTT-AAGAATTTTTAGA 197
YS_P._delicatulus_YW65_2____      AAAAAGTT-GAGAAAAGTCTTTACAGAATTTTTTTTTAAGAATTTTTAAA 198
YS_P._delicatulus_YW46_1____      AAAAAGTT-GAGAAAAGTCTTTACAGGATTTTTTTTTAAGAATTTTTAAA 198
YS_P._delicatulus_YW10_1____      AAAAAGTT-GAGAAAAGTCTTTACAGAATTTTTTTTTAAGAATTTTTAAA 198
YS_P._delicatulus_YW68_3____      AAAAAGTT-GAGAAAAGTCTTTACAGAATTTTTTTTTAAGAATTTTTAAA 198
YS_P._delicatulus_YW72_2____      AAAAAGTT-GAGAAAAGTCTTTACAGAATTTTTTTTTAAGAATTTTTAAA 198
YS_P._delicatulus_YW47_2____      AAAAAGTT-GAGAAAAGTCTTTACAGAATTTTTTTTTAAGAATTTTTAAA 198
YS_P._takakuwai_YW88_1______      AAAAAGTT-GAGAAAAGTCTTTACAGAATTTTTTTT-AAGAATTTTTTGA 197
YS_P._akitaorum_YW16_1______      AAAAAGTT-GAGAAAAGTCTTTACAGAATTTTTTTT-AAGAATTTTTTGA 197
YS_P._akitaorum_YW14_2______      AAAAAGTT-GAGAAAAGTCTTTACAGAATTTTTTTT-AAGAATTTTTTGA 197
YS_P._kawadai_YW12_1______        AAAAAGTT-GAGAAAAGTCTTTACAGAATTTTTTTT-AAGAATTTTTAGA 197
YS_P._takakuwai_YW13_1______      AAAAAGTT-GAGAAAAGTCTTTACAGAATTTTTTTT-AAGAATTTTTAGA 197
YS_P._albisomni_YW19_1______      AAAAAGTT-GAGAAAAGTCTTTACAGAATTTTTTTT-AAGAATTTTTAGA 197
YS_P._albisomni_YW21_1______      AAAAAGTT-GAGAAAAGTCTTTACAGAATTTTTTTT-AAGAATTTTTAGA 197
YS_P._albisomni_YW23_1______      AAAAAGTT-GAGAAAAGTCT--ACAGAATTTTTTTT-AAGAATTTTTAGA 195
YS_P._kawadai_YW03_1________      AAAAAGTT-GAGAAAAGTCTTTACAGAATTTTTTTT-AAGAATTTTTAGA 197
YS_P._takakuwai_YW54_1______      AAAAAGTT-GAGAAAAGTCTTTACAGAATTTTTTTT-AAGAATTTTTAGA 197
YS_P._takakuwai_YW73_3______      AAAAAGTT-GAGAAAAGTCTTTACAGAATTTTTTTT-AAGAATTTTTAGA 197
YS_P._acuticollis_YW07_8____      AAAAAGTT-GAGAAAAGTCTTTACAGAATTTTTTTT-AAGAATTTTTAGA 197
YS_P._albisomni_YW08_1______      AAAAAGTT-GAGAAAAGTCTTTGCAGAATTTTTTTT-AAGAATTTTTAGA 197
YS_P._albisomni_YW09_1______      AAAAAGTT-GAGAAAAGTCTTTACAGAATTTTTTTT-AAGAATTTTTAGA 197
YS_P._takakuwai_YW38_1______      AAAAAGTT-GAGAAAAGTCTTTACAGAATTTTTTTT-AAGAATTTTTAGA 197
YS_P._angularis_YW25_8______      AAAAAGTCTGAGAAAAGTCTTTACAG-ATTTTTTTTGAAGAATTTTTAGA 199
                                  *******  ** ********   ***              *******  *

YS_P._viridicuprus_YW75_3___      TCTGGTTCTGTGTAGTTCTGTGTATATTTATTTAGAGAGAGCACTGTGGA 247
YS_P._viridicuprus_YW76_3___      TCTGGTTCTGTGTAGTTCTGTGTATATTTATTTAGAGAGAGCACTGTGGA 247
YS_P._viridicuprus_YW78_3___      TCTGGTTCTGTGTAGTTCTGTGTATATTTATTTAGAGAGAGCACTGTGGA 247
YS_P._viridicuprus_YW58_1___      TCTGGTTCTGTGTAGTTCTGTGTATATTTATTTAGAGAGAGCACTGTGGA 245
YS_P._viridicuprus_YW04_1___      TCTGGTTCTGTGTAGTTCTGTGTATATTTATTTAGAGAGAGCACTGTGGA 247
YS_P._hongwonpyoi_YW34_8____      TCTGGTTCTGTGTAGTTCTGTGTAT--TTATTTAGAGAGAGCACTGTGGA 246
```

```
YS_P._hongwonpyoi_YW34_2a___         TCTGGTTCTGTGTAGTTCTGTGTATATTTATTTAGAGAGAGCACTGTGGA 248
YS_P._hongwonpyoi_YW33_8____         TCTGGTTCTGTGTAGTTCTGTGTATATTTATTTAGAGAGAGCACTGTGGA 246
YS_P._hongwonpyoi_YW24_1____         TCTGGTTCTGTGTAGTTCTGTGTATATTTATTTAGAGAGAGCACTGTGGA 248
YS_P._hongwonpyoi_YW05_8____         TCTGGTTCTGTGTAGTTCTGTGTATATTTATTTAGAGAGAGCACTGTGGA 248
YS_P._hongwonpyoi_YW35_8___          TCTGGTTCTGTGTAGTTCTGTGTATATTTATTTAGAGAGAGCACTGTGGA 246
YS_P._hongwonpyoi_YW37_8____         TCTGGTTCTGTGTAGTTCTGTGTATATTTATGTAGAGAGAGCACTGTGGA 248
YS_P._hongwonpyoi_YW36_1____         TCTGGTTCTGTGTAGTTCTGTGTATATTTATTTAGAGAGAGCACTGTGGA 248
YS_P._sue_YW45_1____________         TCTGGTTCTGTGTAGTTCTGTGTATATTTATTTAGAGAGAGCACTGTGGA 247
YS_P._sue_YW80_3____________         TCTGGTTCTGTGTAGTTCTGTGTATATTTATTTAGAGAGAGCACTGTGGA 247
YS_P._takakuwai_YW43_2______         TCTGGTTCTGTGTAGTTCTGTGTATATTTATTTAGAGAGAGCACTGTGGA 234
YS_P._urushiyamai_YW48_1____         TCTGGTTCTGTGTAGTTCTGTGTATATTTATTTAGAGAGAGCACTGTGGA 247
YS_P._urushiyamai_YW50_1____         TCTGGTTCTGTGTAGTTCTGTGTATATTTATTTAGAGAGAGCACTGTGGA 247
YS_P._urushiyamai_YW49_2____         TCTGGTTCTGTGTAGTTCTGTGTATATTTATTTAGAGAGAGCACTGTGGA 247
YS_P._delicatulus_YW65_2____         TCTGGTTCTGTGTAGTTCAGTGTATATTTATATAGAGAGAGCACTGTGGA 248
YS_P._delicatulus_YW46_1____         TCTGGTTCTGTGTAGTTCAGTGTATATTTATATAGAGAGAGCACTGTGGA 248
YS_P._delicatulus_YW10_1____         TCTGGTTCTGTGTAGTTCAGTGTATATTTATATAGAGAGAGCACTGTGGA 248
YS_P._delicatulus_YW68_3____         TCTGGTTCTGTGTAGTTCAGTGTATATTTATATAGAGAGAGCACTGTGGA 248
YS_P._delicatulus_YW72_2____         TCTGGTTCTGTGTAGTTCAGTGTATATTTATATAGAGAGAGCACTGTGGA 248
YS_P._delicatulus_YW47_2____         TCTGGTTCTGTGTAGTTCAGTGTATATTTATATAGAGAGAGCACTGTGGA 248
YS_P._takakuwai_YW88_1______         TCTGGTTCTGTGTAGTTCTGTGTATATTTATTTAGAGAGAGCACTGTGGA 247
YS_P._akitaorum_YW16_1______         TCTGGTTCTGTGTAGTTCTGTGTATATTTATTTAGAGAGAGCACTGTGGA 247
YS_P._akitaorum_YW14_2______         TCTGGTTCTGTGTAGTTCTGTGTATATTTATTTAGAGAGAGCACTGTGGA 247
YS_P._kawadai_YW12_1______           TCTGGTTCTGTGTAGTTCTGTGTATATTTATTTAGAGAGAGCACTGTGGA 247
YS_P._takakuwai_YW13_1______         TCTGGTTCTGTGTAGTTCTGTGTATATTTATTTAGAGAGAGCACTGTGGA 247
YS_P._albisomni_YW19_1______         TCTGGTTCTGTGTAGTTCTGTGTATATTTATTTAGAGAGAGCACTGTGGA 247
YS_P._albisomni_YW21_1______         TCTGGTTCTGTGTAGTTCTGTGTATATTTATTTAGAGAGAGCACTGTGGA 247
YS_P._albisomni_YW23_1______         TCTGGTTCTGTGTAGTTCTGTGTATATTTATTTAGAGAGAGCACTGTGGA 245
YS_P._kawadai_YW03_1________         TCTGGTTCTGTGTAGTTCTGTGTATATTTATTTAGAGAGAGCACTGTGGA 247
YS_P._takakuwai_YW54_1______         TCTGGTTCTGTGTAGTTCTGTGTATATTTATTTAGAGAGAGCACTGTGGA 247
YS_P._takakuwai_YW73_3______         TCTGGTTCTGTGTAGTTCTGTGTATATTTATTTAGAGAGAGCACTGTGGA 247
YS_P._acuticollis_YW07_8____         TCTGGCTCTGTGTAGTTCTGTGTATATTTATTTAGAGAGAGCACTGTGGA 247
YS_P._albisomni_YW08_1______         TCTGGTTCTGTGTAGTTCTGTGTATATTTATTTAGAGAGAGCACTGTGGA 247
YS_P._albisomni_YW09_1______         TCTGGTTCTGTGTAGTTCTGTGTATATTTATTTAGAGAGAGCACTGTGGA 247
YS_P._takakuwai_YW38_1______         TCTGGTTCTGTGTAGTTCTGTGTATATTTATTTAGAGAGAGCACTGTGGA 247
YS_P._angularis_YW25_8______         TCTGGTTCTGTGTAGTTCTGTGTATATATATTTAGAGAGAGCAGTGTGGT 249
                                     ***** ************ ******   *** *********** *****
```

```
YS_P._viridicuprus_YW75_3___        TCTGAGTTAGATAGAGACAAATTTTAGGAACAAGAGTCGAGTCGAAATCG 297
YS_P._viridicuprus_YW76_3___        TCTGAGTTAGATAGAGACAAATTTTAGGAACAAGAGTCGAGTCGAAATCG 297
YS_P._viridicuprus_YW78_3___        TCTGAGTTAGATAGAGACAAATTTTAGGAACAAGAGTCGAGTCGAAATCG 297
YS_P._viridicuprus_YW58_1___        TCTGAGTTAGATAGAGACAAATTTTAGGAACAAGAGTCGAGTCGAAATCG 295
YS_P._viridicuprus_YW04_1___        TCTGAGTTAGATAGAGACAAATTTTAGGAACAAGAGTCGAGTCGAAATCG 297
YS_P._hongwonpyoi_YW34_8____        TCTGAGTTAGATAGAGACAAATTTTAGGAACAAGAGTCGAGTCGAAATCG 296
YS_P._hongwonpyoi_YW34_2a___        TCTGAGTTAGATAGAGACAAATTTTAGGAACAAGAGTCGAGTCGAAATCG 298
YS_P._hongwonpyoi_YW33_8____        TCTGAGTTAGATAGAGACAAATTTTAGGAACAAGAGTCGAGTCGAAATCG 296
YS_P._hongwonpyoi_YW24_1____        TCTGAGTTAGATAGAGACAAATTTTAGGAACAAGAGTCGAGTCGAAATCG 298
YS_P._hongwonpyoi_YW05_8____        TCTGAGTTAGATAGAGACAAATTTTAGGAACAAGAGTCGAGTCGAAATCG 298
YS_P._hongwonpyoi_YW35_8___         TCTGAGTTAGATAGAGACAAATTTTAGGAACAAGAGTCGAGTCGAAATCG 296
YS_P._hongwonpyoi_YW37_8____        TCTGAGTTAGATAGAGACAAATTTTAGGAACAAGAGTCGAGTCGAAATCG 298
YS_P._hongwonpyoi_YW36_1____        TCTGAGTTAGATAGAGACAAATTTTAGGAACAAGAGTCGAGTCGAAATCG 298
YS_P._sue_YW45_1____________        TCTGAGTTAGATAGAGACAAATTTTAGGAACAAGAGTCGAGTCGAAGTCG 297
YS_P._sue_YW80_3____________        TCTGAGTTAGATAGAGACAAATTTTAGGAACAAGAGTCGAGTCGAAGTCG 297
YS_P._takakuwai_YW43_2______        TCTGAGTTAGATAGAGACAAATTTTAGGAACAAGAGTCGAGTCGAAATCG 284
YS_P._urushiyamai_YW48_1____        TCTGAGTTAGATAGAGACAAATTTTAGGAACAAGAGTCGAGTCAAAATCG 297
YS_P._urushiyamai_YW50_1____        TCTGAGTTAGATAGAGACAAATTTTAGGAACAAGAGTCGAGTCAAAATCG 297
YS_P._urushiyamai_YW49_2____        TCTGAGTTAGATAGAGACAAATTTTAGGAACAAGAGTCGAGTCAAAATCG 297
YS_P._delicatulus_YW65_2____        TCTGAGTTAGATAGAGACAAATTTTAGGAACAAGAGTCGAGTCGAAATCG 298
YS_P._delicatulus_YW46_1____        TCTGAGTTAGATAGAGACAAATTTTAGGAACAAGAGTCGAGTCGAAATCG 298
YS_P._delicatulus_YW10_1____        TCTGAGTTAGATAGAGACAAATTTTAGGAACAAGAGTCGAGTCGAAATCG 298
YS_P._delicatulus_YW68_3____        TCTGAGTTAGATAGAGACAAATTTTAGGAACAAGAGTCGAGTCGAAATCG 298
YS_P._delicatulus_YW72_2____        TCTGAGTTAGATAGAGACAAATTTTAGGAACAAGAGTCGAGTCGAAATCG 298
YS_P._delicatulus_YW47_2____        TCTGAGTTAGATAGAGACAAATTTTAGGAACAAGAGTCGAGTCGAAATCG 298
YS_P._takakuwai_YW88_1______        TCTGAGTTAGATAGAGACAAATTTTAGGAACAAGAGTCGAGTCGAAATCG 297
YS_P._akitaorum_YW16_1______        TCTGAGTTAGATAGAGACAAATTTTAGGAACAAGAGTCGAGTCGAAATCG 297
YS_P._akitaorum_YW14_2______        TCTGAGTTAGATAGAGACAAATTTTAGGAACAAGAGTCGAGTCGAAATCG 297
YS_P._kawadai_YW12_1______          TCTGAGTTAGATAGAGACAAATTTTAGGAACAAGAGTCGAGTCGAAATCG 297
YS_P._takakuwai_YW13_1______        TCTGAGTTAGATAGAGACAAATTTTAGGAACAAGAGTCGAGTCGAAATCG 297
YS_P._albisomni_YW19_1______        TCTGAGTTAGATAGAGACAAATTTTAGGAACAAGAGTCGAGTCGAAATCG 297
YS_P._albisomni_YW21_1______        TCTGAGTTAGATAGAGACAAATTTTAGGAACAAGAGTCGAGTCGAAATCG 297
YS_P._albisomni_YW23_1______        TCTGAGTTAGATAGAGACAAATTTTAGGAACAAGAGTCGAGTCGAAATCG 295
YS_P._kawadai_YW03_1________        TCTGAGTTAGATAGAGACAAATTTTAGGAACAAGAGTCGAGTCGAAATCG 297
YS_P._takakuwai_YW54_1______        TCTGAGTTAGAAAGAGACAAATTTTAGGAACAAGAGTCGAGTCGAAATCG 297
```

```
YS_P._takakuwai_YW73_3______      TCTGAGTTAGATAGAGACAAATTTTAGGAACAAGAGTCGAGTCGAAATCG 297
YS_P._acuticollis_YW07_8____      TCTGAGTTAGATAGAGACAAATTTTAGGAACAAGAGTCGAGTCGAAATCG 297
YS_P._albisomni_YW08_1______      TCTGAGTTAGATAGAGACAAATTTTAGGAACAAGAGTCGAGTCGAAATCG 297
YS_P._albisomni_YW09_1______      TCTGAGTTAGATAGAGACAAATTTTAGGAACAAGAGTCGAGTCGAAATCG 297
YS_P._takakuwai_YW38_1______      TCTGAGTTAGATAGAGACAAATTTTAGGAACAAGAGTCGAGTCGAAATCG 297
YS_P._angularis_YW25_8______      TCTGAGTTAGATAGAGACGAATTTTAGGAACGAGAGTCGA-----AATCG 294
                                  *********** ****** ************ ********     * ***

YS_P._viridicuprus_YW75_3___      GTCACGTGTTAAAACGATAGACATGGAAAGGAAGCATAACGGTAGAGTAA 347
YS_P._viridicuprus_YW76_3___      GTCACGTGTTAAAACGATAGACATGGAAAGGAAGCATAACGGTAGAGTAA 347
YS_P._viridicuprus_YW78_3___      GTCACGTGTTAAAACGATAGACATGGAAAGGAAGCATAACGGTAGAGTAA 347
YS_P._viridicuprus_YW58_1___      GTCACGTGTTAAAACGATAGACATGGAAAGGAAGCATAACGGTAGAGTAA 345
YS_P._viridicuprus_YW04_1___      GTCACGTGTTAAAACGATAGACATGGAAAGGAAGCATAACGGTAGAGTAA 347
YS_P._hongwonpyoi_YW34_8____      GTCACGTGTTAAAACGATAGACATGGAAAGGAAGCATAACGGTAGAGTAA 346
YS_P._hongwonpyoi_YW34_2a___      GTCACGTGTTAAAACGATAGACATGGAAAGGAAGCATAACGGTAGAGTAA 348
YS_P._hongwonpyoi_YW33_8____      GTCACGTGTTAAAACGATAGACATGGAAAGGAAGCATAACGGTAGAGTAA 346
YS_P._hongwonpyoi_YW24_1____      GTCACGTGTTAAAACGATAGACATGGAAAGGAAGCATAACGGTAGAGTAA 348
YS_P._hongwonpyoi_YW05_8____      GTCACGTGTTAAAACGATAGACATGGAAAGGAAGCATAACGGTAGAGTAA 348
YS_P._hongwonpyoi_YW35_8___       GTCACGTGTTAAAACGATAGACATGGAAAGGAAGCATAACGGTAGAGTAA 346
YS_P._hongwonpyoi_YW37_8____      GTCACGTGTTAAAACGATAGACATGGAAAGGAAGCATAACGGTAGAGTAA 348
YS_P._hongwonpyoi_YW36_1____      GTCACGTGTTAAAACGATAGACATGGAAAGGAAGCATAACGGTAGAATAA 348
YS_P._sue_YW45_1____________      GTCACGTGTTGAAATGATAGACATGGAAAGGAAGCATAACGGTAGAGTAA 347
YS_P._sue_YW80_3____________      GTCACGTGTTGAAATGATAGACATGGAAAGGAAGCATAACGGTAGAGTAA 347
YS_P._takakuwai_YW43_2______      GTCACGTGTTGAAATGATAGACATGGAAAGGAAGCATAACGGTAGAGTAA 334
YS_P._urushiyamai_YW48_1____      GTCACGTGTTGAAACGATAGACATGGAAAGGAAGCATAACGGTAGAGTAA 347
YS_P._urushiyamai_YW50_1____      GTCACGTGTTGAAACGATAGACATGGAAAGGAAGCATAACGGTAGAGTAA 347
YS_P._urushiyamai_YW49_2____      GTCACGTGTTGAAACGATAGACATGGAAAGGAAGCATAACGGTAGAGTAA 347
YS_P._delicatulus_YW65_2____      GTCACGTGTTGAAACGATAGACATGGAAAGGAAGCATAACGGTAGAGTAA 348
YS_P._delicatulus_YW46_1____      GTCACGTGTTGAAACGATAGACATGGAAAGGAAGCATAACGGTAGAGTAA 348
YS_P._delicatulus_YW10_1____      GTCACGTGTTGAAACGATAGACATGGAAAGGAAGCATAACGGTAGAGTAA 348
YS_P._delicatulus_YW68_3____      GTCACGTGTTGAAACGATAGACATGGAAAGGAAGCATAACGGTAGAGTAA 348
YS_P._delicatulus_YW72_2____      GTCACGTGTTGAAACGATAGACATGGAAAGGAAGCATAACGGTAGAGTAA 348
YS_P._delicatulus_YW47_2____      GTCACGTGTTGAAACGATAGACATGGAAAGGAAGCATAACGGTAGAGTAA 348
YS_P._takakuwai_YW88_1______      GTCACGTGTTGAAACGATAGACATGGAAAGGAAGCATAACGGTAGAGTAA 347
YS_P._akitaorum_YW16_1______      GTCACGTGTTGAAACGATAGACATGGAAAGGAAGCATAACGGTAGAGTAA 347
YS_P._akitaorum_YW14_2______      GTCACGTGTTGAAACGATAGACATGGAAAGGAAGCATAACGGTAGAGTAA 347
```

```
YS_P._kawadai_YW12_1______          GTCACGTGTTGAAACGATAGACATGGAAAGGAAGCATAACGGTAGAGTAA 347
YS_P._takakuwai_YW13_1______        GTCACGTGTTGAAACGATAGACATGGAAAGGAAGCATAACGGTAGAGTAA 347
YS_P._albisomni_YW19_1______        GTCACGTGTTGAAACGATAGACATGGAAAGGAAGGATAACGGTAGAGTAA 347
YS_P._albisomni_YW21_1______        GTCACGTGTTGAAACGATAGACATGGAAAGGAAGGATAACGGTAGAGTAA 347
YS_P._albisomni_YW23_1______        GTCACGTGTTGAAACGATAGACATGGAAAGGAAGGATAACGGTAGAGTAA 345
YS_P._kawadai_YW03_1________        GTCACGTGTTGAAACGATAGACATGGAAAGGAAGGATAACGGTAGAGTAA 347
YS_P._takakuwai_YW54_1______        GTCACGTGTTGAAACGATAGACATGGAAAGGAAGGATAACGGTAGAGTAA 347
YS_P._takakuwai_YW73_3______        GTCACGTGTTGAAACGATAGACATGGAAAGGAAGGATAACGGTAGAGTAA 347
YS_P._acuticollis_YW07_8____        GTCACGTGTTGAAACGATAGACATGGAAAGGAAGGATAACGGTAGAGTAA 347
YS_P._albisomni_YW08_1______        GTCACGTGTTGAAACGATAGACATGGAAAGGAAGGATAACGGTAGAGTAA 347
YS_P._albisomni_YW09_1______        GTCACGTGTTGAAACGATAGACATGGAAAGGAAGGATAACGGTAGAGTAA 347
YS_P._takakuwai_YW38_1______        GTCACGTGTTGAAACGATAGACATGGAAAGGAAGCATAACGGTAGAGTAA 347
YS_P._angularis_YW25_8______        GTCACGTGTTGAAACGATAGACATTGACAGGAAGAATAACGGCAGAGTAA 344
                                    ********** *** ********* ** ****** ******* *** ***

YS_P._viridicuprus_YW75_3___        GAAAAGTGTTAAGAATTTTGAAAAATATTGTTTTGTAG------------ 385
YS_P._viridicuprus_YW76_3___        GAAAAGTGTTAAGAATTTTGAAAAATATTGTTTTGTAG------------ 385
YS_P._viridicuprus_YW78_3___        GAAAAGTGTTAAGAATTTTGAAAAATATTGTTTTGTAG------------ 385
YS_P._viridicuprus_YW58_1___        GAAAAGTGTTAAGAATTTTGAAAAATATTGTTTTGTAG------------ 383
YS_P._viridicuprus_YW04_1___        GAAAAGTGTTAAGAATTTTGAAAAATATTGTTTTGTAG------------ 385
YS_P._hongwonpyoi_YW34_8____        GAAAAGTGTTAAGAATTTTGAAAAATATTGTTTTGTAG------------ 384
YS_P._hongwonpyoi_YW34_2a___        GAAAAGTGTTAAGAATTTTGAAAAATATTGTTTTGTAG------------ 386
YS_P._hongwonpyoi_YW33_8____        GAAAAGTGTTAAGAATTTTGAAAAATATTGTTTTGTAG------------ 384
YS_P._hongwonpyoi_YW24_1____        GAAAAGTGTTAAGAATTTTGAAAAATATTGTTTTGTAG------------ 386
YS_P._hongwonpyoi_YW05_8____        GAAAAGTGTTAAGAATTTTGAAAAATATTGTTTTGTAG------------ 386
YS_P._hongwonpyoi_YW35_8___         GAAAAGTGTTAAGAATTTTGAAAAATATTGTTTTGTAG------------ 384
YS_P._hongwonpyoi_YW37_8____        GAAAAGTGTTAAGAATTTTGAAAAATATTGTTTTGTAG------------ 386
YS_P._hongwonpyoi_YW36_1____        GAAAAGTGTTAAGAATTTTGAAAAATATTGTTTTGTAG------------ 386
YS_P._sue_YW45_1____________        GAAAAGTGTTAAGAATTTTGAAAAA-ATTGTGTTGTAG------------ 384
YS_P._sue_YW80_3____________        GAAAAGTGTTAAGAATTTTGAAAAA-ATTGTGTTGTAG------------ 384
YS_P._takakuwai_YW43_2______        GAAAAGTGTTAAGAATTTTGAAAAA-ATTGTGTTGTAG------------ 371
YS_P._urushiyamai_YW48_1____        GAAAAGTGTTAAGAATTTTGAAAAA-ATTGTTTTGTAG------------ 384
YS_P._urushiyamai_YW50_1____        GAAAAGTGTTAAGAATTTTGAAAAA-ATTGTTTTGTAG------------ 384
YS_P._urushiyamai_YW49_2____        GAAAAGTGTTAAGAATTTTGAAAAA-ATTGTTTTGTAG------------ 384
YS_P._delicatulus_YW65_2____        GAAAAGTGTTAAGAATTTTGAAAAA-ATTGTTTTGTAG------------ 385
YS_P._delicatulus_YW46_1____        GAAAAGTGTTAAGAATTTTGAAAAA-ATTGTTTTGTAG------------ 385
```

```
YS_P._delicatulus_YW10_1____      GAAAAGTGTTAAGAATTTTGAAAAA-ATTGTTTTGTAG------------ 385
YS_P._delicatulus_YW68_3____      GAAAAGTGTTAAGAATTTTGAATAA-ATTGTTTTGTAG------------ 385
YS_P._delicatulus_YW72_2____      GAAAAGTGTTAAGAATTTTGAATAA-ATTGTTTTGTAG------------ 385
YS_P._delicatulus_YW47_2____      GAAAAGTGTTAAGAATTTTGAATAA-ATTGTTTTGTAG------------ 385
YS_P._takakuwai_YW88_1______      GAAAAGTGTTAAGAATTTTGAAAAA-ATTGTTTTGTAG------------ 384
YS_P._akitaorum_YW16_1______      GAAAAGTGTTAAGAATTTTGAAAAA-ATTGTTTTGTAG------------ 384
YS_P._akitaorum_YW14_2______      GAAAAGTGTTAAGAATTTTGAAAAA-ATTGTTTTGTAG------------ 384
YS_P._kawadai_YW12_1______        GAAAAGTGTTAAGAATTTTGAAAAA-ATTGTTTTGTAG------------ 384
YS_P._takakuwai_YW13_1______      GAAAAGTGTTAAGAATTTTGAAAAA-ATTGTTTTGTAGAGACAAATTTTA 396
YS_P._albisomni_YW19_1______      GAAAAGTGTTAAGAATTTTGAAAAA-ATTGTTTTGTAG------------ 384
YS_P._albisomni_YW21_1______      GAAAAGTGTTAAGAATTTTGAAAAA-ATTGTTTTGTAG------------ 384
YS_P._albisomni_YW23_1______      GAAAAGTGTTAAGAATTTTGAAAAA-ATTGTTTTGTAG------------ 382
YS_P._kawadai_YW03_1________      GAAAAGTGTTAAGAATTTTGAAAAA-ATTGTTTTGTAG------------ 384
YS_P._takakuwai_YW54_1______      GAAAAGTGTTAAGAATTTTGAAACA-ATTGTTTTGTAG------------ 384
YS_P._takakuwai_YW73_3______      GAAAAGTGTTAAGAATTTTGAAAAA-ATTGTTTTGTAG------------ 384
YS_P._acuticollis_YW07_8____      GAAAAGTGTTAAGAATTTTGAAAAA-ATTGTTTTGTAG------------ 384
YS_P._albisomni_YW08_1______      GAAAAGTGTTAAGAATTTTGAAAAA-ATTGTTTTGTAG------------ 384
YS_P._albisomni_YW09_1______      GAAAAGTGTTAAGAATTTTGAAAAA-ATTGTTTTGTAG------------ 384
YS_P._takakuwai_YW38_1______      GAAAAGTGTTAAGAATTTTGAAAAA-ATTGTTTTGTAG------------ 384
YS_P._angularis_YW25_8______      GAAAAGTGTTAAGAATTTTGAAAAATAATGTTTTGTAG------------ 382
                                  **********************  * * *** ******

YS_P._viridicuprus_YW75_3___      --------------------------------------------------
YS_P._viridicuprus_YW76_3___      --------------------------------------------------
YS_P._viridicuprus_YW78_3___      --------------------------------------------------
YS_P._viridicuprus_YW58_1___      --------------------------------------------------
YS_P._viridicuprus_YW04_1___      --------------------------------------------------
YS_P._hongwonpyoi_YW34_8____      --------------------------------------------------
YS_P._hongwonpyoi_YW34_2a___      --------------------------------------------------
YS_P._hongwonpyoi_YW33_8____      --------------------------------------------------
YS_P._hongwonpyoi_YW24_1____      --------------------------------------------------
YS_P._hongwonpyoi_YW05_8____      --------------------------------------------------
YS_P._hongwonpyoi_YW35_8___       --------------------------------------------------
YS_P._hongwonpyoi_YW37_8____      --------------------------------------------------
YS_P._hongwonpyoi_YW36_1____      --------------------------------------------------
YS_P._sue_YW45_1____________      --------------------------------------------------
```

```
YS_P._sue_YW80_3____________          --------------------------------------------------
YS_P._takakuwai_YW43_2______          --------------------------------------------------
YS_P._urushiyamai_YW48_1____          --------------------------------------------------
YS_P._urushiyamai_YW50_1____          --------------------------------------------------
YS_P._urushiyamai_YW49_2____          --------------------------------------------------
YS_P._delicatulus_YW65_2____          --------------------------------------------------
YS_P._delicatulus_YW46_1____          --------------------------------------------------
YS_P._delicatulus_YW10_1____          --------------------------------------------------
YS_P._delicatulus_YW68_3____          --------------------------------------------------
YS_P._delicatulus_YW72_2____          --------------------------------------------------
YS_P._delicatulus_YW47_2____          --------------------------------------------------
YS_P._takakuwai_YW88_1______          --------------------------------------------------
YS_P._akitaorum_YW16_1______          --------------------------------------------------
YS_P._akitaorum_YW14_2______          --------------------------------------------------
YS_P._kawadai_YW12_1______            --------------------------------------------------
YS_P._takakuwai_YW13_1______          GGAACAAGAGTCGAGTCGAAATCGGTCACGTGTTGAAACGATAGACATGG 446
YS_P._albisomni_YW19_1______          --------------------------------------------------
YS_P._albisomni_YW21_1______          --------------------------------------------------
YS_P._albisomni_YW23_1______          --------------------------------------------------
YS_P._kawadai_YW03_1________          --------------------------------------------------
YS_P._takakuwai_YW54_1______          --------------------------------------------------
YS_P._takakuwai_YW73_3______          --------------------------------------------------
YS_P._acuticollis_YW07_8____          --------------------------------------------------
YS_P._albisomni_YW08_1______          --------------------------------------------------
YS_P._albisomni_YW09_1______          --------------------------------------------------
YS_P._takakuwai_YW38_1______          --------------------------------------------------
YS_P._angularis_YW25_8______          --------------------------------------------------


YS_P._viridicuprus_YW75_3___          --------------------------------------------------
YS_P._viridicuprus_YW76_3___          --------------------------------------------------
YS_P._viridicuprus_YW78_3___          --------------------------------------------------
YS_P._viridicuprus_YW58_1___          --------------------------------------------------
YS_P._viridicuprus_YW04_1___          --------------------------------------------------
YS_P._hongwonpyoi_YW34_8____          --------------------------------------------------
YS_P._hongwonpyoi_YW34_2a___          --------------------------------------------------
```

```
YS_P._hongwonpyoi_YW33_8____      --------------------------------------------------
YS_P._hongwonpyoi_YW24_1____      --------------------------------------------------
YS_P._hongwonpyoi_YW05_8____      --------------------------------------------------
YS_P._hongwonpyoi_YW35_8___       --------------------------------------------------
YS_P._hongwonpyoi_YW37_8____      --------------------------------------------------
YS_P._hongwonpyoi_YW36_1____      --------------------------------------------------
YS_P._sue_YW45_1____________      --------------------------------------------------
YS_P._sue_YW80_3____________      --------------------------------------------------
YS_P._takakuwai_YW43_2______      --------------------------------------------------
YS_P._urushiyamai_YW48_1____      --------------------------------------------------
YS_P._urushiyamai_YW50_1____      --------------------------------------------------
YS_P._urushiyamai_YW49_2____      --------------------------------------------------
YS_P._delicatulus_YW65_2____      --------------------------------------------------
YS_P._delicatulus_YW46_1____      --------------------------------------------------
YS_P._delicatulus_YW10_1____      --------------------------------------------------
YS_P._delicatulus_YW68_3____      --------------------------------------------------
YS_P._delicatulus_YW72_2____      --------------------------------------------------
YS_P._delicatulus_YW47_2____      --------------------------------------------------
YS_P._takakuwai_YW88_1______      --------------------------------------------------
YS_P._akitaorum_YW16_1______      --------------------------------------------------
YS_P._akitaorum_YW14_2______      --------------------------------------------------
YS_P._kawadai_YW12_1______        --------------------------------------------------
YS_P._takakuwai_YW13_1______      AAAGGAAGCATAACGGTAGAGTAAGAAAAGTGTTAAGAATTTTGAAAAAA 496
YS_P._albisomni_YW19_1______      --------------------------------------------------
YS_P._albisomni_YW21_1______      --------------------------------------------------
YS_P._albisomni_YW23_1______      --------------------------------------------------
YS_P._kawadai_YW03_1________      --------------------------------------------------
YS_P._takakuwai_YW54_1______      --------------------------------------------------
YS_P._takakuwai_YW73_3______      --------------------------------------------------
YS_P._acuticollis_YW07_8____      --------------------------------------------------
YS_P._albisomni_YW08_1______      --------------------------------------------------
YS_P._albisomni_YW09_1______      --------------------------------------------------
YS_P._takakuwai_YW38_1______      --------------------------------------------------
YS_P._angularis_YW25_8______      --------------------------------------------------
```

YS_P._viridicuprus_YW75_3___ -----------GTAGTAGAAGAAGAGTGTTAGGAGTATTAAATGGTTGAG 424

YS_P._viridicuprus_YW76_3___ -----------GTAGTAGAAGAAGAGTGTTAGGAGTATTAAATGGTTGAG 424

YS_P._viridicuprus_YW78_3___ -----------GTAGTAGAAGAAGAGTGTTAGGAGTATTAAATGGTTGAG 424

YS_P._viridicuprus_YW58_1___ -----------GTAGTAGAAGAAGAGTGTTAGGAGTATTAAATGGTTGAG 422

YS_P._viridicuprus_YW04_1___ -----------GTAGTAGAAGAAGAGTGTTAGGAGTATTAAATGGTTGAG 424

YS_P._hongwonpyoi_YW34_8____ -----------GTAGTAGAAGAAGAGTGTTAGGAGTATTAAATGGTTGAG 423

YS_P._hongwonpyoi_YW34_2a___ -----------GTAGTAGAAGAAGAGTGTTAGGAGTATTAAATGGTTGAG 425

YS_P._hongwonpyoi_YW33_8____ -----------GTAGTAGAAGAAGAGTGTTAGGAGTATTAAATGGTTGAG 423

YS_P._hongwonpyoi_YW24_1____ -----------GTAGTAGAAGAAGAGTGTTAGGAGTATTAAATGGTTGAG 425

YS_P._hongwonpyoi_YW05_8____ -----------GTAGTAGAAGAAGAGTGTTAGGAGTATTAAATGGTTGAG 425

YS_P._hongwonpyoi_YW35_8___ -----------GTAGTAGAAGAAGAGTGTTAGGAGTATTAAATGGTTGAG 423

YS_P._hongwonpyoi_YW37_8____ -----------GTAGTAGAAGAAGAGTGTTAGGAGTATTAAATGGTTGAG 425

YS_P._hongwonpyoi_YW36_1____ -----------GTAGTAGAAGAAGAGTGTTAGGAGTATTAAATGGTTGAG 425

YS_P._sue_YW45_1____________ -----------GTAATAGAAGAAACTCGTTAGGAGTATTAAATGGTTCAG 423

YS_P._sue_YW80_3____________ -----------GTAATAGAAGAAACTCGTTAGGAGTATTAAATGGTTCAG 423

YS_P._takakuwai_YW43_2______ -----------GTAATAGAAGAAACTCGTTAGGAGTATTAAATGGTTCAG 410

YS_P._urushiyamai_YW48_1____ -----------GTAATAGAAGAAACTCGTTAGGAGTATTAAATGGTTGAG 423

YS_P._urushiyamai_YW50_1____ -----------GTAATAGAAGAAACTCGTTAGGAGTATTAAATGGTTGAG 423

YS_P._urushiyamai_YW49_2____ -----------GTAATAGAAGAAACTCGTTAGGAGTATTAAATGGTTGAG 423

YS_P._delicatulus_YW65_2____ -----------GTAGTAGAAGAAACTCGTTAGGAGTATTAAATGGTTGAG 424

YS_P._delicatulus_YW46_1____ -----------GTAGTAGAAGAAACTCGTTAGGAGTATTAAATGGTTGAG 424

YS_P._delicatulus_YW10_1____ -----------GTAGTAGAAGAAACTCGTTAGGAGTATTAAATGGTTGAG 424

YS_P._delicatulus_YW68_3____ -----------GTAGTAGAAGAAACTCGTTAGGAGTATTAAATGGTTGAG 424

YS_P._delicatulus_YW72_2____ -----------GTAGTAGAAGAAACTCGTTAGGAGTATTAAATGGTTGAG 424

YS_P._delicatulus_YW47_2____ -----------GTAGTAGAAGAAACTCGTTAGGAGTATTAAATGGTTGAG 424

YS_P._takakuwai_YW88_1______ -----------GTAGTAGAAGAAACTCGTTAGGAGTATTAAATGGTTGAG 423

YS_P._akitaorum_YW16_1______ -----------GTAGTAGAAGAAACTCGTTAGGAGTATTAAATGGTTGAG 423

YS_P._akitaorum_YW14_2______ -----------GTAGTAGAAGAAACTCGTTAGGAGTATTAAATGGTTGAG 423

YS_P._kawadai_YW12_1______ -----------GTAGTAGAAGAAACTCGCTATGAGTATTAAATGGTTGAG 423

YS_P._takakuwai_YW13_1______ TTGTTTTGTAGGTAGTAGAAGAAACTCGCTATGAGTATTAAATGGTTGAG 546

YS_P._albisomni_YW19_1______ -----------GTAGTAGAAGAAACTCGTTAGGAGTATTAAATGGTTGAG 423

YS_P._albisomni_YW21_1______ -----------GTAGTAGAAGAAACTCGTTAGGAGTATTAAATGGTTGAG 423

YS_P._albisomni_YW23_1______ -----------GTAGTAGAAGAAACTCGTTAGGAGTATTAAATGGTTGAG 421

YS_P._kawadai_YW03_1________ -----------GTAGTAGAAGAAACTCGTTAGGAGTATTAAATGGTTGAG 423

YS_P._takakuwai_YW54_1______ -----------GTAGTAGAAGAAACTCGTTAGGAGTATTAAATGGTTGAG 423

YS_P._takakuwai_YW73_3______ -----------GTAGTAGAAGAAACTCGTTAGGAGTATTAAATGGTTGAG 423

```
YS_P._acuticollis_YW07_8____         -----------GTAGTAGAAGAAACTCGTTAGGAGTATTAAATGGTTGAG 423
YS_P._albisomni_YW08_1______         -----------GTAGTAGAAGAAACTCGTTAGGAGTATTAAATGGTTGAG 423
YS_P._albisomni_YW09_1______         -----------GTAGTAGAAGAAACTCGTTAGGAGTATTAAATGGTTGAA 423
YS_P._takakuwai_YW38_1______         -----------GTAGTAGAAGAAACTCGTTAGGAGTATTAAATGGTTGAG 423
YS_P._angularis_YW25_8______         -----------GTAGTAGAAGAAGAGTGTTAGGAGTATAAAATGGTAGAA 421
                                                *** ********    * ** ****** *******  *

YS_P._viridicuprus_YW75_3___         GAAAAATCGAAGCGGGAGCGATGGGGTCACGTGACTAGCGCATAGTTAAG 474
YS_P._viridicuprus_YW76_3___         GAAAAATCGAAGCGGGAGCGATGGGGTCACGTGACTAGCGCATAGTTAAG 474
YS_P._viridicuprus_YW78_3___         GAAAAATCGAAGCGGGAGCGATGGGGTCACGTGACTAGCGCATAGTTAAG 474
YS_P._viridicuprus_YW58_1___         GAAAAATCGAACCGGGAGCGATGGGGTCACGTGACTAGCGCATAGTTAAG 472
YS_P._viridicuprus_YW04_1___         GAAAAATCGAAGCGGGAGCGATGGGGTCACGTGACTAGCGCATAGTTAAG 474
YS_P._hongwonpyoi_YW34_8____         GAAAAATCGAAGCGGGAGCGATGGAGTCACGTGACTAGCGCATAGTTAAG 473
YS_P._hongwonpyoi_YW34_2a___         GAAAAATCGAAGCGGGAGCGATGGAGTCACGTGACTAGCGCATAGTTAAG 475
YS_P._hongwonpyoi_YW33_8____         GAAAAATCGAAGCGGGAGCGATGGGGTCACGTGACTAGCGCATAGTTAAG 473
YS_P._hongwonpyoi_YW24_1____         GAAAAATCGAAGCGGGAGCGATGGGGTCACGTGACTAGCGCATAGTTAAG 475
YS_P._hongwonpyoi_YW05_8____         GAAAAATCGAAGCGGGAGCGATGGGGTCACGTGACTAGCGCATAGTTAAG 475
YS_P._hongwonpyoi_YW35_8___          GAAAAATCGAAGCGGGAGCGATGGGGTCACGTGACTAGCGCATAGTTAAG 473
YS_P._hongwonpyoi_YW37_8____         GAAAAATCGAAGCGGGAGCGATGGGGTCACGTGACTAGCGCATAGTTAAG 475
YS_P._hongwonpyoi_YW36_1____         GAAAAATCGAAGCGGGAGCGATGGAGTCACGTGACTAGTGCATAGTTAAG 475
YS_P._sue_YW45_1____________         GAAAAATCGAAGCGGGAGCGATGAGGTCACGTGACTAGCGCATAGTTAAG 473
YS_P._sue_YW80_3____________         GAAAAATCGAAGCGGGAGCGATGAGGTCACGTGACTAGCGCATAGTTAAG 473
YS_P._takakuwai_YW43_2______         GAAAAATCGAAGCGGGAGCGATGAGGTCACGTGACTAGCGCATAGTTAAG 460
YS_P._urushiyamai_YW48_1____         GAAAAATCGAAGCGGGAGCGATGGGGTCACGTGACTAGCGCATAGTTAAG 473
YS_P._urushiyamai_YW50_1____         GAAAAATCGAAGCGGGAGCGATGGGGTCACGTGACTAGCGCATAGTTAAG 473
YS_P._urushiyamai_YW49_2____         GAAAAATCGAAGCGGGAGCGATGGGGTCACGTGACTAGCGCATAGTTAAG 473
YS_P._delicatulus_YW65_2____         GAAAAATCGAAGCGGGAGCGATGGGGTCACGTGACTAGGGCATAGTTAAG 474
YS_P._delicatulus_YW46_1____         GAAAAATCGAAGCGGGAGCGATGGGGTCACGTGACTAGCGCATAGTTAAG 474
YS_P._delicatulus_YW10_1____         GAAAAATCGAAGCGGGAGCGATGGGGTCACGTGACTAGCGCATAGTTAAG 474
YS_P._delicatulus_YW68_3____         GAAAAATCGAAGCGGGAGCGATGGGGTCACGTGACTAGCGCATAGTTAAG 474
YS_P._delicatulus_YW72_2____         GAAAAATCGAAGCGGGAGCGATGGGGTCACGTGACTAGCGCATAGTTAAG 474
YS_P._delicatulus_YW47_2____         GAAAAATCGAAGCGGGAGCGATGGGGTCACGTGACTAGCGCATAGTTAAG 474
YS_P._takakuwai_YW88_1______         GAAAAATCGAAGCGGGAGCGATGGGGTCACGTGACTAGAGCATAGTTAAG 473
YS_P._akitaorum_YW16_1______         GAAAAATCGAAGCGGGAGCGATGGGGTCACGTGACTAGAGCATAGTTAAG 473
YS_P._akitaorum_YW14_2______         GAAAAATCGAAGCGGGAGCGATGGGGTCACGTGACTAGAGCATAGTTAAG 473
YS_P._kawadai_YW12_1______           GAAAAATCGAAGCGGGAGCGACGGGGTCACGTGACTAGCGCATAGTTAAG 473
```

```
YS_P._takakuwai_YW13_1______        GAAAAATCGAAGCGGGAGCGACGGGGTCACGTGACTAGCGCATAGTTAAG 596
YS_P._albisomni_YW19_1______        GAAAAATCGAAGCGGGAGCGATGGGGTCACGTGACTAGCGCATAGTTAAG 473
YS_P._albisomni_YW21_1______        GAAAAATCGAAGCGGGAGCGATGGGGTCACGTGACTAGCGCATAGTTAAG 473
YS_P._albisomni_YW23_1______        GAAAAATCGAAGCGGGAGCGATGGGGTCACGTGACTAGCGCATAGTTAAG 471
YS_P._kawadai_YW03_1________        GAAAAATCGAAGCGGGAGCGATGGGGTCACGTGACTAGCGCATAGTTAAG 473
YS_P._takakuwai_YW54_1______        GAAAAATCGAAGCGGGAGCGATGGGGTCACGTGACTAGCGCATAGTTAAG 473
YS_P._takakuwai_YW73_3______        GAAAAATCGAAGCGGGAGCGATGGGGTCACGTGACTAGCGCATAGTTAAG 473
YS_P._acuticollis_YW07_8____        GAAAAATCGAAGCGGGAGCGATGGGGTCACGTGACTAGCGCATAGTTAAG 473
YS_P._albisomni_YW08_1______        GAAAAATCGAAGCGGGAGCGATGGGGTCACGTGACTAGCGCATAGTTAAG 473
YS_P._albisomni_YW09_1______        GAAAAATCGAAGCGGGAGCGATGGGGTCACGTGACTAGCGCATAGTTAAG 473
YS_P._takakuwai_YW38_1______        GAAAAATCGAAGCGGGAGCGATGGGGTCACGTGACTAGCGCATAGTTAAG 473
YS_P._angularis_YW25_8______        GAAAAATCGAAGCGGTAGCGATGAGATCACGTGACTAGCACATAGTTAAG 471
                                    *********** *** ***** *   ************  **********

YS_P._viridicuprus_YW75_3___        AAAGCGAAAAAAAGATTGCAGCACGAGAGTTTCGCGTATGGTCTCCCACT 524
YS_P._viridicuprus_YW76_3___        AAAGCGAAAAAAAGATTGCAGCACGAGAGTTTCGCGTATGGTCTCCCACT 524
YS_P._viridicuprus_YW78_3___        AAAGCGAAAAAAAGATTGCAGCACGAGAGTTTCGCGTATGGTCTCCCACT 524
YS_P._viridicuprus_YW58_1___        AAAGCGAAAAAAAGATTGCAGCACGAGAGTTTCGCGTATGGTCTCCCACT 522
YS_P._viridicuprus_YW04_1___        AAAGCGAAAAAAAGATTGCAGCACGAGAGTTTCGCGTATGGTCTCCCACT 524
YS_P._hongwonpyoi_YW34_8____        AAAGCGAAAAAAAGATTGCAGCACGAGAGTTTCGCGTATGGTCTCCCACT 523
YS_P._hongwonpyoi_YW34_2a___        AAAGCGAAAAAAAGATTGCAGCACGAGAGTTTCGCGTATGGTCTCCCACT 525
YS_P._hongwonpyoi_YW33_8____        AAAGCGAAAAAAAGATTGCAGCACGAGAGTTTCGCGTATGGTCTCCCACT 523
YS_P._hongwonpyoi_YW24_1____        AAAGCGAAAAAAAGATTGCAGCACGAGAGTTTCGCGTATGGTCTCCCACT 525
YS_P._hongwonpyoi_YW05_8____        AAAGCGAAAAAAAGATTGCAGCACGAGAGTTTCGCGTATGGTCTCCCACT 525
YS_P._hongwonpyoi_YW35_8___         AAAGCGAAAAAAAGATTGCAGCACGAGAGTTTCGCGTATGGTCTCCCACT 523
YS_P._hongwonpyoi_YW37_8____        AAAGCGAAAAAAAGATTGCAGCACGAGAGTTTCGCGTATGGTCTCCCACT 525
YS_P._hongwonpyoi_YW36_1____        AAAGCGAAAAAAAGATTGCAGCACGAGAGTTTCGCGTATGGTCTCCCACT 525
YS_P._sue_YW45_1____________        AAAGCGAAAAAAAGATTGCAGCACGAGAGTTTCGCGTATGGTCTCCCACT 523
YS_P._sue_YW80_3____________        AAAGCGAAAAAAAGATTGCAGCACGAGAGTTTCGCGTATGGTCTCCCACT 523
YS_P._takakuwai_YW43_2______        AAAGCGAAAAAAAGATTGCAGCACGAGAGTTTCGCGTATGGTCTCCCACT 510
YS_P._urushiyamai_YW48_1____        AAAGCGAAAAAAAGATTGCAGCACGAGAGTTTCGCGTATGGTCTCCCACT 523
YS_P._urushiyamai_YW50_1____        AAAGCGAAAAAAAGATTGCAGCACGAGAGTTTCGCGTATGGTCTCCCACT 523
YS_P._urushiyamai_YW49_2____        AAAGCGAAAAAAAGATTGCAGCACGAGAGTTTCGCGTATGGTCTCCCACT 523
YS_P._delicatulus_YW65_2____        AAAGCGAAAAAAAGATTGCAGCACGAGAGTTTCGCGTATGGTCTCCCACT 524
YS_P._delicatulus_YW46_1____        AAAGCGAAAAAAAGATTGCAGCACGAGAGTTTCGCGTATGGTCTCCCACT 524
YS_P._delicatulus_YW10_1____        AAAGCGAAAAAAAGATTGCAGCACGAGAGTTTCGCGTATGGTCTCCCACT 524
```

YS_P._delicatulus_YW68_3____ AAAGCGAAAAAAAGATTGCAGCACGAGAGTTTCGCGTATGGTCTCCCACT 524
YS_P._delicatulus_YW72_2____ AAAGCGAAAAAAAGATTGCAGCACGAGAGTTTCGCGTATGGTCTCCCACT 524
YS_P._delicatulus_YW47_2____ AAAGCGAAAAAAAGATTGCAGCACGAGAGTTTCGCGTATGGTCTCCCACT 524
YS_P._takakuwai_YW88_1______ AAAGCGAAAAAAAGATTGCAGCACGAGAGTTTCGCGTATGGTCTCCCACT 523
YS_P._akitaorum_YW16_1______ AAAGCGAAAAAAAGATTGCAGCACGAGAGTTTCGCGTATGGTCTCCCACT 523
YS_P._akitaorum_YW14_2______ AAAGCGAAAAAAAGATTGCAGCACGAGAGTTTCGCGTATGGTCTCCCACT 523
YS_P._kawadai_YW12_1______ AAAGCGAAAAAAAGATTGCAGCACGAGAGTTTCGCGTATGGTCTCCCACT 523
YS_P._takakuwai_YW13_1______ AAAGCGAAAAAAAGATTGCAGCACGAGAGTTTCGCGTATGGTCTCCCACT 646
YS_P._albisomni_YW19_1______ AAAGCGAAAAAAAGATTGCAGCACGAGAGTTTCGCGTATGGTCTCCCACT 523
YS_P._albisomni_YW21_1______ AAAGCGAAAAAAAGATTGCAGCACGAGAGTTTCGCGTATGGTCTCCCACT 523
YS_P._albisomni_YW23_1______ AAAGCGAAAAAAAGATTGCAGCACGAGAGTTTCGCGTATGGTCTCCCACT 521
YS_P._kawadai_YW03_1________ AAAGCGAAAAAAAGATTGCAGCACGAGAGTTTCGCGTATGGTCTCCCACT 523
YS_P._takakuwai_YW54_1______ AAAGCGAAAAAAAGATTGCAGCACGAGAGTTTCGCGTATGGTCTCCCACT 523
YS_P._takakuwai_YW73_3______ AAAGCGAAAAAAAGATTGCAGCACGAGAGTTTCGCGTATGGTCTCCCACT 523
YS_P._acuticollis_YW07_8____ AAAGCGAAAAAAAGATTGCAGCACGAGAGTTTCGCGTATGGTCTCCCACT 523
YS_P._albisomni_YW08_1______ AAAGCGAAAAAAAGATTGCAGCACGAGAGTTTCGCGTATGGTCTCCCACT 523
YS_P._albisomni_YW09_1______ AAAGCGAAAAAAAGATTGCAGCACGAGAGTTTCGCGTATGGTCTCCCACT 523
YS_P._takakuwai_YW38_1______ AAAGCGAAAAAAAGATTGCAGCACGAGAGTTTCGCGTATGGTCTCCCACT 523
YS_P._angularis_YW25_8______ AAAGCGAAAAAAAGATTGCAGCACGAGAGTTTCGCGTATGGTCTCCCACT 521
****

YS_P._viridicuprus_YW75_3___ ACACTACTCGGTCTCGCTCTTAGCAGCTTAACTACAGTTGATCGGACGGG 574
YS_P._viridicuprus_YW76_3___ ACACTACTCGGTCTCGCTCTTAGCAGCTTAACTACAGTTGATCGGACGGG 574
YS_P._viridicuprus_YW78_3___ ACACTACTCGGTCTCGCTCTTAGCAGCTTAACTACAGTTGATCGGACGGG 574
YS_P._viridicuprus_YW58_1___ ACACTACTCGGTCTCGCTCTTAGCAGCTTAACTACAGTTGATCGGACGGG 572
YS_P._viridicuprus_YW04_1___ ACACTACTCGGTCTCGCTCTTAGCAGCTTAACTACAGTTGATCGGACGGG 574
YS_P._hongwonpyoi_YW34_8____ ACACTACTCGGTCTCGCTCTTAGCAGCTTAACTACAGTTGATCGGACGGG 573
YS_P._hongwonpyoi_YW34_2a___ ACACTACTCGGTCTCGCTCTTAGCAGCTTAACTACAGTTGATCGGACGGG 575
YS_P._hongwonpyoi_YW33_8____ ACACTACTCGGTCTCGCTCTTAGCAGCTTAACTACAGTTGATCGGACGGG 573
YS_P._hongwonpyoi_YW24_1____ ACACTACTCGGTCTCGCTCTTAGCAGCTTAACTACAGTTGATCGGACGGG 575
YS_P._hongwonpyoi_YW05_8____ ACACTACTCGGTCTCGCTCTTAGCAGCTTAACTACAGTTGATCGGACGGG 575
YS_P._hongwonpyoi_YW35_8___ ACACTACTCGGTCTCGCTCTTAGCAGCTTAACTACAGTTGATCGGACGGG 573
YS_P._hongwonpyoi_YW37_8____ ACACTACTCGGTCTCGCTCTTAGCAGCTTAACTACAGTTGATCGGACGGG 575
YS_P._hongwonpyoi_YW36_1____ ACACTACTCGGTCTCGCTCTTAGCAGCTTAACTACAGTTGATCGGACGGG 575
YS_P._sue_YW45_1____________ ACACTACTCGGTCTCGCTCTTAGCAGCTTAACTACAGTTGATCGGACGGG 573
YS_P._sue_YW80_3____________ ACACTACTCGGTCTCGCTCTTAGCAGCTTAACTACAGTTGATCGGACGGG 573

```
YS_P._takakuwai_YW43_2______     ACACTACTCGGTCTCGCTCTTAGCAGCTTAACTACAGTTGATCGGACGGG 560
YS_P._urushiyamai_YW48_1____     ACACTACTCGGTCTCGCTCTTAGCAGCTTAACTACAGTTGATCGGACGGG 573
YS_P._urushiyamai_YW50_1____     ACACTACTCGGTCTCGCTCTTAGCAGCTTAACTACAGTTGATCGGACGGG 573
YS_P._urushiyamai_YW49_2____     ACACTACTCGGTCTCGCTCTTAGCAGCTTAACTACAGTTGATCGGACGGG 573
YS_P._delicatulus_YW65_2____     ACACTACTCGGTCTCGCTCTTAGCAGCTTAACTACAGTTGATCGGACGGG 574
YS_P._delicatulus_YW46_1____     ACACTACTCGGTCTCGCTCTTAGCAGCTTAACTACAGTTGATCGGACGGG 574
YS_P._delicatulus_YW10_1____     ACACTACTCGGTCTCGCTCTTAGCAGCTTAACTACAGTTGATCGGACGGG 574
YS_P._delicatulus_YW68_3____     ACACTACTCGGTCTCGCTCTTAGCAGCTTAACTACAGTTGATCGGACGGG 574
YS_P._delicatulus_YW72_2____     ACACTACTCGGTCTCGCTCTTAGCAGCTTAACTACAGTTGATCGGACGGG 574
YS_P._delicatulus_YW47_2____     ACACTACTCGGTCTCGCTCTTAGCAGCTTAACTACAGTTGATCGGACGGG 574
YS_P._takakuwai_YW88_1______     ACACTACTCGGTCTCGCTCTTAGCAGCTTAACTACAGTTGATCGGACGGG 573
YS_P._akitaorum_YW16_1______     ACACTACTCGGTCTCGCTCTTAGCAGCTTAACTACAGTTGATCGGACGGG 573
YS_P._akitaorum_YW14_2______     ACACTACTCGGTCTCGCTCTTAGCAGCTTAACTACAGTTGATCGGACGGG 573
YS_P._kawadai_YW12_1______       ACACTACTCGGTCTCGCTCTTAGCAGCTTAACTACAGTTGATCGGACGGG 573
YS_P._takakuwai_YW13_1______     ACACTACTCGGTCTCGCTCTTAGCAGCTTAACTACAGTTGATCGGACGGG 696
YS_P._albisomni_YW19_1______     ACACTACTCGGTCTCGCTCTTAGCAGCTTAACTACAGTTGATCGGACGGG 573
YS_P._albisomni_YW21_1______     ACACTACTCGGTCTCGCTCTTAGCAGCTTAACTACAGTTGATCGGACGGG 573
YS_P._albisomni_YW23_1______     ACACTACTCGGTCTCGCTCTTAGCAGCTTAACTACAGTTGATCGGACGGG 571
YS_P._kawadai_YW03_1________     ACACTACTCGGTCTCGCTCTTAGCAGCTTAACTACAGTTGATCGGACGGG 573
YS_P._takakuwai_YW54_1______     ACACTACTCGGTCTCGCTCTTAGCAGCTTAACTACAGTTGATCGGACGGG 573
YS_P._takakuwai_YW73_3______     ACACTACTCGGTCTCGCTCTTAGCAGCTTAACTACAGTTGATCGGACGGG 573
YS_P._acuticollis_YW07_8____     ACACTACTCGGTCTCGCTCTTAGCAGCTTAACTACAGTTGATCGGACGGG 573
YS_P._albisomni_YW08_1______     ACACTACTCGGTCTCGCTCTTAGCAGCTTAACTACAGTTGATCGGACGGG 573
YS_P._albisomni_YW09_1______     ACACTACTCGGTCTCGCTCTTAGCAGCTTAACTACAGTTGATCGGACGGG 573
YS_P._takakuwai_YW38_1______     ACACTACTCGGTCTCGCTCTTAGCAGCTTAACTACAGTTGATCGGACGGG 573
YS_P._angularis_YW25_8______     ACACTACTCGGTCTCGCTCTTAGCAGCTTAACTACAGTTGATCGGACGGG 571
                                 **************************************************

YS_P._viridicuprus_YW75_3___     AAACGGTGCTTTCTGCTAGATATGGCCGCAACCGAAATACATAAGATTCC 624
YS_P._viridicuprus_YW76_3___     AAACGGTGCTTTCTGCTAGATATGGCCGCAACCGAAATACATAAGATTCC 624
YS_P._viridicuprus_YW78_3___     AAACGGTGCTTTCTGCTAGATATGGCCGCAACCGAAATACATAAGATTCC 624
YS_P._viridicuprus_YW58_1___     AAACGGTGCTTTCTGCTAGATATGGCCGCAACCGAAATACATAAGATTCC 622
YS_P._viridicuprus_YW04_1___     AAACGGTGCTTTCTGCTAGATATGGCCGCAACCGAAATACATAAGATTCC 624
YS_P._hongwonpyoi_YW34_8____     AAACGGTGCTTTCTGCTAGATATGGCCGCAACCGAAATACATAAGATTCC 623
YS_P._hongwonpyoi_YW34_2a___     AAACGGTGCTTTCTGCTAGATATGGCCGCAACCGAAATACATAAGATTCC 625
YS_P._hongwonpyoi_YW33_8____     AAACGGTGCTTTCTGCTAGATATGGCCGCAACCGAAATACATAAGATTCC 623
```

```
YS_P._hongwonpyoi_YW24_1____       AAACGGTGCTTTCTGCTAGATATGGCCGCAACCGAAATACATAAGATTCC 625
YS_P._hongwonpyoi_YW05_8____       AAACGGTGCTTTCTGCTAGATATGGCCGCAACCGAAATACATAAGATTCC 625
YS_P._hongwonpyoi_YW35_8___        AAACGGTGCTTTCTGCTAGATATGGCCGCAACCGAAATACATAAGATTCC 623
YS_P._hongwonpyoi_YW37_8____       AAACGGTGCTTTCTGCTAGATATGGCCGCAACCGAAATACATAAGATTCC 625
YS_P._hongwonpyoi_YW36_1____       AAACGGTGCTTTCTGCTAGATATGGCCGCAACCGAAATACATAAGATTCC 625
YS_P._sue_YW45_1____________       AAACGGTGCTTTCTGCTAGATATGGCCGCAACCGAAATACATAAGATTCC 623
YS_P._sue_YW80_3____________       AAACGGTGCTTTCTGCTAGATATGGCCGCAACCGAAATACATAAGATTCC 623
YS_P._takakuwai_YW43_2______       AAACGGTGCTTTCTGCTAGATATGGCCGCAACCGAAATACATAAGATTCC 610
YS_P._urushiyamai_YW48_1____       AAACGGTGCTTTCTGCTAGATATGGCCGCAACCGAAATACATAAGATTCC 623
YS_P._urushiyamai_YW50_1____       AAACGGTGCTTTCTGCTAGATATGGCCGCAACCGAAATACATAAGATTCC 623
YS_P._urushiyamai_YW49_2____       AAACGGTGCTTTCTGCTAGATATGGCCGCAACCGAAATACATAAGATTCC 623
YS_P._delicatulus_YW65_2____       AAACGGTGCTTTCTGCTAGATATGGCCGCAACCGAAATACATAAGATTCC 624
YS_P._delicatulus_YW46_1____       AAACGGTGCTTTCTGCTAGATATGGCCGCAACCGAAATACATAAGATTCC 624
YS_P._delicatulus_YW10_1____       AAACGGTGCTTTCTGCTAGATATGGCCGCAACCGAAATACATAAGATTCC 624
YS_P._delicatulus_YW68_3____       AAACGGTGCTTTCTGCTAGATATGGCCGCAACCGAAATACATAAGATTCC 624
YS_P._delicatulus_YW72_2____       AAACGGTGCTTTCTGCTAGATATGGCCGCAACCGAAATACATAAGATTCC 624
YS_P._delicatulus_YW47_2____       AAACGGTGCTTTCTGCTAGATATGGCCGCAACCGAAATACATAAGATTCC 624
YS_P._takakuwai_YW88_1______       AAACGGTGCTTTCTGCTAGATATGGCCGCAACCGAAATACATAAGATTCC 623
YS_P._akitaorum_YW16_1______       AAACGGTGCTTTCTGCTAGATATGGCCGCAACCGAAATACATAAGATTCC 623
YS_P._akitaorum_YW14_2______       AAACGGTGCTTTCTGCTAGATATGGCCGCAACCGAAATACATAAGATTCC 623
YS_P._kawadai_YW12_1______         AAACGGTGCTTTCTGCTAGATATGGCCGCAACCGAAATACATAAGATTCC 623
YS_P._takakuwai_YW13_1______       AAACGGTGCTTTCTGCTAGATATGGCCGCAACCGAAATACATAAGATTCC 746
YS_P._albisomni_YW19_1______       AAACGGTGCTTTCTGCTAGATATGGCCGCAACCGAAATACATAAGATTCC 623
YS_P._albisomni_YW21_1______       AAACGGTGCTTTCTGCTAGATATGGCCGCAACCGAAATACATAAGATTCC 623
YS_P._albisomni_YW23_1______       AAACGGTGCTTTCTGCTAGATATGGCCGCAACCGAAATACATAAGATTCC 621
YS_P._kawadai_YW03_1________       AAACGGTGCTTTCTGCTAGATATGGCCGCAACCGAAATACATAAGATTCC 623
YS_P._takakuwai_YW54_1______       AAACGGTGCTTTCTGCTAGATATGGCCGCAACCGAAATACATAAGATTCC 623
YS_P._takakuwai_YW73_3______       AAACGGTGCTTTCTGCTAGATATGGCCGCAACCGAAATACATAAGATTCC 623
YS_P._acuticollis_YW07_8____       AAACGGTGCTTTCTGCTAGATATGGCCGCAACCGAAATACATAAGATTCC 623
YS_P._albisomni_YW08_1______       AAACGGTGCTTTCTGCTAGATATGGCCGCAACCGAAATACATAAGATTCC 623
YS_P._albisomni_YW09_1______       AAACGGTGCTTTCTGCTAGATATGGCCGCAACCGAAATACATAAGATTCC 623
YS_P._takakuwai_YW38_1______       AAACGGTGCTTTCTGCTAGATATGGCCGCAACCGAAATACATAAGATTCC 623
YS_P._angularis_YW25_8______       AAACGGTGCTTTCTGCTAGATATGGCCGCAACCGAAATACATAAGATTCC 621
                                   **************************************************

YS_P._viridicuprus_YW75_3___       AGCGTACCATATGGAAACCGGGGGG---TCTTAGGTTCATTATTTCGAAA 671
```

```
YS_P._viridicuprus_YW76_3___      AGCGTACCATATGGAAACCGGGGGG---TCTTAGGTTCATTATTTCGAAA 671
YS_P._viridicuprus_YW78_3___      AGCGTACCATATGGAAACCGGGGGG---TCTTAGGTTCATTATTTCGAAA 671
YS_P._viridicuprus_YW58_1___      AGCGTACCATATGGAAACCGGGGGG---TCTTAGGTTCATTATTTCGAAA 669
YS_P._viridicuprus_YW04_1___      AGCGTACCATATGGAAACCGGGGGG---TCTTAGGTTCATTATTTCGAAA 671
YS_P._hongwonpyoi_YW34_8____      AGCGTACCATATGGAAACCGGGGGG---TCTTAGGTTCATTATTTCGAAA 670
YS_P._hongwonpyoi_YW34_2a___      AGCGTACCATATGGAAACCGGGGGG---TCTTAGGTTCATTATTTCGAAA 672
YS_P._hongwonpyoi_YW33_8____      AGCGTACCATATGGAAACCGGGGGG---TCTTAGGTTCATTATTTCGAAA 670
YS_P._hongwonpyoi_YW24_1____      AGCGTACCATATGGAAACCGGGGGG---TCTTAGGTTCATTATTTCGAAA 672
YS_P._hongwonpyoi_YW05_8____      AGCGTACCATATGGAAACCGGGGGG---TCTTAGGTTCATTATTTCGAAA 672
YS_P._hongwonpyoi_YW35_8___       AGCGTACCATATGGAAACCGGGGGG---TCTTAGGTTCATTATTTCGAAA 670
YS_P._hongwonpyoi_YW37_8____      AGCGTACCATATGGAAACCGGGGGG---TCTTAGGTTCATTATTTCGAAA 672
YS_P._hongwonpyoi_YW36_1____      AGCGTACCATATGGAAACCGGGGGG---TCTTAGGTTCATTATTTCGAAA 672
YS_P._sue_YW45_1____________      AGCGTACTATACGGAAACCGGGGGG---TCTTAGGTTCATTATTTCGAAA 670
YS_P._sue_YW80_3____________      AGCGTACTATACGGAAACCGGGGGG---TCTTAGGTTCATTATTTCGAAA 670
YS_P._takakuwai_YW43_2______      AGCGTACTATACGGAAACCGGGGGG---TCTTAGGTTCATTATTTCGAAA 657
YS_P._urushiyamai_YW48_1____      AGCGTACCATACGGAAACCGGGGGG---TCTTAGGTTCATTATTTCGAAA 670
YS_P._urushiyamai_YW50_1____      AGCGTACCATACGGAAACCGGGGGG---TCTTAGGTTCATTATTTCGAAA 670
YS_P._urushiyamai_YW49_2____      AGCGTACCATACGGAAACCGGGGGG---TCTTAGGTTCATTATTTCGAAA 670
YS_P._delicatulus_YW65_2____      AGCGTACCATATGGAAACCGGGGGGG--TCTTAGGTTCATTATTTCGAAA 672
YS_P._delicatulus_YW46_1____      AGCGTACCATATGGAAACCGGGGGGG--TCTTAGGTTCATTATTTCGAAA 672
YS_P._delicatulus_YW10_1____      AGCGTACCATATGGAAACCGGGGGGG--TCTTAGGTTCATTATTTCGAAA 672
YS_P._delicatulus_YW68_3____      AGCGTACCATATGGAAACCGGGGGGGG-TCTTAGGTTCATTATTTCGAAA 673
YS_P._delicatulus_YW72_2____      AGCGTACCATATGGAAACCGGGGGGGGGTCTTAGGTTCATTATTTCGAAA 674
YS_P._delicatulus_YW47_2____      AGCGTACCATATGGAAACCGGGGGGG--TCTTAGGTTCATTATTTCGAAA 672
YS_P._takakuwai_YW88_1______      AGCGTACCATATGGAAACCGGGGGG---TCTTAGGTTCATTATTTCGAAA 670
YS_P._akitaorum_YW16_1______      AGCGTACCATATGGAAACCGGGGGG---TCTTAGGTTCATTATTTCGAAA 670
YS_P._akitaorum_YW14_2______      AGCGTACCATATGGAAACCGGGGGG---TCTTAGGTTCATTATTTCGAAA 670
YS_P._kawadai_YW12_1______        AGCGTACCATATGGAAACCGGGGGG---TCTTAGGTTCATTATTTCGAAA 670
YS_P._takakuwai_YW13_1______      AGCGTACCATATGGAAACCGGGGGG---TCTTAGGTTCATTATTTCGAAA 793
YS_P._albisomni_YW19_1______      AGCGTACCATATGGAAACCGGGGGG---TCTTAGGTTCATTATTTCGAAA 670
YS_P._albisomni_YW21_1______      AGCGTACCATATGGAAACCGGGGGG---TCTTAGGTTCATTATTTCGAAA 670
YS_P._albisomni_YW23_1______      AGCGTACCATATGGAAACCGGGGGG---TCTTAGGTTCATTATTTCGAAA 668
YS_P._kawadai_YW03_1________      AGCGTACCATATGGAAACCGGGGGG---TCTTAGGTTCATTATTTCGAAA 670
YS_P._takakuwai_YW54_1______      AGCGTACCATATGGAAACCGGGGGG---TCTTAGGTTCATTATTTCGAAA 670
YS_P._takakuwai_YW73_3______      AGCGTACCATATGGAAACCGGGGGG---TCTTAGGTTCATTATTTCGAAA 670
YS_P._acuticollis_YW07_8____      AGCGTACCATATGGAAACCGGGGGG---TCTTAGGTTCATTATTTCGAAA 670
```

```
YS_P._albisomni_YW08_1______        AGCGTACCATATGGAAACCGGGGGG---TCTTAGGTTCATTATTTCGAAA 670
YS_P._albisomni_YW09_1______        AGCGTACCATATGGAAACCGGGGGG---TCTTAGGTTCATTATTTCGAAA 670
YS_P._takakuwai_YW38_1______        AGCGTACCATATGGAAACCGGGGGG---TCTTAGGTTCATTATTTCGAAA 670
YS_P._angularis_YW25_8______        AGCGCACTATATGGAAACCGGGGGG---TCTTAGGTTCATTATTTCGAAA 668
                                    **** ** *** *************   *********************

YS_P._viridicuprus_YW75_3___        AATCAAGGGGGGA-ATACCACA-TATTTGGGGATTCAGGTATGTTAAGTA 719
YS_P._viridicuprus_YW76_3___        AATCAAGGGGGGA-ATACCACA-TATTTGGGGATTCAGGTATGTTAAGTA 719
YS_P._viridicuprus_YW78_3___        AATCAAGGGGGGA-ATACCACA-TATTTGGGGATTCAGGTATGTTAAGTA 719
YS_P._viridicuprus_YW58_1___        AATCAAGGGGGGA-ATACCACA-TATTTGGGGATTCAGGTATGTTAAGTA 717
YS_P._viridicuprus_YW04_1___        AATCAAGGGGGGA-ATACCACA-TATTTGGGGATTCAGGTATGTTAAGTA 719
YS_P._hongwonpyoi_YW34_8____        AATCAAGGGGGGA-ATACCACAATATTTGGGGATTCAGGTATGTTAAGTA 719
YS_P._hongwonpyoi_YW34_2a___        AATCAAGGGGGGA-ATACCACAATATTTGGGGATTCAGGTATGTTAAGTA 721
YS_P._hongwonpyoi_YW33_8____        AATCAAGGGGGGA-ATACCACA-TATTTGGGGATTCAGGTATGTTAAGTA 718
YS_P._hongwonpyoi_YW24_1____        AATCAAGGGGGGA-ATACCACA-TATTTGGGGATTCAGGTATGTTAAGTA 720
YS_P._hongwonpyoi_YW05_8____        AATCAAGGGGGGA-ATACCACA-TATTTGGGGATTCAGGTATGTTAAGTA 720
YS_P._hongwonpyoi_YW35_8___         AATCAAGGGGGGA-ATACCACA-TATTTGGGGATTCAGGTATGTTAAGTA 718
YS_P._hongwonpyoi_YW37_8____        AATCAAGGGGGGA-ATACCACA-TATTTGGGGATTCAGGTATGTTAAGTA 720
YS_P._hongwonpyoi_YW36_1____        AATCAAGGGGGGA-ATACCACA-TATTTGGGGATTCAGGTATGTTAAGTA 720
YS_P._sue_YW45_1____________        TATCAAGGGGGGG-ATACCACA-TATTTGGGGATTCAGGTATGTTAAGTA 718
YS_P._sue_YW80_3____________        TATCAAGGGGGGG-ATACCACA-TATTTGGGGATTCAGGTATGTTAAGTA 718
YS_P._takakuwai_YW43_2______        TATCAAGGGGGGG-ATACCACA-TATTTGGGGATTCAGGTATGTTAAGTA 705
YS_P._urushiyamai_YW48_1____        TATCAAGGGGGGG-ATACCACA-TATTTGGGGATTCAGGTATGTTAAGTA 718
YS_P._urushiyamai_YW50_1____        TATCAAGGGGGGG-ATACCACA-TATTTGGGGATTCAGGTATGTTAAGTA 718
YS_P._urushiyamai_YW49_2____        TATCAAGGGGGGG-ATACCACA-TATTTGGGGATTCAGGTATGTTAAGTA 718
YS_P._delicatulus_YW65_2____        TATCAAGGGGGGG-ATACCACA-TATTTGGGGATTCAGGTATGTTAAGTA 720
YS_P._delicatulus_YW46_1____        TATCAAGGGGGGG-ATACCACA-TATTTGGGGATTCAGGTATGTTAAGTA 720
YS_P._delicatulus_YW10_1____        TATCAAGGGGGGG-ATACCACA-TATTTGGGGATCCAGGTATGTTAAGTA 720
YS_P._delicatulus_YW68_3____        TATCAAGGGGAGG-ATACCACA-TATTTGGGGATTCAGGTATGTTAAGTA 721
YS_P._delicatulus_YW72_2____        TATCAAGGGGAGG-ATACCACA-TATTTGGGGATTCAGGTATGTTAAGTA 722
YS_P._delicatulus_YW47_2____        TATCAAGGGGGGG-ATACCACA-TATTTGGGGATTCAGGTATGTTAAGTA 720
YS_P._takakuwai_YW88_1______        TATCAAGGGGGGG-ATACCACA-TATTTGGGGATTCAGGTATGTTAAGTA 718
YS_P._akitaorum_YW16_1______        TATCAAGGGGGGG-ATACCACA-TATTTGGGGATTCAGGTATGTTAAGTA 718
YS_P._akitaorum_YW14_2______        TATCAAGGGGGGG-ATACCACA-TATTTGGGGATTCAGGTATGTTAAGTA 718
YS_P._kawadai_YW12_1______          TATCAAGGGGGGG-ATACCACA-TATTTGGGGATTCAGGTATGTTAAGTA 718
YS_P._takakuwai_YW13_1______        TATCAAGGGGGGG-ATACCACA-TATTTGGGGATTCAGGTATGTTAAGTA 841
```

```
YS_P._albisomni_YW19_1______        TATCAAGGGGGGG-ATACCACA-TATTTGGGGATTCAGGTATGTTAAGTA 718
YS_P._albisomni_YW21_1______        TATCAAGGGGGGG-ATACCACA-TATTTGGGGATTCAGGTATGTTAAGTA 718
YS_P._albisomni_YW23_1______        TATCAAGGGGGGG-ATACCACA-TATTTGGGGATTCAGGTATGTTAAGTA 716
YS_P._kawadai_YW03_1________        TATCAAGGGGGGG-ATACCACA-TATTTGGGGATTCAGGTATGTCAAGTA 718
YS_P._takakuwai_YW54_1______        TATCAAGGGGGGG-ATACCACA-TATTTGGGGATTCAGGTATGTTAAGTA 718
YS_P._takakuwai_YW73_3______        TATCAAGGGGGGGGATACCACA-TATTTGGGGATTCAGGTATGTTAAGTA 719
YS_P._acuticollis_YW07_8____        TATCAAGGGGGGG-ATACCACA-TATTTGGGGATTCAGGTATGTTAAGTA 718
YS_P._albisomni_YW08_1______        TATCAAGGGGGGA--TACCACA-TATTTGGGGATTCAGGTATGTTAAGTA 717
YS_P._albisomni_YW09_1______        TATCAAGGGGGGG-ATACCACA-TATTTGGGGATTCAGGTATGTTAAGTA 718
YS_P._takakuwai_YW38_1______        TATCAAGGGGGGG-ATACCACA-TATTTGGGGATTCAGGTATGTTAAGTA 718
YS_P._angularis_YW25_8______        AATTAAGGGGAGGAATACCACA-TATTTGGGGATTCAGGTATGCTAAGTA 717
                                     ** ****** *   ******* *********** ********  *****

YS_P._viridicuprus_YW75_3___        TGATGAGGAACCTACACAAGCCTACAAGTACATACTGGACAGAGATAGAG 769
YS_P._viridicuprus_YW76_3___        TGATGAGGAACCTACACAAGCCTACAAGTACATACTGGACAGAGATAGAG 769
YS_P._viridicuprus_YW78_3___        TGATGAGGAACCTACACAAGCCTACAAGTACATACTGGACAGAGATAGAG 769
YS_P._viridicuprus_YW58_1___        TGATGAGGAACCTACACAAGCCTACAAGTACATACTGGACAGAGATAGAG 767
YS_P._viridicuprus_YW04_1___        TGATGAGGAACCTACACAAGCCTACAAGTACATACTGGACAGAGATAGAG 769
YS_P._hongwonpyoi_YW34_8____        TGATGAGGAACCTACACAAGCCTACAAGTACATACTGGACAGAGATAGAG 769
YS_P._hongwonpyoi_YW34_2a___        TGATGAGGAACCTACACAAGCCTACAAGTACATACTGGACAGAGATAGAG 771
YS_P._hongwonpyoi_YW33_8____        TGATGAGGAACCTACACAAGCCTACAAGTACATACTGGACAGAGATAGAG 768
YS_P._hongwonpyoi_YW24_1____        TGATGAGGAACCTACACAAGCCTACAAGTACATACTGGACAGAGATAGAG 770
YS_P._hongwonpyoi_YW05_8____        TGATGAGGAACCTACACAAGCCTACAAGTACATACTGGACAGAGATAGAG 770
YS_P._hongwonpyoi_YW35_8___         TGATGAGGAACCTACACAAGCCTACAAGTACATACTGGACAGAGATAGAG 768
YS_P._hongwonpyoi_YW37_8____        TGATGAGGAACCTACACAAGCCTACAAGTACATACTGGACAGAGATAGAG 770
YS_P._hongwonpyoi_YW36_1____        TGATGAGGAACCTACACAAGCTTACAAGTACATACTGGACAGAGATAGAG 770
YS_P._sue_YW45_1____________        TGACGAGGAACCTACACAAGCCTACAAGTACATACTGGACAGAGATAGAG 768
YS_P._sue_YW80_3____________        TGACGAGGAACCTACACAAGCCTACAAGTACATACTGGACAGAGATAGAG 768
YS_P._takakuwai_YW43_2______        TGACGAGGAACCTACACAAGCCTACAAGTACATACTGGACAGAGATAGAG 755
YS_P._urushiyamai_YW48_1____        TGATGAGGAACCTACACAAGCCTACAAGTACATACTGGACAGAGATAGAG 768
YS_P._urushiyamai_YW50_1____        TGATGAGGAACCTACACAAGCCTACAAGTACATACTGGACAGAGATAGAG 768
YS_P._urushiyamai_YW49_2____        TGATGAGGAACCTACACAAGCCTACAAGTACATACTGGACAGAGATAGAG 768
YS_P._delicatulus_YW65_2____        TGATGAGGAACCTACACAAGCCTACAAGTACATACTGGACAGAGATAGAG 770
YS_P._delicatulus_YW46_1____        TGATGAGGAACCTACACAAGCCTACAAGTACATACTGGACAGAGATAGAG 770
YS_P._delicatulus_YW10_1____        TGATGAGGAACCTACACAAGCCTACAAGTACATACTGGACAGAGATAGAG 770
YS_P._delicatulus_YW68_3____        TGATGAGGAACCTACACAAGCCTACAAGTACATACTGGACAGAGATAGAG 771
```

```
YS_P._delicatulus_YW72_2____        TGATGAGGAACCTACACAAGCCTACAAGTACATACTGGACAGAGATAGAG 772
YS_P._delicatulus_YW47_2____        TGATGAGGAACCTACACAAGCCTACAAGTACATACTGGACAGAGATAGAG 770
YS_P._takakuwai_YW88_1______        TGATGAGGAACCTACACAAGCCTACAAGTACATACTGGACAGAGATAGAG 768
YS_P._akitaorum_YW16_1______        TGATGAGGAACCTACACAAGCCTACAAGTACATACTGGACAGAGATAGAG 768
YS_P._akitaorum_YW14_2______        TGATGAGGAACCTACACAAGCCTACAAGTACATACTGGACAGAGATAGAG 768
YS_P._kawadai_YW12_1______          TGATGAGGAACCTACACAAGCCTACGAGTACATACTGGACTGAGATAGAG 768
YS_P._takakuwai_YW13_1______        TGATGAGGAACCTACACAAGCCTACGAGTACATACTGGACTGAGATAGAG 891
YS_P._albisomni_YW19_1______        TGATGAGGAACCTACACAAGCCTACGAGTACATACTGGACTGAGATAGAG 768
YS_P._albisomni_YW21_1______        TGATGAGGAACCTACACAAGCCTACGAGTACATACTGGACTGAGATAGAG 768
YS_P._albisomni_YW23_1______        TGATGAGGAACCTACACAAGCCTACGAGTACATACTGGACTGAGATAGAG 766
YS_P._kawadai_YW03_1________        TGATGAGGAACCTACACAAGCCTACGAGTACATACTGGACTGAGATAGAG 768
YS_P._takakuwai_YW54_1______        TGATGAGGAACCTACACAAGCCTACGAGTACATACTGGACTGAGATAGAG 768
YS_P._takakuwai_YW73_3______        TGATGAGGAACCTACACAAGCCTACGAGTACATACTGGACTGAGATAGAG 769
YS_P._acuticollis_YW07_8____        TGATGAGGAACCTACACAAGCCTACGAGTACATACTGGACTGAGATAGAG 768
YS_P._albisomni_YW08_1______        TGATGAGGAACCTACACAAGCCTACGAGTACATACTGGACTGAGATAGAG 767
YS_P._albisomni_YW09_1______        TGATGAGGAACCTACACAAGCCTACGAGTACATACTGGACTGAGATAGAG 768
YS_P._takakuwai_YW38_1______        TGATGAGGAACCTACACAAGCCTACGAGTACATACTGGACTGAGATAGAG 768
YS_P._angularis_YW25_8______        TGATCAGGAACCTACACATGCCTACAAGTACATACTGAACAGAGAAAGAG 767
                                    ***  ************* ** *** *********** ** **** ****

YS_P._viridicuprus_YW75_3___        AAAGGAATAGAGTATTGGAAGAAATGTAATTTATATTTGTAAAATTGACG 819
YS_P._viridicuprus_YW76_3___        AAAGGAATAGAGTATTGGAAGAAATGTAATTTATATTTGTAAAATTGACG 819
YS_P._viridicuprus_YW78_3___        AAAGGAATAGAGTATTGGAAGAAATGTAATTTATATTTGTAAAATTGACG 819
YS_P._viridicuprus_YW58_1___        AAAGGAATAGAGTATTGGAAGAAATGTAATTTATATTTGTAAAATTGACG 817
YS_P._viridicuprus_YW04_1___        AAAGGAATAGAGTATTGGAAGAAATGTAATTTATATTTGTAAAATTGACG 819
YS_P._hongwonpyoi_YW34_8____        AAAGGAATAGAGTATTGGAAGAAATGTAATTTATATTTGTAAAATTGACG 819
YS_P._hongwonpyoi_YW34_2a___        AAAGGAATAGAGTATTGGAAGAAATGTAATTTATATTTGTAAAATTGACG 821
YS_P._hongwonpyoi_YW33_8____        AAAGGAATAGAGTATTGGAAGAAATGTAATTTATATTTGTAAAATTGACG 818
YS_P._hongwonpyoi_YW24_1____        AAAGGAATAGAGTATTGGAAGAAATGTAATTTATATTTGTAAAATTGACG 820
YS_P._hongwonpyoi_YW05_8____        AAAGGAATAGAGTATTGGAAGAAATGTAATTTATATTTGTAAAATTGACG 820
YS_P._hongwonpyoi_YW35_8___         AAAGGAATAGAGTATTGGAAGAAATGTAATTTATATTTGTAAAATTGACG 818
YS_P._hongwonpyoi_YW37_8____        AAAGGAATAGAGTATTGGAAGAAATGTAATTTATATTTGTAAAATTGACG 820
YS_P._hongwonpyoi_YW36_1____        AAAGGAATAGAGTATTGGAAGAAATGTAATTTATATTTGTAAAATTGACG 820
YS_P._sue_YW45_1____________        AAAGGAATAGAGGATTGGAAGAAATGTAATTTATATTTGTAAAATTGACG 818
YS_P._sue_YW80_3____________        AAAGGAATAGAGGATTGGAAGAAATGTAATTTATATTTGTAAAATTGACG 818
YS_P._takakuwai_YW43_2______        AAAGGAATAGAGGATTGGAAGAAATGTAATTTATATTTGCAAAATTGACG 805
```

```
YS_P._urushiyamai_YW48_1____        AAAGGAATAGAGGATTGGAAGAAATGTAATTTATATTTGTAAAATTGACG 818
YS_P._urushiyamai_YW50_1____        AAAGGAATAGAGGATTGGAAGAAATGTAATTTATATTTGTAAAATTGACG 818
YS_P._urushiyamai_YW49_2____        AAAGGAATAGAGGATTGGAAGAAATGTAATTTATATTTGTAAAATTGACG 818
YS_P._delicatulus_YW65_2____        AAAGGAATAGAGGATTGGAAGAAATGTAATTTATATTTGTAAAATTGACG 820
YS_P._delicatulus_YW46_1____        AAAGGAATAGAGGATTGGAAGAAATGTAATTTATATTTGTAAAATTGACG 820
YS_P._delicatulus_YW10_1____        AAAGGAATAGAGGATTGGAAGAAATGTAATTTATATTTGTAAAATTGACG 820
YS_P._delicatulus_YW68_3____        AAAGGAATAGAGGATTGGAAGAAATGTAATTTATATTTGTAAAATTGACG 821
YS_P._delicatulus_YW72_2____        AAAGGAATAGAGGATTGGAAGAAATGTAATTTATATTTGTAAAATTGACG 822
YS_P._delicatulus_YW47_2____        AAAGGAATAGAGGATTGGAAGAAATGTAATTTATATTTGTAAAATTGACG 820
YS_P._takakuwai_YW88_1______        AAAGGAATAGAGGATTGGAAGAAATGTAATTTATATTTGTAAAATTGACG 818
YS_P._akitaorum_YW16_1______        AAAGGAATAGAGGATTGGAAGAAATGTAATTTATATTTGTAAAATTGACG 818
YS_P._akitaorum_YW14_2______        AAAGGAATAGAGGATTGGAAGAAATGTAATTTATATTTGTAAAATTGACG 818
YS_P._kawadai_YW12_1______          AAAGGAATAGAGGACTGGAAGAAATGTAATTTATATTTGTAAAATTGACG 818
YS_P._takakuwai_YW13_1______        AAAGGAATAGAGGACTGGAAGAAATGTAATTTATATTTGTAAAATTGACG 941
YS_P._albisomni_YW19_1______        AAAGGAATAGAGGACTGGAAGAAATGTAATTTATATTTGTAAAATTGACG 818
YS_P._albisomni_YW21_1______        AAAGGAATAGAGGACTGGAAGAAATGTAATTTATATTTGTAAAATTGACG 818
YS_P._albisomni_YW23_1______        AAAGGAATAGAGGACTGGAAGAAATGTAATTTATATTTGTAAAATTGACG 816
YS_P._kawadai_YW03_1________        AAAGGAATAGAGGACTGGAAGAAATGTAATTTATATTTGTAAAATTGACG 818
YS_P._takakuwai_YW54_1______        AAAGGAATAGAGGACTGGAAGAAATGTAATTTATATTTGTAAAATTGACG 818
YS_P._takakuwai_YW73_3______        AAAGGAATAGAGGACTGGAAGAAATGTAATTTATATTTGTAAAATTGACG 819
YS_P._acuticollis_YW07_8____        AAAGGAATAGAGGACTGGAAGAAATGTAATTTATATTTGTAAAATTGACG 818
YS_P._albisomni_YW08_1______        AAAGGAATAGAGGACTGGAAGAAATGTAATTTATATTTGTAAAATTGACG 817
YS_P._albisomni_YW09_1______        AAAGGAATAGAGGACTGGAAGAAATGTAATTTATATTTGTAAAATTGACG 818
YS_P._takakuwai_YW38_1______        AAAGGAATAGAGGACTGGAAGAAATGTAATTTATATTTGTAAAATTGACG 818
YS_P._angularis_YW25_8______        AAAGGAATAGAGGATTGGAAGAAATGTAATTTATATTTGTAAAATTGACG 817
                                    ************ * ************************ **********

YS_P._viridicuprus_YW75_3___        GAAATGTGACAAAGAATGAAATATAGATGATTGTGTTATAGGAAGACAAA 869
YS_P._viridicuprus_YW76_3___        GAAATGTGACAAAGAATGAAATATAGATGATTGTGTTATAGGAAGACAAA 869
YS_P._viridicuprus_YW78_3___        GAAATGTGACAAAGAATGAAATATAGATGATTGTGTTATAGGAAGACAAA 869
YS_P._viridicuprus_YW58_1___        GAAATGTGACAAAGAATGAAATATAGATGATTGTGTTATAGGAAGACAAA 867
YS_P._viridicuprus_YW04_1___        GAAATGTGACAAAGAATGAAATATAGATGATTGTGTTATAGGAAGACAAA 869
YS_P._hongwonpyoi_YW34_8____        GAAATGTGACAAAGAATGGAATATAGATGATTGTGTTATAGGAAGACAAA 869
YS_P._hongwonpyoi_YW34_2a___        GAAATGTGACAAAGAATGGAATATAGATGATTGTGTTATAGGAAGACAAA 871
YS_P._hongwonpyoi_YW33_8____        GAAATGTGACAAAGAATGGAATATAGATGATTGTGTTATAGGAAGACAAA 868
YS_P._hongwonpyoi_YW24_1____        GAAATGTGACAAAGAATGGAATATAGATGATTGTGTTATAGGAAGACAAG 870
```

```
YS_P._hongwonpyoi_YW05_8____      GAAATGTGACAAAGAATGGAATATAGATGATTGTGTTATAGGAAGACAAA 870
YS_P._hongwonpyoi_YW35_8___       GAAATGTGACAAAGAATGGAATATAGATGATTGTGTTATAGGAAGACAAA 868
YS_P._hongwonpyoi_YW37_8____      GAAATGTGACAAAGAATGGAATATAGATGATTGTGTTATAGGAAGACAAA 870
YS_P._hongwonpyoi_YW36_1____      GAAATGTGACAAAGAATGGAATATAGATGATTGTGTTATAGGAAGACAAA 870
YS_P._sue_YW45_1____________      GAAATGTGACAAAGAATGGAATATAGATGATTGTGTTATAGGAAGACAAA 868
YS_P._sue_YW80_3____________      GAAATGTGACAAAGAATGGAATATAGATGATTGTGTTATAGGAAGACAAA 868
YS_P._takakuwai_YW43_2______      GAAATGTGACAAAGAATGGAATATAGATGATTGTGTTATAGGAAGACAAA 855
YS_P._urushiyamai_YW48_1____      GAAATGTGACAAAGAATGGAATATAGATGATTGTGTTATAGGAAGACAAA 868
YS_P._urushiyamai_YW50_1____      GAAATGTGACAAAGAATGGAATATAGATGATTGTGTTATAGGAAGACAAA 868
YS_P._urushiyamai_YW49_2____      GAAATGTGACAAAGAATGGAATATAGATGATTGTGTTATAGGAAGACAAA 868
YS_P._delicatulus_YW65_2____      GAAATGTGACAAAGAATGGAATATAGATGATTGTGTTATAGGAAGACAAA 870
YS_P._delicatulus_YW46_1____      GAAATGTGACAAAGAATGGAATATAGATGATTGTGTTATAGGAAGACAAA 870
YS_P._delicatulus_YW10_1____      GAAATGTGACAAAGAATGGAATATAGATGATTGTGTTATAGGAAGACAAA 870
YS_P._delicatulus_YW68_3____      GAAATGTGACAAAGAATGGAATATAGATGATTGTGTTATAGGAAGACAAA 871
YS_P._delicatulus_YW72_2____      GAAATGTGACAAAGAATGGAATATAGATGATTGTGTTATAGGAAGACAAA 872
YS_P._delicatulus_YW47_2____      GAAATGTGACAAAGAATGGAATATAGATGATTGTGTTATAGGAAGACAAA 870
YS_P._takakuwai_YW88_1______      GAAATGTGACAAAGAATGGAATATAGATGATTGTGTTATAGGAAGACAAA 868
YS_P._akitaorum_YW16_1______      GAAATGTGACAAAGAATGGAATATAGATGATTGTGTTATAGGAAGACAAA 868
YS_P._akitaorum_YW14_2______      GAAATGTGACAAAGAATGGAATATAGATGATTGTGTTATAGGAAGACAAA 868
YS_P._kawadai_YW12_1______        GAAATGTGACAAAGAATGGAATATAGATGATTGTGTTATAGGAAGACAAA 868
YS_P._takakuwai_YW13_1______      GAAATGTGACAAAGAATGGAATATAGATGATTGTGTTATAGGAAGACAAA 991
YS_P._albisomni_YW19_1______      GAAATGTGACAAAGAATGGAATATAGATGATTGTGTTATAGGAAGACAAA 868
YS_P._albisomni_YW21_1______      GAAATGTGACAAAGAATGGAATATAGATGATTGTGTTATAGGAAGACAAA 868
YS_P._albisomni_YW23_1______      GAAATGTGACAAAGAATGGAATATAGATGATTGTGTTATAGGAAGACAAA 866
YS_P._kawadai_YW03_1________      GAAATGTGACAAAGAATGGAATATAGATGATTGTGTTATAGGAAGACAAA 868
YS_P._takakuwai_YW54_1______      GAAATGTGACAAAGAATGGAATATAGATGATTGTGTTATAGGAAGACAAA 868
YS_P._takakuwai_YW73_3______      GAAATGTGACAAAGAATGGAATATAGATGATTGTGTTATAGGAAGACAAA 869
YS_P._acuticollis_YW07_8____      GAAATGTGACAAAGAATGGAATATAGATGATTGTGTTATAGGAAGACAAA 868
YS_P._albisomni_YW08_1______      GAAATGTGACAAAGAATGGAATATAGATGATTGTGTTATAGGAAGACAAA 867
YS_P._albisomni_YW09_1______      GAAATGTGACAAAGAATGGAATATAGATGATTGTGTTATAGGAAGACAAA 868
YS_P._takakuwai_YW38_1______      GAAATGTGACAAAGAATGGAATATAGATGATTGTGTTATAGGAAGACAAA 868
YS_P._angularis_YW25_8______      GAAATGTGACAAAGAATGGAATATAGATGATTGTGTTATAGGAAGACAAA 867
                                  ****************** ******************************

YS_P._viridicuprus_YW75_3___      GAGGTGGACAAGGCGGGTATTTATAAGTAGAGGGTAGACGAAGTGGAAAC 919
YS_P._viridicuprus_YW76_3___      GAGGTGGACAAGGCGGGTATTTATAAGTAGAGGGTAGACGAAGTGGAAAC 919
```

YS_P._viridicuprus_YW78_3___	GAGGTGGACAAGGCGGGTATTTATAAGTAGAGGGTAGACGAAGTGGAAAC	919
YS_P._viridicuprus_YW58_1___	GAGGTGGACAAGGCGGGTATTTATAAGTAGAGGGTAGACGAAGTGGAAAC	917
YS_P._viridicuprus_YW04_1___	GAGGTGGACAAGGCGGGTATTTATAAGTAGAGGGTAGACGAAGTGGAAAC	919
YS_P._hongwonpyoi_YW34_8____	GAGGTGGACAAGGCGGGTATTTATAAGTAGAGGGTAGACGAAGTGGAAAC	919
YS_P._hongwonpyoi_YW34_2a___	GAGGTGGACAAGGCGGGTATTTATAAGTAGAGGGTAGACGAAGTGGAAAC	921
YS_P._hongwonpyoi_YW33_8____	GAGGTGGACAAGGCGGGTATTTATAAGTAGAGGGTAGACGAAGTGGAAAC	918
YS_P._hongwonpyoi_YW24_1____	GAGGTGGACAAGGCGGGTATTTATAAGTAGAGGGTAGACGAAGTGGAAAC	920
YS_P._hongwonpyoi_YW05_8____	GAGGTGGACAAGGCGGGTATTTATAAGTAGAGGGTAGACGAAGTGGAAAC	920
YS_P._hongwonpyoi_YW35_8___	GAGGTGGACAAGGCGGGTATTTATAAGTAGAGGGTAGACGAAGTGGAAAC	918
YS_P._hongwonpyoi_YW37_8____	GAGGTGGACAAGGCGGGTATTTATAAGTAGAGGGTAGACGAAGTGGAAAC	920
YS_P._hongwonpyoi_YW36_1____	GAGGCGGACAAGGCGGGTATTTATAAGTAGAGGGTAGACGAAGTGGAAAC	920
YS_P._sue_YW45_1____________	GAGGTGGACAAGGCGGGTATTTATAAGTAAAGGGTAGACGAAGTGGAAAC	918
YS_P._sue_YW80_3____________	GAGGTGGACAAGGCGGGTATTTATAAGTAAAGGGTAGACGAAGTGGAAAC	918
YS_P._takakuwai_YW43_2______	GAGGTGGACAAGGCGGGTATTTATAAGTAAAGGGTAGACGAAGTGGAAAC	905
YS_P._urushiyamai_YW48_1____	GAGGTGGACAAGGCGGGTATTTATAAGTAGAGGGTAGACGAAGTGGAAAC	918
YS_P._urushiyamai_YW50_1____	GAGGTGGACAAGGCGGGTATTTATAAGTAGAGGGTAGACGAAGTGGAAAC	918
YS_P._urushiyamai_YW49_2____	GAGGTGGACAAGGCGGGTATTTATAAGTAGAGGGTAGACGAAGTGGAAAC	918
YS_P._delicatulus_YW65_2____	GAGGTGGACAAGGCGGGTATTTATAAGTAGAGGGTAGACGAAGTGGAAAC	920
YS_P._delicatulus_YW46_1____	GAGGTGGACAAGGCGGGTATTTATAAGTAGAGGGTAGACGAAGTGGAAAC	920
YS_P._delicatulus_YW10_1____	GAGGTGGACAAGGCGGGTATTTATAAGTAGAGGGTAGACGAAGTGGAAAC	920
YS_P._delicatulus_YW68_3____	GAGGTGGACAAGGCGGGTATTTATAAGTAGAGGGTAGACGAAGTGGAAAC	921
YS_P._delicatulus_YW72_2____	GAGGTGGACAAGGCGGGTATTTATAAGTAGAGGGTAGACGAAGTGGAAAC	922
YS_P._delicatulus_YW47_2____	GAGGTGGACAAGGCGGGTATTTATAAGTAGAGGGTAGACGAAGTGGAAAC	920
YS_P._takakuwai_YW88_1______	GAGGTGGACAAGGCGGGTATTTATAAGTAGAGGGTAGACGAAGTGGAAAC	918
YS_P._akitaorum_YW16_1______	GAGGTGGACAAGGCGGGTATTTATAAGTAGAGGGTAGACGAAGTGGAAAC	918
YS_P._akitaorum_YW14_2______	GAGGTGGACAAGGCGGGTATTTATAAGTAGAGGGTAGACGAAGTGGAAAC	918
YS_P._kawadai_YW12_1______	GAGGTGGACAAGGCGGGTATTTATAAGTAGAGGGTAGACGAAGTGGAAAC	918
YS_P._takakuwai_YW13_1______	GAGGTGGACAAGGCGGGTATTTATAAGTAGAGGGTAGACGAAGTGGAAAC	1041
YS_P._albisomni_YW19_1______	GAGGTGGACAAGGCGGGTATTTATAAGTAGAGGGTAGACGAAGTGGAAAC	918
YS_P._albisomni_YW21_1______	GAGGTGGACAAGGCGGGTATTTATAAGTAGAGGGTAGACGAAGTGGAAAC	918
YS_P._albisomni_YW23_1______	GAGGTGGACAAGGCGGGTATTTATAAGTAGAGGGTAGACGAAGTGGAAAC	916
YS_P._kawadai_YW03_1________	GAGGTGGACAAGGCGGGTATTTATAAGTAGAGGGTAGACGAAGTGGAAAC	918
YS_P._takakuwai_YW54_1______	GAGGTGGACAAGGCGGGTATTTATAAGTAGAGGGTAGACGAAGTGGAAAC	918
YS_P._takakuwai_YW73_3______	GAGGTGGACAAGGCGGGTATTTATAAGTAGAGGGTAGACGAAGTGGAAAC	919
YS_P._acuticollis_YW07_8____	GAGGTGGACAAGGCGGGTATTTATAAGTAGAGGGTAGACGAAGTGGAAAC	918
YS_P._albisomni_YW08_1______	GAGGTGGACAAGGCGGGTATTTATAAGTAGAGGGTAGACGAAGTGGAAAC	917

```
YS_P._albisomni_YW09_1______      GAGGTGGACAAGGCGGGTATTTATAAGTAGAGGGTAGACGAAGTGGAAAC 918
YS_P._takakuwai_YW38_1______      GAGGTGGACAAGGCGGGTATTTATAAGTAGAGGGTAGACGAAGTGGAAAC 918
YS_P._angularis_YW25_8______      GAGGTGGTCAAGGCGGGTATTTATAAGTAGAGGGTGGACGAAGTGGAAAC 917
                                  **** ** ********************* ***** **************

YS_P._viridicuprus_YW75_3___      CGATGGAAGCTAATGAGGCTTGACACAAAACACGGTTGCAACAAGAATAA 969
YS_P._viridicuprus_YW76_3___      TGATGGAAGCTAATGAGGCTTGACACAAAACACGGTTGCAACAAGAATAA 969
YS_P._viridicuprus_YW78_3___      TGATGGAAGCTAATGAGGCTTGACACAAAACACGGTTGCAACAAGAATAA 969
YS_P._viridicuprus_YW58_1___      TGATGGAAGCTAATGAGGCTTGACACAAAACACGGTTGCAACAAGAATAA 967
YS_P._viridicuprus_YW04_1___      TGATGGAAGCTAATGAGGCTTGACACAAAACACGGTTGCAACAAGAATAA 969
YS_P._hongwonpyoi_YW34_8____      TGATGGAAGCTAATGAGGCTTGACACAAAACACGGTTGCAACAAGAATAA 969
YS_P._hongwonpyoi_YW34_2a___      TGATGGAAGCTAATGAGGCTTGACACAAAACACGGTTGCAACAAGAATAA 971
YS_P._hongwonpyoi_YW33_8____      TGATGGAAGCTAATGAGGCTTGACACAAAACACGGTTGCAACAAGAATAA 968
YS_P._hongwonpyoi_YW24_1____      TGATGGAAGCTAATGAGGCTTGACACAAAACACGGTTGCAACAAGAATAA 970
YS_P._hongwonpyoi_YW05_8____      TGATGGAAGCTAATGAGGCTTGACACAAAACACGGTTGCAACAAGAATAA 970
YS_P._hongwonpyoi_YW35_8___       TGATGGAAGCTAATGAGGCTTGACACAAAACACGGTTGCAACAAGAATAA 968
YS_P._hongwonpyoi_YW37_8____      TGATGGAAGCTAATGAGGCTTGACACAAAACACGGTTGCAACAAGAATAA 970
YS_P._hongwonpyoi_YW36_1____      TGATGGAAGCTAATGAGGCTTGACACAAAACACGGTTGCAACAAGAATAA 970
YS_P._sue_YW45_1____________      TGATGGAAGCAAATGAGGCTTGACACAAAACACGGTTGCAACAAGAATAA 968
YS_P._sue_YW80_3____________      TGATGGAAGCAAATGAGGCTTGACACAAAACACGGTTGCAACAAGAATAA 968
YS_P._takakuwai_YW43_2______      TGATGGAAGCAAATGAGGCTTGACACAAAACACGGTTGCAACAAGAATAA 955
YS_P._urushiyamai_YW48_1____      TGATGGAAGCAAATGAGGCTTGACACAAAACACGGTTGCAACAAGAATAA 968
YS_P._urushiyamai_YW50_1____      TGATGGAAGCAAATGAGGCTTGACACAAAACACGGTTGCAACAAGAATAA 968
YS_P._urushiyamai_YW49_2____      TGATGGAAGCAAATGAGGCTTGACACAAAACACGGTTGCAACAAGAATAA 968
YS_P._delicatulus_YW65_2____      TGATGGAAGCATATGAGGCTTGACACAAAACACGGTTGCAACAAGAATAA 970
YS_P._delicatulus_YW46_1____      TGATGGAAGCATATGAGGCTTGACACAAAACACGGTTGCAACAAGAATAA 970
YS_P._delicatulus_YW10_1____      TGATGGAAGCATATGAGGCTTGACACAAAACACGGTTGCAACAAGAATAA 970
YS_P._delicatulus_YW68_3____      TGATGGAAGCATATAAGGCTTGACACAAAACACGGTTGCAACAAGAATAA 971
YS_P._delicatulus_YW72_2____      TGATGGAAGCATATAAGGCTTGACACAAAACACGGTTGCAACAAGAATAA 972
YS_P._delicatulus_YW47_2____      TGATGGAAGCATATAAGGCTTGACACAAAACACGGTTGCAACAAGAATAA 970
YS_P._takakuwai_YW88_1______      TGATGGAAGCAAATGAGGCTTGACACAAAACACGGTTGCAACAAGAATAA 968
YS_P._akitaorum_YW16_1______      TGATGGAAGCAAATGAGGCTTGACACAAAACACGGTTGCAACAAGAATAA 968
YS_P._akitaorum_YW14_2______      TGATGGAAGCAAATGAGGCTTGACACAAAACACGGTTGCAACAAGAATAA 968
YS_P._kawadai_YW12_1______        TGATGGAAGCAAATGAGGCTTGACACAAAACACGGTTGCAACAAGAATAA 968
YS_P._takakuwai_YW13_1______      TGATGGAAGCAAATGAGGCTTGACACAAAACACGGTTGCAACAAGAATAA 1091
YS_P._albisomni_YW19_1______      TGATGGAAGCAAATGAGGCTTGACACAAAACACGGTTGCAACAAGAATAA 968
```

```
YS_P._albisomni_YW21_1______       TGATGGAAGCAAATGAGGCTTGACACAAAACACGGTTGCAACAAGAATAA 968
YS_P._albisomni_YW23_1______       TGATGGAAGCAAATGAGGCTTGACACAAAACACGGTTGCAACAAGAATAA 966
YS_P._kawadai_YW03_1________       TGATGGAAGCAAATGAGGCTTGACACAAAACACGGTTGCAACAAGAATAA 968
YS_P._takakuwai_YW54_1______       TGATGGAAGCAAATGAGGCTTGACACAAAACACGGTTGCAACAAGAATAA 968
YS_P._takakuwai_YW73_3______       TGATGGAAGCAAATGAGGCTTGACACAAAACACGGTTGCAACAAGAATAA 969
YS_P._acuticollis_YW07_8____       TGATGGAAGCAAATGAGGCTTGACACAAAACACGGTTGCAACAAGAATAA 968
YS_P._albisomni_YW08_1______       TGATGGAAGCAAATGAGGCTTGACACAAAACACGGTTGCAACAAGAATAA 967
YS_P._albisomni_YW09_1______       TGATGGAAGCAAATGAGGCTTGACACAAAACACGGTTGCAACAAGAATAA 968
YS_P._takakuwai_YW38_1______       TGATGGAAGCAAATGAGGCTTGACACAAAACACGGTTGCAACAAGAATAA 968
YS_P._angularis_YW25_8______       TGATGGAAGCAAACGAAGCTTGACACAAAACACGGTTGCAACAAGAATAA 967
                                    *********  *  * *********************************

YS_P._viridicuprus_YW75_3___       CAAACAAAGGAA--TGTGCATGAGCGTGTTCAGCAGGTTAGGCAAAAAAA 1017
YS_P._viridicuprus_YW76_3___       CAAACAAAGGAA--TGTGCATGAGCGTGTTCAGCAGGTTAGGCAAAAAAA 1017
YS_P._viridicuprus_YW78_3___       CAAACAAAGGAA--TGTGCATGAGCGTGTTCAGCAGGTTAGGCAAAAAAA 1017
YS_P._viridicuprus_YW58_1___       CAAACAAAGGAA--TGTGCATGAGCGTGTTCAGCAGGTTAGGCAAAAAAA 1015
YS_P._viridicuprus_YW04_1___       CAAACAAAGGAA--TGTGCATGAGCGTGTTCAGCAGGTTAGGCAAAAAAA 1017
YS_P._hongwonpyoi_YW34_8____       CAAACAAAGGAA--TGTGCATGAGCGTGTTCAGCAGGTTAGGCAAAAAAA 1017
YS_P._hongwonpyoi_YW34_2a___       CAAACAAAGGAA--TGTGCATGAGCGTGTTCAGCAGGTTAGGCAAAAAAA 1019
YS_P._hongwonpyoi_YW33_8____       CAAACAAAGGAA--TGTGCATGAGCGTGTTCAGCAGGTTAGGCAAAAAAA 1016
YS_P._hongwonpyoi_YW24_1____       CAAACAAAGGAA--TGTGCATGAGCGTGTTCAGCAGGTTAGGCAAAAAAA 1018
YS_P._hongwonpyoi_YW05_8____       CAAACAAAGGAA--TGTGCATGAGCGTGTTCAGCAGGTTAGGCAAAAAAA 1018
YS_P._hongwonpyoi_YW35_8___        CAAACAAAGGAA--TGTGCATGAGCGTGTTCAGCAGGTTAGGCAAAAAAA 1016
YS_P._hongwonpyoi_YW37_8____       CAAACAAAGGAA--TGTGCATGAGCGTGTTCAGCAGGTTAGGCAAAAAAA 1018
YS_P._hongwonpyoi_YW36_1____       CAAACAAAGGAA--TGTGCATGAGCGTGTTCAGCAGGTTAGGCAAAAAAA 1018
YS_P._sue_YW45_1____________       CAAACAAAGGAA--TGTGCATGAGCGTGTTCAGCAGGTTAGGCAAAAAAA 1016
YS_P._sue_YW80_3____________       CAAACAAAGGAA--TGTGCATGAGCGTGTTCAGCAGGTTAGGCAAAAAAA 1016
YS_P._takakuwai_YW43_2______       CAAACAAAGGAA--TGTGCATGAGCGTGTTCAGCAGGTTAGGCAAAAAAA 1003
YS_P._urushiyamai_YW48_1____       CAAACAAAGGAA--TGTGCATGAGCGTGTTCAGCAGGTTAGGCAAAAAAA 1016
YS_P._urushiyamai_YW50_1____       CAAACAAAGGAA--TGTGCATGAGCGTGTTCAGCAGGTTAGGCAAAAAAA 1016
YS_P._urushiyamai_YW49_2____       CAAACAAAGGAA--TGTGCATGAGCGTGTTCAGCAGGTTAGGCAAAAAAA 1016
YS_P._delicatulus_YW65_2____       CAAACAAAGGAA--TGTGCATGAGCGTGTTCAGCAGGTTAGGCAAAAAAA 1018
YS_P._delicatulus_YW46_1____       CAAACAAAGGAA--TGTGCATGAGCGTGTTCAGCAGGTTAGGCAAAAAAA 1018
YS_P._delicatulus_YW10_1____       CAAACAAAGGAA--TGTGCATGAGCGTGTTCAGCAGGTTAGGCAAAAAAA 1018
YS_P._delicatulus_YW68_3____       CAAACAAAGGAA--TGTGCATGAGCGTGTTCAGCAGGTTAGGCAAAAAAA 1019
YS_P._delicatulus_YW72_2____       CAAACAAAGGAA--TGTGCATGAGCGTGTTCAGCAGGTTAGGCAAAAAAA 1020
```

```
YS_P._delicatulus_YW47_2____        CAAACAAAGGAA--TGTGCATGAGCGTGTTCAGCAGGTTAGGCAAAAAAA 1018
YS_P._takakuwai_YW88_1______        CAAACAAAGGAAAATGTGCATGAGCGTGTTCAGCAGGTTAGGCAAAAAAA 1018
YS_P._akitaorum_YW16_1______        CAAACAAAGGAAAATGTGCATGAGCGTGTTCAGCAGGTTAGGCAAAAAAA 1018
YS_P._akitaorum_YW14_2______        CAAACAAAGGAAAATGTGCATGAGCGTGTTCAGCAGGTTAGGCAAAAAAA 1018
YS_P._kawadai_YW12_1______          CAAACAAAGGAA--TGTGCATGAGCGTGTTCAGCAGGTTAGGCAAAAAAA 1016
YS_P._takakuwai_YW13_1______        CAAACAAAGGAA--TGTGCATGAGCGTGTTCAGCAGGTTAGGCAAAAAAA 1139
YS_P._albisomni_YW19_1______        CAAACAAAGGAA--TGTGCATGAGCGTGTTCAGCAGGTTAGGCAAAAAAA 1016
YS_P._albisomni_YW21_1______        CAAACAAAGGAA--TGTGCATGAGCGTGTTCAGCAGGTTAGGCAAAAAAA 1016
YS_P._albisomni_YW23_1______        CAAACAAAGGAA--TGTGCATGAGCGTGTTCAGCAGGTTAGGCAAAAAAA 1014
YS_P._kawadai_YW03_1________        CAAACAAAGGAA--TGTGCATGAGCGTGTTCAGCAGGTTAGGCAAAAAAA 1016
YS_P._takakuwai_YW54_1______        CAAACAAAGGAA--TGTGCATGAGCGTGTTCAGCAGGTTAGGCAAAAAAA 1016
YS_P._takakuwai_YW73_3______        CAAACAAAGGAA--TGTGCATGAGCGTGTTCAGCAGGTTAGGCAAAAAAA 1017
YS_P._acuticollis_YW07_8____        CAAACAAAGGAA--TGTGCATGAGCGTGTTCAGCAGGTTAGGCAAAAAAA 1016
YS_P._albisomni_YW08_1______        CAAACAAAGGAA--TGTGCATGAGCGTGTTCAGCAGGTTAGGCAAAAAAA 1015
YS_P._albisomni_YW09_1______        CAAACAAAGGAA--TGTGCATGAGCGTGTTCAGCAGGTTAGGCAAAAAAA 1016
YS_P._takakuwai_YW38_1______        CAAACAAAGGAA--TGTGCATGAGCGTGTTCAGCAGGTTAGGCAAAAAAA 1016
YS_P._angularis_YW25_8______        CAAACAAAGGAA--TGTGCATGAGCGTGTTCAGCAGGTTAGGCAAAAAAA 1015
                                    ************  ************************************

YS_P._viridicuprus_YW75_3___        ATTTTGCAACATAGTGCGAACTGGACGCGAATGGCATAAATATGAGTGGA 1067
YS_P._viridicuprus_YW76_3___        ATTTTGCAACATAGTGCGAACTGGACGCGAATGGCATAAATATGAGTGGA 1067
YS_P._viridicuprus_YW78_3___        ATTTTGCAACATAGTGCGAACTGGACGCGAATGGCATAAATATGAGTGGA 1067
YS_P._viridicuprus_YW58_1___        ATTTTGCAACATAGTGCGAACTGGACGCGAATGGCATAAATATGAGTGGA 1065
YS_P._viridicuprus_YW04_1___        ATTTTGCAACATAGTGCGAACTGGACGCGAATGGCATAAATATGAGTGGA 1067
YS_P._hongwonpyoi_YW34_8____        ATTTTGCAACATAGTGCGAACTGGACGCGAATGGCATAAATATGAGTGGA 1067
YS_P._hongwonpyoi_YW34_2a___        ATTTTGCAACATAGTGCGAACTGGACGCGAATGGCATAAATATGAGTGGA 1069
YS_P._hongwonpyoi_YW33_8____        ATTTTGCAACATAGTGCGAACTGGACGCGAATGGCATAAATATGAGTGGA 1066
YS_P._hongwonpyoi_YW24_1____        ATTTTGCAACATAGTGCGAACTGGACGCGAATGGCATAAATATGAGTGGA 1068
YS_P._hongwonpyoi_YW05_8____        ATTTTGCAACATAGTGCGAACTGGACGCGAATGGCATAAATATGAGTGGA 1068
YS_P._hongwonpyoi_YW35_8___         ATTTTGCAACATAGTGCGAACTGGACGCGAATGGCATAAATATGAGTGGA 1066
YS_P._hongwonpyoi_YW37_8____        ATTTTGCAACATAGTGCGAACTGGACGCGAATGGCATAAATATGAGTGGA 1068
YS_P._hongwonpyoi_YW36_1____        ATTTTGCAACATAGTGCGAACTGGACGCGAATGGCATAAATATGAGTGGA 1068
YS_P._sue_YW45_1____________        ATTTTGCAACATAGTGCGAACTGGACGCGAATGGCATAAATATGAGTGGA 1066
YS_P._sue_YW80_3____________        ATTTTGCAACATAGTGCGAACTGGACGCGAATGGCATAAATATGAGTGGA 1066
YS_P._takakuwai_YW43_2______        ATTTTGCAACATAGTGCGAACTGGACGCGAATGGCATAAATATGAGTGGA 1053
YS_P._urushiyamai_YW48_1____        ATTTTGCAACATAGTGCGAACTGGACGCGAATGGCATAAATATGAGTGGA 1066
```

```
YS_P._urushiyamai_YW50_1____      ATTTTGCAACATAGTGCGAACTGGACGCGAATGGCATAAATATGAGTGGA 1066
YS_P._urushiyamai_YW49_2____      ATTTTGCAACATAGTGCGAACTGGACGCGAATGGCATAAATATGAGTGGA 1066
YS_P._delicatulus_YW65_2____      ATTT-GCAACATAGTGCGAACTGGACGCGAATGGCATAAATATGAGTGGA 1067
YS_P._delicatulus_YW46_1____      ATTT-GCAACATAGTGCGAACTGGACGCGAATGGCATAAATATGAGTGGA 1067
YS_P._delicatulus_YW10_1____      ATTT-GCAACATAGTGCGAACTGGACGCGAATGGCATAAATATGAGTGGA 1067
YS_P._delicatulus_YW68_3____      ATTT-GCAACATAGTGCGAACTGGACGCGAATGGCATAAATATGAGTGGA 1068
YS_P._delicatulus_YW72_2____      ATTT-GCAACATAGTGCGAACTGGACGCGAATGGCATAAATATGAGTGGA 1069
YS_P._delicatulus_YW47_2____      ATTT-GCAACATAGTGCGAACTGGACGCGAATGGCATAAATATGAGTGGA 1067
YS_P._takakuwai_YW88_1______      A-TTTGCAACATAGTGCGAACTGGACGCGAATGGCATAAATATGAGTGGA 1067
YS_P._akitaorum_YW16_1______      A-TTTGCAACATAGTGCGAACTGGACGCGAATGGCATAAATATGAGTGGA 1067
YS_P._akitaorum_YW14_2______      A-TTTGCAACATAGTGCGAACTGGACGCGAATGGCATAAATATGAGTGGA 1067
YS_P._kawadai_YW12_1______        ATTTTGCAACATAGTGCGAACTGGACGCGAATGGCATAAATATGAGTGGA 1066
YS_P._takakuwai_YW13_1______      ATTTTGCAACATAGTGCGAACTGGACGCGAATGGCATAAATATGAGTGGA 1189
YS_P._albisomni_YW19_1______      ATTTTGCAACATAGTGCGAACTGGACGCGAATGGCATAAATATGAGTGGA 1066
YS_P._albisomni_YW21_1______      ATTTTGCAACATAGTGCGAACTGGACGCGAATGGCATAAATATGAGTGGA 1066
YS_P._albisomni_YW23_1______      ATTTTGCAACATAGTGCGAACTGGACGCGAATGGCATAAATATGAGTGGA 1064
YS_P._kawadai_YW03_1________      ATTTTGCAACATAGTGCGAACTGGACGCGAATGGCATAAATATGAGTGGA 1066
YS_P._takakuwai_YW54_1______      ATTTTGCAACATAGTGCGAACTGGACGCGAATGGCATAAATATG--TGGA 1064
YS_P._takakuwai_YW73_3______      ATTTTGCAACATAGTGCGAACTGGACGCGAATGGCATAAATATGAGTGGA 1067
YS_P._acuticollis_YW07_8____      ATTTTGCAACATAGTGCGAACTGGACGCGAATGGCATAAATATGAGTGGA 1066
YS_P._albisomni_YW08_1______      ATTTTGCAACATAGTGCGAACTGGACGCGAATGGCATAAATATGAGTGGA 1065
YS_P._albisomni_YW09_1______      ATTTTGCAACATAGTGCGAACTGGACGCGAATGGCATAAATATGAGTGGA 1066
YS_P._takakuwai_YW38_1______      AATTTGCAACATAGTGCGAACTGGACGCGAATGGCATAAATATGAGTGGA 1066
YS_P._angularis_YW25_8______      ATTTTGCAACATAGTGCGAACTGGACGCGAATAGCATAAATATGGGTGGA 1065
                                  * ** *************************** ***********  ****

YS_P._viridicuprus_YW75_3___      AGGTAGAATGAGAGTGAAGAGAGAACGGGGATGAAAGAGAAAGGCATATA 1117
YS_P._viridicuprus_YW76_3___      AGGTAGAATGAGAGTGAAGAGAGAACGGGGATGAAAGAGAAAGGCATATA 1117
YS_P._viridicuprus_YW78_3___      AGGTAGAATGAGAGTGAAGAGAGAACGGGGATGAAAGAGAAAGGCATATA 1117
YS_P._viridicuprus_YW58_1___      AGGTAGAATGAGAGTGAAGAGAGAACGGGGATGAAAGAGAAAGGCATATA 1115
YS_P._viridicuprus_YW04_1___      AGGTAGAATGAGAGTGAAGAGAGAACGGGGATGAAAGAGAAAGGCATATA 1117
YS_P._hongwonpyoi_YW34_8____      AGGTAGAATGAGAGTGAAGAGAGAACGGGGATGAAAGAGAAAGGCATATA 1117
YS_P._hongwonpyoi_YW34_2a___      AGGTAGAATGAGAGTGAAGAGAGAACGGGGATGAAAGAGAAAGGCATATA 1119
YS_P._hongwonpyoi_YW33_8____      AGGTAGAATGAGAGTGAAGAGAGAACGGGGATGAAAGAGAAAGGCATATA 1116
YS_P._hongwonpyoi_YW24_1____      AGGTAGAATGAGAGTGAAGAGAGAACGGGGATGAAAGAGAAAGGCATATA 1118
YS_P._hongwonpyoi_YW05_8____      AGGTAGAATGAGAGTGAAGAGAGAACGGGGATGAAAGAGAAAGGCATATA 1118
```

```
YS_P._hongwonpyoi_YW35_8___       AGGTAGAATGAGAGTGAAGAGAGAACGGGGATGAAAGAGAAAGGCATATA 1116
YS_P._hongwonpyoi_YW37_8____      AGGTAGAATGAGAGTGAAGAGAGAACGGGGATGAAAGAGAAAGGCATATA 1118
YS_P._hongwonpyoi_YW36_1____      AGGTAGAATGAGAGTGAAGAGAGAACGGGGATGAAAGAGAAAGGCATATA 1118
YS_P._sue_YW45_1____________      AGGTAGAATGAGAATGAAGAGAGAACGGAGATGAAAGAGAAAGGCATATA 1116
YS_P._sue_YW80_3____________      AGGTAGAATGAGAATGAAGAGAGAACGGAGATGAAAGAGAAAGGCATATA 1116
YS_P._takakuwai_YW43_2______      AGGTAGAATGAGAATGAAGAGAGAACGGAGATGAAAGAGAAAGGCATATA 1103
YS_P._urushiyamai_YW48_1____      AGGTAGAATGAGAATGAAGAGAGAACGGGGATGAAAGAGAAAGGCATATA 1116
YS_P._urushiyamai_YW50_1____      AGGTAGAATGAGAATGAAGAGAGAACGGGGATGAAAGAGAAAGGCATATA 1116
YS_P._urushiyamai_YW49_2____      AGGTAGAATGAGAATGAAGAGAGAACGGGGATGAAAGAGAAAGGCATATA 1116
YS_P._delicatulus_YW65_2____      AGGTAGAATGAGAATGAAGAGAGAACGGGGATGAAAGAGAAAGGCATATA 1117
YS_P._delicatulus_YW46_1____      AGGTAGAATGAGAATGAAGAGAGAACGGGGATGAAAGAGAAAGGCATATA 1117
YS_P._delicatulus_YW10_1____      AGGTAGAATGAGAATGAAGAGAGAACGGGGATGAAAGAGAAAGGCATATA 1117
YS_P._delicatulus_YW68_3____      AGGTAGAATGAGAATGAAGAGAGAACGGGGATGAAAGAGAAAGGCATATA 1118
YS_P._delicatulus_YW72_2____      AGGTAGAATGAGAATGAAGAGAGAACGGGGATGAAAGAGAAAGGCATATA 1119
YS_P._delicatulus_YW47_2____      AGGTAGAATGAGAATGAAGAGAGAACGGGGATGAAAGAGAAAGGCATATA 1117
YS_P._takakuwai_YW88_1______      AGGTAGAATGTGGATGAAGAGAGAACGGGGATGAAAGAGAAAGGCATATA 1117
YS_P._akitaorum_YW16_1______      AGGTAGAATGAGGATGAAGAGAGAACGGGGATGAAAGAGAAAGGCATATA 1117
YS_P._akitaorum_YW14_2______      AGGTAGAATGAGGATGAAGAGAGAACGGGGATGAAAGAGAAAGGCATATA 1117
YS_P._kawadai_YW12_1______        AGGTAGAATGAGAATGAAGAGAGAACGGGGATGAAAGAAAAAGGCATATA 1116
YS_P._takakuwai_YW13_1______      AGGTAGAATGAGAATGAAGAGAGAACGGGGATGAAAGAAAAAGGCATATA 1239
YS_P._albisomni_YW19_1______      AGGTAGAATGAGAATGAAGAGAGAACGGGGATGAAAGAGAAAGGCATATA 1116
YS_P._albisomni_YW21_1______      AGGTAGAATGAGAATGAAGAGAGAACGGGGATGAAAGAGAAAGGCATATA 1116
YS_P._albisomni_YW23_1______      AGGTAGAATGAGAATGAAGAGAGAACGGGGATGAAAGAGAAAGGCATATA 1114
YS_P._kawadai_YW03_1________      AGGTAGAATGAGAATGAAGAGAGAACGGGGATGAAAGAGAAAGGCATATA 1116
YS_P._takakuwai_YW54_1______      AGGTAGAATGAGAATGAAGAGAGAACGGGGATGAAAGAGAAAGGCATATA 1114
YS_P._takakuwai_YW73_3______      AGGTAGAATGAGAATGAAGAGAGAACGGGGATGAAAGAGAAAGGCATATA 1117
YS_P._acuticollis_YW07_8____      AGGTAGAATGAGAATGAAGAGAGAACGGGGATGAAAGAGAAAGGCATATA 1116
YS_P._albisomni_YW08_1______      AGGTAGAATGAGAATGAAGAGAGAACGGGGATGAAAGAGAAAGGCATATA 1115
YS_P._albisomni_YW09_1______      AGGTAGAATGAGAATGAAGAGAGAACGGGGATGAAAGAGAAAGGCATATA 1116
YS_P._takakuwai_YW38_1______      AGGTAGAATGAGAATGAAGAGAGAACGGGGATGAAAGAGAAAGGCATATA 1116
YS_P._angularis_YW25_8______      AGGTAGAATGAGAATGAAGAGAGAACGGGGATGAAAGAAGAAGGCATATA 1115
                                  ********** *  ************** *********  **********

YS_P._viridicuprus_YW75_3___      TATGGAAATGGGGGAGAGATATGGGAGATGAAGATGGATGTTGGCGATTG 1167
YS_P._viridicuprus_YW76_3___      TATGGAAATGGGGGAGAGATATGGGAGATGAAGATGGATGTTGGCGATTG 1167
YS_P._viridicuprus_YW78_3___      TATGGAAATGGGGGAGAGATATGGGAGATGAAGATGGATGTTGGCGATTG 1167
```

YS_P._viridicuprus_YW58_1___	TATGGAAATGGGGGAGAGATATGGGAGATGAAGATGGATGTTGGCGATTG	1165
YS_P._viridicuprus_YW04_1___	TATGGAAATGGGGGAGAGATATGGGAGATGAAGATGGATGTTGGCGATTG	1167
YS_P._hongwonpyoi_YW34_8____	TATGGAAATGGGGGAGAGATATGGGAGATGAAGATGGATGTTGGCGATTG	1167
YS_P._hongwonpyoi_YW34_2a___	TATGGAAATGGGGGAGAGATATGGGAGATGAAGATGGATGTTGGCGATTG	1169
YS_P._hongwonpyoi_YW33_8____	TATGGAAATGGGGGAGAGATATGGGAGATGAAGATGGATGTTGGCGATTG	1166
YS_P._hongwonpyoi_YW24_1____	TATGGAAATGGGGGAGAGATATGGGAGATGAAGATGGATGTTGGCGATTG	1168
YS_P._hongwonpyoi_YW05_8____	TATGGAAATGGGGGAGAGATATGGGAGATGAAGATGGATGTTGGCGATTG	1168
YS_P._hongwonpyoi_YW35_8___	TATGGAAATGGGGGAGAGATATGGGAGATGAAGATGGATGTTGGCGATTG	1166
YS_P._hongwonpyoi_YW37_8____	TATGGAAATGGGGGAGAGATATGGGAGATGAAGATGGATGTTGGCGATTG	1168
YS_P._hongwonpyoi_YW36_1____	TATGGAAATGGGGGAGAGATATGGGAGATGAAGATGGATGTTGGCGATTG	1168
YS_P._sue_YW45_1____________	TATGGAAATGGGGGAGAGATATGGGAGATGAAGATGGATGTTGGCGATTG	1166
YS_P._sue_YW80_3____________	TATGGAAATGGGGGAGAGATATGGGAGATGAAGATGGATGTTGGCGATTG	1166
YS_P._takakuwai_YW43_2______	TATGGAAATGGGGGAGAGATATGGGAGATGAAGATGGATGTTGGCGATTG	1153
YS_P._urushiyamai_YW48_1____	TATGGAAATGGGGGAGAGATATGGGAGATGAAGATGGATGTTGGCGATTG	1166
YS_P._urushiyamai_YW50_1____	TATGGAAATGGGGGAGAGATATGGGAGATGAAGATGGATGTTGGCGATTG	1166
YS_P._urushiyamai_YW49_2____	TATGGAAATGGGGGAGAGATATGGGAGATGAAGATGGATGTTGGCGATTG	1166
YS_P._delicatulus_YW65_2____	TATGGAAATGGGGGAGAGATATGGGAGATGAAGAAGGATGTTGGCGATTG	1167
YS_P._delicatulus_YW46_1____	TATGGAAATGGGGGAGAGATATGGGAGATGAAGAAGGATGTTGGCGATTG	1167
YS_P._delicatulus_YW10_1____	TATGGAAATGGGGGAGAGATATGGGAGATGAAGAAGGATGTTGGCGATTG	1167
YS_P._delicatulus_YW68_3____	TATGGAAATGGGGGAGAGATATGGGAGATGAAGATGGATGTTGGCGATTG	1168
YS_P._delicatulus_YW72_2____	TATGGAAATGGGGGAGAGATATGGGAGATGAAGATGGATGTTGGCGATTG	1169
YS_P._delicatulus_YW47_2____	TATGGAAATGGGGGAGAGATATGGGAGATGAAGATGGATGTTGGCGATTG	1167
YS_P._takakuwai_YW88_1______	TATGGAAATGGGGGAGAGATATGGGAGATGAAGATGGATGTTGGCGATTG	1167
YS_P._akitaorum_YW16_1______	TATGGAAATGGGGGAGAGATATGGGAGATGAAGATGGATGTTGGCGATTG	1167
YS_P._akitaorum_YW14_2______	TATGGAAATGGGGGAGAGATATGGGAGATGAAGATGGATGTTGGCGATTG	1167
YS_P._kawadai_YW12_1______	TATGGAAATGGGGGAGAGATATGGGAGATGAAGATGGATGTTGGCGATTG	1166
YS_P._takakuwai_YW13_1______	TATGGAAATGGGGGAGAGATATGGGAGATGAAGATGGATGTTGGCGATTG	1289
YS_P._albisomni_YW19_1______	TATGGAAATGGGGGAGAGATATGGGAGATGAAGATGGATGTTGGCGATTG	1166
YS_P._albisomni_YW21_1______	TATGGAAATGGGGGAGAGATATGGGAGATGAAGATGGATGTTGGCGATTG	1166
YS_P._albisomni_YW23_1______	TATGGAAATGGGGGAGAGATATGGGAGATGAAGATGGATGTTGGCGATTG	1164
YS_P._kawadai_YW03_1________	TATGGAAATGGGGGAGAGATATGGGAGATGAAGATGGATGTTGGCGATTG	1166
YS_P._takakuwai_YW54_1______	TATGGAAATGGGGGAGAGATATGGGAGATGAAGATGGATGTTGGCGATTG	1164
YS_P._takakuwai_YW73_3______	TATGGAAATGGGGGAGAGATATGGGAGATGAAGATGGATGTTGGCGATTG	1167
YS_P._acuticollis_YW07_8____	TATGGAAATGGGGGAGAGATATGGGAGATGAAGATGGATGTTGGCGATTG	1166
YS_P._albisomni_YW08_1______	TATGGAAATGGGGGAGAGATATGGGAGATGAAGATGGATGTTGGCGATTG	1165
YS_P._albisomni_YW09_1______	TATGGAAATGGGGGAGAGATATGGGAGATGAAGATGGATGTTGGCGATTG	1166

```
YS_P._takakuwai_YW38_1______        TATGGAAATGGGGGAGAGATATGGGAGATGAAGATGGATGTTGGCGATTG 1166
YS_P._angularis_YW25_8______        TATGGAAATGGAGGAGAGATGTGGGAGATGAAGATGGATATTGGCGATTG 1165
                                    *********** ******** ************* **** **********

YS_P._viridicuprus_YW75_3___        GGGTGTGAAGAGAAGAGTATGAACGAGCAATGAGCAGTGGAGAGAGAACA 1217
YS_P._viridicuprus_YW76_3___        GGGTGTGAAGAGAAGAGTATGAACGAGCAATGAGCAGTGGAGAGAGAACA 1217
YS_P._viridicuprus_YW78_3___        GGGTGTGAAGAGAAGAGTATGAACGAGCAATGAGCAGTGGAGAGAGAACA 1217
YS_P._viridicuprus_YW58_1___        GGGTGTGAAGAGAAGAGTATGAACGAGCAATGAGCAGTGGAGAGAGAACA 1215
YS_P._viridicuprus_YW04_1___        GGGTGTGAAGAGAAGAGTATGAACGAGCAATGAGCAGTGGAGAGAGAACA 1217
YS_P._hongwonpyoi_YW34_8____        GGGTGTGAAGAGAAGAGTATGAACGAGCAATGAGCAGTGGAGAGAGAACA 1217
YS_P._hongwonpyoi_YW34_2a___        GGGTGTGAAGAGAAGAGTATGAACGAGCAATGAGCAGTGGAGAGAGAACA 1219
YS_P._hongwonpyoi_YW33_8____        GGGTGTGAAGAGAAGAGTGTGAACGAGCAATGAGCAGTGGAGAGAGAACA 1216
YS_P._hongwonpyoi_YW24_1____        GGGTGTGAAGAGAAGAGTGTGAACGAGCAATGAGCAGTGGAGAGAGAACA 1218
YS_P._hongwonpyoi_YW05_8____        GGGTGTGAAGAGAAGAGTGTGAACGAGCAATGAGCAGTGGAGAGAGAACA 1218
YS_P._hongwonpyoi_YW35_8___         GGGTGTGAAGAGAAGAGTGTGAACGAGCAATGAGCAGTGGAGAGAGAACA 1216
YS_P._hongwonpyoi_YW37_8____        GGGTGTGAAGAGAAGAGTGTGAACGAGCAATGAGCAGTGGAGAGAGAACA 1218
YS_P._hongwonpyoi_YW36_1____        GGGTGTGAAGAGAAGAGTATGAACGAGCAATGAGCAGTGGAGAGAGAACA 1218
YS_P._sue_YW45_1____________        GGGTGTGAAGAGAAGAGTATGAACGAGCAATGAGCAGTGGAGAGAGAACA 1216
YS_P._sue_YW80_3____________        GGGTGTGAAGAGAAGAGTATGAACGAGCAATGAGCAGTGGAGAGAGAACA 1216
YS_P._takakuwai_YW43_2______        GGGTGTGAAGAGAAGAGTATGAACGAGCAATGAGCAGTGGAGAGAGAACA 1203
YS_P._urushiyamai_YW48_1____        GGGTGTGAAGAGAAGAGTATGAACGAGCAATGAGCAGTGGAGAGAGAACA 1216
YS_P._urushiyamai_YW50_1____        GGGTGTGAAGAGAAGAGTATGAACGAGCCATGAGCAGTGGAGAGAGAACA 1216
YS_P._urushiyamai_YW49_2____        GGGTGTGAAGAGAAGAGTATGAACGAGCAATGAGCAGTGGAGAGAGAACA 1216
YS_P._delicatulus_YW65_2____        GGGTGTGAAGAGAAGAGTATGAACGAGCAATGAGCAGTGGAGAGAGAACA 1217
YS_P._delicatulus_YW46_1____        GGGTGTGAAGAGAAGAGTATGGACGAGCAATGAGCAGTGGAGAGAGAACA 1217
YS_P._delicatulus_YW10_1____        GGGTGTGAAGAGAAGAGTATGAACGAGCAATGAGCAGTGGAGAGAGAACA 1217
YS_P._delicatulus_YW68_3____        GGGTGTGAAGAGAAGAGTATGAACGAGCAATGAGCAGTGGAGAGAGAACA 1218
YS_P._delicatulus_YW72_2____        GGGTGTGAAGAGAAGAGTATGAACGAGCAATGAGCAGTGGAGAGAGAACA 1219
YS_P._delicatulus_YW47_2____        GGGTGTGAAGAGAAGAGTATGAACGAGCAATGAGCAGTGGAGAGAGAACA 1217
YS_P._takakuwai_YW88_1______        GGGTGTGAAGAGAACAGTATGAACGAGCAATGAGCAGTGGAGAGAGAACA 1217
YS_P._akitaorum_YW16_1______        GGGTGTGAAGAGAACAGTATGAACGAGCAATGAGCAGTGGAGAGAGAACA 1217
YS_P._akitaorum_YW14_2______        GGGTGTGAAGAGAACAGTATGAACGAGCAATGAGCAGTGGAGAGAGAACA 1217
YS_P._kawadai_YW12_1______          GGGTGTGAAGAGAAGAGTATGAACGAGCAATGAGCAGTGGAGAGAGAACA 1216
YS_P._takakuwai_YW13_1______        GGGTGTGAAGAGAAGAGTATGAACGAGCAATGAGCAGTGGAGAGAGAACA 1339
YS_P._albisomni_YW19_1______        GGGTGTGAAGAGAAGAGTATGAACGAGCAATGAGCAGTGGAGAGAGAACA 1216
YS_P._albisomni_YW21_1______        GGGTGTGAAGAGAAGAGTATGAACGAGCAATGAGCAGTGGAGAGAGAACA 1216
```

```
YS_P._albisomni_YW23_1______        GGGTGTGAAGAGAAGAGTATGAACGAGCAATGAGCAGTGGAGAGAGAACA 1214
YS_P._kawadai_YW03_1________        GGGTGTGAAGAGAAGAGTATGAACGAGCAATGAGCAGTGGAGAGAGAACA 1216
YS_P._takakuwai_YW54_1______        GGGTGTGAAGAGAAGAGTATGAACGAGCAATGAGCAGTGGAGAGAGAACA 1214
YS_P._takakuwai_YW73_3______        GGGTGTGAAGAGAAGAGTATGAACGAGCAATGAGCAGTGGAGAGAGAACA 1217
YS_P._acuticollis_YW07_8____        GGGTGTGAAGAGAAGAGTATGAACGAGCAATGAGCAGTGGAGAGAGAACA 1216
YS_P._albisomni_YW08_1______        GGGTGTGAAGAGAAGAGTATGAACGAGCAATGAGCAGTGGAGAGAGAACA 1215
YS_P._albisomni_YW09_1______        GGGTGTGAAGAGAAGAGTATGAACGAGCAATGAGCAGTGGAGAGAGAACA 1216
YS_P._takakuwai_YW38_1______        GGGTGTGAAGAGAAGAGTATGAACGAGCAATGAGCAGTGGAGAGAGAACA 1216
YS_P._angularis_YW25_8______        GGGTGTGAAGAGAAGAGTATGGACGAGCAATGAGCAGTGGGGAGAAAACA 1215
                                    ************* *** ** ****** *********** **** ****

YS_P._viridicuprus_YW75_3___        GGGTAGCGGAAACAGAGAGAGAATGAG----AGAAATATGAGAAGCTGCG 1263
YS_P._viridicuprus_YW76_3___        GGGTAGCGGAAACAGAGAGAGAATGAG----AGAAATATGAGAAGCTGCG 1263
YS_P._viridicuprus_YW78_3___        GGGTAGCGGAAACAGAGAGAGAATGAG----AGAAATATGAGAAGCTGCG 1263
YS_P._viridicuprus_YW58_1___        GGGTAGCGGAAACAGAGAGAGAATGAG----AGAAATATGAGAAGCTGCG 1261
YS_P._viridicuprus_YW04_1___        GGGTAGCGGAAACAGAGAGAGAATGAG----AGAAATATGAGAAGCTGCG 1263
YS_P._hongwonpyoi_YW34_8____        GGGTAGCGGAAACAGAGAGAGAATGAG----AGAAATATGAGAAGCTGCG 1263
YS_P._hongwonpyoi_YW34_2a___        GGGTAGCGGAAACAGAGAGAGAATGAG----AGAAATATGAGAAGCTGCG 1265
YS_P._hongwonpyoi_YW33_8____        GGGTAGCGGAAACAGAGAGAGAATGAG----AGAAATATGAGAAGCTGCG 1262
YS_P._hongwonpyoi_YW24_1____        GGGTAGCGGAAACAGAGAGAGAATGAG----AGAAATATGAGAAGCTGCG 1264
YS_P._hongwonpyoi_YW05_8____        GGGTAGCGGAAACAGAGAGAGAATGAG----AGAAATATGAGAAGCTGCG 1264
YS_P._hongwonpyoi_YW35_8___         GGGTAGCGGAAACAGAGAGAGAATGAG----AGAAATATGAGAAGCTGCG 1262
YS_P._hongwonpyoi_YW37_8____        GGGTAGCGGAAACAGAGAGAGAATGAG----AGAAATATGAGAAGCTGCG 1264
YS_P._hongwonpyoi_YW36_1____        GGGTAGCGGAAACAGAGAGAGGGTGAG----AGAAATATGAGAAGTTGCG 1264
YS_P._sue_YW45_1____________        GGGTAGCGGAAACAGAGAGAGAATGAG----AGAAATATGAGAAGTTGCG 1262
YS_P._sue_YW80_3____________        GGGTAGCGGAAACAGAGAGAGAATGAG----AGAAATATGAGAAGTTGCG 1262
YS_P._takakuwai_YW43_2______        GGGTAGCGGAAATAGAGAGAGAATGAG----AGAAATATGAGAAGTTGCG 1249
YS_P._urushiyamai_YW48_1____        GGGTAGCGGAAACAGAGAGAGAATGAG----AGAAATATGAGAAGTTGCG 1262
YS_P._urushiyamai_YW50_1____        GGGTAGCGGAAACAGAGAGAGAATGAG----AGAAATATGAGAAGTTGCG 1262
YS_P._urushiyamai_YW49_2____        GGGTAGCGGAAACAGAGAGAGAATGAG----AGAAATATGAGAAGTTGCG 1262
YS_P._delicatulus_YW65_2____        GGGTAGCGGAAACAGAGAGAGAATGAG----AGAAATATGAGAAGTTGCG 1263
YS_P._delicatulus_YW46_1____        GGGTAGCGGAAACAGAGAGAGAATGAG----AGAAATATGAGAAGTTGCG 1263
YS_P._delicatulus_YW10_1____        GGGTAGCGGAAACAGAGAGAGAATGAG----AGAAATATGAGAAGTTGCG 1263
YS_P._delicatulus_YW68_3____        GGGTAGCGGAAACAGAGAGAGAATGAG----AGAAATATGAGAAGTTGCG 1264
YS_P._delicatulus_YW72_2____        GGGTAGCGGAAACAGAGAGAGAATGAG----AGAAATATGAGAAGTTGCG 1265
YS_P._delicatulus_YW47_2____        GGGTAGCGGAAACAGAGAGAGAATGAG----AGAAATATGAGAAGTTGCG 1263
```

```
YS_P._takakuwai_YW88_1______       GGGTAGCGGAAACAGAGAGAGAATGAG----AGAAATATGAGAAGTTGCG 1263
YS_P._akitaorum_YW16_1______       GGGTAGCGGAAACAGAGAGAGAATGAG----AGAAATATGAGAAGTTGCG 1263
YS_P._akitaorum_YW14_2______       GGGTAGCGGAAACAGAGAGAGAATGAG----AGAAATATGAGAAGTTGCG 1263
YS_P._kawadai_YW12_1______         GGGTAGCGGAAACAGAGAGAGAATGAG----AGAAATATGAGAAGTTGCG 1262
YS_P._takakuwai_YW13_1______       GGGTAGCGGAAACAGAGAGAGAATGAG----AGAAATATGAGAAGTTGCG 1385
YS_P._albisomni_YW19_1______       GGGTAGCGGAAACAGAGAGAGAATGAG----AGAAATATGAGAAGTTGCG 1262
YS_P._albisomni_YW21_1______       GGGTAGCGGAAACAGAGAGAGAATGAG----AGAAATATGAGAAGTTGCG 1262
YS_P._albisomni_YW23_1______       GGGTAGCGGAAACAGAGAGAGAATGAG----AGAAATATGAGAAGTTGCG 1260
YS_P._kawadai_YW03_1________       GGGTAGCGGAAACAGAGAGAGAATGAG----AGAAATATGAGAAGTTGCG 1262
YS_P._takakuwai_YW54_1______       GGGTAGCGGAAACAGAGAGAGAATGAG----AGAAATATGAGAAGTTGCG 1260
YS_P._takakuwai_YW73_3______       GGGTAGCGGAAACAGAGAGAGAATGAG----AGAAATATGAGAAGTTGCG 1263
YS_P._acuticollis_YW07_8____       GGGTAGCGGAAACAGAGAGAGAATGAG----AGAAATATGAGAAGTTGCG 1262
YS_P._albisomni_YW08_1______       GGGTAGCGGAAACAGAGAGAGAATGAG----AGAAATATGAGAAGTTGCG 1261
YS_P._albisomni_YW09_1______       GGGTAGCGGAAACAGAGAGAGAATGAG----AGAAATATGAGAAGTTGCG 1262
YS_P._takakuwai_YW38_1______       GGGTAGCGGAAACAGAGAGAGAATGAG----AGAAATATGAGAAGTTGCG 1262
YS_P._angularis_YW25_8______       GGGTAGCGGAAAGAGAGAGAGAGTGAGTGAGAGAAATATGGGAAGTTGCG 1265
                                   ************ ********  ****    ********* **** ****

YS_P._viridicuprus_YW75_3___       ATACAATA-----GAGTGACAGAAGATCAAGCTTGAGGTGTATAGGATCA 1308
YS_P._viridicuprus_YW76_3___       ATACAATA-----GAGTGACAGAAGATCAAGCTTGAGGTGTATAGGATCA 1308
YS_P._viridicuprus_YW78_3___       ATACAATA-----GAGTGACAGAAGATCAAGCTTGAGGTGTATAGGATCA 1308
YS_P._viridicuprus_YW58_1___       ATACAATA-----GAGTGACAGAAGATCAAGCTTGAGGTGTATAGGATCA 1306
YS_P._viridicuprus_YW04_1___       ATACAATA-----GAGTGACAGAAGATCAAGCTTGAGGTGTATAGGATCA 1308
YS_P._hongwonpyoi_YW34_8____       ATACAATA-----GAGTGACAGAAGATCAAGCTTGAGGTGTATAGGATCA 1308
YS_P._hongwonpyoi_YW34_2a___       ATACAATA-----GAGTGACAGAAGATCAAGCTTGAGGTGTATAGGATCA 1310
YS_P._hongwonpyoi_YW33_8____       ATACAATA-----GAGTGACAGAAGATCAAGCTTGAGGTGTATAGGATCA 1307
YS_P._hongwonpyoi_YW24_1____       ATACAATA-----GAGTGACAGAAGATCAAGCTTGAGGTGTATAGGATCA 1309
YS_P._hongwonpyoi_YW05_8____       ATACAATA-----GAGTGACAGAAGATCAAGCTTGAGGTGTATAGGATCA 1309
YS_P._hongwonpyoi_YW35_8___        ATACAATA-----GAGTGACAGAAGATCAAGCTTGAGGTGTATAGGATCA 1307
YS_P._hongwonpyoi_YW37_8____       ATACAATA-----GAGTGACAGAAGATCAAGCTTGAGGTGTATAGGATCA 1309
YS_P._hongwonpyoi_YW36_1____       ACACAATA-----GAGTGACAGAAGATCAAGCTTGAGGTGTATAGGATCA 1309
YS_P._sue_YW45_1____________       ATACAATA-----GAGTGACAGAAGATCAAGCTTGAGGTGTAGAGGATCA 1307
YS_P._sue_YW80_3____________       ATACAATA-----GAGTGACAGAAGATCAAGCTTGAGGTGTAGAGGATCA 1307
YS_P._takakuwai_YW43_2______       ATACAATA-----GAGTGACAGAAGATCAAGCTTGAGGTGTAGAGGATCA 1294
YS_P._urushiyamai_YW48_1____       ATACAATA-----GAGTGACAGAAGATCAAGCTTGAGGTGTAGAGGATCA 1307
YS_P._urushiyamai_YW50_1____       ATACAATA-----GAGTGACAGAAGATCAAGCTTGAGGTGTAGAGGATCA 1307
```

```
YS_P._urushiyamai_YW49_2____        ATACAATA-----GAGTGACAGAAGATCAAGCTTGAGGTGTAGAGGATCA 1307
YS_P._delicatulus_YW65_2____        ATACAATACAATAGAGTGACAGAAGATCAAGCTTGAGGTGTAGAGGATCA 1313
YS_P._delicatulus_YW46_1____        ATACAATACAATAGAGTGACAGAAGATCAAGCTTGAGGTGTAGAGGATCA 1313
YS_P._delicatulus_YW10_1____        ATACAATACAATAGAGTGACAGAAGATCAAGCTTGAGGTGTAGAGGATCA 1313
YS_P._delicatulus_YW68_3____        ATACAATA-----GAGTGACAGAAGATCAAGCTTGAGGTGTAGAGGATCA 1309
YS_P._delicatulus_YW72_2____        ATACAATA-----GAGTGACAGAAGATCAAGCTTGAGGTGTAGAGGATCA 1310
YS_P._delicatulus_YW47_2____        ATACAATA-----GAGTGACAGAAGATCAAGCTTGAGGTGTAGAGGATCA 1308
YS_P._takakuwai_YW88_1______        ATACAATA-----GAGTGACAGAAGATCAAGCTTGAGGTGTAGAGGATCA 1308
YS_P._akitaorum_YW16_1______        ATACAATA-----GAGTGACAGAAGATCAAGCTTGAGGTGTAGAGGATCA 1308
YS_P._akitaorum_YW14_2______        ATACAATA-----GAGTGACAGAAGATCAAGCTTGAGGTGTAGAGGATCA 1308
YS_P._kawadai_YW12_1______          ATACAATA-----GAGTGACAGAAGATCAAGCTTGAGGTGTAGAGGATCA 1307
YS_P._takakuwai_YW13_1______        ATACAATA-----GAGTGACAGAAGATCAAGCTTGAGGTGTAGAGGATCA 1430
YS_P._albisomni_YW19_1______        ATACAATA-----GAGTGACAGAAGATCAAGCTTGAGGTGTAGAGGATCA 1307
YS_P._albisomni_YW21_1______        ATACAATA-----GAGTGACAGAAGATCAAGCTTGAGGTGTAGAGGATCA 1307
YS_P._albisomni_YW23_1______        ATACAATA-----GAGTGACAGAAGATCAAGCTTGAGGTGTAGAGGATCA 1305
YS_P._kawadai_YW03_1________        ATACAATA-----GAGTGACAGAAGATCAAGCTTGAGGTGTAGAGGATCA 1307
YS_P._takakuwai_YW54_1______        ATACAATA-----GAGTGACAGAAGATCAAGCTTGAGGTGTAGAGGATCA 1305
YS_P._takakuwai_YW73_3______        ATACAATA-----GAGTGACAGAAGATCAAGCTTGAGGTGTAGAGGATCA 1308
YS_P._acuticollis_YW07_8____        ATACAATA-----GAGTGACAGAAGATCAAGCTTGAGGTGTAGAGGATCA 1307
YS_P._albisomni_YW08_1______        ATACAATA-----GAGTGACAGAAGATCAAGCTTGAGGTGTAGAGGATCA 1306
YS_P._albisomni_YW09_1______        ATACAATA-----GAGTGACAGAAGATCAAGCTTGAGGTGTAGAGGATCA 1307
YS_P._takakuwai_YW38_1______        ATACAATA-----GAGTGACAGAAGATCAAGCTTGAGGTGTAGAGGATCA 1307
YS_P._angularis_YW25_8______        ATAGAATC-----GAGTGACAGAAGATCAAGCTTGAGGTGTATAGCAGCA 1310
                                    * * ***      ***************************** ** * **

YS_P._viridicuprus_YW75_3___        GCA----------------------------AGTTAGCAGCAAGAAGTGA 1330
YS_P._viridicuprus_YW76_3___        GCA----------------------------AGTTAGCAGCAAGAAGTGA 1330
YS_P._viridicuprus_YW78_3___        GCA----------------------------AGTTAGCAGCAAGAAGTGA 1330
YS_P._viridicuprus_YW58_1___        GCA----------------------------AGTTAGCAGCAAGAAGTGA 1328
YS_P._viridicuprus_YW04_1___        GCA----------------------------AGTTAGCAGCAAGAAGTGA 1330
YS_P._hongwonpyoi_YW34_8____        GCA----------------------------AGTTAGCAGCAAGAAGTGA 1330
YS_P._hongwonpyoi_YW34_2a___        GCA----------------------------AGTTAGCAGCAAGAAGTGA 1332
YS_P._hongwonpyoi_YW33_8____        GCA----------------------------AGTTAGCAGCAAGAAGTGA 1329
YS_P._hongwonpyoi_YW24_1____        GCA----------------------------AGTTAGCAGCAAGAAGTGA 1331
YS_P._hongwonpyoi_YW05_8____        GCA----------------------------AGTTAGCAGCAAGAAGTGA 1331
YS_P._hongwonpyoi_YW35_8___         GCA----------------------------AGTTAGCAGCAAGAAGTGA 1329
```

```
YS_P._hongwonpyoi_YW37_8____      GCA----------------------------AGTTAGCAGCAAGAAGTGA 1331
YS_P._hongwonpyoi_YW36_1____      GCA----------------------------AGTTAGCAGCAAGAAGTGA 1331
YS_P._sue_YW45_1____________      GCA----------------------------AGTTAGCAACAAGAAGTGA 1329
YS_P._sue_YW80_3____________      GCA----------------------------AGTTAGCAACAAGAAGTGA 1329
YS_P._takakuwai_YW43_2______      GCA----------------------------AGTTAGCAACAAGAAGTGA 1316
YS_P._urushiyamai_YW48_1____      GCA----------------------------AGTTAGCAACAAGAAGTGA 1329
YS_P._urushiyamai_YW50_1____      GCA----------------------------AGTTAGCAACAAGAAGTGA 1329
YS_P._urushiyamai_YW49_2____      GCA----------------------------AGTTAGCAACAAGAAGTGA 1329
YS_P._delicatulus_YW65_2____      GCA----------------------------AGTTAGCGACAAGAAGTGA 1335
YS_P._delicatulus_YW46_1____      GCA----------------------------AGTTAGCGACAAGAAGTGA 1335
YS_P._delicatulus_YW10_1____      GCA----------------------------AGTTAGCGACAAGAAGTGA 1335
YS_P._delicatulus_YW68_3____      GCA----------------------------AGTTAGCGACAAGAAGTGA 1331
YS_P._delicatulus_YW72_2____      GCA----------------------------AGTTAGCGACAAGAAGTGA 1332
YS_P._delicatulus_YW47_2____      GCA----------------------------AGTTAGCGACAAGAAGTGA 1330
YS_P._takakuwai_YW88_1______      GCA----------------------------AGTTAGCAACAAGAAGTGA 1330
YS_P._akitaorum_YW16_1______      GCA----------------------------AGTTAGCAACAAGAAGTGA 1330
YS_P._akitaorum_YW14_2______      GCA----------------------------AGTTAGCAACAAGAAGTGA 1330
YS_P._kawadai_YW12_1______        GCA----------------------------AGTTAGCAACAAGAAGTGA 1329
YS_P._takakuwai_YW13_1______      GCA----------------------------AGTTAGCAACAAGAAGTGA 1452
YS_P._albisomni_YW19_1______      GCA----------------------------AGTTAGCAACAAGAAGTGA 1329
YS_P._albisomni_YW21_1______      GCA----------------------------AGATAGCAACAAGAAGTGA 1329
YS_P._albisomni_YW23_1______      GCA----------------------------AGTTAGCAACAAGAAGTGA 1327
YS_P._kawadai_YW03_1________      GCA----------------------------AGTTAGCAACAAGAAGTGA 1329
YS_P._takakuwai_YW54_1______      GCA----------------------------AGTTAGCAACAAGAAGTGA 1327
YS_P._takakuwai_YW73_3______      GCA----------------------------AGTTAGCAACAAGAAGTGA 1330
YS_P._acuticollis_YW07_8____      GCA----------------------------AGTTAGCAACAAGAAGTGA 1329
YS_P._albisomni_YW08_1______      GCA----------------------------AGTTAGCAACAAGAAGTGA 1328
YS_P._albisomni_YW09_1______      GCA----------------------------AGTTAGCAACAAGAAGTGA 1329
YS_P._takakuwai_YW38_1______      GCA----------------------------AGTTAGCAACAAGAAGTGA 1329
YS_P._angularis_YW25_8______      GCATGTTAGCAGCAGCAAGTTAGCAGCAGCAAGTTAGCAGCACGAAGTGA 1360
                                  ***                            ** ****  ** *******

YS_P._viridicuprus_YW75_3___      AAGGTAGAGATGGGATAGTATTGGGTGGCATAGGAAGAGGTCGTGTGAGG 1380
YS_P._viridicuprus_YW76_3___      AAGGTAGAGATGGGATAGTATTGGGTGGCATAGGAAGAGGTCGTGTGAGG 1380
YS_P._viridicuprus_YW78_3___      AAGGTAGAGATGGGATAGTATTGGGTGGCATAGGAAGAGGTCGTGTGAGG 1380
YS_P._viridicuprus_YW58_1___      AAGGTAGAGATGGGATAGTATTGGGTGGCATAGGAAGAGGTCGTGTGAGG 1378
```

YS_P._viridicuprus_YW04_1___ AAGGTAGAGATGGGATAGTATTGGGTGGCATAGGAAGAGGTCGTGTGAGG 1380

YS_P._hongwonpyoi_YW34_8____ AAGGTAGAGATGGGATAGTATTGGGTGGCATAGGAAGAGGTCGTGTGAGG 1380

YS_P._hongwonpyoi_YW34_2a___ AAGGTAGAGATGGGATAGTATTGGGTGGCATAGGAAGAGGTCGTGTGAGG 1382

YS_P._hongwonpyoi_YW33_8____ AAGGTAGAGATGGGATAGTATTGGGAGGCATAGGAAGAGGTCGTGTGAGG 1379

YS_P._hongwonpyoi_YW24_1____ AAGGTAGAGATGGGATAGTATTGGGAGGCATAGGAAGAGGTCGTGTGAGG 1381

YS_P._hongwonpyoi_YW05_8____ AAGGTAGAGATGGGATAGTATTGGGAGGCATAGGAAGAGGTCGTGTGAGG 1381

YS_P._hongwonpyoi_YW35_8___ AAGGTAGAGATGGGATAGTATTGGGTGGCATAGGAAGAGGTCGTGTGAGG 1379

YS_P._hongwonpyoi_YW37_8____ AAGGTAGAGATGGGATAGTATTGGGTGGCATAGGAAGAGGTCGTGTGAGG 1381

YS_P._hongwonpyoi_YW36_1____ AAGGTAGAGATGGGATAGTATTGGGTGGCATAGGAAGAGGTCGTGTGAGG 1381

YS_P._sue_YW45_1____________ AAGGTAGAGATGGGATAGTATTGGGTGGCATAGGAAGAGGTCGTGTGCGG 1379

YS_P._sue_YW80_3____________ AAGGTAGAGATGGGATAGTATTGGGTGGCATAGGAAGAGGTCGTGTGCGG 1379

YS_P._takakuwai_YW43_2______ AAGGTAGAGATGGGATAGTATTGGGTGGCATAGGAAGAGGTCGTGTGCGG 1366

YS_P._urushiyamai_YW48_1____ AAGGTAGAGATGGGATAGTATTGGGTGGCATAGGAAGAGGTCGTGTGCGG 1379

YS_P._urushiyamai_YW50_1____ AAGGTAGAGATGGGATAGTATTGGGTGGCATAGGAAGAGGTCGTGTGCGG 1379

YS_P._urushiyamai_YW49_2____ AAGGTAGAGATGGGATAGTATTGGGTGGCATAGGAAGAGGTCGTGTGCGG 1379

YS_P._delicatulus_YW65_2____ AAGGTAGAGATGGGATAGTATTGGGTGGCATAGGAAGAGGTCGTGTGCGG 1385

YS_P._delicatulus_YW46_1____ AAGGTAGAGATGGGATAGTATTGGGTGGCATAGGAAGAGGTCGTGTGCGG 1385

YS_P._delicatulus_YW10_1____ AAGGTAGAGATGGGATAGTATTGGGTGGCATAGGAAGAGGTCGTGTGCGG 1385

YS_P._delicatulus_YW68_3____ AAGGTAGAGATGGGATAGTATTGGGTGGCATAGGAAGAGGTCGTGTGCGG 1381

YS_P._delicatulus_YW72_2____ AAGGTAGAGATGGGATAGTATTGGGTGGCATAGGAAGAGGTCGTGTGCGG 1382

YS_P._delicatulus_YW47_2____ AAGGTAGAGATGGGATAGTATTGGGTGGCATAGGAAGAGGTCGTGTGCGG 1380

YS_P._takakuwai_YW88_1______ AAGGTAGAGATGGGATAGTATTGGGTG-CATAGGAAGAGGTCGTGTGCGG 1379

YS_P._akitaorum_YW16_1______ AAGGTAGAGATGGGATAGTATTGGGTGGCATAGGAAGAGGTCGTGTGCGG 1380

YS_P._akitaorum_YW14_2______ AAGGTAGAGATGGGATAGTATTGGGTGGCATAGGAAGAGGTCGTGTGCGG 1380

YS_P._kawadai_YW12_1______ AAGGTAGAGATGGGATAGTATTGGGTGGCATAGGAAGAGGTCGTGTGCGG 1379

YS_P._takakuwai_YW13_1______ AAGGTAGAGATGGGATAGTATTGGGTGGCATAGGAAGAGGTCGTGTGCGG 1502

YS_P._albisomni_YW19_1______ AAGGTAGAGATGGGATAGTATTGGGTGGCATAGGAAGAGGTCGTGTGCGG 1379

YS_P._albisomni_YW21_1______ AAGGTAGAGATGGGATAGTATTGGGTGGCATAGGAAGAGGTCGTGTGCGG 1379

YS_P._albisomni_YW23_1______ AAGGTAGAGATGGGATAGTATTGGGTGGCATAGGAAGAGGTCGTGTGCGG 1377

YS_P._kawadai_YW03_1________ AAGGTAGAGATGGGATAGTATTGGGTGGCATAGGAAGAGGTCGTGTGCGG 1379

YS_P._takakuwai_YW54_1______ AAGGTAGAGATGGGATAGTATTGGGTGGCATAGGAAGAGGTCGTGTGCGG 1377

YS_P._takakuwai_YW73_3______ AAGGTAGAGATGGGATAGTATTGGGTGGCATAGGAAGAGGTCGTGTGCGG 1380

YS_P._acuticollis_YW07_8____ AAGGTAGAGATGGGATAGTATTGGGTGGCATAGGAAGAGGTCGTGTGCGG 1379

YS_P._albisomni_YW08_1______ AAGGTAGAGATGGGATAGTATTGGGTGGCATAGGAAGAGGTCGTGTGCGG 1378

YS_P._albisomni_YW09_1______ AAGGTAGAGATGGGATAGTATTGGGTGGCATAGGAAGAGGTCGTGTGCGG 1379

YS_P._takakuwai_YW38_1______ AAGGTAGAGATGGGATAGTATTGGGTGGCATAGGAAGAGGTCGTGTGCGG 1379

```
YS_P._angularis_YW25_8______        AAGGTAGAGATGGGATAGTATTGGGTGGGATAGGAAGAGGTCGTGTGCGG 1410
                                    ************************* *  ****************** **

YS_P._viridicuprus_YW75_3___        -TAGCCAAAATATTTTGGACGGAAAAAGACCGAACTGGCGAAATGCGACC 1429
YS_P._viridicuprus_YW76_3___        -TAGCCAAAATATTTTGGACGGAAAAAGACCGAACTGGCGAAATGCGACC 1429
YS_P._viridicuprus_YW78_3___        -TAGCCAAAATATTTTGGACGGAAAAAGACCGAACTGGCGAAATGCGACC 1429
YS_P._viridicuprus_YW58_1___        -TAGCCAAAATATTTTGGACGGAAAAAGACCGAACTGGCGAAATGCGACC 1427
YS_P._viridicuprus_YW04_1___        -TAGCCAAAATATTCTGGACGGAAAAAGACCGAACTGGCGAAATGCGACC 1429
YS_P._hongwonpyoi_YW34_8____        -TAGCCAAAATATTTTGGACGGAAAAAGACCGAACTGGCGAAATGCGACC 1429
YS_P._hongwonpyoi_YW34_2a___        -TAGCCAAAATATTTTGGACGGAAAAAGACCGAACTGGCGAAATGCGACC 1431
YS_P._hongwonpyoi_YW33_8____        -TAGCCAAAATATTTTGGACGGAAAAAGACCGAACTGGCGAAATGCGACC 1428
YS_P._hongwonpyoi_YW24_1____        -TAGCCAAAATATTTTGGACGGAAAAAGACCGAACTGGCGAAATGCGACC 1430
YS_P._hongwonpyoi_YW05_8____        -TAGCCAAAATATTTTGGACGGAAAAAGACCGAACTGGCGAAATGCGACC 1430
YS_P._hongwonpyoi_YW35_8___         -TAGCCAAAATATTTTGGACGGAAAAAGACCGAACTGGCGAAATGCGACC 1428
YS_P._hongwonpyoi_YW37_8____        -TAGCCAAAATATTTTGGACGGAAAAAGACCGAACTGGCGAAATGCGACC 1430
YS_P._hongwonpyoi_YW36_1____        -TAGCCAAAATATTTTGGACGGAAAAAGACCGAACTGGCGAAATGCGACC 1430
YS_P._sue_YW45_1____________        -TAGCCAAAATATTTTGGACGGAAAAAGACCGAACTGGCGAAATGCGACC 1428
YS_P._sue_YW80_3____________        -TAGCCAAAATATTTTGGACGGAAAAAGACCGAACTGGCGAAATGCGACC 1428
YS_P._takakuwai_YW43_2______        -TAGCCAAAATATTTTGGACGGAAAAAGACCGAACTGGCGAAATGCGACC 1415
YS_P._urushiyamai_YW48_1____        -TAGCCAAAATATTTTGGACGGAAAAAGACCGAACTGGCGAAATGCGACC 1428
YS_P._urushiyamai_YW50_1____        -TAGCCAAAATATTTTGGACGGAAAAAGACCGAACTGGCGAAATGCGACC 1428
YS_P._urushiyamai_YW49_2____        -TAGCCAAAATATTTTGGACGGAAAAAGACCGAACTGGCGAAATGCGACC 1428
YS_P._delicatulus_YW65_2____        -TAGCCAAAATATTTTGGACGGAAAAAGACCGAACTGGCGAAATGCGACC 1434
YS_P._delicatulus_YW46_1____        -TAGCCAAAATATTTTGGACGGAAAAAGACCGAACTGGCGAAATGCGACC 1434
YS_P._delicatulus_YW10_1____        -TAGCCAAAATATTTTGGACGGAAAAAGACCGAACTGGCGAAATGCGACC 1434
YS_P._delicatulus_YW68_3____        GTAGCCAAAATATTTTGGACGGAAAAAGACCGAACTGGCGAAATGCGACC 1431
YS_P._delicatulus_YW72_2____        GTAGCCAAAATATTTTGGACGGAAAAAGACCGAACTGGCGAAATGCGACC 1432
YS_P._delicatulus_YW47_2____        -TAGCCAAAATATTTTGGACGGAAAAAGACCGAACTGGCGAAATGCGACC 1429
YS_P._takakuwai_YW88_1______        -TAGCCAAAATATTTTGGACGGAAAAAGACCGAACTGGCGAAATGCGACC 1428
YS_P._akitaorum_YW16_1______        -TAGCCAAAATATTTTGGACGGAAAAAGACCGAACTGGCGAAATGCGACC 1429
YS_P._akitaorum_YW14_2______        -TAGCCAAAATATTTTGGACGGAAAAAGACCGAACTGGCGAAATGCGACC 1429
YS_P._kawadai_YW12_1______          -TAGCCAAAATATTTTGGACGGAAAAAGACCGAACTGGCGAAATGCGACC 1428
YS_P._takakuwai_YW13_1______        -TAGCCAAAATATTTTGGACGGAAAAAGACCGAACTGGCGAAATGCGACC 1551
YS_P._albisomni_YW19_1______        -TAGCCAAAATATTTTGGACGGAAAAAGACCGAACTGGCGAAATGCGACC 1428
YS_P._albisomni_YW21_1______        -TAGCCAAAATATTTTGGACGGAAAAAGACCGAACTGGCGAAATGCGACC 1428
YS_P._albisomni_YW23_1______        -TAGCCAAAATATTTTGGACGGAAAAAGACCGAACTGGCGAAATGCGACC 1426
```

```
YS_P._kawadai_YW03_1________        -TAGCCAAAATATTTTGGACGGAAAAAGACCGAACTGGCGAAATGCGACC 1428
YS_P._takakuwai_YW54_1______        -TAGCCAAAATATTTTGGACGGAAAAAGACCGAACTGGCGAAATGCGACC 1426
YS_P._takakuwai_YW73_3______        -TAGCCAAAATATTTTGGACGGAAAAAGACCGAACTGGCGAAATGCGACC 1429
YS_P._acuticollis_YW07_8____        -TAGCCAAAATATTTTGGACGGAAAAAGACCGAACTGGCGAAATGCGACC 1428
YS_P._albisomni_YW08_1______        -TAGCCAAAATATTTTGGACGGAAAAAGACCGAACTGGCGAAATGCGACC 1427
YS_P._albisomni_YW09_1______        -TAGCCAAAATATTTTGGACGGAAAAAGACCGAACTGGCGAAATGCGACC 1428
YS_P._takakuwai_YW38_1______        -TAGCCAAAATATTTTGGACGGAAAAAGACCGAACTGGCGAAATGCGACC 1428
YS_P._angularis_YW25_8______        -TAGCCAAAATATTTTGGACGGAAAAAGACCGAACTGGCGAAATGCGACC 1459
                                     ************* ***********************************

YS_P._viridicuprus_YW75_3___        -AAGTCATTGCATAAAAAAA--GACTAAACTTGAATTGATAATGTGATGA 1476
YS_P._viridicuprus_YW76_3___        -AAGTCATTGCATAAAAAAA--GACTAAACTTGAATTGATAATGTGATGA 1476
YS_P._viridicuprus_YW78_3___        -AAGTCATTGCATAAAAAAAAAGACTAAACTTGAATTGATAATGTGATGA 1478
YS_P._viridicuprus_YW58_1___        -AAGTCATTGCATAAAAAAA--GACTAAACTTGAATTGATAATGTGATGA 1474
YS_P._viridicuprus_YW04_1___        -AAGTCATTGCATAAAAAAAA-GACTAAACTTGAATTGATAATGTGATGA 1477
YS_P._hongwonpyoi_YW34_8____        -AAGTCATTGCATAAAAAAAAAGACTAAACTTGAATTGATAATGTGATGA 1478
YS_P._hongwonpyoi_YW34_2a___        -AAGTCATTGCATAAAAAAAAAGACTAAACTTGAATTGATAATGTGATGA 1480
YS_P._hongwonpyoi_YW33_8____        -AAGTCATTGCATAAAAAAA--GACTAAACTTGAATTGATAATGTGATGA 1475
YS_P._hongwonpyoi_YW24_1____        -AAGTCATTGCATAAAAAAA--GACTAAACTTGAATTGATAATGTGATGA 1477
YS_P._hongwonpyoi_YW05_8____        -AAGTCATTGCATAAAAAAA--GACTAAACTTGAATTGATAATGTGATGA 1477
YS_P._hongwonpyoi_YW35_8___         -AAGTCATTGCATAAAAAAAAAGACTAAACTTGAATTGATAATGTGATGA 1477
YS_P._hongwonpyoi_YW37_8____        -AAGTCATTGCATAAAAAAAAAGACTAAACTTGAATTGATAATGTGATGA 1479
YS_P._hongwonpyoi_YW36_1____        -AAGTCATTGCATAAAAAAAAAGACTAAACTTGAATTGATAATGTGATGA 1479
YS_P._sue_YW45_1____________        -AAGTCATTGCATAAAAA----GACTAAACTTGAATTGATAATGTGATGA 1473
YS_P._sue_YW80_3____________        -AAGTCATTGCATAAAAA----GACTAAACTTGAATTGATAATGTGATGA 1473
YS_P._takakuwai_YW43_2______        -AAGTCATTGCATAAAAAAA--GACTAAACTTGAATTGATAATGTGATGA 1462
YS_P._urushiyamai_YW48_1____        -AAGTCATTGCATTAAAAAA--GACTAAACTTGAATTGATAATGTGATGA 1475
YS_P._urushiyamai_YW50_1____        -AAGTCATTGCATTAAAAAA--GACTAAACTTGAATTGATAATGTGATGA 1475
YS_P._urushiyamai_YW49_2____        -AAGTCATTGCATTAAAA----GACTAAACTTGAATTGATAATGTGATGA 1473
YS_P._delicatulus_YW65_2____        CAAGTCATTGCATAAAAAAA--GACTAAACTTGAATTGATAATGTGATGA 1482
YS_P._delicatulus_YW46_1____        CAAGTCATTGCATAAAAAAA--GACTAAACTTGAATTGATAATGTGATGA 1482
YS_P._delicatulus_YW10_1____        CAAGTCATTGCATAAAAAAA--GACTAAACTTGAATTGATAATGTGATGA 1482
YS_P._delicatulus_YW68_3____        CAAGTCATTGCATAAAAAAA--GACTAAACTTGAATTGATAATGTGATGA 1479
YS_P._delicatulus_YW72_2____        CAAGTCATTGCATAAAAAAA--GACTAAACTTGAATTGATAATGTGATGA 1480
YS_P._delicatulus_YW47_2____        CAAGTCATTGCATAAAAAAA--GACTAAACTTGAATTGATAATGAGATGA 1477
YS_P._takakuwai_YW88_1______        -AAGTCATTGCATAAAAAAA--GACTAAACTTGAATTGATAATGTGATGA 1475
```

```
YS_P._akitaorum_YW16_1______       -AAGTCATTGCATAAAAAAA--GACTAAACTTGAATTGATAATGTGATGA 1476
YS_P._akitaorum_YW14_2______       -AAGTCATTGCATAAAAAAA--GACTAAACTTGAATTGATAATGTGATGA 1476
YS_P._kawadai_YW12_1______         -AAGTCATTGCATAAAAA----GACTAAACTTGAATTGATAATGTGATGA 1473
YS_P._takakuwai_YW13_1______       -AAGTCATTGCATAAAAA----GACTAAACTTGAATTGATAATGTGATGA 1596
YS_P._albisomni_YW19_1______       -AAGTCATTGCATAAAAA----GACTAAACTTGAATTGATAATGTGATGA 1473
YS_P._albisomni_YW21_1______       -AAGTCATTGCATAAAAA----GACTAAACTTGAATTGATAATGTGATGA 1473
YS_P._albisomni_YW23_1______       -AAGTCATTGCATAAAAA----GACTAAACTTGAATTGATAATGTGATGA 1471
YS_P._kawadai_YW03_1________       -AAGTCATTGCATAAAAA----GACTAAACTTGAATTGATAATGTGATGA 1473
YS_P._takakuwai_YW54_1______       -AAGTCATTGCATAAAAAAA--GACTAAACTTGAATTGATAATGTGATGA 1473
YS_P._takakuwai_YW73_3______       -AAGTCATTGCATAAAAAAA--GACTAAACTTGAATTGATAATGTGATGA 1476
YS_P._acuticollis_YW07_8____       -AAGTCATTGCATAAAAAAA--GACTAAACTTGAATTAATAATGTGATGA 1475
YS_P._albisomni_YW08_1______       -AAGTCATTGCATAAAAAAA--GACTAAACTTGAATTGATAATGTGATGA 1474
YS_P._albisomni_YW09_1______       -AAGTCATTGCATAAAAAAA--GACTAAACTTGAATTGATAATGTGATGA 1475
YS_P._takakuwai_YW38_1______       -AAGTCATTGCATAAAAAAA--GACTAAACTTGAATTGATAATGTGATGA 1475
YS_P._angularis_YW25_8______       -AAGTCATTGCATAAAAAAAAAGACTAAACTTCAATTGATAATGTGATGA 1508
                                    ************ ****    ********** **** ****** *****

YS_P._viridicuprus_YW75_3___       GTAACCCGCACAAATCTTTACGTCTCAAAAAACTTTTTCCCTTGAGAAAG 1526
YS_P._viridicuprus_YW76_3___       GTAACCCGCACAAATCTTTACGTCTCAAAAAACTTTTTCCCTTGAGAAAG 1526
YS_P._viridicuprus_YW78_3___       GTAACCCGCACAAATCTTTACGTCTCAAAAAACTTTTTCCCTTGAGAAAG 1528
YS_P._viridicuprus_YW58_1___       GTAACCCGCACAAATCTTTACGTCTCAAAAAACTTTTTCCCTTGAGAAAG 1524
YS_P._viridicuprus_YW04_1___       GTAACCCGCACAAATCTTTACGTCTCAAAAAACTTTTTCCCTTGAGAAAG 1527
YS_P._hongwonpyoi_YW34_8____       GTAACCCGCACAAATCTTTACGTCTCAAAAAACTTTTTCCCTTGAGAAAG 1528
YS_P._hongwonpyoi_YW34_2a___       GTAACCCGCACAAATCTTTACGTCTCAAAAAACTTTTTCCCTTGAGAAAG 1530
YS_P._hongwonpyoi_YW33_8____       GTAACCCGCACAAATCTTTACGTCTCAAAAAACTTTTTCCCTTGAGAAAG 1525
YS_P._hongwonpyoi_YW24_1____       GTAACCCGCACAAATCTTTACGTCTCAAAAAACTTTTTCCCTTGAGAAAG 1527
YS_P._hongwonpyoi_YW05_8____       GTAACCCGCACAAATCTTTACGTCTCAAAAAACTTTTTCCCTTGAGAAAG 1527
YS_P._hongwonpyoi_YW35_8___        GTAACCCGCACAAATCTTTACGTCTCAAAAAACTTTTTCCCTTGAGAAAG 1527
YS_P._hongwonpyoi_YW37_8____       GTAACCCGCACAAATCTTTACGTCTCAAAAAACTTTTTCCCTTGAGAAAG 1529
YS_P._hongwonpyoi_YW36_1____       GTAACCCGCACAAATCTTTACGTCTCAAAAAACTTTTTCCCTTGAGAAAG 1529
YS_P._sue_YW45_1____________       GTAACCCGCACAAATCTTTACGTCTCAAAAAACTTTTTCCCTTGAGAAAG 1523
YS_P._sue_YW80_3____________       GTAACCCGCACAAATCTTTACGTCTCAAAAAACTTTTTCCCTTGAGAAAG 1523
YS_P._takakuwai_YW43_2______       GTAACCCGCACAAATCTTTACGTCTCAAAAAACTTTTTCCCTTGAGAAAG 1512
YS_P._urushiyamai_YW48_1____       GTAACCCGCACAAATCTTTACGTCTCAAAAAACTTTTTCCCTTGAGAAAG 1525
YS_P._urushiyamai_YW50_1____       GTAACCCGCACAAATCTTTACGTCTCAAAAAACTTTTTCCCTTGAGAAAG 1525
YS_P._urushiyamai_YW49_2____       GTAACCCGCACAAATCTTTACGTCTCAAAAAACTTTTTCCCTTGAGAAAG 1523
```

```
YS_P._delicatulus_YW65_2____      GTAACCCGCACAAATCTTTACGTCTCAAAAAACTTTTTCCCTTGAGAAAG 1532
YS_P._delicatulus_YW46_1____      GTAACCCGCACAAATCTTTACGTCTCAAAAAACTTTTTCCCTTGAGAAAG 1532
YS_P._delicatulus_YW10_1____      GTAACCCGCACAAATCTTTACGTCTCAAAAAACTTTTTCCCTTGAGAAAG 1532
YS_P._delicatulus_YW68_3____      GTAACCCGCGCAAATCTTTACGTCTCAAAAAACTTTTTCCCTTGAGAAAG 1529
YS_P._delicatulus_YW72_2____      GTAACCCGCGCAAATCTTTACGTCTCAAAAAACTTTTTCCCTTGAGAAAG 1530
YS_P._delicatulus_YW47_2____      GTAACCCGCGCAAATCTTTACGTCTCAAAAAACTTTTTCCCTTGAGAAAG 1527
YS_P._takakuwai_YW88_1______      GTAACCCGCACAAATCTTTACGTCTCAAAAAACTTTTTCCCTTGCGAAAG 1525
YS_P._akitaorum_YW16_1______      GTAACCCGCACAAATCTTTACGTCTCAAAAAACTTTTTCCCTTGCGAAAG 1526
YS_P._akitaorum_YW14_2______      GTAACCCGCACAAATCTTTACGTCTCAAAAAACTTTTTCCCTTGCGAAAG 1526
YS_P._kawadai_YW12_1______        GTAACCCGCACAAATCTTTACGTCTCAAAAAACTTTTTCCCTTGAGAAAG 1523
YS_P._takakuwai_YW13_1______      GTAACCCGCACAAATCTTTACGTCTCAAAAAACTTTTTCCCTTGAGAAAG 1646
YS_P._albisomni_YW19_1______      GTAACCCGCACAAATCTTTACGTCTCAAAAAACTTTTTCCCTTGAGAAAG 1523
YS_P._albisomni_YW21_1______      GTAACCCGCACAAATCTTTACGTCTCAAAAAACTTTTTCCCTTGAGAAAG 1523
YS_P._albisomni_YW23_1______      GTAACCCGCACAAATCTTTACGTCTCAAAAAACTTTTTCCCTTGAGAAAG 1521
YS_P._kawadai_YW03_1________      GTAACCCGCACAAATCTTTACGTCTCAAAAAACTTTTTCCCTTGAGAAAG 1523
YS_P._takakuwai_YW54_1______      GTAACCCGCACAAATCTTTACGTCTCAAAAAACTTTTTCCCTTGAGAAAG 1523
YS_P._takakuwai_YW73_3______      GTAACCCGCACAAATCTTTACGTCTCAAAAAACTTTTTCCCTTGAGAAAG 1526
YS_P._acuticollis_YW07_8____      GTAACCCGCACAAATCTTTACGTCTCAAAAAACTTTTTCCCTTGAGAAAG 1525
YS_P._albisomni_YW08_1______      GTAACCCGCACAAATCTTTACGTCTCAAAAAACTTTTTCCCTTGAGAAAG 1524
YS_P._albisomni_YW09_1______      GTAACCCGCACAAATCTTTACGTCTCAAAAAACTTTTTCCCTTGAGAAAG 1525
YS_P._takakuwai_YW38_1______      GTAACCCGCACAAATCTTTACGTCTCAAAAAACTTTTTCCCTTGAGAAAG 1525
YS_P._angularis_YW25_8______      GTAACCCGCACAAATCTTTACGTCTCAAAAAACTTTTTCCCTTGAGAAAG 1558
                                  ********* ********************************** *****

YS_P._viridicuprus_YW75_3___      TTGAGAAGAAATATTTTTAGTTTGTGCAAGGGAATAGAGGTGGTGGATCA 1576
YS_P._viridicuprus_YW76_3___      TTGAGAAGAAATATTTTTAGTTTGTGCAAGGGAATAGAGGTGGTGGATCA 1576
YS_P._viridicuprus_YW78_3___      TTGAGAAGAAATATTTTTAGTTTGTGCAAGGGAATAGAGGTGGTGGATCA 1578
YS_P._viridicuprus_YW58_1___      TTGAGAAGAAATATTTTTAGTTTGTGCAAGGGAATAGAGGTGGTGGATCA 1574
YS_P._viridicuprus_YW04_1___      TTGAGAAGAAATATTTTTAGTTTGTGCAAGGGAATAGAGGTGGTGGATCA 1577
YS_P._hongwonpyoi_YW34_8____      TTGAGAAGAAATATTTTTAGTTTGTGCAAGGGAATAGAGGTGGTGGATCA 1578
YS_P._hongwonpyoi_YW34_2a___      TTGAGAAGAAATATTTTTAGTTTGTGCAAGGGAATAGAGGTGGTGGATCA 1580
YS_P._hongwonpyoi_YW33_8____      TTGAGAAGAAATATTTTTAGTTTGTGCAAGGGAATAGAGGTGGTGGATCA 1575
YS_P._hongwonpyoi_YW24_1____      TTGAGAAGAAATATTTTTAGTTTGTGCAAGGGAATAGAGGTGGTGGATCA 1577
YS_P._hongwonpyoi_YW05_8____      TTGAGAAGAAATATTTTTAGTTTGTGCAAGGGAATAGAGGTGGTGGATCA 1577
YS_P._hongwonpyoi_YW35_8___       TTGAGAAGAAATATTTTTAGTTTGTGCAAGGGAATAGAGGTGGTGGATCA 1577
YS_P._hongwonpyoi_YW37_8____      TTGAGAAGAAATATTTTTAGTTTGTGCAAGGGAATAGAGGTGGTGGATCA 1579
```

```
YS_P._hongwonpyoi_YW36_1____      TTGAGAAGAAATATTTTTAGTTTGTGCAAGGGAATAGAGGTGGTGGATCA 1579
YS_P._sue_YW45_1____________      TTGAAAAGAAATATTTTTAGTTTGCGCAAGGGAAAAGAGGTGGTGGATCA 1573
YS_P._sue_YW80_3____________      TTGAAAAGAAATATTTTTAGTTTGCGCAAGGGAAAAGAGGTGGTGGATCA 1573
YS_P._takakuwai_YW43_2______      TTGAAAAGAAATATTTTTAGTTTGCGCAAGGGAAAAGAGGTGGTGGATCA 1562
YS_P._urushiyamai_YW48_1____      TTGAAAAGAAATATTTTTAGTTTGCGCAAGGGAAAAGAGGTGGTGGATCA 1575
YS_P._urushiyamai_YW50_1____      TTGAAAAGAAATATTTTTAGTTTGCGCAAGGGAAAAGAGGTGGTGGATCA 1575
YS_P._urushiyamai_YW49_2____      TTGAAAAGAAATATTTTTAGTTTGCGCAAGGGAAAAGAGGTGGTGGATCA 1573
YS_P._delicatulus_YW65_2____      TTGAAAAGAAATATTTTTAGTTTGTGCAAGGGAAAAGAGGTGGTGGATCA 1582
YS_P._delicatulus_YW46_1____      TTGAAAAGAAATATTTTTAGTTTGTGCAAGGGAAAAGAGGTGGTGGATCA 1582
YS_P._delicatulus_YW10_1____      TTGAAAAGAAATATTTTTAGTTTGTGCAAGGGAAAAGAGGTGGTGGATCA 1582
YS_P._delicatulus_YW68_3____      TTGAAAAGAAATATTTTTAGTTTGTGCAAGGGAAAAGAGGTGGTGGATCA 1579
YS_P._delicatulus_YW72_2____      TTGAAAAGAAATATTTTTAGTTTGTGCAAGGGAAAAGAGGTGGTGGATCA 1580
YS_P._delicatulus_YW47_2____      TTGAAAAGAAATATTTTTAGTTTGTGCAAGGGAAAAGAGGTGGTGGATCA 1577
YS_P._takakuwai_YW88_1______      TTGAGAAGAAATAATTTTAGTTTGTGCAAGGGAAAAGAGGTGGTGGATCA 1575
YS_P._akitaorum_YW16_1______      TTGAGAAGAAATAATTTTAGTTTGTGCAAGGGAAAAGAGGTGGTTGATCA 1576
YS_P._akitaorum_YW14_2______      TTGAGAAGAAATAATTTTAGTTTGTGCAAGGGAAAAGAGGTGGTGGATCA 1576
YS_P._kawadai_YW12_1______        TTGAGAAGAAATATTTTTAGTTTGTGCAAGGGAAAAGAGGTGGTGGATCA 1573
YS_P._takakuwai_YW13_1______      TTGAGAAGAAATATTTTTAGTTTGTGCAAGGGAAAAGAGGTGGTGGATCA 1696
YS_P._albisomni_YW19_1______      TTGAGAAGAAATATTTTTAGTTTGTGCAAGGGAAAAGAGGTGGTGGATCA 1573
YS_P._albisomni_YW21_1______      TTGAGAAGAAATATTTTTAGTTTGTGCAAGGAAAAAGAGGTGGTGGATCA 1573
YS_P._albisomni_YW23_1______      TTGAGAAGAAATATTTTTAGTTTGTGCAAGGGAAAAGAGGTGGTGGATCA 1571
YS_P._kawadai_YW03_1________      TTGAGAAGAAATATTTTTAGTTTGTGCAAGGGAAAAGAGGTGGTGGATCA 1573
YS_P._takakuwai_YW54_1______      TTGAGAAGAAATATTTTTAGTTTGTGCAAGGGAAAAGAGGTGGTGGATCA 1573
YS_P._takakuwai_YW73_3______      TTGAGAAGAAATATTTTTAGTTTGTGCAAGGGAAAAGAGGTGGTGGATCA 1576
YS_P._acuticollis_YW07_8____      TTGAGAAGAAATATTTTTAGTTTGTGCAAGGGAAAAGAGGTGGTGGATCA 1575
YS_P._albisomni_YW08_1______      TTGAGAAGAAATATTTTTAGTTTGTGCAAGGGAAAAGAGGTGGTGGATCA 1574
YS_P._albisomni_YW09_1______      TTGAGAAGAAATATTTTTAGTTTGTGCAAGGGAAAAGAGGTGGTGGATCA 1575
YS_P._takakuwai_YW38_1______      CTGAGAAGAAATATTTTTAGTTTGAGCAAGGGAAAAGAGGTGGTGGATCA 1575
YS_P._angularis_YW25_8______      TTGAGAAGAAATATTTTTAGTTTGCGCAAAGGAATAGAGGTGGTGGATCA 1608
                                   *** ******** ********** **** * ** ********* *****

YS_P._viridicuprus_YW75_3___      GAAAACATCAGAGATATGCTGGGAGGAATTACATGCGAAAGCATGAAACA 1626
YS_P._viridicuprus_YW76_3___      GAAAACATCAGAGATATGCTGGGAGGAATTACATGCGAAAGCATGAAACA 1626
YS_P._viridicuprus_YW78_3___      GAAAACATCAGAGATATGCTGGGAGGAATTACATGCGAAAGCATGAAACA 1628
YS_P._viridicuprus_YW58_1___      GAAAACATCAGAGATATGCTGGGAGGAATTACATGCGAAAGCATGAAACA 1624
YS_P._viridicuprus_YW04_1___      GAAAACATCAGAGATATGCTGGGAGGAATTACATGCGAAAGCATGAAACA 1627
```

```
YS_P._hongwonpyoi_YW34_8____      GAAAACATCAGAGATATGCTGGGAGGAATTACATGCGAAAGCATGAAACA 1628
YS_P._hongwonpyoi_YW34_2a___      GAAAACATCAGAGATATGCTGGGAGGAATTACATGCGAAAGCATGAAACA 1630
YS_P._hongwonpyoi_YW33_8____      GAAAACATCAGAGATATGCTGGGAGGAATTACATGCGAAAGCATGAAACA 1625
YS_P._hongwonpyoi_YW24_1____      GAAAACATCAGAGATATGCTGGGAGGAATTACATGCGAAAGCATGAAACA 1627
YS_P._hongwonpyoi_YW05_8____      GAAAACATCAGAGATATGCTGGGAGGAATTACATGCGAAAGCATGAAACA 1627
YS_P._hongwonpyoi_YW35_8___       GAAAACATCAGAGATATGCTGGGAGGAATTACATGCGAAAGCATGAAACA 1627
YS_P._hongwonpyoi_YW37_8____      GAAAACATCAGAGATATGCTGGGAGGAATTACATGCGAAAGCATGAAACA 1629
YS_P._hongwonpyoi_YW36_1____      GAAAACATCAGAGATATGCTGGGAGGAATTACATGCGAAAGCATGAAACA 1629
YS_P._sue_YW45_1____________      GAAAACATCAGAGATATGCTGGGAGGAATTACATGCGAAAGCATGAAACA 1623
YS_P._sue_YW80_3____________      GAAAACATCAGAGATATGCTGGGAGGAATTACATGCGAAAGCATGAAACA 1623
YS_P._takakuwai_YW43_2______      GAAAACATCAGAGATATGCTGGGAGGAATTACATGCGAAAGCATGAAACA 1612
YS_P._urushiyamai_YW48_1____      GAAAACATCAGAGATACGCTGGGAGGAATTACATGCGAAAGCATGAAACA 1625
YS_P._urushiyamai_YW50_1____      GAAAACATCAGAGATACGCTGGGAGGAATTACATGCGAAAGCATGAAACA 1625
YS_P._urushiyamai_YW49_2____      GAAAACATCAGAGATACGCTGGGAGGAATTACATGCGAAAGCATGAAACA 1623
YS_P._delicatulus_YW65_2____      GAAAACATCAGAGATATGCTGGGAGGAATTACATGCGAAAGCATGAAACA 1632
YS_P._delicatulus_YW46_1____      GAAAACATCAGAGATATGCTGGGAGGAATTACATGCGAAAGCATGAAACA 1632
YS_P._delicatulus_YW10_1____      GAAAACATCAGAGATATGCTGGGAGGAATTACATGCGAAAGCATGAAACA 1632
YS_P._delicatulus_YW68_3____      GAAAACATCAGAGATATGCTGGGAGGAATTACATGCGAAAGCATGAAACA 1629
YS_P._delicatulus_YW72_2____      GAAAACATCAGAGATATGCTGGGAGGAATTACATGCGAAAGCATGAAACA 1630
YS_P._delicatulus_YW47_2____      GAAAACATCAGAGATATGCTGGGAGGAATTACATGCGAAAGCATGAAACA 1627
YS_P._takakuwai_YW88_1______      GAAAACATCAGAGATATGCTGGGAGGAATTACATGCGAAAGCATGAAACA 1625
YS_P._akitaorum_YW16_1______      GAAAACATCAGAGATATGCTGGGAGGAATTACATGCGAAAGCATGAAACA 1626
YS_P._akitaorum_YW14_2______      GAAAACATCAGAGATATGCTGGGAGGAATTACATGCGAAAGCATGAAACA 1626
YS_P._kawadai_YW12_1_______       GAAAACATCAGAGATATGCTGGGAGGAATTACATGCGAAAGCATGAAACA 1623
YS_P._takakuwai_YW13_1______      GAAAACATCAGAGATATGCTGGGAGGAATTACATGCGAAAGCATGAAACA 1746
YS_P._albisomni_YW19_1______      GAAAACATCAGAGATATGCTGGGAGGAATTACATGCGAAAGCATGAAACA 1623
YS_P._albisomni_YW21_1______      GAAAACATCAGAGATATGCTGGGAGGAATTACATGCGAAAGCATGAAACA 1623
YS_P._albisomni_YW23_1______      GAAAACATCAGAGATATGCTGGGAGGAATTACATGCGAAAGCATGAAACA 1621
YS_P._kawadai_YW03_1________      GAAAACATCAGAGATATGCTGGGAGGAATTACATGCGAAAGCATGAAACA 1623
YS_P._takakuwai_YW54_1______      GAAAACATCAGAGATATGCTGGGAGGAATTACATGCGAAAGCATGAAACA 1623
YS_P._takakuwai_YW73_3______      GAAAACATCAGAGATATGCTGGGAGGAATTACATGCGAAAGCATGAAACA 1626
YS_P._acuticollis_YW07_8____      GAAAACATCAGAGATATGCTGGGAGGAATTACATGCGAAAGCATGAAACA 1625
YS_P._albisomni_YW08_1______      GAAAACATCAGAGATATGCTGGGAGGAATTACATGCGAAAGCATGAAACA 1624
YS_P._albisomni_YW09_1______      GAAAACATCAGAGATATGCTGGGAGGAATTACATGCGAAAGCATGAAACA 1625
YS_P._takakuwai_YW38_1______      GAAAACATCAGAGATATGCTGGGAGGAATTACATGCGAAAGCATGAAACA 1625
YS_P._angularis_YW25_8______      GAAAACATCAGAGATATGCTGGGAGGAATTACATGCGAAAGCATGAAACA 1658
```

```
                                    **************** *********************************

YS_P._viridicuprus_YW75_3___        GTTGTAAAACAATTCAGATAATGAGTTGTAGTAACATTGTTTTTATGGCC 1676
YS_P._viridicuprus_YW76_3___        GTTGTAAAACAATTCAGATAATGAGTTGTAGTAACATTGTTTTTATGGCC 1676
YS_P._viridicuprus_YW78_3___        GTTGTAAAACAATTCAGATAATGAGTTGTAGTAACATTGTTTTTATGGCC 1678
YS_P._viridicuprus_YW58_1___        GTTGTAAAACAATTCAGATAATGAGTTGTAGTAACATTGTTTTTATGGCC 1674
YS_P._viridicuprus_YW04_1___        GTTGTAAAACAATTCAGATAATGAGTTGTAGTAACATTGTTTTTATGGCC 1677
YS_P._hongwonpyoi_YW34_8____        GTTGTAAAACAATTCAGATAATGAGTTGTAGTAACATTGTTTTTATGGCC 1678
YS_P._hongwonpyoi_YW34_2a___        GTTGTAAAACAATTCAGATAATGAGTTGTAGTAACATTGTTTTTATGGCC 1680
YS_P._hongwonpyoi_YW33_8____        GTTGTAAAACAATTCAGATAATGAGTTGTAGTAACATTGTTTTTATGGCC 1675
YS_P._hongwonpyoi_YW24_1____        GTTGTAAAACAATTCAGATAATGAGTTGTAGTAACATTGTTTTTATGGCC 1677
YS_P._hongwonpyoi_YW05_8____        GTTGTAAAACAATTCAGATAATGAGTTGTAGTAACATTGTTTTTATGGCC 1677
YS_P._hongwonpyoi_YW35_8___         GTTGTAAAACAATTCAGATAATGAGTTGTAGTAACATTGTTTTTATGGCC 1677
YS_P._hongwonpyoi_YW37_8____        GTTGTAAAACAATTCAGATAATGAGTTGTAGTAACATTGTTTTTATGGCC 1679
YS_P._hongwonpyoi_YW36_1____        GTTGTAAAACAATTCAGATAATGAGTTGTAGTAACATTGTTTTTATGGCC 1679
YS_P._sue_YW45_1____________        GTTGTAAAACAATTCAGATAATGAGTTGTAGTAACATTGTTTATATGGCC 1673
YS_P._sue_YW80_3____________        GTTGTAAAACAATTCAGATAATGAGTTGTAGTAACATTGTTTATATGGCC 1673
YS_P._takakuwai_YW43_2______        GTTGTAAAACAATTCAGATAATGAGTTGTAGTAACATTGTTTATATGGCC 1662
YS_P._urushiyamai_YW48_1____        GTTGTAAAACAATTCAGATAATGAGTTGTAGTAACATTGTTTATATGGCC 1675
YS_P._urushiyamai_YW50_1____        GTTGTAAAACAATTCAGATAATGAGTTGTAGTAACATTGTTTATATGGCC 1675
YS_P._urushiyamai_YW49_2____        GTTGTAAAACAATTCAGATAATGAGTTGTAGTAACATTGTTTATATGGCC 1673
YS_P._delicatulus_YW65_2____        GTTGTAAAACAATTCAGATAATGAGTTGTAGTAACATTGTTTATATGGCC 1682
YS_P._delicatulus_YW46_1____        GTTGTAAAACAATTCAGATAATGAGTTGTAGTAACATTGTTTATATGGCC 1682
YS_P._delicatulus_YW10_1____        GTTGTAAAACAATTCAGATAATGAGTTGTAGTAACATTGTTTATATGGCC 1682
YS_P._delicatulus_YW68_3____        GTTGTAAAACAATTCAGATAATGAGTTGTAGTAACATTGTTTATATGGCC 1679
YS_P._delicatulus_YW72_2____        GTTGTAAAACAATTCAGATAATGAGTTGTAGTAACATTGTTTATATGGCC 1680
YS_P._delicatulus_YW47_2____        GTTGTAAAACAATTCAGATAATGAGTTGTAGTAACATTGTTTATATGGCC 1677
YS_P._takakuwai_YW88_1______        GTTGTAAAACAATTCAGATAATGAGTTGTAGTAACATTGTTTATATGGCC 1675
YS_P._akitaorum_YW16_1______        GTTGTAAAACAATTCAGATAATGAGTTGTAGTAACATTGTTTATATGGCC 1676
YS_P._akitaorum_YW14_2______        GTTGTAAAACAATTCAGATAATGAGTTGTAGTAACATTGTTTATATGGCC 1676
YS_P._kawadai_YW12_1______          GTTGTAAAACAATTCAGATAATGAGTTGTAGTAACATTGTTTATATGGCC 1673
YS_P._takakuwai_YW13_1______        GTTGTAAAACAATTCAGATAATGAGTTGTAGTAACATTGTTTATATGGCC 1796
YS_P._albisomni_YW19_1______        GTTGTAAAACAATTCAGATAATGAGTTGTAGTAACATTGTTTATATGGCC 1673
YS_P._albisomni_YW21_1______        GTTGTAAAACAATTCAGATAATGAGTTGTAGTAACATTGTTTATATGGCC 1673
YS_P._albisomni_YW23_1______        GTTGTAAAACAATTCAGATAATGAGTTGTAGTAACATTGTTTATATGGCC 1671
YS_P._kawadai_YW03_1________        GTTGTAAAACAATTCAGATAATGAGTTGTAGTAACATTGTTTATATGGCC 1673
```

```
YS_P._takakuwai_YW54_1______       GTTGTAAAACAATTCAGATAATGAGTTGTAGTAACATTGTTTATATGGCC 1673
YS_P._takakuwai_YW73_3______       GTTGTAAAACAATTCAGATAATGAGTTGTAGTAACATTGTTTATATGGCC 1676
YS_P._acuticollis_YW07_8____       GTTGTAAAACAATTCAGATAATGAGTTGTAGTAACATTGTTTATATGGCC 1675
YS_P._albisomni_YW08_1______       GTTGTAAAACAATTCAGATAATGAGTTGTAGTAACATTGTTTATATGGCC 1674
YS_P._albisomni_YW09_1______       GTTGTAAAACAATTCAGATAATGAGTTGTAGTAACATTGTTTATATGGCC 1675
YS_P._takakuwai_YW38_1______       GTTGTAAAACAATTCAGATAATGAGTTGTAGTAACATTGTTTATATGGCC 1675
YS_P._angularis_YW25_8______       GTTGTAAAACAATTCAGATAATGAGTTGTAGTAACATTGTTTTTATGGCC 1708
                                   ****************************************** *******

YS_P._viridicuprus_YW75_3___       TCGTTGTTAAGTGGAGAGAGTTTAATGGTGAACGTAAGTTTGGCATTGCT 1726
YS_P._viridicuprus_YW76_3___       TCGTTGTTAAGTGGAGAGAGTTTAATGGTGAACGTAAGTTTGGCATTGCT 1726
YS_P._viridicuprus_YW78_3___       TCGTTGTTAAGTGGAGAGAGTTTAATGGTGAACGTAAGTTTGGCATTGCT 1728
YS_P._viridicuprus_YW58_1___       TCGTTGTTAAGTGGAGAGAGTTTAATGGTGAACGTAAGTTTGGCATTGCT 1724
YS_P._viridicuprus_YW04_1___       TCGTTGTTAAGTGGAGAGAGTTTAATGGTGAACGTAAGTTTGGCATTGCT 1727
YS_P._hongwonpyoi_YW34_8____       TCGTTGTTAAGTGGAGAGAGTTTAATGGTGAACGTAAGTTTGGCATTGCT 1728
YS_P._hongwonpyoi_YW34_2a___       TCGTTGTTAAGTGGAGAGAGTTTAATGGTGAACGTAAGTTTGGCATTGCT 1730
YS_P._hongwonpyoi_YW33_8____       TCGTTGTTAAGTGGAGAGAGTTTAATGGTGAACGTAAGTTTGGCATTGCT 1725
YS_P._hongwonpyoi_YW24_1____       TCGTTGTTAAGTGGAGAGAGTTTAATGGTGAACGTAAGTTTGGCATTGCT 1727
YS_P._hongwonpyoi_YW05_8____       TCGTTGTTAAGTGGAGAGAGTTTAATGGTGAACGTAAGTTTGGCATTGCT 1727
YS_P._hongwonpyoi_YW35_8___        TCGTTGTTAAGTGGAGAGAGTTTAATGGTGAACGTAAGTTTGGCATTGCT 1727
YS_P._hongwonpyoi_YW37_8____       TCGTTGTTAAGTGGAGAGAGTTTAATGGTGAACGTAAGTTTGGCATTGCT 1729
YS_P._hongwonpyoi_YW36_1____       TCGTTGTTAAGTGGAGAGAGTTTAATGGTGAACGTAAGTTTGGCATTGCT 1729
YS_P._sue_YW45_1____________       TCGTTGTTAAGTGGAGAGAGTTTAATGGTGAACGTAAGTTTGGCATTGCT 1723
YS_P._sue_YW80_3____________       TCGTTGTTAAGTGGAGAGAGTTTAATGGTGAACGTAAGTTTGGCATTGCT 1723
YS_P._takakuwai_YW43_2______       TCGTTGTTAAGTGGAGAGAGTTTAATGGTGAACGTAAGTTTGGCATTGCT 1712
YS_P._urushiyamai_YW48_1____       TCGTTGTTAAGTGGAGAGAGTTTAATGGTGAACGTAAGTTTGGCATTGCT 1725
YS_P._urushiyamai_YW50_1____       TCGTTGTTAAGTGGAGAGAGTTTAATGGTGAACGTAAGTTTGGCATTGCT 1725
YS_P._urushiyamai_YW49_2____       TCGTTGTTAAGTGGAGAGAGTTTAATGGTGAACGTAAGTTTGGCATTGCT 1723
YS_P._delicatulus_YW65_2____       TTGTTGTTAAGTGGAGAGAGTTTAATGGTGAACGTAAGTTTGGCATTGCT 1732
YS_P._delicatulus_YW46_1____       TTGTTGTTAAGTGGAGAGAGTTTAATGGTGAACGTAAGTTTGGCATTGCT 1732
YS_P._delicatulus_YW10_1____       TTGTTGTTAAGTGGAGAGAGTTTAATGGTGAACGTAAGTTTGGCATTGCT 1732
YS_P._delicatulus_YW68_3____       TCGTTGTTAAGTGGAGAGAGTTTAATGGTGAACGTAAGTTTGGCATTGCT 1729
YS_P._delicatulus_YW72_2____       TCGTTGTTAAGTGGAGAGAGTTTAATGGTGAACGTAAGTTTGGCATTGCT 1730
YS_P._delicatulus_YW47_2____       TCGTTGTTAAGTGGAGAGAGTTTAATGGTGAACGTAAGTTTGGCATTGCT 1727
YS_P._takakuwai_YW88_1______       TCGTTGTTAAGTGGAGAGAGTTTAATGGTGAACGTAAGTTTGGCATTGCT 1725
YS_P._akitaorum_YW16_1______       TCGTTGTTAAGTGGAGAGAGTTTAATGGTGAACGTAAGTTTGGCATTGCT 1726
```

```
YS_P._akitaorum_YW14_2______        TCGTTGTTAAGTGGAGAGAGTTTAATGGTGAACGTAAGTTTGGCATTGCT 1726
YS_P._kawadai_YW12_1______          TCGTTGTTAAGTGGAGAGAGTTTAATGGTGAACGTAAGTTTGGCATTGCT 1723
YS_P._takakuwai_YW13_1______        TCGTTGTTAAGTGGAGAGAGTTTAATGGTGAACGTAAGTTTGGCATTGCT 1846
YS_P._albisomni_YW19_1______        TCGTTGTTAAGTGGAGAGAGTTTAATGGTGAACGTAAGTTTGGCATTGCT 1723
YS_P._albisomni_YW21_1______        TCGTTGTTAAGTGGAGAGAGTTTAATGGTGAACGTAAGTTTGGCATTGCT 1723
YS_P._albisomni_YW23_1______        TCGTTGTTAAGTGGAGAGAGTTTAATGGTGAACGTAAGTTTGGCATTGCT 1721
YS_P._kawadai_YW03_1________        TCGTTGTTAAGTGGAGAGAGTTTAATGGTGAACGTAAGTTTGGCATTGCT 1723
YS_P._takakuwai_YW54_1______        TCGTTGTTAAGTGGAGAGAGTTTAATGGTGAACGTAAGTTTGGCATTGCT 1723
YS_P._takakuwai_YW73_3______        TCGTTGTTAAGTGGAGAGAGTTTAATGGTGAACGTAAGTTTGGCATTGCT 1726
YS_P._acuticollis_YW07_8____        TCGTTGTTAAGTGGAGAGAGTTTAATGGTGAACGTAAGTTTGGCATTGCT 1725
YS_P._albisomni_YW08_1______        TCGTTGTTAAGTGGAGAGAGTTTAATGGTGAACGTAAGTTTGGCATTGCT 1724
YS_P._albisomni_YW09_1______        TCGTTGTTAAGTGGAGAGAGTTTAATGGTGAACGTAAGTTTGGCATTGCT 1725
YS_P._takakuwai_YW38_1______        TCGTTGTTAAGTGGAGAGAGTTTAATGGTGAACGTAAGTTTGGCATTGCT 1725
YS_P._angularis_YW25_8______        TCGTTGTTAAGTGGAGAGAGTTTAATGGTGAACGTAAGTTTGGCATTGCT 1758
                                    * ************************************************

YS_P._viridicuprus_YW75_3___        TTTTACGCTATGACAACATGGG-TGATCTTGCCTTGTGCAAGAAAACCTG 1775
YS_P._viridicuprus_YW76_3___        TTTTACGCTATGACAACATGGG-TGATCTTGCCTTGTGCAAGAAAACCTG 1775
YS_P._viridicuprus_YW78_3___        TTTTACGCTATGACAACATGGG-TGATCTTGCCTTGTGCAAGAAAACCTG 1777
YS_P._viridicuprus_YW58_1___        TTTTACGCTATGACAACATGGG-TGATCTTGCCTTGTGCAAGAAAACCTG 1773
YS_P._viridicuprus_YW04_1___        TTTTACGCTATGACAACATGGG-TGATCTTGCCTTGTGCAAGAAAACCTG 1776
YS_P._hongwonpyoi_YW34_8____        TTTTACGCTATGACAACATGGG-TGATCTTGCCTTGTGCAAGAAAACCTG 1777
YS_P._hongwonpyoi_YW34_2a___        TTTTACGCTATGACAACATGGG-TGATCTTGCCTTGTGCAAGAAAACCTG 1779
YS_P._hongwonpyoi_YW33_8____        TTTTACGCTATGACAACATGGG-TGATCTTGCCTTGTGCAAGAAAACCTG 1774
YS_P._hongwonpyoi_YW24_1____        TTTTACGCTATGACAACATGGG-TGATCTTGCCTTGTGCAAGAAAACCTG 1776
YS_P._hongwonpyoi_YW05_8____        TTTTACGCTATGACAACATGGG-TGATCTTGCCTTGTGCAAGAAAACCTG 1776
YS_P._hongwonpyoi_YW35_8___         TTTTACGCTATGACAACATGGG-TGATCTTGCCTTGTGCAAGAAAACCTG 1776
YS_P._hongwonpyoi_YW37_8____        TTTTACGCTATGACAACATGGG-TGATCTTGCCTTGTGCAAGAAAACCTG 1778
YS_P._hongwonpyoi_YW36_1____        TTTTACGCTATGACAACATGGG-TGATCTTGCCTTGTGCAAGAAAACCTG 1778
YS_P._sue_YW45_1____________        TTTTACGCTATGACAACATGGG-TGATCTTGCCTTGTGCAAGAAAACCTG 1772
YS_P._sue_YW80_3____________        TTTTACGCTATGACAACATGGG-TGATCTTGCCTTGTGCAAGAAAACCTG 1772
YS_P._takakuwai_YW43_2______        TTTTACGCTATGACAACATGGG-TGATCTTGCCTTGTGCAAGAAAACCTG 1761
YS_P._urushiyamai_YW48_1____        TTTTACGCTATGACAACATGGG-TGATCTTGCCTTGTGCAAGAAAACCTG 1774
YS_P._urushiyamai_YW50_1____        TTTTACGCTATGACAACATGGG-TGATCTTGCCTTGTGCAAGAAAACCTG 1774
YS_P._urushiyamai_YW49_2____        TTTTACGCTATGACAACATGGG-TGATCTTGCCTTGTGCAAGAAAACCTG 1772
YS_P._delicatulus_YW65_2____        TTTTACGCTATGACAACATGGG-TGATCTTGCCTTGTGCAAGAAAACCTG 1781
```

```
YS_P._delicatulus_YW46_1____        TTTTACGCTATGACAACATGGG-TGATCTTGCCTTGTGCAAGAAAACCTG 1781
YS_P._delicatulus_YW10_1____        TTTTACGCTATGACAACATGGG-TGATCTTGCCTTGTGCAAGAAAACCTG 1781
YS_P._delicatulus_YW68_3____        TTTTACGCTATGACAACATGGG-TGATCTTGCCTTGTGCAAGAAAACCTG 1778
YS_P._delicatulus_YW72_2____        TTTTACGCTATGACAACATGGG-TGATCTTGCCTTGTGCAAGAAAACCTG 1779
YS_P._delicatulus_YW47_2____        TTTTACGCTATGACAACATGGG-TGATCTTGCCTTGTGCAAGAAAACCTG 1776
YS_P._takakuwai_YW88_1______        TTTTACGCTATGACAACATGGG-TGATCTTGCCTTGTGCAAGAAAACCTG 1774
YS_P._akitaorum_YW16_1______        TTTTACGCTATGACAACATGGG-TGATCTTGCCTTGTGCAAGAAAACCTG 1775
YS_P._akitaorum_YW14_2______        TTTTACGCTATGACAACATGGG-TGATCTTGCCTTGTGCAAGAAAACCTG 1775
YS_P._kawadai_YW12_1______          TTTTACGCTATGACAACATGGG-TGATCTTGCCTTGTGCAAGAAAACCTG 1772
YS_P._takakuwai_YW13_1______        TTTTACGCTATGACAACATGGG-TGATCTTGCCTTGTGCAAGAAAACCTG 1895
YS_P._albisomni_YW19_1______        TTTTACGCTATGACAACATGGGGTGATCTTGCCTTGTGCAAGAAAACCTG 1773
YS_P._albisomni_YW21_1______        TTTTACGCTATGACAACATGGG-TGATCTTGCCTTGTGCAAGAAAACCTG 1772
YS_P._albisomni_YW23_1______        TTTTACGCTATGACAACATGGG-TGATCTTGCCTTGTGCAAGAAAACCTG 1770
YS_P._kawadai_YW03_1________        TTTTACGCTATGACAACATGGG-TGATCTTGCCTTGTGCAAGAAAACCTG 1772
YS_P._takakuwai_YW54_1______        TTTTACGCTATGACAACATGGG-TGATCTTGCCTTGTGCAAGAAAACCTG 1772
YS_P._takakuwai_YW73_3______        TTTTACGCTATGACAACATGGG-TGATCTTGCCTTGTGCAAGAAAACCTG 1775
YS_P._acuticollis_YW07_8____        TTTTACGCTATGACAACATGGG-TGATCTTGCCTTGTGCAAGAAAACCTG 1774
YS_P._albisomni_YW08_1______        TTTTACGCTATGACAACATGGG-TGATCTTGCCTTGTGCAAGAAAACCTG 1773
YS_P._albisomni_YW09_1______        TTTTACGCTATGACAACATGGG-TGATCTTGCCTTGTGCAAGAAAACCTG 1774
YS_P._takakuwai_YW38_1______        TTTTACGCTATGACAACATGGG-TGATCTTGCCTTGTGCAAGAAAACCTG 1774
YS_P._angularis_YW25_8______        CTTTACGCTATGACAACATGGG-TGATCTTGCCTTGTGCAAGAAAACCTG 1807
                                     ********************* ***************************

YS_P._viridicuprus_YW75_3___        AACAGTCTCATACTAGATATCTAACGATTCTATGTATGAGCGCTGAAGGA 1825
YS_P._viridicuprus_YW76_3___        AACAGTCTCATACTAGATATCTAACGATTCTATGTATGAGCGCTGAAGGA 1825
YS_P._viridicuprus_YW78_3___        AACAGTCTCATACTAGATATCTAACGATTCTATGTATGAGCGCTGAAGGA 1827
YS_P._viridicuprus_YW58_1___        AACAGTCTCATACTAGATATCTAACGATTCTATGTATGAGCGCTGAAGGA 1823
YS_P._viridicuprus_YW04_1___        AACAGTCTCATACTAGATATCTAACGATTCTATGTATGAGCGCTGAAGGA 1826
YS_P._hongwonpyoi_YW34_8____        AACAGTCTCATACTAGATATCTAACGATTCTATGTATGAGCGCTGAAGGA 1827
YS_P._hongwonpyoi_YW34_2a___        AACAGTCTCATACTAGATATCTAACGATTCTATGTATGAGCGCTGAAGGA 1829
YS_P._hongwonpyoi_YW33_8____        AACAGTCTCATACTAGATATCTAACGATTCTATGTATGAGCGCTGAAGGA 1824
YS_P._hongwonpyoi_YW24_1____        AACAGTCTCATACTAGATATCTAACGATTCTATGTATGAGCGCTGAAGGA 1826
YS_P._hongwonpyoi_YW05_8____        AACAGTCTCATACTAGATATCTAACGATTCTATGTATGAGCGCTGAAGGA 1826
YS_P._hongwonpyoi_YW35_8___         AACAGTCTCATACTAGATATCTAACGATTCTATGTATGAGCGCTGAAGGA 1826
YS_P._hongwonpyoi_YW37_8____        AACAGTCTCATACTAGATATCTAACGATTCTATGTATGAGCGCTGAAGGA 1828
YS_P._hongwonpyoi_YW36_1____        AACAGTCTCATACTAGATATCTAACGATTCTATGTATGAGCGCTGAAGGA 1828
```

YS_P._sue_YW45_1____________ AACAGTCTCATACTAGATATCTAACGATTCTATGTATGAGCGCTGAAGGA 1822

YS_P._sue_YW80_3____________ AACAGTCTCATACTAGATATCTAACGATTCTATGTATGAGCGCTGAAGGA 1822

YS_P._takakuwai_YW43_2______ AACAGTCTCATACTAGATATCTAACGATTCTATGTATGAGCGCTGAAGGA 1811

YS_P._urushiyamai_YW48_1____ AACAGTCTCATACTAGATATCTAACGATTCTATGTATGAGCGCTGAAGGA 1824

YS_P._urushiyamai_YW50_1____ AACAGTCTCATACTAGATATCTAACGATTCTATGTATGAGCGCTGAAGGA 1824

YS_P._urushiyamai_YW49_2____ AACAGTCTCATACTAGATATCTAACGATTCTATGTATGAGCGCTGAAGGA 1822

YS_P._delicatulus_YW65_2____ AACAGTCTCATACTAGATATCTAACGATTCTATGTATGAGCGCTGAAGGA 1831

YS_P._delicatulus_YW46_1____ AACAGTCTCATACTAGATATCTAACGATTCTATGTATGAGCGCTGAAGGA 1831

YS_P._delicatulus_YW10_1____ AACAGTCTCATACTAGATATCTAACGATTCTATGTATGAGCGCTGAAGGA 1831

YS_P._delicatulus_YW68_3____ AACAGTCTCATACTAGATATCTAACGATTCTATGTATGAGCGCTGAAGGA 1828

YS_P._delicatulus_YW72_2____ AACAGTCTCATACTAGATATCTAACGATTCTATGTATGAGCGCTGAAGGA 1829

YS_P._delicatulus_YW47_2____ AACAGTCTCATACTAGATATCTAACGATTCTATGTATGAGCGCTGAAGGA 1826

YS_P._takakuwai_YW88_1______ AACAGTCTCATACTAGATATCTAACGATTCTATGTATGAGCGCTGAAGGA 1824

YS_P._akitaorum_YW16_1______ AACAGTCTCATACTAGATATCTAACGATTCTATGTATGAGCGCTGAAGGA 1825

YS_P._akitaorum_YW14_2______ AACAGTCTCATACTAGATATCTAACGATTCTATGTATGAGCGCTGAAGGA 1825

YS_P._kawadai_YW12_1______ AACAGTCTCATACTAGATATCTAACGATTCTATGTATGAGCGCTGAAGGA 1822

YS_P._takakuwai_YW13_1______ AACAGTCTCATACTAGATATCTAACGATTCTATGTATGAGCGCTGAAGGA 1945

YS_P._albisomni_YW19_1______ AACAGTCTCATACTAGATATCTAACGATTCTATGTATGAGCGCTGAAGGA 1823

YS_P._albisomni_YW21_1______ AACAGTCTCATACTAGATATCTAACGATTCTATGTATGAGCGCTGAAGGA 1822

YS_P._albisomni_YW23_1______ AACAGTCTCATACTAGATATCTAACGATTCTATGTATGAGCGCTGAAGGA 1820

YS_P._kawadai_YW03_1________ AACAGTCTCATACTAGATATCTAACGATTCTATGTATGAGCGCTGCAGGA 1822

YS_P._takakuwai_YW54_1______ AACAGTCTCATACTAGATATCTAACGATTCTATGTATGAGCGCTGAAGGA 1822

YS_P._takakuwai_YW73_3______ AACAGTCTCATACTAGATATCTAACGATTCTATGTATGAGCGCTGAAGGA 1825

YS_P._acuticollis_YW07_8____ AACAGTCTCATACTAGATATCTAACGATTCTATGTATGAGCGCTGAAGGA 1824

YS_P._albisomni_YW08_1______ AACAGTCTCATACTAGATATCTAACGATTCTATGTATGAGCGCTGAAGGA 1823

YS_P._albisomni_YW09_1______ AACAGTCTCATACTAGATATCTAACGATTCTATGTATGAGCGCTGAAGGA 1824

YS_P._takakuwai_YW38_1______ AACAGTCTCATACTAGATATCTAACGATTCTATGTATGAGCGCTGAAGGA 1824

YS_P._angularis_YW25_8______ AACAGTCTCATACTAGATATCTAACGATTCTATGTATGAGCGCTGAAGGA 1857

*** ****

YS_P._viridicuprus_YW75_3___ TTTAGTGGATTACTAGCTGATAGCAAACATTCATTAAAACTTATTTGGGG 1875

YS_P._viridicuprus_YW76_3___ TTTAGTGGATTACTAGCTGATAGCAAACATTCATTAAAACTTATTTGGGG 1875

YS_P._viridicuprus_YW78_3___ TTTAGTGGATTACTAGCTGATAGCAAACATTCATTAAAACTTATTTGGGG 1877

YS_P._viridicuprus_YW58_1___ TTTAGTGGATTACTAGCTGATAGCAAACATTCATTAAAACTTATTTGGGG 1873

YS_P._viridicuprus_YW04_1___ TTTAGTGGATTACTAGCTGATAGCAAACATTCATTAAAACTTATTTGGGG 1876

YS_P._hongwonpyoi_YW34_8____ TTTAGTGGATTACTAGCTGATAGCAAACATTCATTAAAACTTATTTGGGG 1877

```
YS_P._hongwonpyoi_YW34_2a___     TTTAGTGGATTACTAGCTGATAGCAAACATTCATTAAAACTTATTTGGGG 1879
YS_P._hongwonpyoi_YW33_8____     TTTAGTGGATTACTAGCTGATAGCAAACATTCATTAAAACTTATTTGGGG 1874
YS_P._hongwonpyoi_YW24_1____     TTTAGTGGATTACTAGCTGATAGCAAACATTCATTAAAACTTATTTGGGG 1876
YS_P._hongwonpyoi_YW05_8____     TTTAGTGGATTACTAGCTGATAGCAAACATTCATTAAAACTTATTTGGGG 1876
YS_P._hongwonpyoi_YW35_8___      TTTAGTGGATTACTAGCTGATAGCAAACATTCATTAAAACTTATTTGGGG 1876
YS_P._hongwonpyoi_YW37_8____     TTTAGTGGATTACTAGCTGATAGCAAACATTCATTAAAACTTATTTGGGG 1878
YS_P._hongwonpyoi_YW36_1____     TTTAGTGGATTACTAGCTGATAGCAAACATTCATTAAAACTTATTTGGGG 1878
YS_P._sue_YW45_1_____________    TTTGGTGGATTACTAGCTGATAGCAAACATTCATTAAAACTTATTTGGGG 1872
YS_P._sue_YW80_3_____________    TTTGGTGGATTACTAGCTGATAGCAAACATTCATTAAAACTTATTTGGGG 1872
YS_P._takakuwai_YW43_2_______    TTTGGTGGATTACTAGCTGATAGCAAACATTCATTAAAACTTATTTGGGG 1861
YS_P._urushiyamai_YW48_1_____    TTTGGTGGATTACTAGCTGATAGCAAACATTCATTAAAACTTATTTGGGG 1874
YS_P._urushiyamai_YW50_1_____    TTTGGTGGATTACTAGCTGATAGCAAACATTCATTAAAACTTATTTGGGG 1874
YS_P._urushiyamai_YW49_2_____    TTTGGTGGATTACTAGCTGATAGCAAACATTCATTAAAACTTATTTGGGG 1872
YS_P._delicatulus_YW65_2_____    TTTAGTGGATTACTAGCTGATAGCAAACATTCATTAAAACTTATTTGGGG 1881
YS_P._delicatulus_YW46_1_____    TTTAGTGGATTACTAGCTGATAGCAAACATTCATTAAAACTTATTTGGGG 1881
YS_P._delicatulus_YW10_1_____    TTTAGTGGATTACTAGCTGATAGCAAACATTCATTAAAACTTATTTGGGG 1881
YS_P._delicatulus_YW68_3_____    TTTGGTGGATTACTAGCTGATAGCAAACATTCATTAAAACTTATTTGGGG 1878
YS_P._delicatulus_YW72_2_____    TTTGGTGGATTACTAGCTGATAGCAAACATTCATTAAAACTTATTTGGGG 1879
YS_P._delicatulus_YW47_2_____    TTTGGTGGATTACTAGCTGATAGCAAACATTCATTAAAACTTATTTGGGG 1876
YS_P._takakuwai_YW88_1_______    TTTAGTGGATTACTAGCTGATAGCAAACATTCATTAAAACTTTTTTGGGG 1874
YS_P._akitaorum_YW16_1_______    TTTAGTGGATTACTAGCTGATAGCAAACATTCATTAAAACTTTTTTGGGG 1875
YS_P._akitaorum_YW14_2_______    TTTAGTGGATTACTAGCTGATAGCAAACATTCATTAAAACTTTTTTGGGG 1875
YS_P._kawadai_YW12_1_______      TTTAGTGGATTACTAGCTGATAGCAAACATTCATTAAAACTTATTTGGGG 1872
YS_P._takakuwai_YW13_1_______    TTTAGTGGATTACTAGCTGATAGCAAACATTCATTAAAACTTATTTGGGG 1995
YS_P._albisomni_YW19_1_______    TTTAGTGGATTACTAGCTGATAGCAAACATTCATTAAAACTTATTTGGGG 1873
YS_P._albisomni_YW21_1_______    TTTAGTGGATTACTAGCTGATAGCAAACATTCATTAAAACTTATTTGGGG 1872
YS_P._albisomni_YW23_1_______    TTTAGTGGATTACTAGCTGATAGCAAACATTCATTAAAACTTATTTGGGG 1870
YS_P._kawadai_YW03_1_________    TTTAGTGGATTACTAGCTAATAGCAAACATTCATTAAAACTTATTTGGGG 1872
YS_P._takakuwai_YW54_1_______    TTTAGTGGATTACTAGCTGATAGCAAACATTCATTAAAACTTATTTGGGG 1872
YS_P._takakuwai_YW73_3_______    TTTAGTGGATTACTAGCTGATAGCAAACATTCATTAAAACTTATTTGGGG 1875
YS_P._acuticollis_YW07_8_____    TTTAGTGGATTACTAGCTGATAGCAAACATTCATTAAAACTTATTTGGGG 1874
YS_P._albisomni_YW08_1_______    TTTAGTGGATTACTAGCTGATAGCAAACATTCATTAAAACTTATTTGGGG 1873
YS_P._albisomni_YW09_1_______    TTTAGTGGATTACTAGCTGATAGCAAACATTCATTAAAACTTATTTGGGG 1874
YS_P._takakuwai_YW38_1_______    TTTAGTGGATTACTAGCTGATAGCAAACATTCATTAAAACTTATTTGGGG 1874
YS_P._angularis_YW25_8_______    TTTAGTGGATTACTAGCTGATAGCAAACATTCATTAAAACTTATTTGGGG 1907
                                 *** ************** *********************** *******
```

YS_P._viridicuprus_YW75_3___	GTCTGCCTTGACAAATAGCCGGATTAAGTTCTGTGATTTTGCCAAGTGTA	1925
YS_P._viridicuprus_YW76_3___	GTCTGCCTTGACAAATAGCCGGATTAAGTTCTGTGATTTTGCCAAGTGTA	1925
YS_P._viridicuprus_YW78_3___	GTCTGCCTTGACAAATAGCCGGATTAAGTTCTGTGATTTTGCCAAGTGTA	1927
YS_P._viridicuprus_YW58_1___	GTCTGCCTTGACAAATAGCCGGATTAAGTTCTGTGATTTTGCCAAGTGTA	1923
YS_P._viridicuprus_YW04_1___	GTCTGCCTTGACAAATAGCCGGATTAAGTTCTGTGATTTTGCCAAGTGTA	1926
YS_P._hongwonpyoi_YW34_8____	GTCTGCCTTGACAAATAGCCGGATTAAGTTCTGTGATTTTGCCAAGTGTA	1927
YS_P._hongwonpyoi_YW34_2a___	GTCTGCCTTGACAAATAGCCGGATTAAGTTCTGTGATTTTGCCAAGTGTA	1929
YS_P._hongwonpyoi_YW33_8____	GTCTGCCTTGACAAATAGCCGGATTAAGTTCTGTGATTTTGCCAAGTGTA	1924
YS_P._hongwonpyoi_YW24_1____	GTCTGCCTTGACAAATAGCCGGATTAAGTTCTGTGATTTTGCCAAGTGTA	1926
YS_P._hongwonpyoi_YW05_8____	GTCTGCCTTGACAAATAGCCGGATTAAGTTCTGTGATTTTGCCAAGTGTA	1926
YS_P._hongwonpyoi_YW35_8___	GTCTGCCTTGACAAATAGCCGGATTAAGTTCTGTGATTTTGCCAAGTGTA	1926
YS_P._hongwonpyoi_YW37_8____	GTCTGCCTTGACAAATAGCCGGATTAAGTTCTGTGATTTTGCCAAGTGTA	1928
YS_P._hongwonpyoi_YW36_1____	GTCTGCCTTGACAAATAGCCGGATTAAGTTCTGTGATTTTGCCAAGTGTA	1928
YS_P._sue_YW45_1____________	GTCTGCCTTGACAAATAGCCGGATTAAGTTCTGTGATTTTGCCAAGTGTA	1922
YS_P._sue_YW80_3____________	GTCTGCCTTGACAAATAGCCGGATTAAGTTCTGTGATTTTGCCAAGTGTA	1922
YS_P._takakuwai_YW43_2______	GTCTGCCTTGACAAATAGCCGGATTAAGTTCTGTGATTTTGCCAAGTGTA	1911
YS_P._urushiyamai_YW48_1____	GTCTGCCTTGACAAATAGCCGGATTAAGTTCTGTGATTTTGCCAAGTGTA	1924
YS_P._urushiyamai_YW50_1____	GTCTGCCTTGACAAATAGCCGGATTAAGTTCTGTGATTTTGCCAAGTGTA	1924
YS_P._urushiyamai_YW49_2____	GTCTGCCTTGACAAATAGCCGGATTAAGTTCTGTGATTTTGCCAAGTGTA	1922
YS_P._delicatulus_YW65_2____	GTCTGCCTTGACAAATAGCCGGATTAAGTTCTGTGATTTTGCCAAGTGTA	1931
YS_P._delicatulus_YW46_1____	GTCTGCCTTGACAAATAGCCGGATTAAGTTCTGTGATTTTGCCAAGTGTA	1931
YS_P._delicatulus_YW10_1____	GTCTGCCTTGACAAATAGCCGGATTAAGTTCTGTGATTTTGCCAAGTGTA	1931
YS_P._delicatulus_YW68_3____	GTCTGCCTTGACAAATAGCCGGATTAAGTTCTGTGATTTTGCCAAGTGTA	1928
YS_P._delicatulus_YW72_2____	GTCTGCCTTGACAAATAGCCGGATTAAGTTCTGTGATTTTGCCAAGTGTA	1929
YS_P._delicatulus_YW47_2____	GTCTGCCTTGACAAATAGCCGGATTAAGTTCTGTGATTTTGCCAAGTGTA	1926
YS_P._takakuwai_YW88_1______	GTCTGCCTTGACAAATAGCCGGATTAAGTTCTGTGATTTTGCCAAGTGTA	1924
YS_P._akitaorum_YW16_1______	GTCTGCCTTGACAAATAGCCGGATTAAGTTCTGTGATTTTGCCAAGTGTA	1925
YS_P._akitaorum_YW14_2______	GTCTGCCTTGACAAATAGCCGGATTAAGTTCTGTGATTTTGCCAAGTGTA	1925
YS_P._kawadai_YW12_1______	GTCTGCCTTGACAAATAGCCGGATTAAGTTCTGTGATTTTGTCAAGTGTA	1922
YS_P._takakuwai_YW13_1______	GTCTGCCTTGACAAATAGCCGGATTAAGTTCTGTGATTTTGTCAAGTGTA	2045
YS_P._albisomni_YW19_1______	GTCTGCCTTGACAAATAGCCGGATTAAGTTCTGTGATTTTGCCAAGTGTA	1923
YS_P._albisomni_YW21_1______	GTCTGCCTTGACAAATAGCCGGATTAAGTTCTGTGATTTTGCCAAGTGTA	1922
YS_P._albisomni_YW23_1______	GTCTGCCTTGACAAATAGCCGGATTAAGTTCTGTGATTTTGCCAAGTGTA	1920
YS_P._kawadai_YW03_1________	GTCTGCCTTGACAAATAGCCGGATTAAGTTCTGTGATTTTGCCAAGTGTA	1922
YS_P._takakuwai_YW54_1______	GTCTGCCTTGACAAATAGCCGGATTAAGTTCTGTGATTTTGCCAAGTGTA	1922

```
YS_P._takakuwai_YW73_3______      GTCTGCCTTGACAAATAGCCGGATTAAGTTCTGTGATTTTGCCAAGTGTA 1925
YS_P._acuticollis_YW07_8____      GTCTGCCTTGACAAATAGCCGGATTAAGTTCTGTGATTTTGCCAAGTGTA 1924
YS_P._albisomni_YW08_1______      GTCTGCCTTGACAAATAGCCGGATTAAGTTCTGTGATTTTGCCAAGTGTA 1923
YS_P._albisomni_YW09_1______      GTCTGCCTTGACAAATAGCCGGATTAAGTTCTGTGATTTTGCCAAGTGTA 1924
YS_P._takakuwai_YW38_1______      GTCTGCCTTGACAAATAGCCGGATTAAGTTCTGTGATTTTGCCAAGTGTA 1924
YS_P._angularis_YW25_8______      GTCTGCCTTGACAAATAGCCGGATTAAGTTCTGTGATTTTGCCAAGTGTA 1957
                                  ***************************************** ********

YS_P._viridicuprus_YW75_3___      ACCTCCTCCATGGTTGGTTATGTAAAACCTTGTGTTGTAAAAAACTCGAG 1975
YS_P._viridicuprus_YW76_3___      ACCTCCTCCATGGTTGGTTATGTAAAACCTTGTGTTGTAAAAAACTCGAG 1975
YS_P._viridicuprus_YW78_3___      ACCTCCTCCATGGTTGGTTATGTAAAACCTTGTGTTGTAAAAAACTCGAG 1977
YS_P._viridicuprus_YW58_1___      ACCTCCTCCATGGTTGGTTATGTAAAACCTTGTGTTGTAAAAAACTCGAG 1973
YS_P._viridicuprus_YW04_1___      ACCTCCTCCATGGTTGGTTATGTAAAACCTTGTGTTGTAAAAAACTCGAG 1976
YS_P._hongwonpyoi_YW34_8____      ACCTCCTCCATGGTTGGTTATGTAAAACCTTGTGTTGTAAAAAACTCGAG 1977
YS_P._hongwonpyoi_YW34_2a___      ACCTCCTCCATGGTTGGTTATGTAAAACCTTGTGTTGTAAAAAACTCGAG 1979
YS_P._hongwonpyoi_YW33_8____      ACCTCCTCCATGGTTGGTTATGTAAAACCTTGTGTTGTAAAAAACTCGAG 1974
YS_P._hongwonpyoi_YW24_1____      ACCTCCTCCATGGTTGGTTATGTAAAACCTTGTGTTGTAAAAAACTCGAG 1976
YS_P._hongwonpyoi_YW05_8____      ACCTCCTCCATGGTTGGTTATGTAAAACCTTGTGTTGTAAAAAACTCGAG 1976
YS_P._hongwonpyoi_YW35_8___       ACCTCCTCCATGGTTGGTTATGTAAAACCTTGTGTTGTAAAAAACTCGAG 1976
YS_P._hongwonpyoi_YW37_8____      ACCTCCTCCATGGTTGGTTATGTAAAACCTTGTGTTGTAAAAAACTCGAG 1978
YS_P._hongwonpyoi_YW36_1____      ACCTCCTCCATGGTTGGTTATGTAAAACCTTGTGTTGTAAAAAACTCGAG 1978
YS_P._sue_YW45_1____________      ACCTCCTCCATGGTTGGTTATGTAAAACCTTGTGTTGTAAAAAACTCGAG 1972
YS_P._sue_YW80_3____________      ACCTCCTCCATGGTTGGTTATGTAAAACCTTGTGTTGTAAAAAACTCGAG 1972
YS_P._takakuwai_YW43_2______      ACCTCCTCCATGGTTGGTTATGTAAAACCTTGTGTTGTAAAAAACTCGAG 1961
YS_P._urushiyamai_YW48_1____      ACCTCCTCCATGGTTGGTTATGTAAAACCTTGTGTTGTAAAAAACTCGAG 1974
YS_P._urushiyamai_YW50_1____      ACCTCCTCCATGGTTGGTTATGTAAAACCTTGTGTTGTAAAAAACTCGAG 1974
YS_P._urushiyamai_YW49_2____      ACCTCCTCCATGGTTGGTTATGTAAAACCTTGTGTTGTAAAAAACTCGAG 1972
YS_P._delicatulus_YW65_2____      ACCTCCTCCATGGTTGGTTATGTAAAACCTTGTGTTGTAAAAAGCTCGAG 1981
YS_P._delicatulus_YW46_1____      ACCTCCTCCATGGTTGGTTATGTAAAACCTTGTGTTGTAAAAAGCTCGAG 1981
YS_P._delicatulus_YW10_1____      ACCTCCTCCATGGTTGGTTATGTAAAACCTTGTGTTGTAAAAAACTCGAG 1981
YS_P._delicatulus_YW68_3____      ACCTCCTCCATGGTTGGTTATGTAAAACCTTGTGTTGTAAAAAACTCGAG 1978
YS_P._delicatulus_YW72_2____      ACCTCCTCCATGGTTGGTTATGTAAAACCTTGTGTTGTAAAAAACTCGAG 1979
YS_P._delicatulus_YW47_2____      ACCTCCTCCATGGTTGGTTATGTAAAACCTTGTGTTGTAAAAAACTCGAG 1976
YS_P._takakuwai_YW88_1______      ACCTCCTCCATGGTTGGTTATGTAAAACCTTGTGTTGTAAAAAACTCGAG 1974
YS_P._akitaorum_YW16_1______      ACCTCCTCCATGGTTGGTTATGTAAAACCTTGTGTTGTAAAAAACTCGAG 1975
YS_P._akitaorum_YW14_2______      ACCTCCTCCATGGTTGGTTATGTAAAACCTTGTGTTGTAAAAAACTCGAG 1975
```

```
YS_P._kawadai_YW12_1______        ACCTCCTCCATGGTTGGTTATGTAAAACCTTGTGTTGTAAAAAACTCGAG 1972
YS_P._takakuwai_YW13_1______      ACCTCCTCCATGGTTGGTTATGTAAAACCTTGTGTTGTAAAAAACTCGAG 2095
YS_P._albisomni_YW19_1______      ACCTCCTCCATGGTTGGTTATGTAAAACCTTGTGTTGTAAAAAACTCGAG 1973
YS_P._albisomni_YW21_1______      ACCTCCTCCATGGTTGGTTATGTAAAACCTTGTGTTGTAAAAAACTCGAG 1972
YS_P._albisomni_YW23_1______      ACCTCCTCCATGGTTGGTTATGTAAAACCTTGTGTTGTAAAAAACTCGAG 1970
YS_P._kawadai_YW03_1________      ACCTCCTCCATGGTTGGTTATGTAAAACCTTGTGTTGTAAAAAACTCGAG 1972
YS_P._takakuwai_YW54_1______      ACCTCCTCCATGGTTGGTTATGTAAAACCTTGTGTTGTAAAAAACTCGAG 1972
YS_P._takakuwai_YW73_3______      ACCTCCTCCATGGTTGGTTATGTAAAACCTTGTGTTGTAAAAAACTCGAG 1975
YS_P._acuticollis_YW07_8____      ACCTCCTCCATGGTTGGTTATGTAAAACCTTGTGTTGTAAAAAACTCGAG 1974
YS_P._albisomni_YW08_1______      ACCTCCTCCATGGTTGGTTATGTAAAACCTTGTGTTGTAAAAAACTCGAG 1973
YS_P._albisomni_YW09_1______      ACCTCCTCCATGGTTGGTTATGTAAAACCTTGTGTTGTAAAAAACTCGAG 1974
YS_P._takakuwai_YW38_1______      ACCTCCTCCATGGTTGGTTATGTAAAACCTTGTGTTGTAAAAAACTCGAG 1974
YS_P._angularis_YW25_8______      ACCTCCTCCATGGTTGGTTATGTAAAACCTTGTGTTGTAAAAAGCTCGAG 2007
                                  ******************************************* ******

YS_P._viridicuprus_YW75_3___      TGTTTTGCGGACATCATACCAGGGGAATTCAAAGAGGGCTATATGGATGT 2025
YS_P._viridicuprus_YW76_3___      TGTTTTGCGGACATCATACCAGGGGAATTCAAAGAGGGCTATATGGATGT 2025
YS_P._viridicuprus_YW78_3___      TGTTTTGCGGACATCATACCAGGGGAATTCAAAGAGGGCTATGTGGATGT 2027
YS_P._viridicuprus_YW58_1___      TGTTTTGCGGACATCATACCAGGGGAATTCAAAGAGGGCTATATGGATGT 2023
YS_P._viridicuprus_YW04_1___      TGTTTTGCGGACATCATACCAGGGGAATTCAAAGAGGGCTATATGGATGT 2026
YS_P._hongwonpyoi_YW34_8____      TGTTTTGCGGACATCATACCAGGGGAATTCAAAGAGGGCTATATGGATGT 2027
YS_P._hongwonpyoi_YW34_2a___      TGTTTTGCGGACATCATACCAGGGGAATTCAAAGAGGGCTATATGGATGT 2029
YS_P._hongwonpyoi_YW33_8____      TGTTTTGCGGACATCATACCAGGGGAATTCAAAGAGGGCTATATGGATGT 2024
YS_P._hongwonpyoi_YW24_1____      TGTTTTGCGGACATCATACCAGGGGAATTCAAAGAGGGCTATATGGATGT 2026
YS_P._hongwonpyoi_YW05_8____      TGTTTTGCGGACATCATACCAGGGGAATTCAAAGAGGGCTATATGGATGT 2026
YS_P._hongwonpyoi_YW35_8___       TGTTTTGCGGACATCATACCAGGGGAATTCAAAGAGGGCTATATGGATGT 2026
YS_P._hongwonpyoi_YW37_8____      TGTTTTGCGGACATCATACCAGGGGAATTCAAAGAGGGCTATATGGATGT 2028
YS_P._hongwonpyoi_YW36_1____      TGTTTTGCGGACATCATACCAGGGGAATTCAAAGAGGGCTATATGGATGT 2028
YS_P._sue_YW45_1____________      TGTTTTGCGGACATCATACCAGGGGAATTCAAAGAGGGCTATATGGATGT 2022
YS_P._sue_YW80_3____________      TGTTTTGCGGACATCATACCAGGGGAATTCAAAGAGGGCTATATGGATGT 2022
YS_P._takakuwai_YW43_2______      TGTTTTGCGGACATCATACCAGGGGAATTCAAAGAGGGCTATATGGATGT 2011
YS_P._urushiyamai_YW48_1____      TGTTTTGCGGACATCATACCAGGGGAATTCAAAGAGGGCTATATGGATGT 2024
YS_P._urushiyamai_YW50_1____      TGTTTTGCGGACATCATACCAGGGGAATTCAAAGAGGGCTATATGGATGT 2024
YS_P._urushiyamai_YW49_2____      TGTTTTGCGGACATCATACCAGGGGAATTCAAAGAGGGCTATATGGATGT 2022
YS_P._delicatulus_YW65_2____      TGTTTTGCGGACATCATACCAGGGGAATTCAAAGAGGGCTATATGGATGT 2031
YS_P._delicatulus_YW46_1____      TGTTTTGCGGACATCATACCAGGGGAATTCAAAGAGGGCTATATGGATGT 2031
```

```
YS_P._delicatulus_YW10_1____        TGTTTTGCGGACATCATACCAGGGGAATTCAAAGAGGGCTATATGGATGT 2031
YS_P._delicatulus_YW68_3____        TGTTTTGCGGACATCATACCAGGGGAATTCAAAGAGGGCTATATGGATGT 2028
YS_P._delicatulus_YW72_2____        TGTTTTGCGGACATCATACCAGGGGAATTCAAAGAGGGCTATATGGATGT 2029
YS_P._delicatulus_YW47_2____        TGTTTTGCGGACATCATACCAGGGGAATTCAAAGAGGGCTATATGGATGT 2026
YS_P._takakuwai_YW88_1______        TGTTTTGCGGACATCATACCAGGGGAATTCAAAGAGGGCTATATGGATGT 2024
YS_P._akitaorum_YW16_1______        TGTTTTGCGGACATCATACCAGGGGAATTCAAAGAGGGCTATATGGATGT 2025
YS_P._akitaorum_YW14_2______        TGTTTTGCGGACATCATACCAGGGGAATTCAAAGAGGGCTATATGGATGT 2025
YS_P._kawadai_YW12_1______          TGTTTTGCGGACATCATACCAGGGGAATTCAAAGAGGGCTATATGGATGT 2022
YS_P._takakuwai_YW13_1______        TGTTTTGCGGACATCATACCAGGGGAATTCAAAGAGGGCTATATGGATGT 2145
YS_P._albisomni_YW19_1______        TGTTTTGCGGACATCATACCAGGGGAATTCAAAGAGGGCTATATGGATGT 2023
YS_P._albisomni_YW21_1______        TGTTTTACGGACATCATACCAGGGGAATTCAAAGAGGGCTATATGGATGT 2022
YS_P._albisomni_YW23_1______        TGTTTTACGGACATCATACCAGGGGAATTCAAAGAGGGCTATATGGATGT 2020
YS_P._kawadai_YW03_1________        TGTTTTGCGGACATCATACCAGGGGAATTCAAAGAGGGCTATATGGATGT 2022
YS_P._takakuwai_YW54_1______        TGTTTTGCGGACATCATACCAGGGGAATTCAAAGAGGGCTATATGGATGT 2022
YS_P._takakuwai_YW73_3______        TGTTTTGCGGACATCATACCAGGGGAATTCAAAGAGGGCTATATGGATGT 2025
YS_P._acuticollis_YW07_8____        TGTTTTGCGGACATCATACCAGGGGAATTCAAAGAGGGCTATATGGATGT 2024
YS_P._albisomni_YW08_1______        TGTTTTGCGGACATCATACCAGGGGAATTCAAAGAGGGCTATATGGATGT 2023
YS_P._albisomni_YW09_1______        TGTTTTGCGGACATCATACCAGGGGAATTCAAAGAGGGCTATATGGATGT 2024
YS_P._takakuwai_YW38_1______        TGTTTTGCGGACATCATACCAGGGGAATTCAAAGAGGGCTATATGGATGT 2024
YS_P._angularis_YW25_8______        TGTTTTGCGGACATCATACCAGGGGAATTCAAAGAGGGCTATATGGATGT 2057
                                    ****** *********************************** *******

YS_P._viridicuprus_YW75_3___        ATTTGGTTGCGACTCGAGCGTAAAAACTTGAGACGCGAGCACATACCGAT 2075
YS_P._viridicuprus_YW76_3___        ATTTGGTTGCGACTCGAGCGTAAAAACTTGAGACGCGAGCACATACCGAT 2075
YS_P._viridicuprus_YW78_3___        ATTTGGTTGCGACTCGAGCGTAAAAACTTGAGACGCGAGCACATACCGAT 2077
YS_P._viridicuprus_YW58_1___        ATTTGGTTGCGACTCGAGCGTAAAAACTTGAGACGCGAGCACATACCGAT 2073
YS_P._viridicuprus_YW04_1___        ATTTGGTTGCGACTCGAGCGTAAAAACTTGAGACGCGAGCACATACCGAT 2076
YS_P._hongwonpyoi_YW34_8____        ATTTGGTTGCGACTCGAGCGTAAAAACTTGAGACGCGAGCACATACCGAT 2077
YS_P._hongwonpyoi_YW34_2a___        ATTTGGTTGCGACTCGAGCGTAAAAACTTGAGACGCGAGCACATACCGAT 2079
YS_P._hongwonpyoi_YW33_8____        ATTTGGTTGCGACTCGAGCGTAAAAACTTGAGACGCGAGCACATACCGAT 2074
YS_P._hongwonpyoi_YW24_1____        ATTTGGTTGCGACTCGAGCGTAAAAACTTGAGACGCGAGCACATACCGAT 2076
YS_P._hongwonpyoi_YW05_8____        ATTTGGTTGCGACTCGAGCGTAAAAACTTGAGACGCGAGCACATACCGAT 2076
YS_P._hongwonpyoi_YW35_8___         ATTTGGTTGCGACTCGAGCGTAAAAACTTGAGACGCGAGCACATACCGAT 2076
YS_P._hongwonpyoi_YW37_8____        ATTTGGTTGCGACTCGAGCGTAAAAACTTGAGACGCGAGCACATACCGAT 2078
YS_P._hongwonpyoi_YW36_1____        ATTTGGTTGCGACTCGAGCGTAAAAACTTGAGACGCGAGCACATACCGAT 2078
YS_P._sue_YW45_1____________        ATTTGGTTGCGACTCGAGCGTAAAAACTTGAGACGCGAGCACATACCGAT 2072
```

```
YS_P._sue_YW80_3____________        ATTTGGTTGCGACTCGAGCGTAAAAACTTGAGACGCGAGCACATACCGAT 2072
YS_P._takakuwai_YW43_2______        ATTTGGTTGCGACTCGAGCGTAAAAACTTGAGACGCGAGCACATACCGAT 2061
YS_P._urushiyamai_YW48_1____        ATTTGGTTGCGACTCGAGCGTAAAAACTTGAGACGCGAGCACATACCGAT 2074
YS_P._urushiyamai_YW50_1____        ATTTGGTTGCGACTCGAGCGTAAAAACTTGAGACGCAAGCACATACCGAT 2074
YS_P._urushiyamai_YW49_2____        ATTTGGTTGCGACTCGAGCGTAAAAACTTGAGACGCGAGCACATACCGAT 2072
YS_P._delicatulus_YW65_2____        ATTTGGTTGCGACTCGAGCGTAAAAGCTTGAGACGCGAGCACATACCGAT 2081
YS_P._delicatulus_YW46_1____        ATTTGGTTGCGACTCGAGCGTAAAAGCTTGAGACGCGAGCACATACCGAT 2081
YS_P._delicatulus_YW10_1____        ATTTGGTTGCGACTCGAGCGTAAAAGCTTGAGACGCGAGCACATACCGAT 2081
YS_P._delicatulus_YW68_3____        ATTTGGTTGCGACTCGAGCGTAAAAGCTTGAGACGCGAGCACATACCGAT 2078
YS_P._delicatulus_YW72_2____        ATTTGGTTGCGACTCGAGCGTAAAAGCTTGAGACGCGAGCACATACCGAT 2079
YS_P._delicatulus_YW47_2____        ATTTGGTTGCGACTCGAGCGTAAAAGCTTGAGACGCGAGCACATACCGAT 2076
YS_P._takakuwai_YW88_1______        ATTTGGTTGCGACTCGAGCGTAAAAACTTGAGACGCGAGCACATACCGAT 2074
YS_P._akitaorum_YW16_1______        ATTTGGTTGCGACTCGAGCGTAAAAACTTGAGACGCGAGCACATACCGAT 2075
YS_P._akitaorum_YW14_2______        ATTTGGTTGCGACTCGAGCGTAAAAACTTGAGACGCGAGCACATACCGAT 2075
YS_P._kawadai_YW12_1______          ATTTGGTTGCGACTCGAGCGTAAAAACTTGAGACGCGAGCACATACCGAT 2072
YS_P._takakuwai_YW13_1______        ATTTGGTTGCGACTCGAGCGTAAAAACTTGAGACGCGAGCACATACCGAT 2195
YS_P._albisomni_YW19_1______        ATTTGGTTGCGACTCGAGCGTAAAAACTTGAGACGCGAGCACATACCGAT 2073
YS_P._albisomni_YW21_1______        ATTTGGTTGCGACTCGAGCGTAAAAACTTGAGACGCGAGCACATACCGAT 2072
YS_P._albisomni_YW23_1______        ATTTGGTTGCGACTCGAGCGTAAAAACTTGAGACGCGAGCACATACCGAT 2070
YS_P._kawadai_YW03_1________        ATTTGGTTGCGACTCGAGCGTAAAAACTTGAGACGCGAGCACATACCGAT 2072
YS_P._takakuwai_YW54_1______        ATTTGGTTGCGACTCGAGCGTAAAAACTTGAGACGCGAGCACATACCGAT 2072
YS_P._takakuwai_YW73_3______        ATTTGGTTGCGACTCGAGCGTAAAAACTTGAGACGCGAGCACATACCGAT 2075
YS_P._acuticollis_YW07_8____        ATTTGGTTGCGACTCGAGCGTAAAAACTTGAGACGCGAGCACATACCGAT 2074
YS_P._albisomni_YW08_1______        ATTTGGTTGCGACTCGAGCGTAAAAACTTGAGACGCGAGCACATACCGAT 2073
YS_P._albisomni_YW09_1______        ATTTGGTTGCGACTCGAGCGTAAAAACTTGAGACGCGAGCACATACCGAT 2074
YS_P._takakuwai_YW38_1______        ATTTGGTTGCGACTCGAGCGTAAAAACTTGAGACGCGAGCACATACCGAT 2074
YS_P._angularis_YW25_8______        ATTTGGTTGCGACTCGAGCGTAAAAACTTGAGACGCGAGCACATACCGAT 2107
                                    ************************* ********** *************

YS_P._viridicuprus_YW75_3___        TGAATTTATTCAATTGTCAATTTAGAAAACTTCTTGTCAGTCTGAGAACG 2125
YS_P._viridicuprus_YW76_3___        TGAATTTATTCAATTGTCAATTTAGAAAACTTCTTGTCAGTCTGAGAACG 2125
YS_P._viridicuprus_YW78_3___        TGAATTTATTCAATTGTCAATTTAGAAAACTTCTTGTCAGTCTGAGAACG 2127
YS_P._viridicuprus_YW58_1___        TGAATTTATTCAATTGTCAATTTAGAAAACTTCTTGTCAGTCTGAGAACG 2123
YS_P._viridicuprus_YW04_1___        TGAATTTATTCAATTGTCAATTTAGAAAACTTCTTGTCAGTCTGAGAACG 2126
YS_P._hongwonpyoi_YW34_8____        TGAATTTATTCAATTGTCAATTTAGAAAACTTCTTGTCAGTCTGAGAACG 2127
YS_P._hongwonpyoi_YW34_2a___        TGAATTTATTCAATTGTCAATTTAGAAAACTTCTTGTCAGTCTGAGAACG 2129
```

```
YS_P._hongwonpyoi_YW33_8____      TGAATTTATTCAATTGTCAATTTAGAAAACTTCTTGTCAGTCTGAGAACG 2124
YS_P._hongwonpyoi_YW24_1____      TGAATTTATTCAATTGTCAATTTAGAAAACTTCTTGTCAGTCTGAGAACG 2126
YS_P._hongwonpyoi_YW05_8____      TGAATTTATTCAATTGTCAATTTAGAAAACTTCTTGTCAGTCTGAGAACG 2126
YS_P._hongwonpyoi_YW35_8___       TGAATTTATTCAATTGTCAATTTAGAAAACTTCTTGTCAGTCTGAGAACG 2126
YS_P._hongwonpyoi_YW37_8____      TGAATTTATTCAATTGTCAATTTAGAAAACTTCTTGTCAGTCTGAGAACG 2128
YS_P._hongwonpyoi_YW36_1____      TGAATTTATTCAATTGTCAATTTAGAAAACTTCTTGTCAGTCTGAGAACG 2128
YS_P._sue_YW45_1____________      TGAATTTATTCAATTGTCAATTTAGAAAACTTCTTGTCAGTCTGAGAACG 2122
YS_P._sue_YW80_3____________      TGAATTTATTCAATTGTCAATTTAGAAAACTTCTTGTCAGTCTGAGAACG 2122
YS_P._takakuwai_YW43_2______      TGAATTTATTCAATTGTCAATTTAGAAAACTTCTTGTCAGTCTGAGAACG 2111
YS_P._urushiyamai_YW48_1____      TGAATTTATTCAATTGTCAATTTAGAAAACTTCTTGTCAGTCTGAGAACG 2124
YS_P._urushiyamai_YW50_1____      TGAATTTATTCAATTGTCAATTTAGAAAACTTCTTGTCAGTCTGAGAACG 2124
YS_P._urushiyamai_YW49_2____      TGAATTTATTCAATTGTCAATTTAGAAAACTTCTTGTCAGTCTGAGAACG 2122
YS_P._delicatulus_YW65_2____      TGAATTTATTCAATTGTCAATTTAGAAAACTTCTTGTCAGTCTGGGAACG 2131
YS_P._delicatulus_YW46_1____      TGAATTTATTCAATTGTCAATTTAGAAAACTTCTTGTCAGTCTGGGAACG 2131
YS_P._delicatulus_YW10_1____      TGAATTTATTCAATTGTCAATTTAGAAAACTTCTTGTCAGTCTGGGAACG 2131
YS_P._delicatulus_YW68_3____      TGAATTTATTCAATTGTCAATTTAGAAAACTTCTTGTCAGTCTGAGAACG 2128
YS_P._delicatulus_YW72_2____      TGAATTTATTCAATTGTCAATTTAGAAAACTTCTTGTCAGTCTGAGAACG 2129
YS_P._delicatulus_YW47_2____      TGAATTTATTCAATTGTCAATTTAGAAAACTTCTTGTCAGTCTGAGAACG 2126
YS_P._takakuwai_YW88_1______      TGAATTTATTCAATTGTCAATTTAGAAAACTTCTTGTCAGTCTGAGAACG 2124
YS_P._akitaorum_YW16_1______      TGAATTTATTCAATTGTCAATTTAGAAAACTTCTTGTCAGTCTGAGAACG 2125
YS_P._akitaorum_YW14_2______      TGAATTTATTCAATTGTCAATTTAGAAAACTTCTTGTCAGTCTGAGAACG 2125
YS_P._kawadai_YW12_1______        TGAATTTATTCAATTGTCAATTTAGAAAACTTCTTGTCAGTCTGAGAACG 2122
YS_P._takakuwai_YW13_1______      TGAATTTATTCAATTGTCAATTTAGAAAACTTCTTGTCAGTCTGAGAACG 2245
YS_P._albisomni_YW19_1______      TGAATTTATTCAATTGTCAATTTAGAAAACTTCTTGTCAGTCTGAGAACG 2123
YS_P._albisomni_YW21_1______      TGAATTTATTCAATTGTCAATTTAGAAAACTTCTTGTCAGTCTGAGAACG 2122
YS_P._albisomni_YW23_1______      TGAATTTATTCAATTGTCAATTTAGAAAACTTCTTGTCAGTCTGAGAACG 2120
YS_P._kawadai_YW03_1________      TGAATTTATTCAATTGTCAATTTAGAAAACTTCTTGTCAGTCTGAGAACG 2122
YS_P._takakuwai_YW54_1______      TGAATTTATTCAATTGTCAATTTAGAAAACTTCTTGTCAGTCTGAGAACG 2122
YS_P._takakuwai_YW73_3______      TGAATTTATTCAATTGTCAATTTAGAAAACTTCTTGTCAGTCTGAGAACG 2125
YS_P._acuticollis_YW07_8____      TGAATTTATTCAATTGTCAATTTAGAAAACTTCTTGTCAGTCTGAGAACG 2124
YS_P._albisomni_YW08_1______      TGAATTTATTCAATTGTCAATTTAGAAAACTTCTTGTCAGTCTGAGAACG 2123
YS_P._albisomni_YW09_1______      TGAATTTATTCAATTGTCAATTTAGAAAACTTCTTGTCAGTCTGAGAACG 2124
YS_P._takakuwai_YW38_1______      TGAATTTATTCAATTGTCAATTTAGAAAACTTCTTGTCAGTCTGAGAACG 2124
YS_P._angularis_YW25_8______      TGAATTTATTCAATTGTCAATTTAGAAAACTTCTTGTCAGTCTGAGAACG 2157
                                  ******************************************** *****
```

YS_P._viridicuprus_YW75_3___	TAAGTTCTTGGGCTGGTGATGAGACTGCTTTGCAGTCTTAAGATAGTTAT	2175
YS_P._viridicuprus_YW76_3___	TAAGTTCTTGGGCTGGTGATGAGACTGCTTTGCAGTCTTAAGATAGTTAT	2175
YS_P._viridicuprus_YW78_3___	TAAGTTCTTGGGCTGGTGATGAGACTGCTTTGCAGTCTTAAGATAGTTAT	2177
YS_P._viridicuprus_YW58_1___	TAAGTTCTTGGGCTGGTGATGAGACTGCTTTGCAGTCTTAAGATAGTTAT	2173
YS_P._viridicuprus_YW04_1___	TAAGTTCTTGGGCTGGTGATGAGACTGCTTTGCAGTCTTAAGATAGTTAT	2176
YS_P._hongwonpyoi_YW34_8____	TAAGTTCTTGGGCTGGTGATGAGACTGCTTTGCAGTCTTAAGATAGTTAT	2177
YS_P._hongwonpyoi_YW34_2a___	TAAGTTCTTGGGCTGGTGATGAGACTGCTTTGCAGTCTTAAGATAGTTAT	2179
YS_P._hongwonpyoi_YW33_8____	TAAGTTCTTGGGCTGGTGATGAGACTGCTTTGCAGTCTTAAGATAGTTAT	2174
YS_P._hongwonpyoi_YW24_1____	TAAGTTCTTGGGCTGGTGATGAGACTGCTTTGCAGTCTTAAGATAGTTAT	2176
YS_P._hongwonpyoi_YW05_8____	TAAGTTCTTGGGCTGGTGATGAGACTGCTTTGCAGTCTTAAGATAGTTAT	2176
YS_P._hongwonpyoi_YW35_8___	TAAGTTCTTGGGCTGGTGATGAGACTGCTTTGCAGTCTTAAGATAGTTAT	2176
YS_P._hongwonpyoi_YW37_8____	TAAGTTCTTGGGCTGGTGATGAGACTGCTTTGCAGTCTTAAGATAGTTAT	2178
YS_P._hongwonpyoi_YW36_1____	TAAGTTCTTGGGCTGGTGATGAGACTGCTTTGCAGTCTTAAGATAGTTAT	2178
YS_P._sue_YW45_1____________	TCAGTTCTTGGGCTGGTGATGAGACTGCTTTGCAGTCTTAAGATAGTTAT	2172
YS_P._sue_YW80_3____________	TCAGTTCTTGGGCTGGTGATGAGACTGCTTTGCAGTCTTAAGATAGTTAT	2172
YS_P._takakuwai_YW43_2______	TCAGTTCTTGGGCTGGTGATGAGACTGCTTTGCAGTCTTAAGATAGTTAT	2161
YS_P._urushiyamai_YW48_1____	TCAGTTCTTGG-CTGGTGATGAGACTGCTTTGCAGTCCTAAGATAGTTAT	2173
YS_P._urushiyamai_YW50_1____	TCAGTTCTTGG-CTGGTGATGAGACTGCTTTGCAGTCTTAAGATAGTTAT	2173
YS_P._urushiyamai_YW49_2____	TCAGTTCTTGG-CTGGTGATGAGACTGCTTTGCAGTCTTAAGATAGTTAT	2171
YS_P._delicatulus_YW65_2____	TCAGTTCTTGGGCTGGTGATGAGACTGCTTTGCAGTCTTAAGATAGTTAT	2181
YS_P._delicatulus_YW46_1____	TCAGTTCTTGGGCTGGTGATGAGACTGCTTTGCAGTCTTAAGATAGTTAT	2181
YS_P._delicatulus_YW10_1____	TCAGTTCTTGGGCTGGTGATGAGACTGCTTTGCAGTCTTAAGATAGTTAT	2181
YS_P._delicatulus_YW68_3____	TCAGTTCTTGGGCTGGTGATGAGACTGCTTTGCAGTCTTAAGATAGTTAT	2178
YS_P._delicatulus_YW72_2____	TCAGTTCTTGGGCTGGTGATGAGACTGCTTTGCAGTCTTAAGATAGTTAT	2179
YS_P._delicatulus_YW47_2____	TCAGTTCTTGGGCTGGTGATGAGACTGCTTTGCAGTCTTAAGATAGTTAT	2176
YS_P._takakuwai_YW88_1______	TCAGTTCTTGGGCTGGTGATGAGACTGCTTTGCAGTCTTAAGATAGTTAT	2174
YS_P._akitaorum_YW16_1______	TCAGTTCTTGGGCTGGTGATGAGACTGCTTTGCAGTCTTAAGATAGTTAT	2175
YS_P._akitaorum_YW14_2______	TCAGTTCTTGGGCTGGTGATGAGACTGCTTTGCAGTCTTAAGATAGTTAT	2175
YS_P._kawadai_YW12_1______	TCAGTTCTTGGGCTGGTGATGAGACTGCTTTGCAGTCTTAAGATAGTTAT	2172
YS_P._takakuwai_YW13_1______	TCAGTTCTTGGGCTGGTGATGAGACTGCTTTGCAGTCTTAAGATAGTTAT	2295
YS_P._albisomni_YW19_1______	TCAGTTCTTGGGCTGGTGATGAGACTGCTTTGCAGTCTTAAGATAGTTAT	2173
YS_P._albisomni_YW21_1______	TCAGTTCTTGGGCTGGTGATGAGACTGCTTTGCAGTCTTAAGATAGTTAT	2172
YS_P._albisomni_YW23_1______	TCAGTTCTTGGGCTGGTGATGAGACTGCTTTGCAGTCTTAAGATAGTTAT	2170
YS_P._kawadai_YW03_1________	TCAGTTCTTGGGCTGGTGATGAGACTGCTTTGCAGTCTTAAGATAGTTAT	2172
YS_P._takakuwai_YW54_1______	TCAGTTCTTGGGCTGGTGATGAGACTGCTTTGCAGTCTTAAGATAGTTAT	2172
YS_P._takakuwai_YW73_3______	TCAGTTCTTGGGCTGGTGATGGGACTGCTTTGCAGTCTTAAGATAGTTAT	2175

```
YS_P._acuticollis_YW07_8____        TCAGTTCTTGGGCTGGTGATGAGACTGCTTTGCAGTCTTAAGATAGTTAT 2174
YS_P._albisomni_YW08_1______        TCAGTTCTTGGGCTGGTGATGAGACTGCTTTGCAGTCTTAAGATAGTTAT 2173
YS_P._albisomni_YW09_1______        TCAGTTCTTGGGCTGGTGATGAGACTGCTTTGCAGTCTTAAGATAGTTAT 2174
YS_P._takakuwai_YW38_1______        TCAGTTCTTGGGCTGGTGATGAGACTGCTTTGCAGTCTTAAGATAGTTAT 2174
YS_P._angularis_YW25_8______        TCAGTTCTTGGGCTGGTGATGAGACTGCTTTGCAGTCTTAAGATAGTTAT 2207
                                    * ********* ********* *************** ************

YS_P._viridicuprus_YW75_3___        CTGGTTGATCCTGCCA 2191
YS_P._viridicuprus_YW76_3___        CTGGTTGATCCTGCCA 2191
YS_P._viridicuprus_YW78_3___        CTGGTTGATCCTGCCA 2193
YS_P._viridicuprus_YW58_1___        CTGGTTGATCCTGCCA 2189
YS_P._viridicuprus_YW04_1___        CTGGTTGATCCTGCCA 2192
YS_P._hongwonpyoi_YW34_8____        CTGGTTGATCCTGCCA 2193
YS_P._hongwonpyoi_YW34_2a___        CTGGTTGATCCTGCCA 2195
YS_P._hongwonpyoi_YW33_8____        CTGGTTGATCCTGCCA 2190
YS_P._hongwonpyoi_YW24_1____        CTGGTTGATCCTGCCA 2192
YS_P._hongwonpyoi_YW05_8____        CTGGTTGATCCTGCCA 2192
YS_P._hongwonpyoi_YW35_8___         CTGGTTGATCCTGCCA 2192
YS_P._hongwonpyoi_YW37_8____        CTGGTTGATCCTGCCA 2194
YS_P._hongwonpyoi_YW36_1____        CTGGTTGATCCTGCCA 2194
YS_P._sue_YW45_1____________        CTGGTTGATCCTGCCA 2188
YS_P._sue_YW80_3____________        CTGGTTGATCCTGCCA 2188
YS_P._takakuwai_YW43_2______        CTGGTTGATCCTGCCA 2177
YS_P._urushiyamai_YW48_1____        CTGGTTGATCCTGCCA 2189
YS_P._urushiyamai_YW50_1____        CTGGTTGATCCTGCCA 2189
YS_P._urushiyamai_YW49_2____        CTGGTTGATCCTGCCA 2187
YS_P._delicatulus_YW65_2____        CTGGTTGATCCTGCCA 2197
YS_P._delicatulus_YW46_1____        CTGGTTGATCCTGCCA 2197
YS_P._delicatulus_YW10_1____        CTGGTTGATCCTGCCA 2197
YS_P._delicatulus_YW68_3____        CTGGTTGATCCTGCCA 2194
YS_P._delicatulus_YW72_2____        CTGGTTGATCCTGCCA 2195
YS_P._delicatulus_YW47_2____        CTGGTTGATCCTGCCA 2192
YS_P._takakuwai_YW88_1______        CTGGTTGATCCTGCCA 2190
YS_P._akitaorum_YW16_1______        CTGGTTGATCCTGCCA 2191
YS_P._akitaorum_YW14_2______        CTGGTTGATCCTGCCA 2191
YS_P._kawadai_YW12_1______          CTGGTTGATCCTGCCA 2188
```

```
YS_P._takakuwai_YW13_1______      CTGGTTGATCCTGCCA 2311
YS_P._albisomni_YW19_1______      CTGGTTGATCCTGCCA 2189
YS_P._albisomni_YW21_1______      CTGGTTGATCCTGCCA 2188
YS_P._albisomni_YW23_1______      CTGGTTGATCCTGCCA 2186
YS_P._kawadai_YW03_1________      CTGGTTGATCCTGCCA 2188
YS_P._takakuwai_YW54_1______      CTGGTTGATCCTGCCA 2188
YS_P._takakuwai_YW73_3______      CTGGTTGATCCTGCCA 2191
YS_P._acuticollis_YW07_8____      CTGGTTGATCCTGCCA 2190
YS_P._albisomni_YW08_1______      CTGGTTGATCCTGCCA 2189
YS_P._albisomni_YW09_1______      CTGGTTGATCCTGCCA 2190
YS_P._takakuwai_YW38_1______      CTGGTTGATCCTGCCA 2190
YS_P._angularis_YW25_8______      CTGGTTGATCCTGCCA 2223
                                  ****************
```
