# Supplementary material for: Evolutionary Relationship Between Platycerus Stag Beetles and Their Mycangium-Associated Yeast Symbionts
Source: Front Microbiol. 2020 Jun 30;11:1436. doi: 10.3389/fmicb.2020.01436 (PMC7338584; doi:10.3389/fmicb.2020.01436)
Supplement: Supplementary file 8 [file Data_Sheet_8.PDF]

SI Appendix 3. The sequence alignment used for the ITS + IGS phylogenetic analysis using ClustalW.

```
YS_P._acuticollis_YW07_8____        GGAAGTAAAAGTCGTAACAAGGTTTCCGTAGGTGAACCTGCGGAAGGATC 50
YS_P._takakuwai_YW54_1____          GGAAGTAAAAGTCGTAACAAGGTTTCCGTAGGTGAACCTGCGGAAGGATC 50
YS_P._albisomni_YW19_1____          GGAAGTAAAAGTCGTAACAAGGTTTCCGTAGGTGAACCTGCGGAAGGATC 50
YS_P._kawadai_YW53_1____            GGAAGTAAAAGTCGTAACAAGGTTTCCGTAGGTGAACCTGCGGAAGGATC 50
YS_P._kawadai_YW18_1____            GGAAGTAAAAGTCGTAACAAGGTTTCCGTAGGTGAACCTGCGGAAGGATC 50
YS_P._sue_YW45_1____                GGAAGTAAAAGTCGTAACAAGGTTTCCGTAGGTGAACCTGCGGAAGGATC 50
YS_P._sue_YW80_3____                GGAAGTAAAAGTCGTAACAAGGTTTCCGTAGGTGAACCTGCGGAAGGATC 50
YS_P._sugitai_YW44_1____            GGAAGTAAAAGTCGTAACAAGGTTTCCGTAGGTGAACCTGCGGAAGGATC 50
YS_P._urushiyamai_YW50_1____        GGAAGTAAAAGTCGTAACAAGGTTTCCGTAGGTGAACCTGCGGAAGGATC 50
YS_P._delicatulus_YW52_1____        GGAAGTAAAAGTCGTAACAAGGTTTCCGTAGGTGAACCTGCGGAAGGATC 50
YS_P._delicatulus_YW60_2____        GGAAGTAAAAGTCGTAACAAGGTTTCCGTAGGTGAACCTGCGGAAGGATC 50
YS_P._akitaorum_YW16_1____          GGAAGTAAAAGTCGTAACAAGGTTTCCGTAGGTGAACCTGCGGAAGGATC 50
YS_P._hongwonpyoi_YW05_8____        GGAAGTAAAAGTCGTAACAAGGTTTCCGTAGGTGAACCTGCGGAAGGATC 50
YS_P._hongwonpyoi_YW24_1____        GGAAGTAAAAGTCGTAACAAGGTTTCCGTAGGTGAACCTGCGGAAGGATC 50
YS_P._hongwonpyoi_YW33_8____        GGAAGTAAAAGTCGTAACAAGGTTTCCGTAGGTGAACCTGCGGAAGGATC 50
YS_P._hongwonpyoi_YW35_8____        GGAAGTAAAAGTCGTAACAAGGTTTCCGTAGGTGAACCTGCGGAAGGATC 50
YS_P._hongwonpyoi_YW37_8____        GGAAGTAAAAGTCGTAACAAGGTTTCCGTAGGTGAACCTGCGGAAGGATC 50
YS_P._hongwonpyoi_YW34_8____        GGAAGTAAAAGTCGTAACAAGGTTTCCGTAGGTGAACCTGCGGAAGGATC 50
YS_P._hongwonpyoi_YW34_2a___        GGAAGTAAAAGTCGTAACAAGGTTTCCGTAGGTGAACCTGCGGAAGGATC 50
YS_P._viridicuprus_YW15_1____       GGAAGTAAAAGTCGTAACAAGGTTTCCGTAGGTGAACCTGCGGAAGGATC 50
YS_P._hongwonpyoi_YW36_1____        GGAAGTAAAAGTCGTAACAAGGTTTCCGTAGGTGAACCTGCGGAAGGATC 50
YS_P._angularis_YW25_8___           GGAAGTAAAAGTCGTAACAAGGTTTCCGTAGGTGAACCTGCGGAAGGATC 50
                                    **************************************************

YS_P._acuticollis_YW07_8____        ATTACAGTATTCTTTTTGCCAGCGCTTAACTGCGCGGCGAAAAAACCTTA 100
YS_P._takakuwai_YW54_1____          ATTACAGTATTCTTTTTGCCAGCGCTTAACTGCGCGGCGAAAAAACCTTA 100
YS_P._albisomni_YW19_1____          ATTACAGTATTCTTTTTGCCAGCGCTTAACTGCGCGGCGAAAAAACCTTA 100
YS_P._kawadai_YW53_1____            ATTACAGTATTCTTTTTGCCAGCGCTTAACTGCGCGGCGAAAAAACCTTA 100
YS_P._kawadai_YW18_1____            ATTACAGTATTCTTTTTGCCAGCGCTTAACTGCGCGGCGAAAAAACCTTA 100
YS_P._sue_YW45_1____                ATTACAGTATTCTTTTTGCCAGCGCTTAACTGCGCGGCGAAAAAACCTTA 100
YS_P._sue_YW80_3____                ATTACAGTATTCTTTTTGCCAGCGCTTAACTGCGCGGCGAAAAAACCTTA 100
YS_P._sugitai_YW44_1____            ATTACAGTATTCTTTTTGCCAGCGCTTAACTGCGCGGCGAAAAAACCTTA 100
YS_P._urushiyamai_YW50_1____        ATTACAGTATTCTTTTTGCCAGCGCTTAACTGCGCGGCGAAAAAACCTTA 100
YS_P._delicatulus_YW52_1____        ATTACAGTATTCTTTTTGCCAGCGCTTAACTGCGCGGCGAAAAAACCTTA 100
```

```
YS_P._delicatulus_YW60_2____        ATTACAGTATTCTTTTTGCCAGCGCTTAACTGCGCGGCGAAAAAACCTTA 100
YS_P._akitaorum_YW16_1____          ATTACAGTATTCTTTTTGCCAGCGCTTAACTGCGCGGCGAAAAAACCTTA 100
YS_P._hongwonpyoi_YW05_8____        ATTACAGTATTCTTTTTGCCAGCGCTTAACTGCGCGGCGAAAAAACCTTA 100
YS_P._hongwonpyoi_YW24_1____        ATTACAGTATTCTTTTTGCCAGCGCTTAACTGCGCGGCGAAAAAACCTTA 100
YS_P._hongwonpyoi_YW33_8____        ATTACAGTATTCTTTTTGCCAGCGCTTAACTGCGCGGCGAAAAAACCTTA 100
YS_P._hongwonpyoi_YW35_8____        ATTACAGTATTCTTTTTGCCAGCGCTTAACTGCGCGGCGAAAAAACCTTA 100
YS_P._hongwonpyoi_YW37_8____        ATTACAGTATTCTTTTTGCCAGCGCTTAACTGCGCGGCGAAAAAACCTTA 100
YS_P._hongwonpyoi_YW34_8____        ATTACAGTATTCTTTTTGCCAGCGCTTAACTGCGCGGCGAAAAAACCTTA 100
YS_P._hongwonpyoi_YW34_2a___        ATTACAGTATTCTTTTTGCCAGCGCTTAACTGCGCGGCGAAAAAACCTTA 100
YS_P._viridicuprus_YW15_1____       ATTACAGTATTCTTTTTGCCAGCGCTTAACTGCGCGGCGAAAAAACCTTA 100
YS_P._hongwonpyoi_YW36_1____        ATTACAGTATTCTTTTTGCCAGCGCTTAACTGCGCGGCGAAAAAACCTTA 100
YS_P._angularis_YW25_8___           ATTACAGTATTCTTTTTGCCAGCGCTTAACTGCGCGGCGAAAAAACCTTA 100
                                    **************************************************

YS_P._acuticollis_YW07_8____        CACACAGTGTTTTCTTTATTAGAAACTATTGCTTTGGTTTGGCTCAGAAA 150
YS_P._takakuwai_YW54_1____          CACACAGTGTTTTCTTTATTAGAAACTATTGCTTTGGTTTGGCTCAGAAA 150
YS_P._albisomni_YW19_1____          CACACAGTGTTTTCTTTATTAGAAACTATTGCTTTGGTTTGGCTCAGAAA 150
YS_P._kawadai_YW53_1____            CACACAGTGTTTTCTTTATTAGAAACTATTGCTTTGGTTTGGCTCAGAAA 150
YS_P._kawadai_YW18_1____            CACACAGTGTTTTCTTTATTAGAAACTATTGCTTTGGTTTGGCTCAGAAA 150
YS_P._sue_YW45_1____                CACACAGTGTTTTCTTTATTAGAAACTATTGCTTTGGTTTGGCTCAGAAA 150
YS_P._sue_YW80_3____                CACACAGTGTTTTCTTTATTAGAAACTATTGCTTTGGTTTGGCTCAGAAA 150
YS_P._sugitai_YW44_1____            CACACAGTGTTTTCTTTATTAGAAACTATTGCTTTGGTTTGGCTCAGAAA 150
YS_P._urushiyamai_YW50_1____        CACACAGTGTTTTCTTTATTAGAAACTATTGCTTTGGTTTGGCTCAGAAA 150
YS_P._delicatulus_YW52_1____        CACACAGTGTTTTCTTTATTAGAAACTATTGCTTTGGTTTGGCTCAGAAA 150
YS_P._delicatulus_YW60_2____        CACACAGTGTTTTCTTTATTAGAAACTATTGCTTTGGTTTGGCTCAGAAA 150
YS_P._akitaorum_YW16_1____          CACACAGTGTTTTCTTTATTAGAAACTATTGCTTTGGTTTGGCTCAGAAA 150
YS_P._hongwonpyoi_YW05_8____        CACACAGTGTTTTCTTTATTAGAAACTATTGCTTTGGTTTGGCTCAGAAA 150
YS_P._hongwonpyoi_YW24_1____        CACACAGTGTTTTCTTTATTAGAAACTATTGCTTTGGTTTGGCTCAGAAA 150
YS_P._hongwonpyoi_YW33_8____        CACACAGTGTTTTCTTTATTAGAAACTATTGCTTTGGTTTGGCTCAGAAA 150
YS_P._hongwonpyoi_YW35_8____        CACACAGTGTTTTCTTTATTAGAAACTATTGCTTTGGTTTGGCTCAGAAA 150
YS_P._hongwonpyoi_YW37_8____        CACACAGTGTTTTCTTTATTAGAAACTATTGCTTTGGTTTGGCTCAGAAA 150
YS_P._hongwonpyoi_YW34_8____        CACACAGTGTTTTCTTTATTAGAAACTATTGCTTTGGTTTGGCTCAGAAA 150
YS_P._hongwonpyoi_YW34_2a___        CACACAGTGTTTTCTTTATTAGAAACTATTGCTTTGGTTTGGCTCAGAAA 150
YS_P._viridicuprus_YW15_1____       CACACAGTGTTTTCTTTATTAGAAACTATTGCTTTGGTTTGGCTCAGAAA 150
YS_P._hongwonpyoi_YW36_1____        CACACAGTGTTTTCTTTATTAGAAACTATTGCTTTGGTTTGGCTCAGAAA 150
YS_P._angularis_YW25_8___           CACACAGTGTTTTCTTTATTAGAAACTATTGCTTTGGTTTGGCTCAGAAA 150
```

```
                                **************************************************

YS_P._acuticollis_YW07_8____    TGAGTTGGGCCAGAGGTTTACCAAACTTCAATTTTATTGAATTGTTATTT 200
YS_P._takakuwai_YW54_1____      TGAGTTGGGCCAGAGGTTTACCAAACTTCAATTTTATTGAATTGTTATTT 200
YS_P._albisomni_YW19_1____      TGAGTTGGGCCAGAGGTTTACCAAACTTCAATTTTATTGAATTGTTATTT 200
YS_P._kawadai_YW53_1____        TGAGTTGGGCCAGAGGTTTACCAAACTTCAATTTTATTGAATTGTTATTT 200
YS_P._kawadai_YW18_1____        TGAGTTGGGCCAGAGGTTTACCAAACTTCAATTTTATTGAATTGTTATTT 200
YS_P._sue_YW45_1____            TGAGTTGGGCCAGAGGTTTACCAAACTTCAATTTTATTGAATTGTTATTT 200
YS_P._sue_YW80_3____            TGAGTTGGGCCAGAGGTTTACCAAACTTCAATTTTATTGAATTGTTATTT 200
YS_P._sugitai_YW44_1____        TGAGTTGGGCCAGAGGTTTACCAAACTTCAATTTTATTGAATTGTTATTT 200
YS_P._urushiyamai_YW50_1____    TGAGTTGGGCCAGAGGTTTACCAAACTTCAATTTTATTGAATTGTTATTT 200
YS_P._delicatulus_YW52_1____    TGAGTTGGGCCAGAGGTTTACCAAACTTCAATTTTATTGAATTGTTATTT 200
YS_P._delicatulus_YW60_2____    TGAGTTGGGCCAGAGGTTTACCAAACTTCAATTTTATTGAATTGTTATTT 200
YS_P._akitaorum_YW16_1____      TGAGTTGGGCCAGAGGTTTACCAAACTTCAATTTTATTGAATTGTTATTT 200
YS_P._hongwonpyoi_YW05_8____    TGAGTTGGGCCAGAGGTTTACCAAACTTCAATTTTATTGAATTGTTATTT 200
YS_P._hongwonpyoi_YW24_1____    TGAGTTGGGCCAGAGGTTTACCAAACTTCAATTTTATTGAATTGTTATTT 200
YS_P._hongwonpyoi_YW33_8____    TGAGTTGGGCCAGAGGTTTACCAAACTTCAATTTTATTGAATTGTTATTT 200
YS_P._hongwonpyoi_YW35_8____    TGAGTTGGGCCAGAGGTTTACCAAACTTCAATTTTATTGAATTGTTATTT 200
YS_P._hongwonpyoi_YW37_8____    TGAGTTGGGCCAGAGGTTTACCAAACTTCAATTTTATTGAATTGTTATTT 200
YS_P._hongwonpyoi_YW34_8____    TGAGTTGGGCCAGAGGTTTACCAAACTTCAATTTTATTGAATTGTTATTT 200
YS_P._hongwonpyoi_YW34_2a___    TGAGTTGGGCCAGAGGTTTACCAAACTTCAATTTTATTGAATTGTTATTT 200
YS_P._viridicuprus_YW15_1____   TGAGTTGGGCCAGAGGTTTACCAAACTTCAATTTTATTGAATTGTTATTT 200
YS_P._hongwonpyoi_YW36_1____    TGAGTTGGGCCAGAGGTTTACCAAACTTCAATTTTATTGAATTGTTATTT 200
YS_P._angularis_YW25_8___       TGAGTTGGGCCAGAGGTTTACCAAACTTCAATTTTATTGAATTGTTATTT 200
                                **************************************************

YS_P._acuticollis_YW07_8____    TATTAATTTGTCAATTTGTTGATTAAATTCAAAAATCTTCAAAACTTTCA 250
YS_P._takakuwai_YW54_1____      TATTAATTTGTCAATTTGTTGATTAAATTCAAAAATCTTCAAAACTTTCA 250
YS_P._albisomni_YW19_1____      TATTAATTTGTCAATTTGTTGATTAAATTCAAAAATCTTCAAAACTTTCA 250
YS_P._kawadai_YW53_1____        TATTAATTTGTCAATTTGTTGATTAAATTCAAAAATCTTCAAAACTTTCA 250
YS_P._kawadai_YW18_1____        TATTAATTTGTCAATTTGTTGATTAAATTCAAAAATCTTCAAAACTTTCA 250
YS_P._sue_YW45_1____            TATTAATTTGTCAATTTGTTGATTAAATTCAAAAATCTTCAAAACTTTCA 250
YS_P._sue_YW80_3____            TATTAATTTGTCAATTTGTTGATTAAATTCAAAAATCTTCAAAACTTTCA 250
YS_P._sugitai_YW44_1____        TATTAATTTGTCAATTTGTTGATTAAATTCAAAAATCTTCAAAACTTTCA 250
YS_P._urushiyamai_YW50_1____    TATTAATTTGTCAATTTGTTGATTAAATTCAAAAATCTTCAAAACTTTCA 250
YS_P._delicatulus_YW52_1____    TATTAATTTGTCAATTTGTTGATTAAATTCAAAAATCTTCAAAACTTTCA 250
```

```
YS_P._delicatulus_YW60_2____        TATTAATTTGTCAATTTGTTGATTAAATTCAAAAATCTTCAAAACTTTCA 250
YS_P._akitaorum_YW16_1____          TATTAATTTGTCAATTTGTTGATTAAATTCAAAAATCTTCAAAACTTTCA 250
YS_P._hongwonpyoi_YW05_8____        TATTAATTTGTCAATTTGTTGATTAAATTCAAAAATCTTCAAAACTTTCA 250
YS_P._hongwonpyoi_YW24_1____        TATTAATTTGTCAATTTGTTGATTAAATTCAAAAATCTTCAAAACTTTCA 250
YS_P._hongwonpyoi_YW33_8____        TATTAATTTGTCAATTTGTTGATTAAATTCAAAAATCTTCAAAACTTTCA 250
YS_P._hongwonpyoi_YW35_8____        TATTAATTTGTCAATTTGTTGATTAAATTCAAAAATCTTCAAAACTTTCA 250
YS_P._hongwonpyoi_YW37_8____        TATTAATTTGTCAATTTGTTGATTAAATTCAAAAATCTTCAAAACTTTCA 250
YS_P._hongwonpyoi_YW34_8____        TATTAATTTGTCAATTTGTTGATTAAATTCAAAAATCTTCAAAACTTTCA 250
YS_P._hongwonpyoi_YW34_2a___        TATTAATTTGTCAATTTGTTGATTAAATTCAAAAATCTTCAAAACTTTCA 250
YS_P._viridicuprus_YW15_1____       TATTAATTTGTCAATTTGTTGATTAAATTCAAAAATCTTCAAAACTTTCA 250
YS_P._hongwonpyoi_YW36_1____        TATTAATTTGTCAATTTGTTGATTAAATTCAAAAATCTTCAAAACTTTCA 250
YS_P._angularis_YW25_8___           TATTAATTTGTCAATTTGTTGATTAAATTCAAAAATCTTCAAAACTTTCA 250
                                    **************************************************

YS_P._acuticollis_YW07_8____        ACAACGGATCTCTTGGTTCTCGCATCGATGAAGAACGCAGCGAAATGCGA 300
YS_P._takakuwai_YW54_1____          ACAACGGATCTCTTGGTTCTCGCATCGATGAAGAACGCAGCGAAATGCGA 300
YS_P._albisomni_YW19_1____          ACAACGGATCTCTTGGTTCTCGCATCGATGAAGAACGCAGCGAAATGCGA 300
YS_P._kawadai_YW53_1____            ACAACGGATCTCTTGGTTCTCGCATCGATGAAGAACGCAGCGAAATGCGA 300
YS_P._kawadai_YW18_1____            ACAACGGATCTCTTGGTTCTCGCATCGATGAAGAACGCAGCGAAATGCGA 300
YS_P._sue_YW45_1____                ACAACGGATCTCTTGGTTCTCGCATCGATGAAGAACGCAGCGAAATGCGA 300
YS_P._sue_YW80_3____                ACAACGGATCTCTTGGTTCTCGCATCGATGAAGAACGCAGCGAAATGCGA 300
YS_P._sugitai_YW44_1____            ACAACGGATCTCTTGGTTCTCGCATCGATGAAGAACGCAGCGAAATGCGA 300
YS_P._urushiyamai_YW50_1____        ACAACGGATCTCTTGGTTCTCGCATCGATGAAGAACGCAGCGAAATGCGA 300
YS_P._delicatulus_YW52_1____        ACAACGGATCTCTTGGTTCTCGCATCGATGAAGAACGCAGCGAAATGCGA 300
YS_P._delicatulus_YW60_2____        ACAACGGATCTCTTGGTTCTCGCATCGATGAAGAACGCAGCGAAATGCGA 300
YS_P._akitaorum_YW16_1____          ACAACGGATCTCTTGGTTCTCGCATCGATGAAGAACGCAGCGAAATGCGA 300
YS_P._hongwonpyoi_YW05_8____        ACAACGGATCTCTTGGTTCTCGCATCGATGAAGAACGCAGCGAAATGCGA 300
YS_P._hongwonpyoi_YW24_1____        ACAACGGATCTCTTGGTTCTCGCATCGATGAAGAACGCAGCGAAATGCGA 300
YS_P._hongwonpyoi_YW33_8____        ACAACGGATCTCTTGGTTCTCGCATCGATGAAGAACGCAGCGAAATGCGA 300
YS_P._hongwonpyoi_YW35_8____        ACAACGGATCTCTTGGTTCTCGCATCGATGAAGAACGCAGCGAAATGCGA 300
YS_P._hongwonpyoi_YW37_8____        ACAACGGATCTCTTGGTTCTCGCATCGATGAAGAACGCAGCGAAATGCGA 300
YS_P._hongwonpyoi_YW34_8____        ACAACGGATCTCTTGGTTCTCGCATCGATGAAGAACGCAGCGAAATGCGA 300
YS_P._hongwonpyoi_YW34_2a___        ACAACGGATCTCTTGGTTCTCGCATCGATGAAGAACGCAGCGAAATGCGA 300
YS_P._viridicuprus_YW15_1____       ACAACGGATCTCTTGGTTCTCGCATCGATGAAGAACGCAGCGAAATGCGA 300
YS_P._hongwonpyoi_YW36_1____        ACAACGGATCTCTTGGTTCTCGCATCGATGAAGAACGCAGCGAAATGCGA 300
YS_P._angularis_YW25_8___           ACAACGGATCTCTTGGTTCTCGCATCGATGAAGAACGCAGCGAAATGCGA 300
```

```
                                  **************************************************

YS_P._acuticollis_YW07_8____      TAAGTAATATGAATTGCAGATTTTCGTGAATCATCGAATCTTTGAACGCA 350
YS_P._takakuwai_YW54_1____        TAAGTAATATGAATTGCAGATTTTCGTGAATCATCGAATCTTTGAACGCA 350
YS_P._albisomni_YW19_1____        TAAGTAATATGAATTGCAGATTTTCGTGAATCATCGAATCTTTGAACGCA 350
YS_P._kawadai_YW53_1____          TAAGTAATATGAATTGCAGATTTTCGTGAATCATCGAATCTTTGAACGCA 350
YS_P._kawadai_YW18_1____          TAAGTAATATGAATTGCAGATTTTCGTGAATCATCGAATCTTTGAACGCA 350
YS_P._sue_YW45_1____              TAAGTAATATGAATTGCAGATTTTCGTGAATCATCGAATCTTTGAACGCA 350
YS_P._sue_YW80_3____              TAAGTAATATGAATTGCAGATTTTCGTGAATCATCGAATCTTTGAACGCA 350
YS_P._sugitai_YW44_1____          TAAGTAATATGAATTGCAGATTTTCGTGAATCATCGAATCTTTGAACGCA 350
YS_P._urushiyamai_YW50_1____      TAAGTAATATGAATTGCAGATTTTCGTGAATCATCGAATCTTTGAACGCA 350
YS_P._delicatulus_YW52_1____      TAAGTAATATGAATTGCAGATTTTCGTGAATCATCGAATCTTTGAACGCA 350
YS_P._delicatulus_YW60_2____      TAAGTAATATGAATTGCAGATTTTCGTGAATCATCGAATCTTTGAACGCA 350
YS_P._akitaorum_YW16_1____        TAAGTAATATGAATTGCAGATTTTCGTGAATCATCGAATCTTTGAACGCA 350
YS_P._hongwonpyoi_YW05_8____      TAAGTAATATGAATTGCAGATTTTCGTGAATCATCGAATCTTTGAACGCA 350
YS_P._hongwonpyoi_YW24_1____      TAAGTAATATGAATTGCAGATTTTCGTGAATCATCGAATCTTTGAACGCA 350
YS_P._hongwonpyoi_YW33_8____      TAAGTAATATGAATTGCAGATTTTCGTGAATCATCGAATCTTTGAACGCA 350
YS_P._hongwonpyoi_YW35_8____      TAAGTAATATGAATTGCAGATTTTCGTGAATCATCGAATCTTTGAACGCA 350
YS_P._hongwonpyoi_YW37_8____      TAAGTAATATGAATTGCAGATTTTCGTGAATCATCGAATCTTTGAACGCA 350
YS_P._hongwonpyoi_YW34_8____      TAAGTAATATGAATTGCAGATTTTCGTGAATCATCGAATCTTTGAACGCA 350
YS_P._hongwonpyoi_YW34_2a___      TAAGTAATATGAATTGCAGATTTTCGTGAATCATCGAATCTTTGAACGCA 350
YS_P._viridicuprus_YW15_1____     TAAGTAATATGAATTGCAGATTTTCGTGAATCATCGAATCTTTGAACGCA 350
YS_P._hongwonpyoi_YW36_1____      TAAGTAATATGAATTGCAGATTTTCGTGAATCATCGAATCTTTGAACGCA 350
YS_P._angularis_YW25_8___         TAAGTAATATGAATTGCAGATTTTCGTGAATCATCGAATCTTTGAACGCA 350
                                  **************************************************

YS_P._acuticollis_YW07_8____      CATTGCGCCCTTTGGTATTCCAAAGGGCATGCCTGTTTGAGCGTCATTTC 400
YS_P._takakuwai_YW54_1____        CATTGCGCCCTTTGGTATTCCAAAGGGCATGCCTGTTTGAGCGTCATTTC 400
YS_P._albisomni_YW19_1____        CATTGCGCCCTTTGGTATTCCAAAGGGCATGCCTGTTTGAGCGTCATTTC 400
YS_P._kawadai_YW53_1____          CATTGCGCCCTTTGGTATTCCAAAGGGCATGCCTGTTTGAGCGTCATTTC 400
YS_P._kawadai_YW18_1____          CATTGCGCCCTTTGGTATTCCAAAGGGCATGCCTGTTTGAGCGTCATTTC 400
YS_P._sue_YW45_1____              CATTGCGCCCTTTGGTATTCCAAAGGGCATGCCTGTTTGAGCGTCATTTC 400
YS_P._sue_YW80_3____              CATTGCGCCCTTTGGTATTCCAAAGGGCATGCCTGTTTGAGCGTCATTTC 400
YS_P._sugitai_YW44_1____          CATTGCGCCCTTTGGTATTCCAAAGGGCATGCCTGTTTGAGCGTCATTTC 400
YS_P._urushiyamai_YW50_1____      CATTGCGCCCTTTGGTATTCCAAAGGGCATGCCTGTTTGAGCGTCATTTC 400
YS_P._delicatulus_YW52_1____      CATTGCGCCCTTTGGTATTCCAAAGGGCATGCCTGTTTGAGCGTCATTTC 400
```

```
YS_P._delicatulus_YW60_2____      CATTGCGCCCTTTGGTATTCCAAAGGGCATGCCTGTTTGAGCGTCATTTC 400
YS_P._akitaorum_YW16_1____        CATTGCGCCCTTTGGTATTCCAAAGGGCATGCCTGTTTGAGCGTCATTTC 400
YS_P._hongwonpyoi_YW05_8____      CATTGCGCCCTTTGGTATTCCAAAGGGCATGCCTGTTTGAGCGTCATTTC 400
YS_P._hongwonpyoi_YW24_1____      CATTGCGCCCTTTGGTATTCCAAAGGGCATGCCTGTTTGAGCGTCATTTC 400
YS_P._hongwonpyoi_YW33_8____      CATTGCGCCCTTTGGTATTCCAAAGGGCATGCCTGTTTGAGCGTCATTTC 400
YS_P._hongwonpyoi_YW35_8____      CATTGCGCCCTTTGGTATTCCAAAGGGCATGCCTGTTTGAGCGTCATTTC 400
YS_P._hongwonpyoi_YW37_8____      CATTGCGCCCTTTGGTATTCCAAAGGGCATGCCTGTTTGAGCGTCATTTC 400
YS_P._hongwonpyoi_YW34_8____      CATTGCGCCCTTTGGTATTCCAAAGGGCATGCCTGTTTGAGCGTCATTTC 400
YS_P._hongwonpyoi_YW34_2a___      CATTGCGCCCTTTGGTATTCCAAAGGGCATGCCTGTTTGAGCGTCATTTC 400
YS_P._viridicuprus_YW15_1____     CATTGCGCCCTTTGGTATTCCAAAGGGCATGCCTGTTTGAGCGTCATTTC 400
YS_P._hongwonpyoi_YW36_1____      CATTGCGCCCTTTGGTATTCCAAAGGGCATGCCTGTTTGAGCGTCATTTC 400
YS_P._angularis_YW25_8___         CATTGCGCCCTTTGGTATTCCAAAGGGCATGCCTGTTTGAGCGTCATTTC 400
                                  **************************************************

YS_P._acuticollis_YW07_8____      TCTCTCAAACCCTCGGGTTTGGTATTGAGTGATACTCTTAGTTGAACTAG 450
YS_P._takakuwai_YW54_1____        TCTCTCAAACCCTCGGGTTTGGTATTGAGTGATACTCTTAGTTGAACTAG 450
YS_P._albisomni_YW19_1____        TCTCTCAAACCCTCGGGTTTGGTATTGAGTGATACTCTTAGTTGAACTAG 450
YS_P._kawadai_YW53_1____          TCTCTCAAACCCTCGGGTTTGGTATTGAGTGATACTCTTAGTTGAACTAG 450
YS_P._kawadai_YW18_1____          TCTCTCAAACCCTCGGGTTTGGTATTGAGTGATACTCTTAGTTGAACTAG 450
YS_P._sue_YW45_1____              TCTCTCAAACCCTCGGGTTTGGTATTGAGTGATACTCTTAGTTGAACTAG 450
YS_P._sue_YW80_3____              TCTCTCAAACCCTCGGGTTTGGTATTGAGTGATACTCTTAGTTGAACTAG 450
YS_P._sugitai_YW44_1____          TCTCTCAAACCCTCGGGTTTGGTATTGAGTGATACTCTTAGTTGAACTAG 450
YS_P._urushiyamai_YW50_1____      TCTCTCAAACCCTCGGGTTTGGTATTGAGTGATACTCTTAGTTGAACTAG 450
YS_P._delicatulus_YW52_1____      TCTCTCAAACCCTCGGGTTTGGTATTGAGTGATACTCTTAGTTGAACTAG 450
YS_P._delicatulus_YW60_2____      TCTCTCAAACCCTCGGGTTTGGTATTGAGTGATACTCTTAGTTGAACTAG 450
YS_P._akitaorum_YW16_1____        TCTCTCAAACCCTCGGGTTTGGTATTGAGTGATACTCTTAGTTGAACTAG 450
YS_P._hongwonpyoi_YW05_8____      TCTCTCAAACCCTCGGGTTTGGTATTGAGTGATACTCTTAGTTGAACTAG 450
YS_P._hongwonpyoi_YW24_1____      TCTCTCAAACCCTCGGGTTTGGTATTGAGTGATACTCTTAGTTGAACTAG 450
YS_P._hongwonpyoi_YW33_8____      TCTCTCAAACCCTCGGGTTTGGTATTGAGTGATACTCTTAGTTGAACTAG 450
YS_P._hongwonpyoi_YW35_8____      TCTCTCAAACCCTCGGGTTTGGTATTGAGTGATACTCTTAGTTGAACTAG 450
YS_P._hongwonpyoi_YW37_8____      TCTCTCAAACCCTCGGGTTTGGTATTGAGTGATACTCTTAGTTGAACTAG 450
YS_P._hongwonpyoi_YW34_8____      TCTCTCAAACCCTCGGGTTTGGTATTGAGTGATACTCTTAGTTGAACTAG 450
YS_P._hongwonpyoi_YW34_2a___      TCTCTCAAACCCTCGGGTTTGGTATTGAGTGATACTCTTAGTTGAACTAG 450
YS_P._viridicuprus_YW15_1____     TCTCTCAAACCCTCGGGTTTGGTATTGAGTGATACTCTTAGTTGAACTAG 450
YS_P._hongwonpyoi_YW36_1____      TCTCTCAAACCCTCGGGTTTGGTATTGAGTGATACTCTTAGTTGAACTAG 450
YS_P._angularis_YW25_8___         TCTCTCAAACCCTCGGGTTTGGTATTGAGTGATACTCTTAGTC-AACTAG 449
```

```
                                  ******************************************  ******

YS_P._acuticollis_YW07_8____      GCGTTTGCTTGAAAAGTATTGGCACGAGTGGTACTAAATAGTACTGACAG 500
YS_P._takakuwai_YW54_1____        GCGTTTGCTTGAAAAGTATTGGCACGAGTGGTACTAAATAGTACTGACAG 500
YS_P._albisomni_YW19_1____        GCGTTTGCTTGAAAAGTATTGGCACGAGTGGTACTAAATAGTACTGACAG 500
YS_P._kawadai_YW53_1____          GCGTTTGCTTGAAAAGTATTGGCACGAGTGGTACTAAATAGTACTGACAG 500
YS_P._kawadai_YW18_1____          GCGTTTGCTTGAAAAGTATTGGCACGAGTGGTACTAAATAGTACTGACAG 500
YS_P._sue_YW45_1____              GCGTTTGCTTGAAAAGTATTGGCACGAGTGGTACTAAATAGTACTGACAG 500
YS_P._sue_YW80_3____              GCGTTTGCTTGAAAAGTATTGGCACGAGTGGTACTAAATAGTACTGACAG 500
YS_P._sugitai_YW44_1____          GCGTTTGCTTGAAAAGTATTGGCACGAGTGGTACTAAATAGTACTGACAG 500
YS_P._urushiyamai_YW50_1____      GCGTTTGCTTGAAAAGTATTGGCACGAGTGGTACTAAATAGTACTGACAG 500
YS_P._delicatulus_YW52_1____      GCGTTTGCTTGAAAAGTATTGGCACGAGTGGTACTAAATAGTACTGACAG 500
YS_P._delicatulus_YW60_2____      GCGTTTGCTTGAAAAGTATTGGCACGAGTGGTACTAAATAGTACTGACAG 500
YS_P._akitaorum_YW16_1____        GCGTTTGCTTGAAAAGTATTGGCACGAGTGGTACTAAATAGTACTGACAG 500
YS_P._hongwonpyoi_YW05_8____      GCGTTTGCTTGAAAAGTATTGGCACGAGTGGTACTAAATAGTACTGACAG 500
YS_P._hongwonpyoi_YW24_1____      GCGTTTGCTTGAAAAGTATTGGCACGAGTGGTACTAAATAGTACTGACAG 500
YS_P._hongwonpyoi_YW33_8____      GCGTTTGCTTGAAAAGTATTGGCACGAGTGGTACTAAATAGTACTGACAG 500
YS_P._hongwonpyoi_YW35_8____      GCGTTTGCTTGAAAAGTATTGGCACGAGTGGTACTAAATAGTACTGACAG 500
YS_P._hongwonpyoi_YW37_8____      GCGTTTGCTTGAAAAGTATTGGCACGAGTGGTACTAAATAGTACTGACAG 500
YS_P._hongwonpyoi_YW34_8____      GCGTTTGCTTGAAAAGTATTGGCACGAGTGGTACTAAATAGTACTGACAG 500
YS_P._hongwonpyoi_YW34_2a___      GCGTTTGCTTGAAAAGTATTGGCACGAGTGGTACTAAATAGTACTGACAG 500
YS_P._viridicuprus_YW15_1____     GCGTTTGCTTGAAAAGTATTGGCACGAGTGGTACTAAATAGTACTGACAG 500
YS_P._hongwonpyoi_YW36_1____      GCGTTTGCTTGAAAAGTATTGGCACGAGTGGTACTAAATAGTACTGACAG 500
YS_P._angularis_YW25_8___         GCGTTTGCTTGAAAAGTATTGGCACGAGTGGTACTAAATAGTACTGACAG 499
                                  **************************************************

YS_P._acuticollis_YW07_8____      AATATTTCAATGTATTAGGTTTATCCAACTCGTTGAGACTTCTGGCGGTG 550
YS_P._takakuwai_YW54_1____        AATATTTCAATGTATTAGGTTTATCCAACTCGTTGAGACTTCTGGCGGTG 550
YS_P._albisomni_YW19_1____        AATATTTCAATGTATTAGGTTTATCCAACTCGTTGAGACTTCTGGCGGTG 550
YS_P._kawadai_YW53_1____          AATATTTCAATGTATTAGGTTTATCCAACTCGTTGAGACTTCTGGCGGTG 550
YS_P._kawadai_YW18_1____          AATATTTCAATGTATTAGGTTTATCCAACTCGTTGAGACTTCTGGCGGTG 550
YS_P._sue_YW45_1____              AATATTTCAATGTATTAGGTTTATCCAACTCGTTGAGACTTCTGGCGGTG 550
YS_P._sue_YW80_3____              AATATTTCAATGTATTAGGTTTATCCAACTCGTTGAGACTTCTGGCGGTG 550
YS_P._sugitai_YW44_1____          AATATTTCAATGTATTAGGTTTATCCAACTCGTTGAGACTTCTGGCGGTG 550
YS_P._urushiyamai_YW50_1____      AATATTTCAATGTATTAGGTTTATCCAACTCGTTGAGACTTCTGGCGGTG 550
YS_P._delicatulus_YW52_1____      AATATTTCAATGTATTAGGTTTATCCAACTCGTTGAGACTTCTGGCGGTG 550
```

```
YS_P._delicatulus_YW60_2____      AATATTTCAATGTATTAGGTTTATCCAACTCGTTGAGACTTCTGGCGGTG 550
YS_P._akitaorum_YW16_1____        AATATTTCAATGTATTAGGTTTATCCAACTCGTTGAGACTTCTGGCGGTG 550
YS_P._hongwonpyoi_YW05_8____      AATATTTCAATGTATTAGGTTTATCCAACTCGTTGAGACTTCTGGCGGTG 550
YS_P._hongwonpyoi_YW24_1____      AATATTTCAATGTATTAGGTTTATCCAACTCGTTGAGACTTCTGGCGGTG 550
YS_P._hongwonpyoi_YW33_8____      AATATTTCAATGTATTAGGTTTATCCAACTCGTTGAGACTTCTGGCGGTG 550
YS_P._hongwonpyoi_YW35_8____      AATATTTCAATGTATTAGGTTTATCCAACTCGTTGAGACTTCTGGCGGTG 550
YS_P._hongwonpyoi_YW37_8____      AATATTTCAATGTATTAGGTTTATCCAACTCGTTGAGACTTCTGGCGGTG 550
YS_P._hongwonpyoi_YW34_8____      AATATTTCAATGTATTAGGTTTATCCAACTCGTTGAGACTTCTGGCGGTG 550
YS_P._hongwonpyoi_YW34_2a___      AATATTTCAATGTATTAGGTTTATCCAACTCGTTGAGACTTCTGGCGGTG 550
YS_P._viridicuprus_YW15_1____     AATATTTCAATGTATTAGGTTTATCCAACTCGTTGAGACTTCTGGCGGTG 550
YS_P._hongwonpyoi_YW36_1____      AATATTTCAATGTATTAGGTTTATCCAACTCGTTGAGACTTCTGGCGGTG 550
YS_P._angularis_YW25_8___         AATATTTCAATGTATTAGGTTTATCCAACTCGTTGAGACTTCTGGCGGTG 549
                                  **************************************************

YS_P._acuticollis_YW07_8____      AATTTTTGGTATATTGGCTTTGCCTTACAAAACAACAAACAAGTTTGACC 600
YS_P._takakuwai_YW54_1____        AATTTTTGGTATATTGGCTTTGCCTTACAAAACAACAAACAAGTTTGACC 600
YS_P._albisomni_YW19_1____        AATTTTTGGTATATTGGCTTTGCCTTACAAAACAACAAACAAGTTTGACC 600
YS_P._kawadai_YW53_1____          AATTTTTGGTATATTGGCTTTGCCTTACAAAACAACAAACAAGTTTGACC 600
YS_P._kawadai_YW18_1____          AATTTTTGGTATATTGGCTTTGCCTTACAAAACAACAAACAAGTTTGACC 600
YS_P._sue_YW45_1____              AATTTTTGGTATATTGGCTTTGCCTTACAAAACAACAAACAAGTTTGACC 600
YS_P._sue_YW80_3____              AATTTTTGGTATATTGGCTTTGCCTTACAAAACAACAAACAAGTTTGACC 600
YS_P._sugitai_YW44_1____          AATTTTTGGTATATTGGCTTTGCCTTACAAAACAACAAACAAGTTTGACC 600
YS_P._urushiyamai_YW50_1____      AATTTTTGGTATATTGGCTTTGCCTTACAAAACAACAAACAAGTTTGACC 600
YS_P._delicatulus_YW52_1____      AATTTTTGGTATATTGGCTTTGCCTTACAAAACAACAAACAAGTTTGACC 600
YS_P._delicatulus_YW60_2____      AATTTTTGGTATATTGGCTTTGCCTTACAAAACAACAAACAAGTTTGACC 600
YS_P._akitaorum_YW16_1____        AATTTTTGGTATATTGGCTTTGCCTTACAAAACAACAAACAAGTTTGACC 600
YS_P._hongwonpyoi_YW05_8____      AATTTTTGGTATATTGGCTTTGCCTTACAAAACAACAAACAAGTTTGACC 600
YS_P._hongwonpyoi_YW24_1____      AATTTTTGGTATATTGGCTTTGCCTTACAAAACAACAAACAAGTTTGACC 600
YS_P._hongwonpyoi_YW33_8____      AATTTTTGGTATATTGGCTTTGCCTTACAAAACAACAAACAAGTTTGACC 600
YS_P._hongwonpyoi_YW35_8____      AATTTTTGGTATATTGGCTTTGCCTTACAAAACAACAAACAAGTTTGACC 600
YS_P._hongwonpyoi_YW37_8____      AATTTTTGGTATATTGGCTTTGCCTTACAAAACAACAAACAAGTTTGACC 600
YS_P._hongwonpyoi_YW34_8____      AATTTTTGGTATATTGGCTTTGCCTTACAAAACAACAAACAAGTTTGACC 600
YS_P._hongwonpyoi_YW34_2a___      AATTTTTGGTATATTGGCTTTGCCTTACAAAACAACAAACAAGTTTGACC 600
YS_P._viridicuprus_YW15_1____     AATTTTTGGTATATTGGCTTTGCCTTACAAAACAACAAACAAGTTTGACC 600
YS_P._hongwonpyoi_YW36_1____      AATTTTTGGTATATTGGCTTTGCCTTACAAAACAACAAACAAGTTTGACC 600
YS_P._angularis_YW25_8___         AATTTTTGGTATATTGGCTTTGCCTTACAAAACAACAAACAAGTTTGACC 599
```

```
                                    **************************************************

YS_P._acuticollis_YW07_8____        TCAAATCAGGTAGGATTACCCGCTGAACTTAAGCATATCAATAAGCGGAG 650
YS_P._takakuwai_YW54_1____          TCAAATCAGGTAGGATTACCCGCTGAACTTAAGCATATCAATAAGCGGAG 650
YS_P._albisomni_YW19_1____          TCAAATCAGGTAGGATTACCCGCTGAACTTAAGCATATCAATAAGCGGAG 650
YS_P._kawadai_YW53_1____            TCAAATCAGGTAGGATTACCCGCTGAACTTAAGCATATCAATAAGCGGAG 650
YS_P._kawadai_YW18_1____            TCAAATCAGGTAGGATTACCCGCTGAACTTAAGCATATCAATAAGCGGAG 650
YS_P._sue_YW45_1____                TCAAATCAGGTAGGATTACCCGCTGAACTTAAGCATATCAATAAGCGGAG 650
YS_P._sue_YW80_3____                TCAAATCAGGTAGGATTACCCGCTGAACTTAAGCATATCAATAAGCGGAG 650
YS_P._sugitai_YW44_1____            TCAAATCAGGTAGGATTACCCGCTGAACTTAAGCATATCAATAAGCGGAG 650
YS_P._urushiyamai_YW50_1____        TCAAATCAGGTAGGATTACCCGCTGAACTTAAGCATATCAATAAGCGGAG 650
YS_P._delicatulus_YW52_1____        TCAAATCAGGTAGGATTACCCGCTGAACTTAAGCATATCAATAAGCGGAG 650
YS_P._delicatulus_YW60_2____        TCAAATCAGGTAGGATTACCCGCTGAACTTAAGCATATCAATAAGCGGAG 650
YS_P._akitaorum_YW16_1____          TCAAATCAGGTAGGATTACCCGCTGAACTTAAGCATATCAATAAGCGGAG 650
YS_P._hongwonpyoi_YW05_8____        TCAAATCAGGTAGGATTACCCGCTGAACTTAAGCATATCAATAAGCGGAG 650
YS_P._hongwonpyoi_YW24_1____        TCAAATCAGGTAGGATTACCCGCTGAACTTAAGCATATCAATAAGCGGAG 650
YS_P._hongwonpyoi_YW33_8____        TCAAATCAGGTAGGATTACCCGCTGAACTTAAGCATATCAATAAGCGGAG 650
YS_P._hongwonpyoi_YW35_8____        TCAAATCAGGTAGGATTACCCGCTGAACTTAAGCATATCAATAAGCGGAG 650
YS_P._hongwonpyoi_YW37_8____        TCAAATCAGGTAGGATTACCCGCTGAACTTAAGCATATCAATAAGCGGAG 650
YS_P._hongwonpyoi_YW34_8____        TCAAATCAGGTAGGATTACCCGCTGAACTTAAGCATATCAATAAGCGGAG 650
YS_P._hongwonpyoi_YW34_2a___        TCAAATCAGGTAGGATTACCCGCTGAACTTAAGCATATCAATAAGCGGAG 650
YS_P._viridicuprus_YW15_1____       TCAAATCAGGTAGGATTACCCGCTGAACTTAAGCATATCAATAAGCGGAG 650
YS_P._hongwonpyoi_YW36_1____        TCAAATCAGGTAGGATTACCCGCTGAACTTAAGCATATCAATAAGCGGAG 650
YS_P._angularis_YW25_8___           TCAAATCAGGTAGGATTACCCGCTGAACTTAAGCATATCAATAAGCGGAG 649
                                    **************************************************

YS_P._acuticollis_YW07_8____        GATGAGATTAAGCTTCAGTTGTCCGATTTGTTTGTGTTACACAACACAAT 700
YS_P._takakuwai_YW54_1____          GATGAGATTAAGCTTCAGTTGTCCGATTTGTTTGTGTTACACAACACAAT 700
YS_P._albisomni_YW19_1____          GATGAGATTAAGCTTCAGTTGTCCGATTTGTTTGTGTTACACAACACAAT 700
YS_P._kawadai_YW53_1____            GATGAGATTAAGCTTCAGTTGTCCGATTTGTTTGTGTTACACAACACAAT 700
YS_P._kawadai_YW18_1____            GATGAGATTAAGCTTCAGTTGTCCGATTTGTTTGTGTTATACAACACAAT 700
YS_P._sue_YW45_1____                GATGAGATTAAGCTTCAGTTGTCCGATTTGTTTGTGTTACACAACACAAT 700
YS_P._sue_YW80_3____                GATGAGATTAAGCTTCAGTTGTCCGATTTGTTTGTGTTACACAACACAAT 700
YS_P._sugitai_YW44_1____            GATGAGATTAAGCTTCAGTTGTCCGATTTGTTTGTGTTACACAACACAAT 700
YS_P._urushiyamai_YW50_1____        GATGAGATTAAGCTTCAGTTGTCCGATTTGTTTGTGTTACACAACACAAT 700
YS_P._delicatulus_YW52_1____        GATGAGATTAAGCTTCAGTTGTCCGATTTGTTTGTGTTACACAACACAAT 700
```

```
YS_P._delicatulus_YW60_2____        GATGAGATTAAGCTTCAGTTGTCCGATTTGTTTGTGTTACACAACACAAT 700
YS_P._akitaorum_YW16_1____          GATGAGATTAAGCTTCAGTTGTCCGATTTGTTTGTGTTACACAACACAAT 700
YS_P._hongwonpyoi_YW05_8____        GATGAGATTAAGCTTCAGTTGTCCGATTTGTTTGTGTTACACAACACAAT 700
YS_P._hongwonpyoi_YW24_1____        GATGAGATTAAGCTTCAGTTGTCCGATTTGTTTGTGTTACACAACACAAT 700
YS_P._hongwonpyoi_YW33_8____        GATGAGATTAAGCTTCAGTTGTCCGATTTGTTTGTGTTACACAACACAAT 700
YS_P._hongwonpyoi_YW35_8____        GATGAGATTAAGCTTCAGTTGTCCGATTTGTTTGTGTTACACAACACAAT 700
YS_P._hongwonpyoi_YW37_8____        GATGAGATTAAGCTTCAGTTGTCCGATTTGTTTGTGTTACACAACACAAT 700
YS_P._hongwonpyoi_YW34_8____        GATGAGATTAAGCTTCAGTTGTCCGATTTGTTTGTGTTACACAACACAAT 700
YS_P._hongwonpyoi_YW34_2a___        GATGAGATTAAGCTTCAGTTGTCCGATTTGTTTGTGTTACACAACACAAT 700
YS_P._viridicuprus_YW15_1____       GATGAGATTAAGCTTCAGTTGTCCGATTTGTTTGTGTTACACAACACAAT 700
YS_P._hongwonpyoi_YW36_1____        GATGAGATTAAGCTTCAGTTGTCCGATTTGTTTGTGTTACACAACACAAT 700
YS_P._angularis_YW25_8___           GATGAGATTAAGCTTCAGTTGTCCGATTTGTTTGTGTTACACAACACAAT 699
                                    *************************************** **********

YS_P._acuticollis_YW07_8____        CTCCTCTAAGTGATAGTTGGCAGGCGCTAACTAGTATTTAGAGGAGTTTT 750
YS_P._takakuwai_YW54_1____          CTCCTCTAAGTGATAGTTGGCAGGCGCTAACTAGTATTTAGAGGAGTTTC 750
YS_P._albisomni_YW19_1____          CTCCTCTAAGTGATAGTTGGCAGGCGCTAACTAGTATTTAGAGGAGTTTT 750
YS_P._kawadai_YW53_1____            CTCCTCTAAGTGATAGTTGGCAGGCGCTAACTAGTATTTAGAGGAGTTTT 750
YS_P._kawadai_YW18_1____            CTCCTCTAAGTGATAGTTGGCAGGTGCTAACTAGTATTTAGAGGAGTTTT 750
YS_P._sue_YW45_1____                CTCCTCTAAGTGATAGTTGGCAGGCGCTAACTAGTATTTAGAGGAGTTTT 750
YS_P._sue_YW80_3____                CTCCTCTAAGTGATAGTTGGCAGGCGCTAACTAGTATTTAGAGGAGTTTT 750
YS_P._sugitai_YW44_1____            CTCCTCTAAGTGATAGTTGGCAGGCGCTAACTAGTATTTAGAGGAGTTTT 750
YS_P._urushiyamai_YW50_1____        CTCCTCTAAGTGATAGTTGGCAGGTGCTAACTAGTATTTAGAGGAGTTTT 750
YS_P._delicatulus_YW52_1____        CTCCTCTAAGTGATAGTTGGCAGGCGCTAGCTAGTATTTAGAGGAGTTTT 750
YS_P._delicatulus_YW60_2____        CTCCTCTAAGTGATAGTTGGCAGGCGCTAGCTAGTATTTAGAGGAGTTTT 750
YS_P._akitaorum_YW16_1____          CTCCTCTAAGTGATAGTTGGCAGGCGCTAACTAGTATTTAGAGGAGTTTT 750
YS_P._hongwonpyoi_YW05_8____        CTCCTCTAAGTGATAGTTGGCAGGCGCTAACTAGTATTTAGAGGAGTTTT 750
YS_P._hongwonpyoi_YW24_1____        CTCCTCTAAGTGATAGTTGGCAGGCGCTAACTAGTATTTAGAGGAGTTTT 750
YS_P._hongwonpyoi_YW33_8____        CTCCTCTAAGTGATAGTTGGCAGGCGCTAACTAGTATTTAGAGGAGTTTT 750
YS_P._hongwonpyoi_YW35_8____        CTCCTCTAAGTGATAGTTGGCAGGCGCTAACTAGTATTTAGAGGAGTTTT 750
YS_P._hongwonpyoi_YW37_8____        CTCCTCTAAGTGATAGTTGGCAGGCGCTAACTAGTATTTAGAGGAGTTTT 750
YS_P._hongwonpyoi_YW34_8____        CTCCTCTAAGTGATAGTTGGCAGGCGCTAACTAGTATTTAGAGGAGTTTT 750
YS_P._hongwonpyoi_YW34_2a___        CTCCTCTAAGTGATAGTTGGCAGGCGCTAACTAGTATTTAGAGGAGTTTT 750
YS_P._viridicuprus_YW15_1____       CTCCTCTAAGTGATAGTTGGCAGGCGCTAACTAGTATTTAGAGGAGTTTT 750
YS_P._hongwonpyoi_YW36_1____        CTCCTCTAAGTGATAGTTGGCAGGCGCTAACTAGTATTTAGAGGAGTTTT 750
YS_P._angularis_YW25_8___           CTCCTCTAAGTGATAGTTGGCAGGCGCTAACTAGTATTTAGAGGAGTTTT 749
```

```
                                   *********************** **** ******************

YS_P._acuticollis_YW07_8____       -ACATTTCTTGCAAACACTGATGTCCGGGTAACCCCCGATAAAATGGTTG 799
YS_P._takakuwai_YW54_1____         -ACATTTCTTGCAAACACTGATGTCCGGGTAACCCCCGATAAAATGGTTG 799
YS_P._albisomni_YW19_1____         -ACATTTCTTGCAAACACTGATGTCCGGGTAACCCCCGATAAAATGGTTG 799
YS_P._kawadai_YW53_1____           -ACATTTCTTGCAAACACTGATGTCCGGGTAACCCCCGATAAAATGGTTG 799
YS_P._kawadai_YW18_1____           -ACATTTCTTGCAAACACTGATGTCCGGGTAACCCCCGATAAAATGGATG 799
YS_P._sue_YW45_1____               -ACATTTCTTGCAAACACTGATGTCCGGGTAACCCCTGATAAAATGGTTG 799
YS_P._sue_YW80_3____               -ACATTTCTTGCAAACATTGATGTCCGGGTAACCCCTGATAAAATGGTTG 799
YS_P._sugitai_YW44_1____           -ACATTTCTTGCAAACACTGATGTCCGGGTAACCCCTGATAAAATGGTTG 799
YS_P._urushiyamai_YW50_1____       -ACATTTCTTGCAAACACTGATGTCCGGGTAACCCCTGATAAAATGGTTG 799
YS_P._delicatulus_YW52_1____       -ACATTTCTTGCAAACACTGATGTCCGGGTAACCCCTGATAAAATGGTTG 799
YS_P._delicatulus_YW60_2____       -ACATTTCTTGCAAACATTGATGTCCGGGTAACCCCTGATAAAATGGTTG 799
YS_P._akitaorum_YW16_1____         -ACATTTCTTGCAAACACTGATGTCCGGGTAACCCCTGATAAAATGGTTG 799
YS_P._hongwonpyoi_YW05_8____       -ACATTTCTTGCAAACACTGATGTCCGGGTAACCCCTGATAAAATGGTTG 799
YS_P._hongwonpyoi_YW24_1____       -ACATTTCTTGCAAACACTGATGTCCGGGTAACCCCTGATAAAATGGTTG 799
YS_P._hongwonpyoi_YW33_8____       -ACATTTCTTGCAAACACTGATGTCCGGGTAACCCCTGATAAAATGGTTG 799
YS_P._hongwonpyoi_YW35_8____       -ACATTTCTTGCAAACACTGATGTCCGGGTAACCCCTGATAAAATGGTTG 799
YS_P._hongwonpyoi_YW37_8____       -ACATTTCTTGCAAACACTGATGTCCGGGTAACCCCTGATAAAATGGTTG 799
YS_P._hongwonpyoi_YW34_8____       -ACATTTCTTGCAAACACTGATGTCCGGGTAACCCCTGATAAAATGGTTG 799
YS_P._hongwonpyoi_YW34_2a___       -ACATTTCTTGCAAACACTGATGTCCGGGTAACCCCTGATAAAATGGTTG 799
YS_P._viridicuprus_YW15_1____      -ACATTTCTTGCAAACACTGATGTCCGGGTAACCCCTGATAAAATGGTTG 799
YS_P._hongwonpyoi_YW36_1____       -ACATTTCTTGCAAACATTGATGTCCGGGTAACCCCTGATAAAATGGTTG 799
YS_P._angularis_YW25_8___          TACATTTCTTGCAAACACTGATGTCCGGGTAACCCCTGATAAAATGGTTG 799
                                    **************** ****************** ********** **

YS_P._acuticollis_YW07_8____       CGAAAAAGTT-GAGAAAAGTCTTTACAGAATTTTTTTT-AAGAATTTTTA 847
YS_P._takakuwai_YW54_1____         CGAAAAAGTT-GAGAAAAGTCTTTACAGAATTTTTTTT-AAGAATTTTTA 847
YS_P._albisomni_YW19_1____         CGAAAAAGTT-GAGAAAAGTCTTTACAGAATTTTTTTT-AAGAATTTTTA 847
YS_P._kawadai_YW53_1____           CGAAAAAGTT-GAGAAAAGTCTTTACAGAATTTTTTTT-AAGAATTTTTA 847
YS_P._kawadai_YW18_1____           CGAAAAAGTT-GAGAAAAGTCTTTACAGAATTTTTTTT-AAGAATTTTTA 847
YS_P._sue_YW45_1____               CGAAAAAGTT-GAGAAAAGTCTTTACAGAATTCTTTTT-AAGAATTTTTA 847
YS_P._sue_YW80_3____               CGAAAAAGTT-GAGAAAAGTCTTTACAGAATTCTTTTT-AAGAATTTTTA 847
YS_P._sugitai_YW44_1____           CGAAAAAGTT-GAGAAAAGTCTTTACAG--------------AATTTTTA 834
YS_P._urushiyamai_YW50_1____       CGAAAAAGTT-GAGAAAAGTCTTTACAGAATTTTTTTT-AAGAATTTTTA 847
YS_P._delicatulus_YW52_1____       CGAAAAAGTT-GAGAAAAGTCTTTACAGAATTTTTTTTTAAGAATTTTTA 848
```

```
YS_P._delicatulus_YW60_2____        CGAAAAAGTT-GAGAAAAGTCTTTACAGAATTTTTTTTTAAGAATTTTTA 848
YS_P._akitaorum_YW16_1____          CGAAAAAGTT-GAGAAAAGTCTTTACAGAATTTTTTTT-AAGAATTTTTT 847
YS_P._hongwonpyoi_YW05_8____        CGAAAAAGTTTGAAAAAAGTCTTTACAGAATTTTTTTT-AAGAATTTTTA 848
YS_P._hongwonpyoi_YW24_1____        CGAAAAAGTTTGAAAAAAGTCTTTACAGAATTTTTTTT-AAGAATTTTTA 848
YS_P._hongwonpyoi_YW33_8____        CGAAAAAGTTTGAAAAAAGTCT--ACAGAATTTTTTTT-AAGAATTTTTA 846
YS_P._hongwonpyoi_YW35_8____        CGAAAAAGTTTGAAAAAAGTCT--ACAGAATTTTTTTT-AAGAATTTTTA 846
YS_P._hongwonpyoi_YW37_8____        CGAAAAAGTTTGAAAAAAGTCTTTACAGAATTTTTTTT-AAGAATTTTTA 848
YS_P._hongwonpyoi_YW34_8____        CGAAAAAGTTTGAAAAAAGTCTTTACAGAATTTTTTTA-AAGAATTTTTA 848
YS_P._hongwonpyoi_YW34_2a___        CGAAAAAGTTTGAAAAAAGTCTTTACAGAATTTTTTTA-AAGAATTTTTA 848
YS_P._viridicuprus_YW15_1____       CGAAAAAGTTTGAAAAAAGTCTTTACAGAATTTTTTTA-A-GAATTTTTA 847
YS_P._hongwonpyoi_YW36_1____        CGAAAAAGTTTGAGAAAAGTCTTTACAGAATTTTTTTT-AAGAATTTTTA 848
YS_P._angularis_YW25_8___           CGAAAAAGTCTGAGAAAAGTCTTTACAGA-TTTTTTTTGAAGAATTTTTA 848
                                    *********  ** ********  ****              *******

YS_P._acuticollis_YW07_8____        GATCTGGCTCTGTGTAGTTCTGTGTATATTTATTTAGAGAGAGCACTGTG 897
YS_P._takakuwai_YW54_1____          GATCTGGTTCTGTGTAGTTCTGTGTATATTTATTTAGAGAGAGCACTGTG 897
YS_P._albisomni_YW19_1____          GATCTGGTTCTGTGTAGTTCTGTGTATATTTATTTAGAGAGAGCACTGTG 897
YS_P._kawadai_YW53_1____            GATCTGGTTCTGTGTAGTTCTGTGTATATTTATTTAGAGAGAGCACTGTG 897
YS_P._kawadai_YW18_1____            GATCTGGTTCTGTGTAGTTCTGTGTATATTTATTTAGAGAGAGCACTGTG 897
YS_P._sue_YW45_1____                GATCTGGTTCTGTGTAGTTCTGTGTATATTTATTTAGAGAGAGCACTGTG 897
YS_P._sue_YW80_3____                GATCTGGTTCTGTGTAGTTCTGTGTATATTTATTTAGAGAGAGCACTGTG 897
YS_P._sugitai_YW44_1____            GATCTGGTTCTGTGTAGTTCTGTGTATATTTATTTAGAGAGAGCACTGTG 884
YS_P._urushiyamai_YW50_1____        GATCTGGTTCTGTGTAGTTCTGTGTATATTTATTTAGAGAGAGCACTGTG 897
YS_P._delicatulus_YW52_1____        AATCTGGTTCTGTGTAGTTCAGTGTATATTTATATAGAGAGAGCACTGTG 898
YS_P._delicatulus_YW60_2____        AATCTGGTTCTGTGTAGTTCAGTGTATATTTATATAGAGAGAGCACTGTG 898
YS_P._akitaorum_YW16_1____          GATCTGGTTCTGTGTAGTTCTGTGTATATTTATTTAGAGAGAGCACTGTG 897
YS_P._hongwonpyoi_YW05_8____        GATCTGGTTCTGTGTAGTTCTGTGTATATTTATTTAGAGAGAGCACTGTG 898
YS_P._hongwonpyoi_YW24_1____        GATCTGGTTCTGTGTAGTTCTGTGTATATTTATTTAGAGAGAGCACTGTG 898
YS_P._hongwonpyoi_YW33_8____        GATCTGGTTCTGTGTAGTTCTGTGTATATTTATTTAGAGAGAGCACTGTG 896
YS_P._hongwonpyoi_YW35_8____        GATCTGGTTCTGTGTAGTTCTGTGTATATTTATTTAGAGAGAGCACTGTG 896
YS_P._hongwonpyoi_YW37_8____        GATCTGGTTCTGTGTAGTTCTGTGTATATTTATGTAGAGAGAGCACTGTG 898
YS_P._hongwonpyoi_YW34_8____        GATCTGGTTCTGTGTAGTTCTGTGTAT--TTATTTAGAGAGAGCACTGTG 896
YS_P._hongwonpyoi_YW34_2a___        GATCTGGTTCTGTGTAGTTCTGTGTATATTTATTTAGAGAGAGCACTGTG 898
YS_P._viridicuprus_YW15_1____       GATCTGGTTCTGTGTAGTTCTGTGTATATTTATTTAGAGAGAGCACTGTG 897
YS_P._hongwonpyoi_YW36_1____        GATCTGGTTCTGTGTAGTTCTGTGTATATTTATTTAGAGAGAGCACTGTG 898
YS_P._angularis_YW25_8___           GATCTGGTTCTGTGTAGTTCTGTGTATATATATTTAGAGAGAGCAGTGTG 898
```

```
                                    ****** ************ ******   *** *********** ****

YS_P._acuticollis_YW07_8____       GATCTGAGTTAGATAGAGACAAATTTTAGGAACAAGAGTCGAGTCGAAAT 947
YS_P._takakuwai_YW54_1____         GATCTGAGTTAGAAAGAGACAAATTTTAGGAACAAGAGTCGAGTCGAAAT 947
YS_P._albisomni_YW19_1____         GATCTGAGTTAGATAGAGACAAATTTTAGGAACAAGAGTCGAGTCGAAAT 947
YS_P._kawadai_YW53_1____           GATCTGAGTTAGATAGAGACAAATTTTAGGAACAAGAGTCGAGTCGAAAT 947
YS_P._kawadai_YW18_1____           GATCTGAGTTAGATAGAGACAAATTTTAGGAACAAGAGTCGAGTCGAAAT 947
YS_P._sue_YW45_1____               GATCTGAGTTAGATAGAGACAAATTTTAGGAACAAGAGTCGAGTCGAAGT 947
YS_P._sue_YW80_3____               GATCTGAGTTAGATAGAGACAAATTTTAGGAACAAGAGTCGAGTCGAAGT 947
YS_P._sugitai_YW44_1____           GATCTGAGTTAGATAGAGACAAATTTTAGGAACAAGAGTCGAGTCGAAAT 934
YS_P._urushiyamai_YW50_1____       GATCTGAGTTAGATAGAGACAAATTTTAGGAACAAGAGTCGAGTCAAAAT 947
YS_P._delicatulus_YW52_1____       GATCTGAGTTAGATAGAGACAAATTTTAGGAACAAGAGTCGAGTCGAAAT 948
YS_P._delicatulus_YW60_2____       GATCTGAGTTAGATAGAGACAAATTTTAGGAACAAGAGTCGAGTCGAAAT 948
YS_P._akitaorum_YW16_1____         GATCTGAGTTAGATAGAGACAAATTTTAGGAACAAGAGTCGAGTCGAAAT 947
YS_P._hongwonpyoi_YW05_8____       GATCTGAGTTAGATAGAGACAAATTTTAGGAACAAGAGTCGAGTCGAAAT 948
YS_P._hongwonpyoi_YW24_1____       GATCTGAGTTAGATAGAGACAAATTTTAGGAACAAGAGTCGAGTCGAAAT 948
YS_P._hongwonpyoi_YW33_8____       GATCTGAGTTAGATAGAGACAAATTTTAGGAACAAGAGTCGAGTCGAAAT 946
YS_P._hongwonpyoi_YW35_8____       GATCTGAGTTAGATAGAGACAAATTTTAGGAACAAGAGTCGAGTCGAAAT 946
YS_P._hongwonpyoi_YW37_8____       GATCTGAGTTAGATAGAGACAAATTTTAGGAACAAGAGTCGAGTCGAAAT 948
YS_P._hongwonpyoi_YW34_8____       GATCTGAGTTAGATAGAGACAAATTTTAGGAACAAGAGTCGAGTCGAAAT 946
YS_P._hongwonpyoi_YW34_2a___       GATCTGAGTTAGATAGAGACAAATTTTAGGAACAAGAGTCGAGTCGAAAT 948
YS_P._viridicuprus_YW15_1____      GATCTGAGTTAGATAGAGACAAATTTTAGGAACAAGAGTCGAGTCGAAAT 947
YS_P._hongwonpyoi_YW36_1____       GATCTGAGTTAGATAGAGACAAATTTTAGGAACAAGAGTCGAGTCGAAAT 948
YS_P._angularis_YW25_8___          GTTCTGAGTTAGATAGAGACGAATTTTAGGAACGAGAGTCGA-----AAT 943
                                   * *********** ****** ************ ********     * *

YS_P._acuticollis_YW07_8____       CGGTCACGTGTTGAAACGATAGACATGGAAAGGAAGGATAACGGTAGAGT 997
YS_P._takakuwai_YW54_1____         CGGTCACGTGTTGAAACGATAGACATGGAAAGGAAGGATAACGGTAGAGT 997
YS_P._albisomni_YW19_1____         CGGTCACGTGTTGAAACGATAGACATGGAAAGGAAGGATAACGGTAGAGT 997
YS_P._kawadai_YW53_1____           CGGTCACGTGTTGAAACGATAGACATGGAAAGGAAGGATAACGGTAGAGT 997
YS_P._kawadai_YW18_1____           CGGTCACGTGTTGAAACGATAGACATGGAAAGGAAGCATAACGGTAGAGT 997
YS_P._sue_YW45_1____               CGGTCACGTGTTGAAATGATAGACATGGAAAGGAAGCATAACGGTAGAGT 997
YS_P._sue_YW80_3____               CGGTCACGTGTTGAAATGATAGACATGGAAAGGAAGCATAACGGTAGAGT 997
YS_P._sugitai_YW44_1____           CGGTCACGTGTTGAAATGATAGACATGGAAAGGAAGCATAACGGTAGAGT 984
YS_P._urushiyamai_YW50_1____       CGGTCACGTGTTGAAACGATAGACATGGAAAGGAAGCATAACGGTAGAGT 997
YS_P._delicatulus_YW52_1____       CGGTCACGTGTTGAAACGATAGACATGGAAAGGAAGCATAACGGTAGAGT 998
```

```
YS_P._delicatulus_YW60_2____        CGGTCACGTGTTGAAACGATAGACATGGAAAGGAAGCATAACGGTAGAGT 998
YS_P._akitaorum_YW16_1____          CGGTCACGTGTTGAAACGATAGACATGGAAAGGAAGCATAACGGTAGAGT 997
YS_P._hongwonpyoi_YW05_8____        CGGTCACGTGTTAAAACGATAGACATGGAAAGGAAGCATAACGGTAGAGT 998
YS_P._hongwonpyoi_YW24_1____        CGGTCACGTGTTAAAACGATAGACATGGAAAGGAAGCATAACGGTAGAGT 998
YS_P._hongwonpyoi_YW33_8____        CGGTCACGTGTTAAAACGATAGACATGGAAAGGAAGCATAACGGTAGAGT 996
YS_P._hongwonpyoi_YW35_8____        CGGTCACGTGTTAAAACGATAGACATGGAAAGGAAGCATAACGGTAGAGT 996
YS_P._hongwonpyoi_YW37_8____        CGGTCACGTGTTAAAACGATAGACATGGAAAGGAAGCATAACGGTAGAGT 998
YS_P._hongwonpyoi_YW34_8____        CGGTCACGTGTTAAAACGATAGACATGGAAAGGAAGCATAACGGTAGAGT 996
YS_P._hongwonpyoi_YW34_2a___        CGGTCACGTGTTAAAACGATAGACATGGAAAGGAAGCATAACGGTAGAGT 998
YS_P._viridicuprus_YW15_1____       CGGTCACGTGTTAAAACGATAGACATGGAAAGGAAGCATAACGGTAGAGT 997
YS_P._hongwonpyoi_YW36_1____        CGGTCACGTGTTAAAACGATAGACATGGAAAGGAAGCATAACGGTAGAAT 998
YS_P._angularis_YW25_8___           CGGTCACGTGTTGAAACGATAGACATTGACAGGAAGAATAACGGCAGAGT 993
                                    ************ *** ********* ** ****** ******* *** *

YS_P._acuticollis_YW07_8____        AAGAAAAGTGTTAAGAATTTTGAAAAA-ATTGTTTTGTAGGTAGTAGAAG 1046
YS_P._takakuwai_YW54_1____          AAGAAAAGTGTTAAGAATTTTGAAACA-ATTGTTTTGTAGGTAGTAGAAG 1046
YS_P._albisomni_YW19_1____          AAGAAAAGTGTTAAGAATTTTGAAAAA-ATTGTTTTGTAGGTAGTAGAAG 1046
YS_P._kawadai_YW53_1____            AAGAAAAGTGTTAAGAATTTTGAAAAA-ATTGTTTTGTAGGTAGTAGAAG 1046
YS_P._kawadai_YW18_1____            AAGAAAAGTGTTAAGAATTTTGAAAAA-ATTGTTTTGTAGGTAGTAGAAG 1046
YS_P._sue_YW45_1____                AAGAAAAGTGTTAAGAATTTTGAAAAA-ATTGTGTTGTAGGTAATAGAAG 1046
YS_P._sue_YW80_3____                AAGAAAAGTGTTAAGAATTTTGAAAAA-ATTGTGTTGTAGGTAATAGAAG 1046
YS_P._sugitai_YW44_1____            AAGAAAAGTGTTAAGAATTTTGAAAAA-ATTGTGTTGTAGGTAATAGAAG 1033
YS_P._urushiyamai_YW50_1____        AAGAAAAGTGTTAAGAATTTTGAAAAA-ATTGTTTTGTAGGTAATAGAAG 1046
YS_P._delicatulus_YW52_1____        AAGAAAAGTGTTAAGAATTTTGAAAAA-ATTGTTTTGTAGGTAGTAGAAG 1047
YS_P._delicatulus_YW60_2____        AAGAAAAGTGTTAAGAATTTTGAATAA-ATTGTTTTGTAGGTAGTAGAAG 1047
YS_P._akitaorum_YW16_1____          AAGAAAAGTGTTAAGAATTTTGAAAAA-ATTGTTTTGTAGGTAGTAGAAG 1046
YS_P._hongwonpyoi_YW05_8____        AAGAAAAGTGTTAAGAATTTTGAAAAATATTGTTTTGTAGGTAGTAGAAG 1048
YS_P._hongwonpyoi_YW24_1____        AAGAAAAGTGTTAAGAATTTTGAAAAATATTGTTTTGTAGGTAGTAGAAG 1048
YS_P._hongwonpyoi_YW33_8____        AAGAAAAGTGTTAAGAATTTTGAAAAATATTGTTTTGTAGGTAGTAGAAG 1046
YS_P._hongwonpyoi_YW35_8____        AAGAAAAGTGTTAAGAATTTTGAAAAATATTGTTTTGTAGGTAGTAGAAG 1046
YS_P._hongwonpyoi_YW37_8____        AAGAAAAGTGTTAAGAATTTTGAAAAATATTGTTTTGTAGGTAGTAGAAG 1048
YS_P._hongwonpyoi_YW34_8____        AAGAAAAGTGTTAAGAATTTTGAAAAATATTGTTTTGTAGGTAGTAGAAG 1046
YS_P._hongwonpyoi_YW34_2a___        AAGAAAAGTGTTAAGAATTTTGAAAAATATTGTTTTGTAGGTAGTAGAAG 1048
YS_P._viridicuprus_YW15_1____       AAGAAAAGTGTTAAGAATTTTGAAAAATATTGTTTTGTAGGTAGTAGAAG 1047
YS_P._hongwonpyoi_YW36_1____        AAGAAAAGTGTTAAGAATTTTGAAAAATATTGTTTTGTAGGTAGTAGAAG 1048
YS_P._angularis_YW25_8___           AAGAAAAGTGTTAAGAATTTTGAAAAATAATGTTTTGTAGGTAGTAGAAG 1043
```

```
                                    ************************  * * *** ********* ******

YS_P._acuticollis_YW07_8____        AAACTCGTTAGGAGTATTAAATGGTTGAGGAAAAATCGAAGCGGGAGCGA 1096
YS_P._takakuwai_YW54_1____          AAACTCGTTAGGAGTATTAAATGGTTGAGGAAAAATCGAAGCGGGAGCGA 1096
YS_P._albisomni_YW19_1____          AAACTCGTTAGGAGTATTAAATGGTTGAGGAAAAATCGAAGCGGGAGCGA 1096
YS_P._kawadai_YW53_1____            AAACTCGTTAGGAGTATTAAATGGTTGAGGAAAAATCGAAGCGGGAGCGA 1096
YS_P._kawadai_YW18_1____            AAACTCGCTATGAGTATTAAATGGTTGAGGAAAAATCGAAGCGGGAGCGA 1096
YS_P._sue_YW45_1____                AAACTCGTTAGGAGTATTAAATGGTTCAGGAAAAATCGAAGCGGGAGCGA 1096
YS_P._sue_YW80_3____                AAACTCGTTAGGAGTATTAAATGGTTCAGGAAAAATCGAAGCGGGAGCGA 1096
YS_P._sugitai_YW44_1____            AAACTCGTTAGGAGTATTAAATGGTTCAGGAAAAATCGAAGCGGGAGCGA 1083
YS_P._urushiyamai_YW50_1____        AAACTCGTTAGGAGTATTAAATGGTTGAGGAAAAATCGAAGCGGGAGCGA 1096
YS_P._delicatulus_YW52_1____        AAACTCGTTAGGAGTATTAAATGGTTGAGGAAAAATCGAAGCGGGAGCGA 1097
YS_P._delicatulus_YW60_2____        AAACTCGTTAGGAGTATTAAATGGTTGAGGAAAAATCGAAGCGGGAGCGA 1097
YS_P._akitaorum_YW16_1____          AAACTCGTTAGGAGTATTAAATGGTTGAGGAAAAATCGAAGCGGGAGCGA 1096
YS_P._hongwonpyoi_YW05_8____        AAGAGTGTTAGGAGTATTAAATGGTTGAGGAAAAATCGAAGCGGGAGCGA 1098
YS_P._hongwonpyoi_YW24_1____        AAGAGTGTTAGGAGTATTAAATGGTTGAGGAAAAATCGAAGCGGGAGCGA 1098
YS_P._hongwonpyoi_YW33_8____        AAGAGTGTTAGGAGTATTAAATGGTTGAGGAAAAATCGAAGCGGGAGCGA 1096
YS_P._hongwonpyoi_YW35_8____        AAGAGTGTTAGGAGTATTAAATGGTTGAGGAAAAATCGAAGCGGGAGCGA 1096
YS_P._hongwonpyoi_YW37_8____        AAGAGTGTTAGGAGTATTAAATGGTTGAGGAAAAATCGAAGCGGGAGCGA 1098
YS_P._hongwonpyoi_YW34_8____        AAGAGTGTTAGGAGTATTAAATGGTTGAGGAAAAATCGAAGCGGGAGCGA 1096
YS_P._hongwonpyoi_YW34_2a___        AAGAGTGTTAGGAGTATTAAATGGTTGAGGAAAAATCGAAGCGGGAGCGA 1098
YS_P._viridicuprus_YW15_1____       AAGAGTGTTAGGAGTATTAAATGGTTGAGGAAAAATCGAAGCGGGAGCGA 1097
YS_P._hongwonpyoi_YW36_1____        AAGAGTGTTAGGAGTATTAAATGGTTGAGGAAAAATCGAAGCGGGAGCGA 1098
YS_P._angularis_YW25_8___           AAGAGTGTTAGGAGTATAAAATGGTAGAAGAAAAATCGAAGCGGTAGCGA 1093
                                    **    * ** ****** *******  * *************** *****

YS_P._acuticollis_YW07_8____        TGGGGTCACGTGACTAGCGCATAGTTAAGAAAGCGAAAAAAAGATTGCAG 1146
YS_P._takakuwai_YW54_1____          TGGGGTCACGTGACTAGCGCATAGTTAAGAAAGCGAAAAAAAGATTGCAG 1146
YS_P._albisomni_YW19_1____          TGGGGTCACGTGACTAGCGCATAGTTAAGAAAGCGAAAAAAAGATTGCAG 1146
YS_P._kawadai_YW53_1____            TGGGGTCACGTGACTAGCGCATAGTTAAGAAAGCGAAAAAAAGATTGCAG 1146
YS_P._kawadai_YW18_1____            CGGGGTCACGTGACTAGCGCATAGTTAAGAAAGCGAAAAAAAGATTGCAG 1146
YS_P._sue_YW45_1____                TGAGGTCACGTGACTAGCGCATAGTTAAGAAAGCGAAAAAAAGATTGCAG 1146
YS_P._sue_YW80_3____                TGAGGTCACGTGACTAGCGCATAGTTAAGAAAGCGAAAAAAAGATTGCAG 1146
YS_P._sugitai_YW44_1____            TGAGGTCACGTGACTAGCGCATAGTTAAGAAAGCGAAAAAAAGATTGCAG 1133
YS_P._urushiyamai_YW50_1____        TGGGGTCACGTGACTAGCGCATAGTTAAGAAAGCGAAAAAAAGATTGCAG 1146
YS_P._delicatulus_YW52_1____        TGGGGTCACGTGACTAGCGCATAGTTAAGAAAGCGAAAAAAAGATTGCAG 1147
```

```
YS_P._delicatulus_YW60_2____        TGGGGTCACGTGACTAGCGCATAGTTAAGAAAGCGAAAAAAAGATTGCAG 1147
YS_P._akitaorum_YW16_1____          TGGGGTCACGTGACTAGAGCATAGTTAAGAAAGCGAAAAAAAGATTGCAG 1146
YS_P._hongwonpyoi_YW05_8____        TGGGGTCACGTGACTAGCGCATAGTTAAGAAAGCGAAAAAAAGATTGCAG 1148
YS_P._hongwonpyoi_YW24_1____        TGGGGTCACGTGACTAGCGCATAGTTAAGAAAGCGAAAAAAAGATTGCAG 1148
YS_P._hongwonpyoi_YW33_8____        TGGGGTCACGTGACTAGCGCATAGTTAAGAAAGCGAAAAAAAGATTGCAG 1146
YS_P._hongwonpyoi_YW35_8____        TGGGGTCACGTGACTAGCGCATAGTTAAGAAAGCGAAAAAAAGATTGCAG 1146
YS_P._hongwonpyoi_YW37_8____        TGGGGTCACGTGACTAGCGCATAGTTAAGAAAGCGAAAAAAAGATTGCAG 1148
YS_P._hongwonpyoi_YW34_8____        TGGAGTCACGTGACTAGCGCATAGTTAAGAAAGCGAAAAAAAGATTGCAG 1146
YS_P._hongwonpyoi_YW34_2a___        TGGAGTCACGTGACTAGCGCATAGTTAAGAAAGCGAAAAAAAGATTGCAG 1148
YS_P._viridicuprus_YW15_1____       TGGGGTCACGTGACTAGCGCATAGTTAAGAAAGCGAAAAAAAGATTGCAG 1147
YS_P._hongwonpyoi_YW36_1____        TGGAGTCACGTGACTAGTGCATAGTTAAGAAAGCGAAAAAAAGATTGCAG 1148
YS_P._angularis_YW25_8___           TGAGATCACGTGACTAGCACATAGTTAAGAAAGCGAAAAAAAGATTGCAG 1143
                                     *   ************  *******************************

YS_P._acuticollis_YW07_8____        CACGAGAGTTTCGCGTATGGTCTCCCACTACACTACTCGGTCTCGCTCTT 1196
YS_P._takakuwai_YW54_1____          CACGAGAGTTTCGCGTATGGTCTCCCACTACACTACTCGGTCTCGCTCTT 1196
YS_P._albisomni_YW19_1____          CACGAGAGTTTCGCGTATGGTCTCCCACTACACTACTCGGTCTCGCTCTT 1196
YS_P._kawadai_YW53_1____            CACGAGAGTTTCGCGTATGGTCTCCCACTACACTACTCGGTCTCGCTCTT 1196
YS_P._kawadai_YW18_1____            CACGAGAGTTTCGCGTATGGTCTCCCACTACACTACTCGGTCTCGCTCTT 1196
YS_P._sue_YW45_1____                CACGAGAGTTTCGCGTATGGTCTCCCACTACACTACTCGGTCTCGCTCTT 1196
YS_P._sue_YW80_3____                CACGAGAGTTTCGCGTATGGTCTCCCACTACACTACTCGGTCTCGCTCTT 1196
YS_P._sugitai_YW44_1____            CACGAGAGTTTCGCGTATGGTCTCCCACTACACTACTCGGTCTCGCTCTT 1183
YS_P._urushiyamai_YW50_1____        CACGAGAGTTTCGCGTATGGTCTCCCACTACACTACTCGGTCTCGCTCTT 1196
YS_P._delicatulus_YW52_1____        CACGAGAGTTTCGCGTATGGTCTCCCACTACACTACTCGGTCTCGCTCTT 1197
YS_P._delicatulus_YW60_2____        CACGAGAGTTTCGCGTATGGTCTCCCACTACACTACTCGGTCTCGCTCTT 1197
YS_P._akitaorum_YW16_1____          CACGAGAGTTTCGCGTATGGTCTCCCACTACACTACTCGGTCTCGCTCTT 1196
YS_P._hongwonpyoi_YW05_8____        CACGAGAGTTTCGCGTATGGTCTCCCACTACACTACTCGGTCTCGCTCTT 1198
YS_P._hongwonpyoi_YW24_1____        CACGAGAGTTTCGCGTATGGTCTCCCACTACACTACTCGGTCTCGCTCTT 1198
YS_P._hongwonpyoi_YW33_8____        CACGAGAGTTTCGCGTATGGTCTCCCACTACACTACTCGGTCTCGCTCTT 1196
YS_P._hongwonpyoi_YW35_8____        CACGAGAGTTTCGCGTATGGTCTCCCACTACACTACTCGGTCTCGCTCTT 1196
YS_P._hongwonpyoi_YW37_8____        CACGAGAGTTTCGCGTATGGTCTCCCACTACACTACTCGGTCTCGCTCTT 1198
YS_P._hongwonpyoi_YW34_8____        CACGAGAGTTTCGCGTATGGTCTCCCACTACACTACTCGGTCTCGCTCTT 1196
YS_P._hongwonpyoi_YW34_2a___        CACGAGAGTTTCGCGTATGGTCTCCCACTACACTACTCGGTCTCGCTCTT 1198
YS_P._viridicuprus_YW15_1____       CACGAGAGTTTCGCGTATGGTCTCCCACTACACTACTCGGTCTCGCTCTT 1197
YS_P._hongwonpyoi_YW36_1____        CACGAGAGTTTCGCGTATGGTCTCCCACTACACTACTCGGTCTCGCTCTT 1198
YS_P._angularis_YW25_8___           CACGAGAGTTTCGCGTATGGTCTCCCACTACACTACTCGGTCTCGCTCTT 1193
```

```
                                  **************************************************

YS_P._acuticollis_YW07_8____      AGCAGCTTAACTACAGTTGATCGGACGGGAAACGGTGCTTTCTGCTAGAT 1246
YS_P._takakuwai_YW54_1____        AGCAGCTTAACTACAGTTGATCGGACGGGAAACGGTGCTTTCTGCTAGAT 1246
YS_P._albisomni_YW19_1____        AGCAGCTTAACTACAGTTGATCGGACGGGAAACGGTGCTTTCTGCTAGAT 1246
YS_P._kawadai_YW53_1____          AGCAGCTTAACTACAGTTGATCGGACGGGAAACGGTGCTTTCTGCTAGAT 1246
YS_P._kawadai_YW18_1____          AGCAGCTTAACTACAGTTGATCGGACGGGAAACGGTGCTTTCTGCTAGAT 1246
YS_P._sue_YW45_1____              AGCAGCTTAACTACAGTTGATCGGACGGGAAACGGTGCTTTCTGCTAGAT 1246
YS_P._sue_YW80_3____              AGCAGCTTAACTACAGTTGATCGGACGGGAAACGGTGCTTTCTGCTAGAT 1246
YS_P._sugitai_YW44_1____          AGCAGCTTAACTACAGTTGATCGGACGGGAAACGGTGCTTTCTGCTAGAT 1233
YS_P._urushiyamai_YW50_1____      AGCAGCTTAACTACAGTTGATCGGACGGGAAACGGTGCTTTCTGCTAGAT 1246
YS_P._delicatulus_YW52_1____      AGCAGCTTAACTACAGTTGATCGGACGGGAAACGGTGCTTTCTGCTAGAT 1247
YS_P._delicatulus_YW60_2____      AGCAGCTTAACTACAGTTGATCGGACGGGAAACGGTGCTTTCTGCTAGAT 1247
YS_P._akitaorum_YW16_1____        AGCAGCTTAACTACAGTTGATCGGACGGGAAACGGTGCTTTCTGCTAGAT 1246
YS_P._hongwonpyoi_YW05_8____      AGCAGCTTAACTACAGTTGATCGGACGGGAAACGGTGCTTTCTGCTAGAT 1248
YS_P._hongwonpyoi_YW24_1____      AGCAGCTTAACTACAGTTGATCGGACGGGAAACGGTGCTTTCTGCTAGAT 1248
YS_P._hongwonpyoi_YW33_8____      AGCAGCTTAACTACAGTTGATCGGACGGGAAACGGTGCTTTCTGCTAGAT 1246
YS_P._hongwonpyoi_YW35_8____      AGCAGCTTAACTACAGTTGATCGGACGGGAAACGGTGCTTTCTGCTAGAT 1246
YS_P._hongwonpyoi_YW37_8____      AGCAGCTTAACTACAGTTGATCGGACGGGAAACGGTGCTTTCTGCTAGAT 1248
YS_P._hongwonpyoi_YW34_8____      AGCAGCTTAACTACAGTTGATCGGACGGGAAACGGTGCTTTCTGCTAGAT 1246
YS_P._hongwonpyoi_YW34_2a___      AGCAGCTTAACTACAGTTGATCGGACGGGAAACGGTGCTTTCTGCTAGAT 1248
YS_P._viridicuprus_YW15_1____     AGCAGCTTAACTACAGTTGATCGGACGGGAAACGGTGCTTTCTGCTAGAT 1247
YS_P._hongwonpyoi_YW36_1____      AGCAGCTTAACTACAGTTGATCGGACGGGAAACGGTGCTTTCTGCTAGAT 1248
YS_P._angularis_YW25_8___         AGCAGCTTAACTACAGTTGATCGGACGGGAAACGGTGCTTTCTGCTAGAT 1243
                                  **************************************************

YS_P._acuticollis_YW07_8____      ATGGCCGCAACCGAAATACATAAGATTCCAGCGTACCATATGGAAACCGG 1296
YS_P._takakuwai_YW54_1____        ATGGCCGCAACCGAAATACATAAGATTCCAGCGTACCATATGGAAACCGG 1296
YS_P._albisomni_YW19_1____        ATGGCCGCAACCGAAATACATAAGATTCCAGCGTACCATATGGAAACCGG 1296
YS_P._kawadai_YW53_1____          ATGGCCGCAACCGAAATACATAAGATTCCAGCGTACCATATGGAAACCGG 1296
YS_P._kawadai_YW18_1____          ATGGCCGCAACCGAAATACATAAGATTCCAGCGTACCATATGGAAACCGG 1296
YS_P._sue_YW45_1____              ATGGCCGCAACCGAAATACATAAGATTCCAGCGTACTATACGGAAACCGG 1296
YS_P._sue_YW80_3____              ATGGCCGCAACCGAAATACATAAGATTCCAGCGTACTATACGGAAACCGG 1296
YS_P._sugitai_YW44_1____          ATGGCCGCAACCGAAATACATAAGATTCCAGCGTACTATACGGAAACCGG 1283
YS_P._urushiyamai_YW50_1____      ATGGCCGCAACCGAAATACATAAGATTCCAGCGTACCATACGGAAACCGG 1296
YS_P._delicatulus_YW52_1____      ATGGCCGCAACCGAAATACATAAGATTCCAGCGTACCATATGGAAACCGG 1297
```

YS_P._delicatulus_YW60_2____ ATGGCCGCAACCGAAATACATAAGATTCCAGCGTACCATATGGAAACCGG 1297
YS_P._akitaorum_YW16_1____ ATGGCCGCAACCGAAATACATAAGATTCCAGCGTACCATATGGAAACCGG 1296
YS_P._hongwonpyoi_YW05_8____ ATGGCCGCAACCGAAATACATAAGATTCCAGCGTACCATATGGAAACCGG 1298
YS_P._hongwonpyoi_YW24_1____ ATGGCCGCAACCGAAATACATAAGATTCCAGCGTACCATATGGAAACCGG 1298
YS_P._hongwonpyoi_YW33_8____ ATGGCCGCAACCGAAATACATAAGATTCCAGCGTACCATATGGAAACCGG 1296
YS_P._hongwonpyoi_YW35_8____ ATGGCCGCAACCGAAATACATAAGATTCCAGCGTACCATATGGAAACCGG 1296
YS_P._hongwonpyoi_YW37_8____ ATGGCCGCAACCGAAATACATAAGATTCCAGCGTACCATATGGAAACCGG 1298
YS_P._hongwonpyoi_YW34_8____ ATGGCCGCAACCGAAATACATAAGATTCCAGCGTACCATATGGAAACCGG 1296
YS_P._hongwonpyoi_YW34_2a___ ATGGCCGCAACCGAAATACATAAGATTCCAGCGTACCATATGGAAACCGG 1298
YS_P._viridicuprus_YW15_1____ ATGGCCGCAACCGAAATACATAAGATTCCAGCGTACCATATGGAAACCGG 1297
YS_P._hongwonpyoi_YW36_1____ ATGGCCGCAACCGAAATACATAAGATTCCAGCGTACCATATGGAAACCGG 1298
YS_P._angularis_YW25_8___ ATGGCCGCAACCGAAATACATAAGATTCCAGCGCACTATATGGAAACCGG 1293
********************************* ** *** *********

YS_P._acuticollis_YW07_8____ GGGG-TCTTAGGTTCATTATTTCGAAATATCAAGGGG-GGGATACCACA- 1343
YS_P._takakuwai_YW54_1____ GGGG-TCTTAGGTTCATTATTTCGAAATATCAAGGGG-GGGATACCACA- 1343
YS_P._albisomni_YW19_1____ GGGG-TCTTAGGTTCATTATTTCGAAATATCAAGGGG-GGGATACCACA- 1343
YS_P._kawadai_YW53_1____ GGGG-TCTTAGGTTCATTATTTCGAAATATCAAGGGG-GGGATACCACA- 1343
YS_P._kawadai_YW18_1____ GGGG-TCTTAGGTTCATTATTTCGAAATATCAAGGGG-GGGATACCACA- 1343
YS_P._sue_YW45_1____ GGGG-TCTTAGGTTCATTATTTCGAAATATCAAGGGG-GGGATACCACA- 1343
YS_P._sue_YW80_3____ GGGG-TCTTAGGTTCATTATTTCGAAATATCAAGGGG-GGGATACCACA- 1343
YS_P._sugitai_YW44_1____ GGGG-TCTTAGGTTCATTATTTCGAAATATCAAGGGG-GGGATACCACA- 1330
YS_P._urushiyamai_YW50_1____ GGGG-TCTTAGGTTCATTATTTCGAAATATCAAGGGG-GGGATACCACA- 1343
YS_P._delicatulus_YW52_1____ GGGGGTCTTAGGTTCATTATTTCGAAATATCAAGGGG-GGGATACCACA- 1345
YS_P._delicatulus_YW60_2____ GGGGGTCTTAGGTTCATTATTTCGAAATATCAAGGGG-GGGATACCACA- 1345
YS_P._akitaorum_YW16_1____ GGGG-TCTTAGGTTCATTATTTCGAAATATCAAGGGG-GGGATACCACA- 1343
YS_P._hongwonpyoi_YW05_8____ GGGG-TCTTAGGTTCATTATTTCGAAAAATCAAGGGG-GGAATACCACA- 1345
YS_P._hongwonpyoi_YW24_1____ GGGG-TCTTAGGTTCATTATTTCGAAAAATCAAGGGG-GGAATACCACA- 1345
YS_P._hongwonpyoi_YW33_8____ GGGG-TCTTAGGTTCATTATTTCGAAAAATCAAGGGG-GGAATACCACA- 1343
YS_P._hongwonpyoi_YW35_8____ GGGG-TCTTAGGTTCATTATTTCGAAAAATCAAGGGG-GGAATACCACA- 1343
YS_P._hongwonpyoi_YW37_8____ GGGG-TCTTAGGTTCATTATTTCGAAAAATCAAGGGG-GGAATACCACA- 1345
YS_P._hongwonpyoi_YW34_8____ GGGG-TCTTAGGTTCATTATTTCGAAAAATCAAGGGG-GGAATACCACAA 1344
YS_P._hongwonpyoi_YW34_2a___ GGGG-TCTTAGGTTCATTATTTCGAAAAATCAAGGGG-GGAATACCACAA 1346
YS_P._viridicuprus_YW15_1____ GGGG-TCTTAGGTTCATTATTTCGAAAAATCAAGGGG-GGAATACCACA- 1344
YS_P._hongwonpyoi_YW36_1____ GGGG-TCTTAGGTTCATTATTTCGAAAAATCAAGGGG-GGAATACCACA- 1345
YS_P._angularis_YW25_8___ GGGG-TCTTAGGTTCATTATTTCGAAAAATTAAGGGGAGGAATACCACA- 1341

```
                                    **** ********************** ** ****** ** *********

YS_P._acuticollis_YW07_8____        TATTTGGGGATTCAGGTATGTTAAGTATGATGAGGAACCTACACAAGCCT 1393
YS_P._takakuwai_YW54_1____          TATTTGGGGATTCAGGTATGTTAAGTATGATGAGGAACCTACACAAGCCT 1393
YS_P._albisomni_YW19_1____          TATTTGGGGATTCAGGTATGTTAAGTATGATGAGGAACCTACACAAGCCT 1393
YS_P._kawadai_YW53_1____            TATTTGGGGATTCAGGTATGTCAAGTATGATGAGGAACCTACACAAGCCT 1393
YS_P._kawadai_YW18_1____            TATTTGGGGATTCAGGTATGTTAAGTATGATGAGGAACCTACACAAGCCT 1393
YS_P._sue_YW45_1____                TATTTGGGGATTCAGGTATGTTAAGTATGACGAGGAACCTACACAAGCCT 1393
YS_P._sue_YW80_3____                TATTTGGGGATTCAGGTATGTTAAGTATGACGAGGAACCTACACAAGCCT 1393
YS_P._sugitai_YW44_1____            TATTTGGGGATTCAGGTATGTTAAGTATGACGAGGAACCTACACAAGCCT 1380
YS_P._urushiyamai_YW50_1____        TATTTGGGGATTCAGGTATGTTAAGTATGATGAGGAACCTACACAAGCCT 1393
YS_P._delicatulus_YW52_1____        TATTTGGGGATCCAGGTATGTTAAGTATGATGAGGAACCTACACAAGCCT 1395
YS_P._delicatulus_YW60_2____        TATTTGGGGATTCAGGTATGTTAAGTATGATGAGGAACCTACACAAGCCT 1395
YS_P._akitaorum_YW16_1____          TATTTGGGGATTCAGGTATGTTAAGTATGATGAGGAACCTACACAAGCCT 1393
YS_P._hongwonpyoi_YW05_8____        TATTTGGGGATTCAGGTATGTTAAGTATGATGAGGAACCTACACAAGCCT 1395
YS_P._hongwonpyoi_YW24_1____        TATTTGGGGATTCAGGTATGTTAAGTATGATGAGGAACCTACACAAGCCT 1395
YS_P._hongwonpyoi_YW33_8____        TATTTGGGGATTCAGGTATGTTAAGTATGATGAGGAACCTACACAAGCCT 1393
YS_P._hongwonpyoi_YW35_8____        TATTTGGGGATTCAGGTATGTTAAGTATGATGAGGAACCTACACAAGCCT 1393
YS_P._hongwonpyoi_YW37_8____        TATTTGGGGATTCAGGTATGTTAAGTATGATGAGGAACCTACACAAGCCT 1395
YS_P._hongwonpyoi_YW34_8____        TATTTGGGGATTCAGGTATGTTAAGTATGATGAGGAACCTACACAAGCCT 1394
YS_P._hongwonpyoi_YW34_2a___        TATTTGGGGATTCAGGTATGTTAAGTATGATGAGGAACCTACACAAGCCT 1396
YS_P._viridicuprus_YW15_1____       TATTTGGGGATTCAGGTATGTTAAGTATGATGAGGAACCTACACAAGCCT 1394
YS_P._hongwonpyoi_YW36_1____        TATTTGGGGATTCAGGTATGTTAAGTATGATGAGGAACCTACACAAGCTT 1395
YS_P._angularis_YW25_8___           TATTTGGGGATTCAGGTATGCTAAGTATGATCAGGAACCTACACATGCCT 1391
                                    *********** ********  ********  ************* ** *

YS_P._acuticollis_YW07_8____        ACGAGTACATACTGGACTGAGATAGAGAAAGGAATAGAGGACTGGAAGAA 1443
YS_P._takakuwai_YW54_1____          ACGAGTACATACTGGACTGAGATAGAGAAAGGAATAGAGGACTGGAAGAA 1443
YS_P._albisomni_YW19_1____          ACGAGTACATACTGGACTGAGATAGAGAAAGGAATAGAGGACTGGAAGAA 1443
YS_P._kawadai_YW53_1____            ACGAGTACATACTGGACTGAGATAGAGAAAGGAATAGAGGACTGGAAGAA 1443
YS_P._kawadai_YW18_1____            ACGAGTACATACTGGACTGAGATAGAGAAAGGAATAGAGGACTGGAAGAA 1443
YS_P._sue_YW45_1____                ACAAGTACATACTGGACAGAGATAGAGAAAGGAATAGAGGATTGGAAGAA 1443
YS_P._sue_YW80_3____                ACAAGTACATACTGGACAGAGATAGAGAAAGGAATAGAGGATTGGAAGAA 1443
YS_P._sugitai_YW44_1____            ACAAGTACATACTGGACAGAGATAGAGAAAGGAATAGAGGATTGGAAGAA 1430
YS_P._urushiyamai_YW50_1____        ACAAGTACATACTGGACAGAGATAGAGAAAGGAATAGAGGATTGGAAGAA 1443
YS_P._delicatulus_YW52_1____        ACAAGTACATACTGGACAGAGATAGAGAAAGGAATAGAGGATTGGAAGAA 1445
```

```
YS_P._delicatulus_YW60_2____        ACAAGTACATACTGGACAGAGATAGAGAAAGGAATAGAGGATTGGAAGAA 1445
YS_P._akitaorum_YW16_1____          ACAAGTACATACTGGACAGAGATAGAGAAAGGAATAGAGGATTGGAAGAA 1443
YS_P._hongwonpyoi_YW05_8____        ACAAGTACATACTGGACAGAGATAGAGAAAGGAATAGAGTATTGGAAGAA 1445
YS_P._hongwonpyoi_YW24_1____        ACAAGTACATACTGGACAGAGATAGAGAAAGGAATAGAGTATTGGAAGAA 1445
YS_P._hongwonpyoi_YW33_8____        ACAAGTACATACTGGACAGAGATAGAGAAAGGAATAGAGTATTGGAAGAA 1443
YS_P._hongwonpyoi_YW35_8____        ACAAGTACATACTGGACAGAGATAGAGAAAGGAATAGAGTATTGGAAGAA 1443
YS_P._hongwonpyoi_YW37_8____        ACAAGTACATACTGGACAGAGATAGAGAAAGGAATAGAGTATTGGAAGAA 1445
YS_P._hongwonpyoi_YW34_8____        ACAAGTACATACTGGACAGAGATAGAGAAAGGAATAGAGTATTGGAAGAA 1444
YS_P._hongwonpyoi_YW34_2a___        ACAAGTACATACTGGACAGAGATAGAGAAAGGAATAGAGTATTGGAAGAA 1446
YS_P._viridicuprus_YW15_1____       ACAAGTACATACTGGACAGAGATAGAGAAAGGAATAGAGTATTGGAAGAA 1444
YS_P._hongwonpyoi_YW36_1____        ACAAGTACATACTGGACAGAGATAGAGAAAGGAATAGAGTATTGGAAGAA 1445
YS_P._angularis_YW25_8___           ACAAGTACATACTGAACAGAGAAAGAGAAAGGAATAGAGGATTGGAAGAA 1441
                                    ** *********** ** **** **************** * ********

YS_P._acuticollis_YW07_8____        ATGTAATTTATATTTGTAAAATTGACGGAAATGTGACAAAGAATGGAATA 1493
YS_P._takakuwai_YW54_1____          ATGTAATTTATATTTGTAAAATTGACGGAAATGTGACAAAGAATGGAATA 1493
YS_P._albisomni_YW19_1____          ATGTAATTTATATTTGTAAAATTGACGGAAATGTGACAAAGAATGGAATA 1493
YS_P._kawadai_YW53_1____            ATGTAATTTATATTTGTAAAATTGACGGAAATGTGACAAAGAATGGAATA 1493
YS_P._kawadai_YW18_1____            ATGTAATTTATATTTGTAAAATTGACGGAAATGTGACAAAGAATGGAATA 1493
YS_P._sue_YW45_1____                ATGTAATTTATATTTGTAAAATTGACGGAAATGTGACAAAGAATGGAATA 1493
YS_P._sue_YW80_3____                ATGTAATTTATATTTGTAAAATTGACGGAAATGTGACAAAGAATGGAATA 1493
YS_P._sugitai_YW44_1____            ATGTAATTTATATTTGCAAAATTGACGGAAATGTGACAAAGAATGGAATA 1480
YS_P._urushiyamai_YW50_1____        ATGTAATTTATATTTGTAAAATTGACGGAAATGTGACAAAGAATGGAATA 1493
YS_P._delicatulus_YW52_1____        ATGTAATTTATATTTGTAAAATTGACGGAAATGTGACAAAGAATGGAATA 1495
YS_P._delicatulus_YW60_2____        ATGTAATTTATATTTGTAAAATTGACGGAAATGTGACAAAGAATGGAATA 1495
YS_P._akitaorum_YW16_1____          ATGTAATTTATATTTGTAAAATTGACGGAAATGTGACAAAGAATGGAATA 1493
YS_P._hongwonpyoi_YW05_8____        ATGTAATTTATATTTGTAAAATTGACGGAAATGTGACAAAGAATGGAATA 1495
YS_P._hongwonpyoi_YW24_1____        ATGTAATTTATATTTGTAAAATTGACGGAAATGTGACAAAGAATGGAATA 1495
YS_P._hongwonpyoi_YW33_8____        ATGTAATTTATATTTGTAAAATTGACGGAAATGTGACAAAGAATGGAATA 1493
YS_P._hongwonpyoi_YW35_8____        ATGTAATTTATATTTGTAAAATTGACGGAAATGTGACAAAGAATGGAATA 1493
YS_P._hongwonpyoi_YW37_8____        ATGTAATTTATATTTGTAAAATTGACGGAAATGTGACAAAGAATGGAATA 1495
YS_P._hongwonpyoi_YW34_8____        ATGTAATTTATATTTGTAAAATTGACGGAAATGTGACAAAGAATGGAATA 1494
YS_P._hongwonpyoi_YW34_2a___        ATGTAATTTATATTTGTAAAATTGACGGAAATGTGACAAAGAATGGAATA 1496
YS_P._viridicuprus_YW15_1____       ATGTAATTTATATTTGTAAAATTGACGGAAATGTGACAAAGAATGAAATA 1494
YS_P._hongwonpyoi_YW36_1____        ATGTAATTTATATTTGTAAAATTGACGGAAATGTGACAAAGAATGGAATA 1495
YS_P._angularis_YW25_8___           ATGTAATTTATATTTGTAAAATTGACGGAAATGTGACAAAGAATGGAATA 1491
```

```
                                    **************** **************************** ****

YS_P._acuticollis_YW07_8____        TAGATGATTGTGTTATAGGAAGACAAAGAGGTGGACAAGGCGGGTATTTA 1543
YS_P._takakuwai_YW54_1____          TAGATGATTGTGTTATAGGAAGACAAAGAGGTGGACAAGGCGGGTATTTA 1543
YS_P._albisomni_YW19_1____          TAGATGATTGTGTTATAGGAAGACAAAGAGGTGGACAAGGCGGGTATTTA 1543
YS_P._kawadai_YW53_1____            TAGATGATTGTGTTATAGGAAGACAAAGAGGTGGACAAGGCGGGTATTTA 1543
YS_P._kawadai_YW18_1____            TAGATGATTGTGTTATAGGAAGACAAAGAGGTGGACAAGGCGGGTATTTA 1543
YS_P._sue_YW45_1____                TAGATGATTGTGTTATAGGAAGACAAAGAGGTGGACAAGGCGGGTATTTA 1543
YS_P._sue_YW80_3____                TAGATGATTGTGTTATAGGAAGACAAAGAGGTGGACAAGGCGGGTATTTA 1543
YS_P._sugitai_YW44_1____            TAGATGATTGTGTTATAGGAAGACAAAGAGGTGGACAAGGCGGGTATTTA 1530
YS_P._urushiyamai_YW50_1____        TAGATGATTGTGTTATAGGAAGACAAAGAGGTGGACAAGGCGGGTATTTA 1543
YS_P._delicatulus_YW52_1____        TAGATGATTGTGTTATAGGAAGACAAAGAGGTGGACAAGGCGGGTATTTA 1545
YS_P._delicatulus_YW60_2____        TAGATGATTGTGTTATAGGAAGACAAAGAGGTGGACAAGGCGGGTATTTA 1545
YS_P._akitaorum_YW16_1____          TAGATGATTGTGTTATAGGAAGACAAAGAGGTGGACAAGGCGGGTATTTA 1543
YS_P._hongwonpyoi_YW05_8____        TAGATGATTGTGTTATAGGAAGACAAAGAGGTGGACAAGGCGGGTATTTA 1545
YS_P._hongwonpyoi_YW24_1____        TAGATGATTGTGTTATAGGAAGACAAGGAGGTGGACAAGGCGGGTATTTA 1545
YS_P._hongwonpyoi_YW33_8____        TAGATGATTGTGTTATAGGAAGACAAAGAGGTGGACAAGGCGGGTATTTA 1543
YS_P._hongwonpyoi_YW35_8____        TAGATGATTGTGTTATAGGAAGACAAAGAGGTGGACAAGGCGGGTATTTA 1543
YS_P._hongwonpyoi_YW37_8____        TAGATGATTGTGTTATAGGAAGACAAAGAGGTGGACAAGGCGGGTATTTA 1545
YS_P._hongwonpyoi_YW34_8____        TAGATGATTGTGTTATAGGAAGACAAAGAGGTGGACAAGGCGGGTATTTA 1544
YS_P._hongwonpyoi_YW34_2a___        TAGATGATTGTGTTATAGGAAGACAAAGAGGTGGACAAGGCGGGTATTTA 1546
YS_P._viridicuprus_YW15_1____       TAGATGATTGTGTTATAGGAAGACAAAGAGGTGGACAAGGCGGGTATTTA 1544
YS_P._hongwonpyoi_YW36_1____        TAGATGATTGTGTTATAGGAAGACAAAGAGGCGGACAAGGCGGGTATTTA 1545
YS_P._angularis_YW25_8___           TAGATGATTGTGTTATAGGAAGACAAAGAGGTGGTCAAGGCGGGTATTTA 1541
                                    ************************* ***** ** ***************

YS_P._acuticollis_YW07_8____        TAAGTAGAGGGTAGACGAAGTGGAAACTGATGGAAGCAAATGAGGCTTGA 1593
YS_P._takakuwai_YW54_1____          TAAGTAGAGGGTAGACGAAGTGGAAACTGATGGAAGCAAATGAGGCTTGA 1593
YS_P._albisomni_YW19_1____          TAAGTAGAGGGTAGACGAAGTGGAAACTGATGGAAGCAAATGAGGCTTGA 1593
YS_P._kawadai_YW53_1____            TAAGTAGAGGGTAGACGAAGTGGAAACTGATGGAAGCAAATGAGGCTTGA 1593
YS_P._kawadai_YW18_1____            TAAGTAGAGGGTAGACGAAGTGGAAACTGATGGAAGCAAATGAGGCTTGA 1593
YS_P._sue_YW45_1____                TAAGTAAAGGGTAGACGAAGTGGAAACTGATGGAAGCAAATGAGGCTTGA 1593
YS_P._sue_YW80_3____                TAAGTAAAGGGTAGACGAAGTGGAAACTGATGGAAGCAAATGAGGCTTGA 1593
YS_P._sugitai_YW44_1____            TAAGTAAAGGGTAGACGAAGTGGAAACTGATGGAAGCAAATGAGGCTTGA 1580
YS_P._urushiyamai_YW50_1____        TAAGTAGAGGGTAGACGAAGTGGAAACTGATGGAAGCAAATGAGGCTTGA 1593
YS_P._delicatulus_YW52_1____        TAAGTAGAGGGTAGACGAAGTGGAAACTGATGGAAGCATATGAGGCTTGA 1595
```

```
YS_P._delicatulus_YW60_2____        TAAGTAGAGGGTAGACGAAGTGGAAACTGATGGAAGCATATAAGGCTTGA 1595
YS_P._akitaorum_YW16_1____          TAAGTAGAGGGTAGACGAAGTGGAAACTGATGGAAGCAAATGAGGCTTGA 1593
YS_P._hongwonpyoi_YW05_8____        TAAGTAGAGGGTAGACGAAGTGGAAACTGATGGAAGCTAATGAGGCTTGA 1595
YS_P._hongwonpyoi_YW24_1____        TAAGTAGAGGGTAGACGAAGTGGAAACTGATGGAAGCTAATGAGGCTTGA 1595
YS_P._hongwonpyoi_YW33_8____        TAAGTAGAGGGTAGACGAAGTGGAAACTGATGGAAGCTAATGAGGCTTGA 1593
YS_P._hongwonpyoi_YW35_8____        TAAGTAGAGGGTAGACGAAGTGGAAACTGATGGAAGCTAATGAGGCTTGA 1593
YS_P._hongwonpyoi_YW37_8____        TAAGTAGAGGGTAGACGAAGTGGAAACTGATGGAAGCTAATGAGGCTTGA 1595
YS_P._hongwonpyoi_YW34_8____        TAAGTAGAGGGTAGACGAAGTGGAAACTGATGGAAGCTAATGAGGCTTGA 1594
YS_P._hongwonpyoi_YW34_2a___        TAAGTAGAGGGTAGACGAAGTGGAAACTGATGGAAGCTAATGAGGCTTGA 1596
YS_P._viridicuprus_YW15_1____       TAAGTAGAGGGTAGACGAAGTGGAAACTGATGGAAGCTAATGAGGCTTGA 1594
YS_P._hongwonpyoi_YW36_1____        TAAGTAGAGGGTAGACGAAGTGGAAACTGATGGAAGCTAATGAGGCTTGA 1595
YS_P._angularis_YW25_8___           TAAGTAGAGGGTGGACGAAGTGGAAACTGATGGAAGCAAACGAAGCTTGA 1591
                                    ****** ***** ************************  *  * ******

YS_P._acuticollis_YW07_8____        CACAAAACACGGTTGCAACAAGAATAACAAACAAAGGAA--TGTGCATGA 1641
YS_P._takakuwai_YW54_1____          CACAAAACACGGTTGCAACAAGAATAACAAACAAAGGAA--TGTGCATGA 1641
YS_P._albisomni_YW19_1____          CACAAAACACGGTTGCAACAAGAATAACAAACAAAGGAA--TGTGCATGA 1641
YS_P._kawadai_YW53_1____            CACAAAACACGGTTGCAACAAGAATAACAAACAAAGGAA--TGTGCATGA 1641
YS_P._kawadai_YW18_1____            CACAAAACACGGTTGCAACAAGAATAACAAACAAAGGAA--TGTGCATGA 1641
YS_P._sue_YW45_1____                CACAAAACACGGTTGCAACAAGAATAACAAACAAAGGAA--TGTGCATGA 1641
YS_P._sue_YW80_3____                CACAAAACACGGTTGCAACAAGAATAACAAACAAAGGAA--TGTGCATGA 1641
YS_P._sugitai_YW44_1____            CACAAAACACGGTTGCAACAAGAATAACAAACAAAGGAA--TGTGCATGA 1628
YS_P._urushiyamai_YW50_1____        CACAAAACACGGTTGCAACAAGAATAACAAACAAAGGAA--TGTGCATGA 1641
YS_P._delicatulus_YW52_1____        CACAAAACACGGTTGCAACAAGAATAACAAACAAAGGAA--TGTGCATGA 1643
YS_P._delicatulus_YW60_2____        CACAAAACACGGTTGCAACAAGAATAACAAACAAAGGAA--TGTGCATGA 1643
YS_P._akitaorum_YW16_1____          CACAAAACACGGTTGCAACAAGAATAACAAACAAAGGAAAATGTGCATGA 1643
YS_P._hongwonpyoi_YW05_8____        CACAAAACACGGTTGCAACAAGAATAACAAACAAAGGAA--TGTGCATGA 1643
YS_P._hongwonpyoi_YW24_1____        CACAAAACACGGTTGCAACAAGAATAACAAACAAAGGAA--TGTGCATGA 1643
YS_P._hongwonpyoi_YW33_8____        CACAAAACACGGTTGCAACAAGAATAACAAACAAAGGAA--TGTGCATGA 1641
YS_P._hongwonpyoi_YW35_8____        CACAAAACACGGTTGCAACAAGAATAACAAACAAAGGAA--TGTGCATGA 1641
YS_P._hongwonpyoi_YW37_8____        CACAAAACACGGTTGCAACAAGAATAACAAACAAAGGAA--TGTGCATGA 1643
YS_P._hongwonpyoi_YW34_8____        CACAAAACACGGTTGCAACAAGAATAACAAACAAAGGAA--TGTGCATGA 1642
YS_P._hongwonpyoi_YW34_2a___        CACAAAACACGGTTGCAACAAGAATAACAAACAAAGGAA--TGTGCATGA 1644
YS_P._viridicuprus_YW15_1____       CACAAAACACGGTTGCAACAAGAATAACAAACAAAGGAA--TGTGCATGA 1642
YS_P._hongwonpyoi_YW36_1____        CACAAAACACGGTTGCAACAAGAATAACAAACAAAGGAA--TGTGCATGA 1643
YS_P._angularis_YW25_8___           CACAAAACACGGTTGCAACAAGAATAACAAACAAAGGAA--TGTGCATGA 1639
```

```
                                  *************************************** *********

YS_P._acuticollis_YW07_8____      GCGTGTTCAGCAGGTTAGGCAAAAAAAATTTTGCAACATAGTGCGAACTG 1691
YS_P._takakuwai_YW54_1____        GCGTGTTCAGCAGGTTAGGCAAAAAAAATTTTGCAACATAGTGCGAACTG 1691
YS_P._albisomni_YW19_1____        GCGTGTTCAGCAGGTTAGGCAAAAAAAATTTTGCAACATAGTGCGAACTG 1691
YS_P._kawadai_YW53_1____          GCGTGTTCAGCAGGTTAGGCAAAAAAAATTTTGCAACATAGTGCGAACTG 1691
YS_P._kawadai_YW18_1____          GCGTGTTCAGCAGGTTAGGCAAAAAAAATTTTGCAACATAGTGCGAACTG 1691
YS_P._sue_YW45_1____              GCGTGTTCAGCAGGTTAGGCAAAAAAAATTTTGCAACATAGTGCGAACTG 1691
YS_P._sue_YW80_3____              GCGTGTTCAGCAGGTTAGGCAAAAAAAATTTTGCAACATAGTGCGAACTG 1691
YS_P._sugitai_YW44_1____          GCGTGTTCAGCAGGTTAGGCAAAAAAAATTTTGCAACATAGTGCGAACTG 1678
YS_P._urushiyamai_YW50_1____      GCGTGTTCAGCAGGTTAGGCAAAAAAAATTTTGCAACATAGTGCGAACTG 1691
YS_P._delicatulus_YW52_1____      GCGTGTTCAGCAGGTTAGGCAAAAAAAATTT-GCAACATAGTGCGAACTG 1692
YS_P._delicatulus_YW60_2____      GCGTGTTCAGCAGGTTAGGCAAAAAAAATTT-GCAACATAGTGCGAACTG 1692
YS_P._akitaorum_YW16_1____        GCGTGTTCAGCAGGTTAGGCAAAAAAAATTT-GCAACATAGTGCGAACTG 1692
YS_P._hongwonpyoi_YW05_8____      GCGTGTTCAGCAGGTTAGGCAAAAAAAATTTTGCAACATAGTGCGAACTG 1693
YS_P._hongwonpyoi_YW24_1____      GCGTGTTCAGCAGGTTAGGCAAAAAAAATTTTGCAACATAGTGCGAACTG 1693
YS_P._hongwonpyoi_YW33_8____      GCGTGTTCAGCAGGTTAGGCAAAAAAAATTTTGCAACATAGTGCGAACTG 1691
YS_P._hongwonpyoi_YW35_8____      GCGTGTTCAGCAGGTTAGGCAAAAAAAATTTTGCAACATAGTGCGAACTG 1691
YS_P._hongwonpyoi_YW37_8____      GCGTGTTCAGCAGGTTAGGCAAAAAAAATTTTGCAACATAGTGCGAACTG 1693
YS_P._hongwonpyoi_YW34_8____      GCGTGTTCAGCAGGTTAGGCAAAAAAAATTTTGCAACATAGTGCGAACTG 1692
YS_P._hongwonpyoi_YW34_2a___      GCGTGTTCAGCAGGTTAGGCAAAAAAAATTTTGCAACATAGTGCGAACTG 1694
YS_P._viridicuprus_YW15_1____     GCGTGTTCAGCAGGTTAGGCAAAAAAAATTTTGCAACATAGTGCGAACTG 1692
YS_P._hongwonpyoi_YW36_1____      GCGTGTTCAGCAGGTTAGGCAAAAAAAATTTTGCAACATAGTGCGAACTG 1693
YS_P._angularis_YW25_8___         GCGTGTTCAGCAGGTTAGGCAAAAAAAATTTTGCAACATAGTGCGAACTG 1689
                                  ******************************* ******************

YS_P._acuticollis_YW07_8____      GACGCGAATGGCATAAATATGAGTGGAAGGTAGAATGAGAATGAAGAGAG 1741
YS_P._takakuwai_YW54_1____        GACGCGAATGGCATAAATATG--TGGAAGGTAGAATGAGAATGAAGAGAG 1739
YS_P._albisomni_YW19_1____        GACGCGAATGGCATAAATATGAGTGGAAGGTAGAATGAGAATGAAGAGAG 1741
YS_P._kawadai_YW53_1____          GACGCGAATGGCATAAATATGAGTGGAAGGTAGAATGAGAATGAAGAGAG 1741
YS_P._kawadai_YW18_1____          GACGCGAATGGCATAAATATGAGTGGAAGGTAGAATGAGAATGAAGAGAG 1741
YS_P._sue_YW45_1____              GACGCGAATGGCATAAATATGAGTGGAAGGTAGAATGAGAATGAAGAGAG 1741
YS_P._sue_YW80_3____              GACGCGAATGGCATAAATATGAGTGGAAGGTAGAATGAGAATGAAGAGAG 1741
YS_P._sugitai_YW44_1____          GACGCGAATGGCATAAATATGAGTGGAAGGTAGAATGAGAATGAAGAGAG 1728
YS_P._urushiyamai_YW50_1____      GACGCGAATGGCATAAATATGAGTGGAAGGTAGAATGAGAATGAAGAGAG 1741
YS_P._delicatulus_YW52_1____      GACGCGAATGGCATAAATATGAGTGGAAGGTAGAATGAGAATGAAGAGAG 1742
```

```
YS_P._delicatulus_YW60_2____        GACGCGAATGGCATAAATATGAGTGGAAGGTAGAATGAGAATGAAGAGAG 1742
YS_P._akitaorum_YW16_1____          GACGCGAATGGCATAAATATGAGTGGAAGGTAGAATGAGGATGAAGAGAG 1742
YS_P._hongwonpyoi_YW05_8____        GACGCGAATGGCATAAATATGAGTGGAAGGTAGAATGAGAGTGAAGAGAG 1743
YS_P._hongwonpyoi_YW24_1____        GACGCGAATGGCATAAATATGAGTGGAAGGTAGAATGAGAGTGAAGAGAG 1743
YS_P._hongwonpyoi_YW33_8____        GACGCGAATGGCATAAATATGAGTGGAAGGTAGAATGAGAGTGAAGAGAG 1741
YS_P._hongwonpyoi_YW35_8____        GACGCGAATGGCATAAATATGAGTGGAAGGTAGAATGAGAGTGAAGAGAG 1741
YS_P._hongwonpyoi_YW37_8____        GACGCGAATGGCATAAATATGAGTGGAAGGTAGAATGAGAGTGAAGAGAG 1743
YS_P._hongwonpyoi_YW34_8____        GACGCGAATGGCATAAATATGAGTGGAAGGTAGAATGAGAGTGAAGAGAG 1742
YS_P._hongwonpyoi_YW34_2a___        GACGCGAATGGCATAAATATGAGTGGAAGGTAGAATGAGAGTGAAGAGAG 1744
YS_P._viridicuprus_YW15_1____       GACGCGAATGGCATAAATATGAGTGGAAGGTAGAATGAGAGTGAAGAGAG 1742
YS_P._hongwonpyoi_YW36_1____        GACGCGAATGGCATAAATATGAGTGGAAGGTAGAATGAGAGTGAAGAGAG 1743
YS_P._angularis_YW25_8___           GACGCGAATAGCATAAATATGGGTGGAAGGTAGAATGAGAATGAAGAGAG 1739
                                    ********* ***********  ****************  *********

YS_P._acuticollis_YW07_8____        AACGGGGATGAAAGAGAAAGGCATATATATGGAAATGGGGGAGAGATATG 1791
YS_P._takakuwai_YW54_1____          AACGGGGATGAAAGAGAAAGGCATATATATGGAAATGGGGGAGAGATATG 1789
YS_P._albisomni_YW19_1____          AACGGGGATGAAAGAGAAAGGCATATATATGGAAATGGGGGAGAGATATG 1791
YS_P._kawadai_YW53_1____            AACGGGGATGAAAGAGAAAGGCATATATATGGAAATGGGGGAGAGATATG 1791
YS_P._kawadai_YW18_1____            AACGGGGATGAAAGAAAAAGGCATATATATGGAAATGGGGGAGAGATATG 1791
YS_P._sue_YW45_1____                AACGGAGATGAAAGAGAAAGGCATATATATGGAAATGGGGGAGAGATATG 1791
YS_P._sue_YW80_3____                AACGGAGATGAAAGAGAAAGGCATATATATGGAAATGGGGGAGAGATATG 1791
YS_P._sugitai_YW44_1____            AACGGAGATGAAAGAGAAAGGCATATATATGGAAATGGGGGAGAGATATG 1778
YS_P._urushiyamai_YW50_1____        AACGGGGATGAAAGAGAAAGGCATATATATGGAAATGGGGGAGAGATATG 1791
YS_P._delicatulus_YW52_1____        AACGGGGATGAAAGAGAAAGGCATATATATGGAAATGGGGGAGAGATATG 1792
YS_P._delicatulus_YW60_2____        AACGGGGATGAAAGAGAAAGGCATATATATGGAAATGGGGGAGAGATATG 1792
YS_P._akitaorum_YW16_1____          AACGGGGATGAAAGAGAAAGGCATATATATGGAAATGGGGGAGAGATATG 1792
YS_P._hongwonpyoi_YW05_8____        AACGGGGATGAAAGAGAAAGGCATATATATGGAAATGGGGGAGAGATATG 1793
YS_P._hongwonpyoi_YW24_1____        AACGGGGATGAAAGAGAAAGGCATATATATGGAAATGGGGGAGAGATATG 1793
YS_P._hongwonpyoi_YW33_8____        AACGGGGATGAAAGAGAAAGGCATATATATGGAAATGGGGGAGAGATATG 1791
YS_P._hongwonpyoi_YW35_8____        AACGGGGATGAAAGAGAAAGGCATATATATGGAAATGGGGGAGAGATATG 1791
YS_P._hongwonpyoi_YW37_8____        AACGGGGATGAAAGAGAAAGGCATATATATGGAAATGGGGGAGAGATATG 1793
YS_P._hongwonpyoi_YW34_8____        AACGGGGATGAAAGAGAAAGGCATATATATGGAAATGGGGGAGAGATATG 1792
YS_P._hongwonpyoi_YW34_2a___        AACGGGGATGAAAGAGAAAGGCATATATATGGAAATGGGGGAGAGATATG 1794
YS_P._viridicuprus_YW15_1____       AACGGGGATGAAAGAGAAAGGCATATATATGGAAATGGGGGAGAGATATG 1792
YS_P._hongwonpyoi_YW36_1____        AACGGGGATGAAAGAGAAAGGCATATATATGGAAATGGGGGAGAGATATG 1793
YS_P._angularis_YW25_8___           AACGGGGATGAAAGAAGAAGGCATATATATGGAAATGGAGGAGAGATGTG 1789
```

```
                                    ***** *********  ********************* ******** **

YS_P._acuticollis_YW07_8____        GGAGATGAAGATGGATGTTGGCGATTGGGGTGTGAAGAGAAGAGTATGAA 1841
YS_P._takakuwai_YW54_1____          GGAGATGAAGATGGATGTTGGCGATTGGGGTGTGAAGAGAAGAGTATGAA 1839
YS_P._albisomni_YW19_1____          GGAGATGAAGATGGATGTTGGCGATTGGGGTGTGAAGAGAAGAGTATGAA 1841
YS_P._kawadai_YW53_1____            GGAGATGAAGATGGATGTTGGCGATTGGGGTGTGAAGAGAAGAGTATGAA 1841
YS_P._kawadai_YW18_1____            GGAGATGAAGATGGATGTTGGCGATTGGGGTGTGAAGAGAAGAGTATGAA 1841
YS_P._sue_YW45_1____                GGAGATGAAGATGGATGTTGGCGATTGGGGTGTGAAGAGAAGAGTATGAA 1841
YS_P._sue_YW80_3____                GGAGATGAAGATGGATGTTGGCGATTGGGGTGTGAAGAGAAGAGTATGAA 1841
YS_P._sugitai_YW44_1____            GGAGATGAAGATGGATGTTGGCGATTGGGGTGTGAAGAGAAGAGTATGAA 1828
YS_P._urushiyamai_YW50_1____        GGAGATGAAGATGGATGTTGGCGATTGGGGTGTGAAGAGAAGAGTATGAA 1841
YS_P._delicatulus_YW52_1____        GGAGATGAAGAAGGATGTTGGCGATTGGGGTGTGAAGAGAAGAGTATGAA 1842
YS_P._delicatulus_YW60_2____        GGAGATGAAGATGGATGTTGGCGATTGGGGTGTGAAGAGAAGAGTATGAA 1842
YS_P._akitaorum_YW16_1____          GGAGATGAAGATGGATGTTGGCGATTGGGGTGTGAAGAGAACAGTATGAA 1842
YS_P._hongwonpyoi_YW05_8____        GGAGATGAAGATGGATGTTGGCGATTGGGGTGTGAAGAGAAGAGTGTGAA 1843
YS_P._hongwonpyoi_YW24_1____        GGAGATGAAGATGGATGTTGGCGATTGGGGTGTGAAGAGAAGAGTGTGAA 1843
YS_P._hongwonpyoi_YW33_8____        GGAGATGAAGATGGATGTTGGCGATTGGGGTGTGAAGAGAAGAGTGTGAA 1841
YS_P._hongwonpyoi_YW35_8____        GGAGATGAAGATGGATGTTGGCGATTGGGGTGTGAAGAGAAGAGTGTGAA 1841
YS_P._hongwonpyoi_YW37_8____        GGAGATGAAGATGGATGTTGGCGATTGGGGTGTGAAGAGAAGAGTGTGAA 1843
YS_P._hongwonpyoi_YW34_8____        GGAGATGAAGATGGATGTTGGCGATTGGGGTGTGAAGAGAAGAGTATGAA 1842
YS_P._hongwonpyoi_YW34_2a___        GGAGATGAAGATGGATGTTGGCGATTGGGGTGTGAAGAGAAGAGTATGAA 1844
YS_P._viridicuprus_YW15_1____       GGAGATGAAGATGGATGTTGGCGATTGGGGTGTGAAGAGAAGAGTATGAA 1842
YS_P._hongwonpyoi_YW36_1____        GGAGATGAAGATGGATGTTGGCGATTGGGGTGTGAAGAGAAGAGTATGAA 1843
YS_P._angularis_YW25_8___           GGAGATGAAGATGGATATTGGCGATTGGGGTGTGAAGAGAAGAGTATGGA 1839
                                    *********** **** ************************ *** ** *

YS_P._acuticollis_YW07_8____        CGAGCAATGAGCAGTGGAGAGAGAACAGGGTAGCGGAAACAGAGAGAGAA 1891
YS_P._takakuwai_YW54_1____          CGAGCAATGAGCAGTGGAGAGAGAACAGGGTAGCGGAAACAGAGAGAGAA 1889
YS_P._albisomni_YW19_1____          CGAGCAATGAGCAGTGGAGAGAGAACAGGGTAGCGGAAACAGAGAGAGAA 1891
YS_P._kawadai_YW53_1____            CGAGCAATGAGCAGTGGAGAGAGAACAGGGTAGCGGAAACAGAGAGAGAA 1891
YS_P._kawadai_YW18_1____            CGAGCAATGAGCAGTGGAGAGAGAACAGGGTAGCGGAAACAGAGAGAGAA 1891
YS_P._sue_YW45_1____                CGAGCAATGAGCAGTGGAGAGAGAACAGGGTAGCGGAAACAGAGAGAGAA 1891
YS_P._sue_YW80_3____                CGAGCAATGAGCAGTGGAGAGAGAACAGGGTAGCGGAAACAGAGAGAGAA 1891
YS_P._sugitai_YW44_1____            CGAGCAATGAGCAGTGGAGAGAGAACAGGGTAGCGGAAATAGAGAGAGAA 1878
YS_P._urushiyamai_YW50_1____        CGAGCCATGAGCAGTGGAGAGAGAACAGGGTAGCGGAAACAGAGAGAGAA 1891
YS_P._delicatulus_YW52_1____        CGAGCAATGAGCAGTGGAGAGAGAACAGGGTAGCGGAAACAGAGAGAGAA 1892
```

```
YS_P._delicatulus_YW60_2____        CGAGCAATGAGCAGTGGAGAGAGAACAGGGTAGCGGAAACAGAGAGAGAA 1892
YS_P._akitaorum_YW16_1____          CGAGCAATGAGCAGTGGAGAGAGAACAGGGTAGCGGAAACAGAGAGAGAA 1892
YS_P._hongwonpyoi_YW05_8____        CGAGCAATGAGCAGTGGAGAGAGAACAGGGTAGCGGAAACAGAGAGAGAA 1893
YS_P._hongwonpyoi_YW24_1____        CGAGCAATGAGCAGTGGAGAGAGAACAGGGTAGCGGAAACAGAGAGAGAA 1893
YS_P._hongwonpyoi_YW33_8____        CGAGCAATGAGCAGTGGAGAGAGAACAGGGTAGCGGAAACAGAGAGAGAA 1891
YS_P._hongwonpyoi_YW35_8____        CGAGCAATGAGCAGTGGAGAGAGAACAGGGTAGCGGAAACAGAGAGAGAA 1891
YS_P._hongwonpyoi_YW37_8____        CGAGCAATGAGCAGTGGAGAGAGAACAGGGTAGCGGAAACAGAGAGAGAA 1893
YS_P._hongwonpyoi_YW34_8____        CGAGCAATGAGCAGTGGAGAGAGAACAGGGTAGCGGAAACAGAGAGAGAA 1892
YS_P._hongwonpyoi_YW34_2a___        CGAGCAATGAGCAGTGGAGAGAGAACAGGGTAGCGGAAACAGAGAGAGAA 1894
YS_P._viridicuprus_YW15_1____       CGAGCAATGAGCAGTGGAGAGAGAACAGGGTAGCGGAAACAGAGAGAGAA 1892
YS_P._hongwonpyoi_YW36_1____        CGAGCAATGAGCAGTGGAGAGAGAACAGGGTAGCGGAAACAGAGAGAGGG 1893
YS_P._angularis_YW25_8___           CGAGCAATGAGCAGTGGGGAGAAAACAGGGTAGCGGAAAGAGAGAGAGAG 1889
                                    ***** *********** **** **************** ********

YS_P._acuticollis_YW07_8____        TGAG----AGAAATATGAGAAGTTGCGATACAATA-----GAGTGACAGA 1932
YS_P._takakuwai_YW54_1____          TGAG----AGAAATATGAGAAGTTGCGATACAATA-----GAGTGACAGA 1930
YS_P._albisomni_YW19_1____          TGAG----AGAAATATGAGAAGTTGCGATACAATA-----GAGTGACAGA 1932
YS_P._kawadai_YW53_1____            TGAG----AGAAATATGAGAAGTTGCGATACAATA-----GAGTGACAGA 1932
YS_P._kawadai_YW18_1____            TGAG----AGAAATATGAGAAGTTGCGATACAATA-----GAGTGACAGA 1932
YS_P._sue_YW45_1____                TGAG----AGAAATATGAGAAGTTGCGATACAATA-----GAGTGACAGA 1932
YS_P._sue_YW80_3____                TGAG----AGAAATATGAGAAGTTGCGATACAATA-----GAGTGACAGA 1932
YS_P._sugitai_YW44_1____            TGAG----AGAAATATGAGAAGTTGCGATACAATA-----GAGTGACAGA 1919
YS_P._urushiyamai_YW50_1____        TGAG----AGAAATATGAGAAGTTGCGATACAATA-----GAGTGACAGA 1932
YS_P._delicatulus_YW52_1____        TGAG----AGAAATATGAGAAGTTGCGATACAATACAATAGAGTGACAGA 1938
YS_P._delicatulus_YW60_2____        TGAG----AGAAATATGAGAAGTTGCGATACAATA-----GAGTGACAGA 1933
YS_P._akitaorum_YW16_1____          TGAG----AGAAATATGAGAAGTTGCGATACAATA-----GAGTGACAGA 1933
YS_P._hongwonpyoi_YW05_8____        TGAG----AGAAATATGAGAAGCTGCGATACAATA-----GAGTGACAGA 1934
YS_P._hongwonpyoi_YW24_1____        TGAG----AGAAATATGAGAAGCTGCGATACAATA-----GAGTGACAGA 1934
YS_P._hongwonpyoi_YW33_8____        TGAG----AGAAATATGAGAAGCTGCGATACAATA-----GAGTGACAGA 1932
YS_P._hongwonpyoi_YW35_8____        TGAG----AGAAATATGAGAAGCTGCGATACAATA-----GAGTGACAGA 1932
YS_P._hongwonpyoi_YW37_8____        TGAG----AGAAATATGAGAAGCTGCGATACAATA-----GAGTGACAGA 1934
YS_P._hongwonpyoi_YW34_8____        TGAG----AGAAATATGAGAAGCTGCGATACAATA-----GAGTGACAGA 1933
YS_P._hongwonpyoi_YW34_2a___        TGAG----AGAAATATGAGAAGCTGCGATACAATA-----GAGTGACAGA 1935
YS_P._viridicuprus_YW15_1____       TGAG----AGAAATATGAGAAGCTGCGATACAATA-----GAGTGACAGA 1933
YS_P._hongwonpyoi_YW36_1____        TGAG----AGAAATATGAGAAGTTGCGACACAATA-----GAGTGACAGA 1934
YS_P._angularis_YW25_8___           TGAGTGAGAGAAATATGGGAAGTTGCGATAGAATC-----GAGTGACAGA 1934
```

```
                                  ****    ********* **** ***** * ***      **********

YS_P._acuticollis_YW07_8____      AGATCAAGCTTGAGGTGTAGAGGATCAGCA-------------------- 1962
YS_P._takakuwai_YW54_1____        AGATCAAGCTTGAGGTGTAGAGGATCAGCA-------------------- 1960
YS_P._albisomni_YW19_1____        AGATCAAGCTTGAGGTGTAGAGGATCAGCA-------------------- 1962
YS_P._kawadai_YW53_1____          AGATCAAGCTTGAGGTGTAGAGGATCAGCA-------------------- 1962
YS_P._kawadai_YW18_1____          AGATCAAGCTTGAGGTGTAGAGGATCAGCA-------------------- 1962
YS_P._sue_YW45_1____              AGATCAAGCTTGAGGTGTAGAGGATCAGCA-------------------- 1962
YS_P._sue_YW80_3____              AGATCAAGCTTGAGGTGTAGAGGATCAGCA-------------------- 1962
YS_P._sugitai_YW44_1____          AGATCAAGCTTGAGGTGTAGAGGATCAGCA-------------------- 1949
YS_P._urushiyamai_YW50_1____      AGATCAAGCTTGAGGTGTAGAGGATCAGCA-------------------- 1962
YS_P._delicatulus_YW52_1____      AGATCAAGCTTGAGGTGTAGAGGATCAGCA-------------------- 1968
YS_P._delicatulus_YW60_2____      AGATCAAGCTTGAGGTGTAGAGGATCAGCA-------------------- 1963
YS_P._akitaorum_YW16_1____        AGATCAAGCTTGAGGTGTAGAGGATCAGCA-------------------- 1963
YS_P._hongwonpyoi_YW05_8____      AGATCAAGCTTGAGGTGTATAGGATCAGCA-------------------- 1964
YS_P._hongwonpyoi_YW24_1____      AGATCAAGCTTGAGGTGTATAGGATCAGCA-------------------- 1964
YS_P._hongwonpyoi_YW33_8____      AGATCAAGCTTGAGGTGTATAGGATCAGCA-------------------- 1962
YS_P._hongwonpyoi_YW35_8____      AGATCAAGCTTGAGGTGTATAGGATCAGCA-------------------- 1962
YS_P._hongwonpyoi_YW37_8____      AGATCAAGCTTGAGGTGTATAGGATCAGCA-------------------- 1964
YS_P._hongwonpyoi_YW34_8____      AGATCAAGCTTGAGGTGTATAGGATCAGCA-------------------- 1963
YS_P._hongwonpyoi_YW34_2a___      AGATCAAGCTTGAGGTGTATAGGATCAGCA-------------------- 1965
YS_P._viridicuprus_YW15_1____     AGATCAAGCTTGAGGTGTATAGGATCAGCA-------------------- 1963
YS_P._hongwonpyoi_YW36_1____      AGATCAAGCTTGAGGTGTATAGGATCAGCA-------------------- 1964
YS_P._angularis_YW25_8___         AGATCAAGCTTGAGGTGTATAGCAGCAGCATGTTAGCAGCAGCAAGTTAG 1984
                                  ******************* ** * *****

YS_P._acuticollis_YW07_8____      --------AGTTAGCAACAAGAAGTGAAAGGTAGAGATGGGATAGTATTG 2004
YS_P._takakuwai_YW54_1____        --------AGTTAGCAACAAGAAGTGAAAGGTAGAGATGGGATAGTATTG 2002
YS_P._albisomni_YW19_1____        --------AGTTAGCAACAAGAAGTGAAAGGTAGAGATGGGATAGTATTG 2004
YS_P._kawadai_YW53_1____          --------AGTTAGCAACAAGAAGTGAAAGGTAGAGATGGGATAGTATTG 2004
YS_P._kawadai_YW18_1____          --------AGTTAGCAACAAGAAGTGAAAGGTAGAGATGGGATAGTATTG 2004
YS_P._sue_YW45_1____              --------AGTTAGCAACAAGAAGTGAAAGGTAGAGATGGGATAGTATTG 2004
YS_P._sue_YW80_3____              --------AGTTAGCAACAAGAAGTGAAAGGTAGAGATGGGATAGTATTG 2004
YS_P._sugitai_YW44_1____          --------AGTTAGCAACAAGAAGTGAAAGGTAGAGATGGGATAGTATTG 1991
YS_P._urushiyamai_YW50_1____      --------AGTTAGCAACAAGAAGTGAAAGGTAGAGATGGGATAGTATTG 2004
YS_P._delicatulus_YW52_1____      --------AGTTAGCGACAAGAAGTGAAAGGTAGAGATGGGATAGTATTG 2010
```

```
YS_P._delicatulus_YW60_2____        --------AGTTAGCGACAAGAAGTGAAAGGTAGAGATGGGATAGTATTG 2005
YS_P._akitaorum_YW16_1____          --------AGTTAGCAACAAGAAGTGAAAGGTAGAGATGGGATAGTATTG 2005
YS_P._hongwonpyoi_YW05_8____        --------AGTTAGCAGCAAGAAGTGAAAGGTAGAGATGGGATAGTATTG 2006
YS_P._hongwonpyoi_YW24_1____        --------AGTTAGCAGCAAGAAGTGAAAGGTAGAGATGGGATAGTATTG 2006
YS_P._hongwonpyoi_YW33_8____        --------AGTTAGCAGCAAGAAGTGAAAGGTAGAGATGGGATAGTATTG 2004
YS_P._hongwonpyoi_YW35_8____        --------AGTTAGCAGCAAGAAGTGAAAGGTAGAGATGGGATAGTATTG 2004
YS_P._hongwonpyoi_YW37_8____        --------AGTTAGCAGCAAGAAGTGAAAGGTAGAGATGGGATAGTATTG 2006
YS_P._hongwonpyoi_YW34_8____        --------AGTTAGCAGCAAGAAGTGAAAGGTAGAGATGGGATAGTATTG 2005
YS_P._hongwonpyoi_YW34_2a___        --------AGTTAGCAGCAAGAAGTGAAAGGTAGAGATGGGATAGTATTG 2007
YS_P._viridicuprus_YW15_1____       --------AGTTAGCAGCAAGAAGTGAAAGGTAGAGATGGGATAGTATTG 2005
YS_P._hongwonpyoi_YW36_1____        --------AGTTAGCAGCAAGAAGTGAAAGGTAGAGATGGGATAGTATTG 2006
YS_P._angularis_YW25_8___           CAGCAGCAAGTTAGCAGCACGAAGTGAAAGGTAGAGATGGGATAGTATTG 2034
                                            *******  ** ******************************

YS_P._acuticollis_YW07_8____        GGTGGCATAGGAAGAGGTCGTGTGCGGTAGCCAAAATATTTTGGACGGAA 2054
YS_P._takakuwai_YW54_1____          GGTGGCATAGGAAGAGGTCGTGTGCGGTAGCCAAAATATTTTGGACGGAA 2052
YS_P._albisomni_YW19_1____          GGTGGCATAGGAAGAGGTCGTGTGCGGTAGCCAAAATATTTTGGACGGAA 2054
YS_P._kawadai_YW53_1____            GGTGGCATAGGAAGAGGTCGTGTGCGGTAGCCAAAATATTTTGGACGGAA 2054
YS_P._kawadai_YW18_1____            GGTGGCATAGGAAGAGGTCGTGTGCGGTAGCCAAAATATTTTGGACGGAA 2054
YS_P._sue_YW45_1____                GGTGGCATAGGAAGAGGTCGTGTGCGGTAGCCAAAATATTTTGGACGGAA 2054
YS_P._sue_YW80_3____                GGTGGCATAGGAAGAGGTCGTGTGCGGTAGCCAAAATATTTTGGACGGAA 2054
YS_P._sugitai_YW44_1____            GGTGGCATAGGAAGAGGTCGTGTGCGGTAGCCAAAATATTTTGGACGGAA 2041
YS_P._urushiyamai_YW50_1____        GGTGGCATAGGAAGAGGTCGTGTGCGGTAGCCAAAATATTTTGGACGGAA 2054
YS_P._delicatulus_YW52_1____        GGTGGCATAGGAAGAGGTCGTGTGCGGTAGCCAAAATATTTTGGACGGAA 2060
YS_P._delicatulus_YW60_2____        GGTGGCATAGGAAGAGGTCGTGTGCGGTAGCCAAAATATTTTGGACGGAA 2055
YS_P._akitaorum_YW16_1____          GGTGGCATAGGAAGAGGTCGTGTGCGGTAGCCAAAATATTTTGGACGGAA 2055
YS_P._hongwonpyoi_YW05_8____        GGAGGCATAGGAAGAGGTCGTGTGAGGTAGCCAAAATATTTTGGACGGAA 2056
YS_P._hongwonpyoi_YW24_1____        GGAGGCATAGGAAGAGGTCGTGTGAGGTAGCCAAAATATTTTGGACGGAA 2056
YS_P._hongwonpyoi_YW33_8____        GGAGGCATAGGAAGAGGTCGTGTGAGGTAGCCAAAATATTTTGGACGGAA 2054
YS_P._hongwonpyoi_YW35_8____        GGTGGCATAGGAAGAGGTCGTGTGAGGTAGCCAAAATATTTTGGACGGAA 2054
YS_P._hongwonpyoi_YW37_8____        GGTGGCATAGGAAGAGGTCGTGTGAGGTAGCCAAAATATTTTGGACGGAA 2056
YS_P._hongwonpyoi_YW34_8____        GGTGGCATAGGAAGAGGTCGTGTGAGGTAGCCAAAATATTTTGGACGGAA 2055
YS_P._hongwonpyoi_YW34_2a___        GGTGGCATAGGAAGAGGTCGTGTGAGGTAGCCAAAATATTTTGGACGGAA 2057
YS_P._viridicuprus_YW15_1____       GGTGGCATAGGAAGAGGTCGTGTGAGGTAGCCAAAATATTCTGGACGGAA 2055
YS_P._hongwonpyoi_YW36_1____        GGTGGCATAGGAAGAGGTCGTGTGAGGTAGCCAAAATATTTTGGACGGAA 2056
YS_P._angularis_YW25_8___           GGTGGGATAGGAAGAGGTCGTGTGCGGTAGCCAAAATATTTTGGACGGAA 2084
```

```
                                   ** ** ****************** *************** *********

YS_P._acuticollis_YW07_8____       AAAGACCGAACTGGCGAAATGCGACC-AAGTCATTGCATAAAAAAA--GA 2101
YS_P._takakuwai_YW54_1____         AAAGACCGAACTGGCGAAATGCGACC-AAGTCATTGCATAAAAAAA--GA 2099
YS_P._albisomni_YW19_1____         AAAGACCGAACTGGCGAAATGCGACC-AAGTCATTGCATAAAA--A--GA 2099
YS_P._kawadai_YW53_1____           AAAGACCGAACTGGCGAAATGCGACC-AAGTCATTGCATAAAA--A--GA 2099
YS_P._kawadai_YW18_1____           AAAGACCGAACTGGCGAAATGCGACC-AAGTCATTGCATAAAA--A--GA 2099
YS_P._sue_YW45_1____               AAAGACCGAACTGGCGAAATGCGACC-AAGTCATTGCATAAAAA----GA 2099
YS_P._sue_YW80_3____               AAAGACCGAACTGGCGAAATGCGACC-AAGTCATTGCATAAAAA----GA 2099
YS_P._sugitai_YW44_1____           AAAGACCGAACTGGCGAAATGCGACC-AAGTCATTGCATAAAAAAA--GA 2088
YS_P._urushiyamai_YW50_1____       AAAGACCGAACTGGCGAAATGCGACC-AAGTCATTGCATTAAAAAA--GA 2101
YS_P._delicatulus_YW52_1____       AAAGACCGAACTGGCGAAATGCGACCCAAGTCATTGCATAAAAAAA--GA 2108
YS_P._delicatulus_YW60_2____       AAAGACCGAACTGGCGAAATGCGACCCAAGTCATTGCATAAAAAAA--GA 2103
YS_P._akitaorum_YW16_1____         AAAGACCGAACTGGCGAAATGCGACC-AAGTCATTGCATAAAAAAA--GA 2102
YS_P._hongwonpyoi_YW05_8____       AAAGACCGAACTGGCGAAATGCGACC-AAGTCATTGCATAAAAAAA--GA 2103
YS_P._hongwonpyoi_YW24_1____       AAAGACCGAACTGGCGAAATGCGACC-AAGTCATTGCATAAAAAAA--GA 2103
YS_P._hongwonpyoi_YW33_8____       AAAGACCGAACTGGCGAAATGCGACC-AAGTCATTGCATAAAAAAA--GA 2101
YS_P._hongwonpyoi_YW35_8____       AAAGACCGAACTGGCGAAATGCGACC-AAGTCATTGCATAAAAAAAAAGA 2103
YS_P._hongwonpyoi_YW37_8____       AAAGACCGAACTGGCGAAATGCGACC-AAGTCATTGCATAAAAAAAAAGA 2105
YS_P._hongwonpyoi_YW34_8____       AAAGACCGAACTGGCGAAATGCGACC-AAGTCATTGCATAAAAAAAAAGA 2104
YS_P._hongwonpyoi_YW34_2a___       AAAGACCGAACTGGCGAAATGCGACC-AAGTCATTGCATAAAAAAAAAGA 2106
YS_P._viridicuprus_YW15_1____      AAAGACCGAACTGGCGAAATGCGACC-AAGTCATTGCATAAAAAAAA-GA 2103
YS_P._hongwonpyoi_YW36_1____       AAAGACCGAACTGGCGAAATGCGACC-AAGTCATTGCATAAAAAAAAAGA 2105
YS_P._angularis_YW25_8___          AAAGACCGAACTGGCGAAATGCGACC-AAGTCATTGCATAAAAAAAAAGA 2133
                                   ************************** ************ ***     **

YS_P._acuticollis_YW07_8____       CTAAACTTGAATTAATAATGTGATGAGTAACCCGCACAAATCTTTACGTC 2151
YS_P._takakuwai_YW54_1____         CTAAACTTGAATTGATAATGTGATGAGTAACCCGCACAAATCTTTACGTC 2149
YS_P._albisomni_YW19_1____         CTAAACTTGAATTGATAATGTGATGAGTAACCCGCACAAATCTTTACGTC 2149
YS_P._kawadai_YW53_1____           CTAAACTTGAATTGATAATGTGATGAGTAACCCGCACAAATCTTTACGTC 2149
YS_P._kawadai_YW18_1____           CTAAACTTGAATTGATAATGTGATGAGTAACCCGCACAAATCTTTACGTC 2149
YS_P._sue_YW45_1____               CTAAACTTGAATTGATAATGTGATGAGTAACCCGCACAAATCTTTACGTC 2149
YS_P._sue_YW80_3____               CTAAACTTGAATTGATAATGTGATGAGTAACCCGCACAAATCTTTACGTC 2149
YS_P._sugitai_YW44_1____           CTAAACTTGAATTGATAATGTGATGAGTAACCCGCACAAATCTTTACGTC 2138
YS_P._urushiyamai_YW50_1____       CTAAACTTGAATTGATAATGTGATGAGTAACCCGCACAAATCTTTACGTC 2151
YS_P._delicatulus_YW52_1____       CTAAACTTGAATTGATAATGTGATGAGTAACCCGCACAAATCTTTACGTC 2158
```

```
YS_P._delicatulus_YW60_2____        CTAAACTTGAATTGATAATGAGATGAGTAACCCGCGCAAATCTTTACGTC 2153
YS_P._akitaorum_YW16_1____          CTAAACTTGAATTGATAATGTGATGAGTAACCCGCACAAATCTTTACGTC 2152
YS_P._hongwonpyoi_YW05_8____        CTAAACTTGAATTGATAATGTGATGAGTAACCCGCACAAATCTTTACGTC 2153
YS_P._hongwonpyoi_YW24_1____        CTAAACTTGAATTGATAATGTGATGAGTAACCCGCACAAATCTTTACGTC 2153
YS_P._hongwonpyoi_YW33_8____        CTAAACTTGAATTGATAATGTGATGAGTAACCCGCACAAATCTTTACGTC 2151
YS_P._hongwonpyoi_YW35_8____        CTAAACTTGAATTGATAATGTGATGAGTAACCCGCACAAATCTTTACGTC 2153
YS_P._hongwonpyoi_YW37_8____        CTAAACTTGAATTGATAATGTGATGAGTAACCCGCACAAATCTTTACGTC 2155
YS_P._hongwonpyoi_YW34_8____        CTAAACTTGAATTGATAATGTGATGAGTAACCCGCACAAATCTTTACGTC 2154
YS_P._hongwonpyoi_YW34_2a___        CTAAACTTGAATTGATAATGTGATGAGTAACCCGCACAAATCTTTACGTC 2156
YS_P._viridicuprus_YW15_1____       CTAAACTTGAATTGATAATGTGATGAGTAACCCGCACAAATCTTTACGTC 2153
YS_P._hongwonpyoi_YW36_1____        CTAAACTTGAATTGATAATGTGATGAGTAACCCGCACAAATCTTTACGTC 2155
YS_P._angularis_YW25_8___           CTAAACTTCAATTGATAATGTGATGAGTAACCCGCACAAATCTTTACGTC 2183
                                    ******** **** ****** ************** **************

YS_P._acuticollis_YW07_8____        TCAAAAAACTTTTTCCCTTGAGAAAGTTGAGAAGAAATATTTTTAGTTTG 2201
YS_P._takakuwai_YW54_1____          TCAAAAAACTTTTTCCCTTGAGAAAGTTGAGAAGAAATATTTTTAGTTTG 2199
YS_P._albisomni_YW19_1____          TCAAAAAACTTTTTCCCTTGAGAAAGTTGAGAAGAAATATTTTTAGTTTG 2199
YS_P._kawadai_YW53_1____            TCAAAAAACTTTTTCCCTTGAGAAAGTTGAGAAGAAATATTTTTAGTTTG 2199
YS_P._kawadai_YW18_1____            TCAAAAAACTTTTTCCCTTGAGAAAGTTGAGAAGAAATATTTTTAGTTTG 2199
YS_P._sue_YW45_1____                TCAAAAAACTTTTTCCCTTGAGAAAGTTGAAAAGAAATATTTTTAGTTTG 2199
YS_P._sue_YW80_3____                TCAAAAAACTTTTTCCCTTGAGAAAGTTGAAAAGAAATATTTTTAGTTTG 2199
YS_P._sugitai_YW44_1____            TCAAAAAACTTTTTCCCTTGAGAAAGTTGAAAAGAAATATTTTTAGTTTG 2188
YS_P._urushiyamai_YW50_1____        TCAAAAAACTTTTTCCCTTGAGAAAGTTGAAAAGAAATATTTTTAGTTTG 2201
YS_P._delicatulus_YW52_1____        TCAAAAAACTTTTTCCCTTGAGAAAGTTGAAAAGAAATATTTTTAGTTTG 2208
YS_P._delicatulus_YW60_2____        TCAAAAAACTTTTTCCCTTGAGAAAGTTGAAAAGAAATATTTTTAGTTTG 2203
YS_P._akitaorum_YW16_1____          TCAAAAAACTTTTTCCCTTGCGAAAGTTGAGAAGAAATAATTTTAGTTTG 2202
YS_P._hongwonpyoi_YW05_8____        TCAAAAAACTTTTTCCCTTGAGAAAGTTGAGAAGAAATATTTTTAGTTTG 2203
YS_P._hongwonpyoi_YW24_1____        TCAAAAAACTTTTTCCCTTGAGAAAGTTGAGAAGAAATATTTTTAGTTTG 2203
YS_P._hongwonpyoi_YW33_8____        TCAAAAAACTTTTTCCCTTGAGAAAGTTGAGAAGAAATATTTTTAGTTTG 2201
YS_P._hongwonpyoi_YW35_8____        TCAAAAAACTTTTTCCCTTGAGAAAGTTGAGAAGAAATATTTTTAGTTTG 2203
YS_P._hongwonpyoi_YW37_8____        TCAAAAAACTTTTTCCCTTGAGAAAGTTGAGAAGAAATATTTTTAGTTTG 2205
YS_P._hongwonpyoi_YW34_8____        TCAAAAAACTTTTTCCCTTGAGAAAGTTGAGAAGAAATATTTTTAGTTTG 2204
YS_P._hongwonpyoi_YW34_2a___        TCAAAAAACTTTTTCCCTTGAGAAAGTTGAGAAGAAATATTTTTAGTTTG 2206
YS_P._viridicuprus_YW15_1____       TCAAAAAACTTTTTCCCTTGAGAAAGTTGAGAAGAAATATTTTTAGTTTG 2203
YS_P._hongwonpyoi_YW36_1____        TCAAAAAACTTTTTCCCTTGAGAAAGTTGAGAAGAAATATTTTTAGTTTG 2205
YS_P._angularis_YW25_8___           TCAAAAAACTTTTTCCCTTGAGAAAGTTGAGAAGAAATATTTTTAGTTTG 2233
```

```
                                  ******************** ********* ******** **********

YS_P._acuticollis_YW07_8____      TGCAAGGGAAAAGAGGTGGTGGATCAGAAAACATCAGAGATATGCTGGGA 2251
YS_P._takakuwai_YW54_1____        TGCAAGGGAAAAGAGGTGGTGGATCAGAAAACATCAGAGATATGCTGGGA 2249
YS_P._albisomni_YW19_1____        TGCAAGGGAAAAGAGGTGGTGGATCAGAAAACATCAGAGATATGCTGGGA 2249
YS_P._kawadai_YW53_1____          TGCAAGGGAAAAGAGGTGGTGGATCAGAAAACATCAGAGATATGCTGGGA 2249
YS_P._kawadai_YW18_1____          TGCAAGGGAAAAGAGGTGGTGGATCAGAAAACATCAGAGATATGCTGGGA 2249
YS_P._sue_YW45_1____              CGCAAGGGAAAAGAGGTGGTGGATCAGAAAACATCAGAGATATGCTGGGA 2249
YS_P._sue_YW80_3____              CGCAAGGGAAAAGAGGTGGTGGATCAGAAAACATCAGAGATATGCTGGGA 2249
YS_P._sugitai_YW44_1____          CGCAAGGGAAAAGAGGTGGTGGATCAGAAAACATCAGAGATATGCTGGGA 2238
YS_P._urushiyamai_YW50_1____      CGCAAGGGAAAAGAGGTGGTGGATCAGAAAACATCAGAGATACGCTGGGA 2251
YS_P._delicatulus_YW52_1____      TGCAAGGGAAAAGAGGTGGTGGATCAGAAAACATCAGAGATATGCTGGGA 2258
YS_P._delicatulus_YW60_2____      TGCAAGGGAAAAGAGGTGGTGGATCAGAAAACATCAGAGATATGCTGGGA 2253
YS_P._akitaorum_YW16_1____        TGCAAGGGAAAAGAGGTGGTTGATCAGAAAACATCAGAGATATGCTGGGA 2252
YS_P._hongwonpyoi_YW05_8____      TGCAAGGGAATAGAGGTGGTGGATCAGAAAACATCAGAGATATGCTGGGA 2253
YS_P._hongwonpyoi_YW24_1____      TGCAAGGGAATAGAGGTGGTGGATCAGAAAACATCAGAGATATGCTGGGA 2253
YS_P._hongwonpyoi_YW33_8____      TGCAAGGGAATAGAGGTGGTGGATCAGAAAACATCAGAGATATGCTGGGA 2251
YS_P._hongwonpyoi_YW35_8____      TGCAAGGGAATAGAGGTGGTGGATCAGAAAACATCAGAGATATGCTGGGA 2253
YS_P._hongwonpyoi_YW37_8____      TGCAAGGGAATAGAGGTGGTGGATCAGAAAACATCAGAGATATGCTGGGA 2255
YS_P._hongwonpyoi_YW34_8____      TGCAAGGGAATAGAGGTGGTGGATCAGAAAACATCAGAGATATGCTGGGA 2254
YS_P._hongwonpyoi_YW34_2a___      TGCAAGGGAATAGAGGTGGTGGATCAGAAAACATCAGAGATATGCTGGGA 2256
YS_P._viridicuprus_YW15_1____     TGCAAGGGAATAGAGGTGGTGGATCAGAAAACATCAGAGATATGCTGGGA 2253
YS_P._hongwonpyoi_YW36_1____      TGCAAGGGAATAGAGGTGGTGGATCAGAAAACATCAGAGATATGCTGGGA 2255
YS_P._angularis_YW25_8___         CGCAAAGGAATAGAGGTGGTGGATCAGAAAACATCAGAGATATGCTGGGA 2283
                                   **** **** ********* ********************* *******

YS_P._acuticollis_YW07_8____      GGAATTACATGCGAAAGCATGAAACAGTTGTAAAACAATTCAGATAATGA 2301
YS_P._takakuwai_YW54_1____        GGAATTACATGCGAAAGCATGAAACAGTTGTAAAACAATTCAGATAATGA 2299
YS_P._albisomni_YW19_1____        GGAATTACATGCGAAAGCATGAAACAGTTGTAAAACAATTCAGATAATGA 2299
YS_P._kawadai_YW53_1____          GGAATTACATGCGAAAGCATGAAACAGTTGTAAAACAATTCAGATAATGA 2299
YS_P._kawadai_YW18_1____          GGAATTACATGCGAAAGCATGAAACAGTTGTAAAACAATTCAGATAATGA 2299
YS_P._sue_YW45_1____              GGAATTACATGCGAAAGCATGAAACAGTTGTAAAACAATTCAGATAATGA 2299
YS_P._sue_YW80_3____              GGAATTACATGCGAAAGCATGAAACAGTTGTAAAACAATTCAGATAATGA 2299
YS_P._sugitai_YW44_1____          GGAATTACATGCGAAAGCATGAAACAGTTGTAAAACAATTCAGATAATGA 2288
YS_P._urushiyamai_YW50_1____      GGAATTACATGCGAAAGCATGAAACAGTTGTAAAACAATTCAGATAATGA 2301
YS_P._delicatulus_YW52_1____      GGAATTACATGCGAAAGCATGAAACAGTTGTAAAACAATTCAGATAATGA 2308
```

```
YS_P._delicatulus_YW60_2____        GGAATTACATGCGAAAGCATGAAACAGTTGTAAAACAATTCAGATAATGA 2303
YS_P._akitaorum_YW16_1____          GGAATTACATGCGAAAGCATGAAACAGTTGTAAAACAATTCAGATAATGA 2302
YS_P._hongwonpyoi_YW05_8____        GGAATTACATGCGAAAGCATGAAACAGTTGTAAAACAATTCAGATAATGA 2303
YS_P._hongwonpyoi_YW24_1____        GGAATTACATGCGAAAGCATGAAACAGTTGTAAAACAATTCAGATAATGA 2303
YS_P._hongwonpyoi_YW33_8____        GGAATTACATGCGAAAGCATGAAACAGTTGTAAAACAATTCAGATAATGA 2301
YS_P._hongwonpyoi_YW35_8____        GGAATTACATGCGAAAGCATGAAACAGTTGTAAAACAATTCAGATAATGA 2303
YS_P._hongwonpyoi_YW37_8____        GGAATTACATGCGAAAGCATGAAACAGTTGTAAAACAATTCAGATAATGA 2305
YS_P._hongwonpyoi_YW34_8____        GGAATTACATGCGAAAGCATGAAACAGTTGTAAAACAATTCAGATAATGA 2304
YS_P._hongwonpyoi_YW34_2a___        GGAATTACATGCGAAAGCATGAAACAGTTGTAAAACAATTCAGATAATGA 2306
YS_P._viridicuprus_YW15_1____       GGAATTACATGCGAAAGCATGAAACAGTTGTAAAACAATTCAGATAATGA 2303
YS_P._hongwonpyoi_YW36_1____        GGAATTACATGCGAAAGCATGAAACAGTTGTAAAACAATTCAGATAATGA 2305
YS_P._angularis_YW25_8___           GGAATTACATGCGAAAGCATGAAACAGTTGTAAAACAATTCAGATAATGA 2333
                                    **************************************************

YS_P._acuticollis_YW07_8____        GTTGTAGTAACATTGTTTATATGGCCTCGTTGTTAAGTGGAGAGAGTTTA 2351
YS_P._takakuwai_YW54_1____          GTTGTAGTAACATTGTTTATATGGCCTCGTTGTTAAGTGGAGAGAGTTTA 2349
YS_P._albisomni_YW19_1____          GTTGTAGTAACATTGTTTATATGGCCTCGTTGTTAAGTGGAGAGAGTTTA 2349
YS_P._kawadai_YW53_1____            GTTGTAGTAACATTGTTTATATGGCCTCGTTGTTAAGTGGAGAGAGTTTA 2349
YS_P._kawadai_YW18_1____            GTTGTAGTAACATTGTTTATATGGCCTCGTTGTTAAGTGGAGAGAGTTTA 2349
YS_P._sue_YW45_1____                GTTGTAGTAACATTGTTTATATGGCCTCGTTGTTAAGTGGAGAGAGTTTA 2349
YS_P._sue_YW80_3____                GTTGTAGTAACATTGTTTATATGGCCTCGTTGTTAAGTGGAGAGAGTTTA 2349
YS_P._sugitai_YW44_1____            GTTGTAGTAACATTGTTTATATGGCCTCGTTGTTAAGTGGAGAGAGTTTA 2338
YS_P._urushiyamai_YW50_1____        GTTGTAGTAACATTGTTTATATGGCCTCGTTGTTAAGTGGAGAGAGTTTA 2351
YS_P._delicatulus_YW52_1____        GTTGTAGTAACATTGTTTATATGGCCTTGTTGTTAAGTGGAGAGAGTTTA 2358
YS_P._delicatulus_YW60_2____        GTTGTAGTAACATTGTTTATATGGCCTCGTTGTTAAGTGGAGAGAGTTTA 2353
YS_P._akitaorum_YW16_1____          GTTGTAGTAACATTGTTTATATGGCCTCGTTGTTAAGTGGAGAGAGTTTA 2352
YS_P._hongwonpyoi_YW05_8____        GTTGTAGTAACATTGTTTTTATGGCCTCGTTGTTAAGTGGAGAGAGTTTA 2353
YS_P._hongwonpyoi_YW24_1____        GTTGTAGTAACATTGTTTTTATGGCCTCGTTGTTAAGTGGAGAGAGTTTA 2353
YS_P._hongwonpyoi_YW33_8____        GTTGTAGTAACATTGTTTTTATGGCCTCGTTGTTAAGTGGAGAGAGTTTA 2351
YS_P._hongwonpyoi_YW35_8____        GTTGTAGTAACATTGTTTTTATGGCCTCGTTGTTAAGTGGAGAGAGTTTA 2353
YS_P._hongwonpyoi_YW37_8____        GTTGTAGTAACATTGTTTTTATGGCCTCGTTGTTAAGTGGAGAGAGTTTA 2355
YS_P._hongwonpyoi_YW34_8____        GTTGTAGTAACATTGTTTTTATGGCCTCGTTGTTAAGTGGAGAGAGTTTA 2354
YS_P._hongwonpyoi_YW34_2a___        GTTGTAGTAACATTGTTTTTATGGCCTCGTTGTTAAGTGGAGAGAGTTTA 2356
YS_P._viridicuprus_YW15_1____       GTTGTAGTAACATTGTTTTTATGGCCTCGTTGTTAAGTGGAGAGAGTTTA 2353
YS_P._hongwonpyoi_YW36_1____        GTTGTAGTAACATTGTTTTTATGGCCTCGTTGTTAAGTGGAGAGAGTTTA 2355
YS_P._angularis_YW25_8___           GTTGTAGTAACATTGTTTTTATGGCCTCGTTGTTAAGTGGAGAGAGTTTA 2383
```

```
                                    ****************** ******** **********************

YS_P._acuticollis_YW07_8____        ATGGTGAACGTAAGTTTGGCATTGCTTTTTACGCTATGACAACATGGG-T 2400
YS_P._takakuwai_YW54_1____          ATGGTGAACGTAAGTTTGGCATTGCTTTTTACGCTATGACAACATGGG-T 2398
YS_P._albisomni_YW19_1____          ATGGTGAACGTAAGTTTGGCATTGCTTTTTACGCTATGACAACATGGGGT 2399
YS_P._kawadai_YW53_1____            ATGGTGAACGTAAGTTTGGCATTGCTTTTTACGCTATGACAACATGGG-T 2398
YS_P._kawadai_YW18_1____            ATGGTGAACGTAAGTTTGGCATTGCTTTTTACGCTATGACAACATGGG-T 2398
YS_P._sue_YW45_1____                ATGGTGAACGTAAGTTTGGCATTGCTTTTTACGCTATGACAACATGGG-T 2398
YS_P._sue_YW80_3____                ATGGTGAACGTAAGTTTGGCATTGCTTTTTACGCTATGACAACATGGG-T 2398
YS_P._sugitai_YW44_1____            ATGGTGAACGTAAGTTTGGCATTGCTTTTTACGCTATGACAACATGGG-T 2387
YS_P._urushiyamai_YW50_1____        ATGGTGAACGTAAGTTTGGCATTGCTTTTTACGCTATGACAACATGGG-T 2400
YS_P._delicatulus_YW52_1____        ATGGTGAACGTAAGTTTGGCATTGCTTTTTACGCTATGACAACATGGG-T 2407
YS_P._delicatulus_YW60_2____        ATGGTGAACGTAAGTTTGGCATTGCTTTTTACGCTATGACAACATGGG-T 2402
YS_P._akitaorum_YW16_1____          ATGGTGAACGTAAGTTTGGCATTGCTTTTTACGCTATGACAACATGGG-T 2401
YS_P._hongwonpyoi_YW05_8____        ATGGTGAACGTAAGTTTGGCATTGCTTTTTACGCTATGACAACATGGG-T 2402
YS_P._hongwonpyoi_YW24_1____        ATGGTGAACGTAAGTTTGGCATTGCTTTTTACGCTATGACAACATGGG-T 2402
YS_P._hongwonpyoi_YW33_8____        ATGGTGAACGTAAGTTTGGCATTGCTTTTTACGCTATGACAACATGGG-T 2400
YS_P._hongwonpyoi_YW35_8____        ATGGTGAACGTAAGTTTGGCATTGCTTTTTACGCTATGACAACATGGG-T 2402
YS_P._hongwonpyoi_YW37_8____        ATGGTGAACGTAAGTTTGGCATTGCTTTTTACGCTATGACAACATGGG-T 2404
YS_P._hongwonpyoi_YW34_8____        ATGGTGAACGTAAGTTTGGCATTGCTTTTTACGCTATGACAACATGGG-T 2403
YS_P._hongwonpyoi_YW34_2a___        ATGGTGAACGTAAGTTTGGCATTGCTTTTTACGCTATGACAACATGGG-T 2405
YS_P._viridicuprus_YW15_1____       ATGGTGAACGTAAGTTTGGCATTGCTTTTTACGCTATGACAACATGGG-T 2402
YS_P._hongwonpyoi_YW36_1____        ATGGTGAACGTAAGTTTGGCATTGCTTTTTACGCTATGACAACATGGG-T 2404
YS_P._angularis_YW25_8___           ATGGTGAACGTAAGTTTGGCATTGCTCTTTACGCTATGACAACATGGG-T 2432
                                    ************************** ********************* *

YS_P._acuticollis_YW07_8____        GATCTTGCCTTGTGCAAGAAAACCTGAACAGTCTCATACTAGATATCTAA 2450
YS_P._takakuwai_YW54_1____          GATCTTGCCTTGTGCAAGAAAACCTGAACAGTCTCATACTAGATATCTAA 2448
YS_P._albisomni_YW19_1____          GATCTTGCCTTGTGCAAGAAAACCTGAACAGTCTCATACTAGATATCTAA 2449
YS_P._kawadai_YW53_1____            GATCTTGCCTTGTGCAAGAAAACCTGAACAGTCTCATACTAGATATCTAA 2448
YS_P._kawadai_YW18_1____            GATCTTGCCTTGTGCAAGAAAACCTGAACAGTCTCATACTAGATATCTAA 2448
YS_P._sue_YW45_1____                GATCTTGCCTTGTGCAAGAAAACCTGAACAGTCTCATACTAGATATCTAA 2448
YS_P._sue_YW80_3____                GATCTTGCCTTGTGCAAGAAAACCTGAACAGTCTCATACTAGATATCTAA 2448
YS_P._sugitai_YW44_1____            GATCTTGCCTTGTGCAAGAAAACCTGAACAGTCTCATACTAGATATCTAA 2437
YS_P._urushiyamai_YW50_1____        GATCTTGCCTTGTGCAAGAAAACCTGAACAGTCTCATACTAGATATCTAA 2450
YS_P._delicatulus_YW52_1____        GATCTTGCCTTGTGCAAGAAAACCTGAACAGTCTCATACTAGATATCTAA 2457
```

```
YS_P._delicatulus_YW60_2____       GATCTTGCCTTGTGCAAGAAAACCTGAACAGTCTCATACTAGATATCTAA 2452
YS_P._akitaorum_YW16_1____         GATCTTGCCTTGTGCAAGAAAACCTGAACAGTCTCATACTAGATATCTAA 2451
YS_P._hongwonpyoi_YW05_8____       GATCTTGCCTTGTGCAAGAAAACCTGAACAGTCTCATACTAGATATCTAA 2452
YS_P._hongwonpyoi_YW24_1____       GATCTTGCCTTGTGCAAGAAAACCTGAACAGTCTCATACTAGATATCTAA 2452
YS_P._hongwonpyoi_YW33_8____       GATCTTGCCTTGTGCAAGAAAACCTGAACAGTCTCATACTAGATATCTAA 2450
YS_P._hongwonpyoi_YW35_8____       GATCTTGCCTTGTGCAAGAAAACCTGAACAGTCTCATACTAGATATCTAA 2452
YS_P._hongwonpyoi_YW37_8____       GATCTTGCCTTGTGCAAGAAAACCTGAACAGTCTCATACTAGATATCTAA 2454
YS_P._hongwonpyoi_YW34_8____       GATCTTGCCTTGTGCAAGAAAACCTGAACAGTCTCATACTAGATATCTAA 2453
YS_P._hongwonpyoi_YW34_2a___       GATCTTGCCTTGTGCAAGAAAACCTGAACAGTCTCATACTAGATATCTAA 2455
YS_P._viridicuprus_YW15_1____      GATCTTGCCTTGTGCAAGAAAACCTGAACAGTCTCATACTAGATATCTAA 2452
YS_P._hongwonpyoi_YW36_1____       GATCTTGCCTTGTGCAAGAAAACCTGAACAGTCTCATACTAGATATCTAA 2454
YS_P._angularis_YW25_8___          GATCTTGCCTTGTGCAAGAAAACCTGAACAGTCTCATACTAGATATCTAA 2482
                                   **************************************************

YS_P._acuticollis_YW07_8____       CGATTCTATGTATGAGCGCTGAAGGATTTAGTGGATTACTAGCTGATAGC 2500
YS_P._takakuwai_YW54_1____         CGATTCTATGTATGAGCGCTGAAGGATTTAGTGGATTACTAGCTGATAGC 2498
YS_P._albisomni_YW19_1____         CGATTCTATGTATGAGCGCTGAAGGATTTAGTGGATTACTAGCTGATAGC 2499
YS_P._kawadai_YW53_1____           CGATTCTATGTATGAGCGCTGCAGGATTTAGTGGATTACTAGCTAATAGC 2498
YS_P._kawadai_YW18_1____           CGATTCTATGTATGAGCGCTGAAGGATTTAGTGGATTACTAGCTGATAGC 2498
YS_P._sue_YW45_1____               CGATTCTATGTATGAGCGCTGAAGGATTTGGTGGATTACTAGCTGATAGC 2498
YS_P._sue_YW80_3____               CGATTCTATGTATGAGCGCTGAAGGATTTGGTGGATTACTAGCTGATAGC 2498
YS_P._sugitai_YW44_1____           CGATTCTATGTATGAGCGCTGAAGGATTTGGTGGATTACTAGCTGATAGC 2487
YS_P._urushiyamai_YW50_1____       CGATTCTATGTATGAGCGCTGAAGGATTTGGTGGATTACTAGCTGATAGC 2500
YS_P._delicatulus_YW52_1____       CGATTCTATGTATGAGCGCTGAAGGATTTAGTGGATTACTAGCTGATAGC 2507
YS_P._delicatulus_YW60_2____       CGATTCTATGTATGAGCGCTGAAGGATTTGGTGGATTACTAGCTGATAGC 2502
YS_P._akitaorum_YW16_1____         CGATTCTATGTATGAGCGCTGAAGGATTTAGTGGATTACTAGCTGATAGC 2501
YS_P._hongwonpyoi_YW05_8____       CGATTCTATGTATGAGCGCTGAAGGATTTAGTGGATTACTAGCTGATAGC 2502
YS_P._hongwonpyoi_YW24_1____       CGATTCTATGTATGAGCGCTGAAGGATTTAGTGGATTACTAGCTGATAGC 2502
YS_P._hongwonpyoi_YW33_8____       CGATTCTATGTATGAGCGCTGAAGGATTTAGTGGATTACTAGCTGATAGC 2500
YS_P._hongwonpyoi_YW35_8____       CGATTCTATGTATGAGCGCTGAAGGATTTAGTGGATTACTAGCTGATAGC 2502
YS_P._hongwonpyoi_YW37_8____       CGATTCTATGTATGAGCGCTGAAGGATTTAGTGGATTACTAGCTGATAGC 2504
YS_P._hongwonpyoi_YW34_8____       CGATTCTATGTATGAGCGCTGAAGGATTTAGTGGATTACTAGCTGATAGC 2503
YS_P._hongwonpyoi_YW34_2a___       CGATTCTATGTATGAGCGCTGAAGGATTTAGTGGATTACTAGCTGATAGC 2505
YS_P._viridicuprus_YW15_1____      CGATTCTATGTATGAGCGCTGAAGGATTTAGTGGATTACTAGCTGATAGC 2502
YS_P._hongwonpyoi_YW36_1____       CGATTCTATGTATGAGCGCTGAAGGATTTAGTGGATTACTAGCTGATAGC 2504
YS_P._angularis_YW25_8___          CGATTCTATGTATGAGCGCTGAAGGATTTAGTGGATTACTAGCTGATAGC 2532
```

```
                                  ********************* ******* ************** *****

YS_P._acuticollis_YW07_8____      AAACATTCATTAAAACTTATTTGGGGGTCTGCCTTGACAAATAGCCGGAT 2550
YS_P._takakuwai_YW54_1____        AAACATTCATTAAAACTTATTTGGGGGTCTGCCTTGACAAATAGCCGGAT 2548
YS_P._albisomni_YW19_1____        AAACATTCATTAAAACTTATTTGGGGGTCTGCCTTGACAAATAGCCGGAT 2549
YS_P._kawadai_YW53_1____          AAACATTCATTAAAACTTATTTGGGGGTCTGCCTTGACAAATAGCCGGAT 2548
YS_P._kawadai_YW18_1____          AAACATTCATTAAAACTTATTTGGGGGTCTGCCTTGACAAATAGCCGGAT 2548
YS_P._sue_YW45_1____              AAACATTCATTAAAACTTATTTGGGGGTCTGCCTTGACAAATAGCCGGAT 2548
YS_P._sue_YW80_3____              AAACATTCATTAAAACTTATTTGGGGGTCTGCCTTGACAAATAGCCGGAT 2548
YS_P._sugitai_YW44_1____          AAACATTCATTAAAACTTATTTGGGGGTCTGCCTTGACAAATAGCCGGAT 2537
YS_P._urushiyamai_YW50_1____      AAACATTCATTAAAACTTATTTGGGGGTCTGCCTTGACAAATAGCCGGAT 2550
YS_P._delicatulus_YW52_1____      AAACATTCATTAAAACTTATTTGGGGGTCTGCCTTGACAAATAGCCGGAT 2557
YS_P._delicatulus_YW60_2____      AAACATTCATTAAAACTTATTTGGGGGTCTGCCTTGACAAATAGCCGGAT 2552
YS_P._akitaorum_YW16_1____        AAACATTCATTAAAACTTTTTTGGGGGTCTGCCTTGACAAATAGCCGGAT 2551
YS_P._hongwonpyoi_YW05_8____      AAACATTCATTAAAACTTATTTGGGGGTCTGCCTTGACAAATAGCCGGAT 2552
YS_P._hongwonpyoi_YW24_1____      AAACATTCATTAAAACTTATTTGGGGGTCTGCCTTGACAAATAGCCGGAT 2552
YS_P._hongwonpyoi_YW33_8____      AAACATTCATTAAAACTTATTTGGGGGTCTGCCTTGACAAATAGCCGGAT 2550
YS_P._hongwonpyoi_YW35_8____      AAACATTCATTAAAACTTATTTGGGGGTCTGCCTTGACAAATAGCCGGAT 2552
YS_P._hongwonpyoi_YW37_8____      AAACATTCATTAAAACTTATTTGGGGGTCTGCCTTGACAAATAGCCGGAT 2554
YS_P._hongwonpyoi_YW34_8____      AAACATTCATTAAAACTTATTTGGGGGTCTGCCTTGACAAATAGCCGGAT 2553
YS_P._hongwonpyoi_YW34_2a___      AAACATTCATTAAAACTTATTTGGGGGTCTGCCTTGACAAATAGCCGGAT 2555
YS_P._viridicuprus_YW15_1____     AAACATTCATTAAAACTTATTTGGGGGTCTGCCTTGACAAATAGCCGGAT 2552
YS_P._hongwonpyoi_YW36_1____      AAACATTCATTAAAACTTATTTGGGGGTCTGCCTTGACAAATAGCCGGAT 2554
YS_P._angularis_YW25_8___         AAACATTCATTAAAACTTATTTGGGGGTCTGCCTTGACAAATAGCCGGAT 2582
                                  ****************** *******************************

YS_P._acuticollis_YW07_8____      TAAGTTCTGTGATTTTGCCAAGTGTAACCTCCTCCATGGTTGGTTATGTA 2600
YS_P._takakuwai_YW54_1____        TAAGTTCTGTGATTTTGCCAAGTGTAACCTCCTCCATGGTTGGTTATGTA 2598
YS_P._albisomni_YW19_1____        TAAGTTCTGTGATTTTGCCAAGTGTAACCTCCTCCATGGTTGGTTATGTA 2599
YS_P._kawadai_YW53_1____          TAAGTTCTGTGATTTTGCCAAGTGTAACCTCCTCCATGGTTGGTTATGTA 2598
YS_P._kawadai_YW18_1____          TAAGTTCTGTGATTTTGTCAAGTGTAACCTCCTCCATGGTTGGTTATGTA 2598
YS_P._sue_YW45_1____              TAAGTTCTGTGATTTTGCCAAGTGTAACCTCCTCCATGGTTGGTTATGTA 2598
YS_P._sue_YW80_3____              TAAGTTCTGTGATTTTGCCAAGTGTAACCTCCTCCATGGTTGGTTATGTA 2598
YS_P._sugitai_YW44_1____          TAAGTTCTGTGATTTTGCCAAGTGTAACCTCCTCCATGGTTGGTTATGTA 2587
YS_P._urushiyamai_YW50_1____      TAAGTTCTGTGATTTTGCCAAGTGTAACCTCCTCCATGGTTGGTTATGTA 2600
YS_P._delicatulus_YW52_1____      TAAGTTCTGTGATTTTGCCAAGTGTAACCTCCTCCATGGTTGGTTATGTA 2607
```

```
YS_P._delicatulus_YW60_2____        TAAGTTCTGTGATTTTGCCAAGTGTAACCTCCTCCATGGTTGGTTATGTA 2602
YS_P._akitaorum_YW16_1____          TAAGTTCTGTGATTTTGCCAAGTGTAACCTCCTCCATGGTTGGTTATGTA 2601
YS_P._hongwonpyoi_YW05_8____        TAAGTTCTGTGATTTTGCCAAGTGTAACCTCCTCCATGGTTGGTTATGTA 2602
YS_P._hongwonpyoi_YW24_1____        TAAGTTCTGTGATTTTGCCAAGTGTAACCTCCTCCATGGTTGGTTATGTA 2602
YS_P._hongwonpyoi_YW33_8____        TAAGTTCTGTGATTTTGCCAAGTGTAACCTCCTCCATGGTTGGTTATGTA 2600
YS_P._hongwonpyoi_YW35_8____        TAAGTTCTGTGATTTTGCCAAGTGTAACCTCCTCCATGGTTGGTTATGTA 2602
YS_P._hongwonpyoi_YW37_8____        TAAGTTCTGTGATTTTGCCAAGTGTAACCTCCTCCATGGTTGGTTATGTA 2604
YS_P._hongwonpyoi_YW34_8____        TAAGTTCTGTGATTTTGCCAAGTGTAACCTCCTCCATGGTTGGTTATGTA 2603
YS_P._hongwonpyoi_YW34_2a___        TAAGTTCTGTGATTTTGCCAAGTGTAACCTCCTCCATGGTTGGTTATGTA 2605
YS_P._viridicuprus_YW15_1____       TAAGTTCTGTGATTTTGCCAAGTGTAACCTCCTCCATGGTTGGTTATGTA 2602
YS_P._hongwonpyoi_YW36_1____        TAAGTTCTGTGATTTTGCCAAGTGTAACCTCCTCCATGGTTGGTTATGTA 2604
YS_P._angularis_YW25_8___           TAAGTTCTGTGATTTTGCCAAGTGTAACCTCCTCCATGGTTGGTTATGTA 2632
                                    ***************** ********************************

YS_P._acuticollis_YW07_8____        AAACCTTGTGTTGTAAAAAACTCGAGTGTTTTGCGGACATCATACCAGGG 2650
YS_P._takakuwai_YW54_1____          AAACCTTGTGTTGTAAAAAACTCGAGTGTTTTGCGGACATCATACCAGGG 2648
YS_P._albisomni_YW19_1____          AAACCTTGTGTTGTAAAAAACTCGAGTGTTTTGCGGACATCATACCAGGG 2649
YS_P._kawadai_YW53_1____            AAACCTTGTGTTGTAAAAAACTCGAGTGTTTTGCGGACATCATACCAGGG 2648
YS_P._kawadai_YW18_1____            AAACCTTGTGTTGTAAAAAACTCGAGTGTTTTGCGGACATCATACCAGGG 2648
YS_P._sue_YW45_1____                AAACCTTGTGTTGTAAAAAACTCGAGTGTTTTGCGGACATCATACCAGGG 2648
YS_P._sue_YW80_3____                AAACCTTGTGTTGTAAAAAACTCGAGTGTTTTGCGGACATCATACCAGGG 2648
YS_P._sugitai_YW44_1____            AAACCTTGTGTTGTAAAAAACTCGAGTGTTTTGCGGACATCATACCAGGG 2637
YS_P._urushiyamai_YW50_1____        AAACCTTGTGTTGTAAAAAACTCGAGTGTTTTGCGGACATCATACCAGGG 2650
YS_P._delicatulus_YW52_1____        AAACCTTGTGTTGTAAAAAACTCGAGTGTTTTGCGGACATCATACCAGGG 2657
YS_P._delicatulus_YW60_2____        AAACCTTGTGTTGTAAAAAACTCGAGTGTTTTGCGGACATCATACCAGGG 2652
YS_P._akitaorum_YW16_1____          AAACCTTGTGTTGTAAAAAACTCGAGTGTTTTGCGGACATCATACCAGGG 2651
YS_P._hongwonpyoi_YW05_8____        AAACCTTGTGTTGTAAAAAACTCGAGTGTTTTGCGGACATCATACCAGGG 2652
YS_P._hongwonpyoi_YW24_1____        AAACCTTGTGTTGTAAAAAACTCGAGTGTTTTGCGGACATCATACCAGGG 2652
YS_P._hongwonpyoi_YW33_8____        AAACCTTGTGTTGTAAAAAACTCGAGTGTTTTGCGGACATCATACCAGGG 2650
YS_P._hongwonpyoi_YW35_8____        AAACCTTGTGTTGTAAAAAACTCGAGTGTTTTGCGGACATCATACCAGGG 2652
YS_P._hongwonpyoi_YW37_8____        AAACCTTGTGTTGTAAAAAACTCGAGTGTTTTGCGGACATCATACCAGGG 2654
YS_P._hongwonpyoi_YW34_8____        AAACCTTGTGTTGTAAAAAACTCGAGTGTTTTGCGGACATCATACCAGGG 2653
YS_P._hongwonpyoi_YW34_2a___        AAACCTTGTGTTGTAAAAAACTCGAGTGTTTTGCGGACATCATACCAGGG 2655
YS_P._viridicuprus_YW15_1____       AAACCTTGTGTTGTAAAAAACTCGAGTGTTTTGCGGACATCATACCAGGG 2652
YS_P._hongwonpyoi_YW36_1____        AAACCTTGTGTTGTAAAAAACTCGAGTGTTTTGCGGACATCATACCAGGG 2654
YS_P._angularis_YW25_8___           AAACCTTGTGTTGTAAAAAGCTCGAGTGTTTTGCGGACATCATACCAGGG 2682
```

```
                                   ******************* ******************************

YS_P._acuticollis_YW07_8____       GAATTCAAAGAGGGCTATATGGATGTATTTGGTTGCGACTCGAGCGTAAA 2700
YS_P._takakuwai_YW54_1____         GAATTCAAAGAGGGCTATATGGATGTATTTGGTTGCGACTCGAGCGTAAA 2698
YS_P._albisomni_YW19_1____         GAATTCAAAGAGGGCTATATGGATGTATTTGGTTGCGACTCGAGCGTAAA 2699
YS_P._kawadai_YW53_1____           GAATTCAAAGAGGGCTATATGGATGTATTTGGTTGCGACTCGAGCGTAAA 2698
YS_P._kawadai_YW18_1____           GAATTCAAAGAGGGCTATATGGATGTATTTGGTTGCGACTCGAGCGTAAA 2698
YS_P._sue_YW45_1____               GAATTCAAAGAGGGCTATATGGATGTATTTGGTTGCGACTCGAGCGTAAA 2698
YS_P._sue_YW80_3____               GAATTCAAAGAGGGCTATATGGATGTATTTGGTTGCGACTCGAGCGTAAA 2698
YS_P._sugitai_YW44_1____           GAATTCAAAGAGGGCTATATGGATGTATTTGGTTGCGACTCGAGCGTAAA 2687
YS_P._urushiyamai_YW50_1____       GAATTCAAAGAGGGCTATATGGATGTATTTGGTTGCGACTCGAGCGTAAA 2700
YS_P._delicatulus_YW52_1____       GAATTCAAAGAGGGCTATATGGATGTATTTGGTTGCGACTCGAGCGTAAA 2707
YS_P._delicatulus_YW60_2____       GAATTCAAAGAGGGCTATATGGATGTATTTGGTTGCGACTCGAGCGTAAA 2702
YS_P._akitaorum_YW16_1____         GAATTCAAAGAGGGCTATATGGATGTATTTGGTTGCGACTCGAGCGTAAA 2701
YS_P._hongwonpyoi_YW05_8____       GAATTCAAAGAGGGCTATATGGATGTATTTGGTTGCGACTCGAGCGTAAA 2702
YS_P._hongwonpyoi_YW24_1____       GAATTCAAAGAGGGCTATATGGATGTATTTGGTTGCGACTCGAGCGTAAA 2702
YS_P._hongwonpyoi_YW33_8____       GAATTCAAAGAGGGCTATATGGATGTATTTGGTTGCGACTCGAGCGTAAA 2700
YS_P._hongwonpyoi_YW35_8____       GAATTCAAAGAGGGCTATATGGATGTATTTGGTTGCGACTCGAGCGTAAA 2702
YS_P._hongwonpyoi_YW37_8____       GAATTCAAAGAGGGCTATATGGATGTATTTGGTTGCGACTCGAGCGTAAA 2704
YS_P._hongwonpyoi_YW34_8____       GAATTCAAAGAGGGCTATATGGATGTATTTGGTTGCGACTCGAGCGTAAA 2703
YS_P._hongwonpyoi_YW34_2a___       GAATTCAAAGAGGGCTATATGGATGTATTTGGTTGCGACTCGAGCGTAAA 2705
YS_P._viridicuprus_YW15_1____      GAATTCAAAGAGGGCTATATGGATGTATTTGGTTGCGACTCGAGCGTAAA 2702
YS_P._hongwonpyoi_YW36_1____       GAATTCAAAGAGGGCTATATGGATGTATTTGGTTGCGACTCGAGCGTAAA 2704
YS_P._angularis_YW25_8___          GAATTCAAAGAGGGCTATATGGATGTATTTGGTTGCGACTCGAGCGTAAA 2732
                                   **************************************************

YS_P._acuticollis_YW07_8____       AACTTGAGACGCGAGCACATACCGATTGAATTTATTCAATTGTCAATTTA 2750
YS_P._takakuwai_YW54_1____         AACTTGAGACGCGAGCACATACCGATTGAATTTATTCAATTGTCAATTTA 2748
YS_P._albisomni_YW19_1____         AACTTGAGACGCGAGCACATACCGATTGAATTTATTCAATTGTCAATTTA 2749
YS_P._kawadai_YW53_1____           AACTTGAGACGCGAGCACATACCGATTGAATTTATTCAATTGTCAATTTA 2748
YS_P._kawadai_YW18_1____           AACTTGAGACGCGAGCACATACCGATTGAATTTATTCAATTGTCAATTTA 2748
YS_P._sue_YW45_1____               AACTTGAGACGCGAGCACATACCGATTGAATTTATTCAATTGTCAATTTA 2748
YS_P._sue_YW80_3____               AACTTGAGACGCGAGCACATACCGATTGAATTTATTCAATTGTCAATTTA 2748
YS_P._sugitai_YW44_1____           AACTTGAGACGCGAGCACATACCGATTGAATTTATTCAATTGTCAATTTA 2737
YS_P._urushiyamai_YW50_1____       AACTTGAGACGCAAGCACATACCGATTGAATTTATTCAATTGTCAATTTA 2750
YS_P._delicatulus_YW52_1____       AGCTTGAGACGCGAGCACATACCGATTGAATTTATTCAATTGTCAATTTA 2757
```

```
YS_P._delicatulus_YW60_2____        AGCTTGAGACGCGAGCACATACCGATTGAATTTATTCAATTGTCAATTTA 2752
YS_P._akitaorum_YW16_1____          AACTTGAGACGCGAGCACATACCGATTGAATTTATTCAATTGTCAATTTA 2751
YS_P._hongwonpyoi_YW05_8____        AACTTGAGACGCGAGCACATACCGATTGAATTTATTCAATTGTCAATTTA 2752
YS_P._hongwonpyoi_YW24_1____        AACTTGAGACGCGAGCACATACCGATTGAATTTATTCAATTGTCAATTTA 2752
YS_P._hongwonpyoi_YW33_8____        AACTTGAGACGCGAGCACATACCGATTGAATTTATTCAATTGTCAATTTA 2750
YS_P._hongwonpyoi_YW35_8____        AACTTGAGACGCGAGCACATACCGATTGAATTTATTCAATTGTCAATTTA 2752
YS_P._hongwonpyoi_YW37_8____        AACTTGAGACGCGAGCACATACCGATTGAATTTATTCAATTGTCAATTTA 2754
YS_P._hongwonpyoi_YW34_8____        AACTTGAGACGCGAGCACATACCGATTGAATTTATTCAATTGTCAATTTA 2753
YS_P._hongwonpyoi_YW34_2a___        AACTTGAGACGCGAGCACATACCGATTGAATTTATTCAATTGTCAATTTA 2755
YS_P._viridicuprus_YW15_1____       AACTTGAGACGCGAGCACATACCGATTGAATTTATTCAATTGTCAATTTA 2752
YS_P._hongwonpyoi_YW36_1____        AACTTGAGACGCGAGCACATACCGATTGAATTTATTCAATTGTCAATTTA 2754
YS_P._angularis_YW25_8___           AACTTGAGACGCGAGCACATACCGATTGAATTTATTCAATTGTCAATTTA 2782
                                    * ********** **************************************

YS_P._acuticollis_YW07_8____        GAAAACTTCTTGTCAGTCTGAGAACGTCAGTTCTTGGGCTGGTGATGAGA 2800
YS_P._takakuwai_YW54_1____          GAAAACTTCTTGTCAGTCTGAGAACGTCAGTTCTTGGGCTGGTGATGAGA 2798
YS_P._albisomni_YW19_1____          GAAAACTTCTTGTCAGTCTGAGAACGTCAGTTCTTGGGCTGGTGATGAGA 2799
YS_P._kawadai_YW53_1____            GAAAACTTCTTGTCAGTCTGAGAACGTCAGTTCTTGGGCTGGTGATGAGA 2798
YS_P._kawadai_YW18_1____            GAAAACTTCTTGTCAGTCTGAGAACGTCAGTTCTTGGGCTGGTGATGAGA 2798
YS_P._sue_YW45_1____                GAAAACTTCTTGTCAGTCTGAGAACGTCAGTTCTTGGGCTGGTGATGAGA 2798
YS_P._sue_YW80_3____                GAAAACTTCTTGTCAGTCTGAGAACGTCAGTTCTTGGGCTGGTGATGAGA 2798
YS_P._sugitai_YW44_1____            GAAAACTTCTTGTCAGTCTGAGAACGTCAGTTCTTGGGCTGGTGATGAGA 2787
YS_P._urushiyamai_YW50_1____        GAAAACTTCTTGTCAGTCTGAGAACGTCAGTTCTTGG-CTGGTGATGAGA 2799
YS_P._delicatulus_YW52_1____        GAAAACTTCTTGTCAGTCTGGGAACGTCAGTTCTTGGGCTGGTGATGAGA 2807
YS_P._delicatulus_YW60_2____        GAAAACTTCTTGTCAGTCTGAGAACGTCAGTTCTTGGGCTGGTGATGAGA 2802
YS_P._akitaorum_YW16_1____          GAAAACTTCTTGTCAGTCTGAGAACGTCAGTTCTTGGGCTGGTGATGAGA 2801
YS_P._hongwonpyoi_YW05_8____        GAAAACTTCTTGTCAGTCTGAGAACGTAAGTTCTTGGGCTGGTGATGAGA 2802
YS_P._hongwonpyoi_YW24_1____        GAAAACTTCTTGTCAGTCTGAGAACGTAAGTTCTTGGGCTGGTGATGAGA 2802
YS_P._hongwonpyoi_YW33_8____        GAAAACTTCTTGTCAGTCTGAGAACGTAAGTTCTTGGGCTGGTGATGAGA 2800
YS_P._hongwonpyoi_YW35_8____        GAAAACTTCTTGTCAGTCTGAGAACGTAAGTTCTTGGGCTGGTGATGAGA 2802
YS_P._hongwonpyoi_YW37_8____        GAAAACTTCTTGTCAGTCTGAGAACGTAAGTTCTTGGGCTGGTGATGAGA 2804
YS_P._hongwonpyoi_YW34_8____        GAAAACTTCTTGTCAGTCTGAGAACGTAAGTTCTTGGGCTGGTGATGAGA 2803
YS_P._hongwonpyoi_YW34_2a___        GAAAACTTCTTGTCAGTCTGAGAACGTAAGTTCTTGGGCTGGTGATGAGA 2805
YS_P._viridicuprus_YW15_1____       GAAAACTTCTTGTCAGTCTGAGAACGTAAGTTCTTGGGCTGGTGATGAGA 2802
YS_P._hongwonpyoi_YW36_1____        GAAAACTTCTTGTCAGTCTGAGAACGTAAGTTCTTGGGCTGGTGATGAGA 2804
YS_P._angularis_YW25_8___           GAAAACTTCTTGTCAGTCTGAGAACGTCAGTTCTTGGGCTGGTGATGAGA 2832
```

```
                                    ******************** ****** ********* ************

YS_P._acuticollis_YW07_8____        CTGCTTTGCAGTCTTAAGATAGTTATCTGGTTGATCCTGCCA 2842
YS_P._takakuwai_YW54_1____          CTGCTTTGCAGTCTTAAGATAGTTATCTGGTTGATCCTGCCA 2840
YS_P._albisomni_YW19_1____          CTGCTTTGCAGTCTTAAGATAGTTATCTGGTTGATCCTGCCA 2841
YS_P._kawadai_YW53_1____            CTGCTTTGCAGTCTTAAGATAGTTATCTGGTTGATCCTGCCA 2840
YS_P._kawadai_YW18_1____            CTGCTTTGCAGTCTTAAGATAGTTATCTGGTTGATCCTGCCA 2840
YS_P._sue_YW45_1____                CTGCTTTGCAGTCTTAAGATAGTTATCTGGTTGATCCTGCCA 2840
YS_P._sue_YW80_3____                CTGCTTTGCAGTCTTAAGATAGTTATCTGGTTGATCCTGCCA 2840
YS_P._sugitai_YW44_1____            CTGCTTTGCAGTCTTAAGATAGTTATCTGGTTGATCCTGCCA 2829
YS_P._urushiyamai_YW50_1____        CTGCTTTGCAGTCTTAAGATAGTTATCTGGTTGATCCTGCCA 2841
YS_P._delicatulus_YW52_1____        CTGCTTTGCAGTCTTAAGATAGTTATCTGGTTGATCCTGCCA 2849
YS_P._delicatulus_YW60_2____        CTGCTTTGCAGTCTTAAGATAGTTATCTGGTTGATCCTGCCA 2844
YS_P._akitaorum_YW16_1____          CTGCTTTGCAGTCTTAAGATAGTTATCTGGTTGATCCTGCCA 2843
YS_P._hongwonpyoi_YW05_8____        CTGCTTTGCAGTCTTAAGATAGTTATCTGGTTGATCCTGCCA 2844
YS_P._hongwonpyoi_YW24_1____        CTGCTTTGCAGTCTTAAGATAGTTATCTGGTTGATCCTGCCA 2844
YS_P._hongwonpyoi_YW33_8____        CTGCTTTGCAGTCTTAAGATAGTTATCTGGTTGATCCTGCCA 2842
YS_P._hongwonpyoi_YW35_8____        CTGCTTTGCAGTCTTAAGATAGTTATCTGGTTGATCCTGCCA 2844
YS_P._hongwonpyoi_YW37_8____        CTGCTTTGCAGTCTTAAGATAGTTATCTGGTTGATCCTGCCA 2846
YS_P._hongwonpyoi_YW34_8____        CTGCTTTGCAGTCTTAAGATAGTTATCTGGTTGATCCTGCCA 2845
YS_P._hongwonpyoi_YW34_2a___        CTGCTTTGCAGTCTTAAGATAGTTATCTGGTTGATCCTGCCA 2847
YS_P._viridicuprus_YW15_1____       CTGCTTTGCAGTCTTAAGATAGTTATCTGGTTGATCCTGCCA 2844
YS_P._hongwonpyoi_YW36_1____        CTGCTTTGCAGTCTTAAGATAGTTATCTGGTTGATCCTGCCA 2846
YS_P._angularis_YW25_8___           CTGCTTTGCAGTCTTAAGATAGTTATCTGGTTGATCCTGCCA 2874
                                    ******************************************
```
