# Supplementary material for: Evolutionary Relationship Between Platycerus Stag Beetles and Their Mycangium-Associated Yeast Symbionts
Source: Front Microbiol. 2020 Jun 30;11:1436. doi: 10.3389/fmicb.2020.01436 (PMC7338584; doi:10.3389/fmicb.2020.01436)
Supplement: Supplementary file 9 [file Data_Sheet_9.PDF]

SI Appendix 4. The sequence alignment used for the COI phylogenetic analysis using ClustalW.

```
P._acuticollis_site_5_________      TTTGGGATAATCTCTCATATCATTAGACAAGAAAGACAAAAAAAAGAAAC 50
P._takakuwai_site_6__________       TTTGGAATAATCTCTCATATCATTAGACAAGAAAGACAAAAAAAAGAAAC 50
P._takakuwai_site_7___________      TTTGGAATAATCTCTCATATCATTAGACAAGAAAGACAAAAAAAAGAAAC 50
P._takakuwai_site_8___________      TTTGGGATAATCTCTCATATCATTAGACAAGAAAGACAAAAAAAAGAAAC 50
P._albisomni_site_9___________      TTTGGGATAATCTCTCATATCATTAGACAAGAAAGACAAAAAAAAGAAAC 50
P._albisomni_site_3___________      TTTGGGATAATCTCTCATATCATTAGACAAGAAAGACAAAAAAAAGAAAC 50
P._albisomni_site_1___________      TTTGGAATAATCTCTCATATCATTAGACAAGAAAGACAAAAAAAAGAAAC 50
P._albisomni_site_4___________      TTCGGAATAATCTCTCATATCATTAGACAAGAAAGACAAAAAAAAGAAAC 50
P._takakuwai_site_10__________      TTTGGAATAATCTCTCATATCATTAGACAGGAAAGACAAAAAAAAGAAAC 50
P._acuticollis_site_2_________      TTTGGAATAATCTCTCATATCATTAGACAAGAAAGACAAAAAAAAGAAAC 50
P._viridicuprus_site_15_______      TTCGGAATAATCTCTCATATCATTAGACAAGAAAGACAAAAAAAAGAAAC 50
P._viridicuprus_site_16_______      TTCGGAATAATCTCTCATATCATTAGACAAGAGAGACAAAAAAAAGAAAC 50
P._sue_site_22_____________         TTCGGGATAATCTCTCATATCATTAGACAAGAAAGACAAAAAAAAGAAAC 50
P._viridicuprus_site_23_______      TTCGGAATAATCTCTCATATCATTAGACAAGAGAGACAAAAAAAAGAAAC 50
P._viridicuprus_site_25_______      TTCGGAATAATCTCTCATATCATTAGACAAGAGAGACAAAAAAAAGAAAC 50
P._viridicuprus_site_17_______      TTCGGAATAATCTCTCATATCATTAGACAAGAAAGACAAAAAAAAGAAAC 50
P._takakuwai_site_14__________      TTTGGGATAATCTCCCACATTATTAGACAAGAAAGACAAAAAAAAGAAAC 50
P._akitaorum_site_13__________      TTTGGGATAATCTCCCACATTATTAGACAAGAAAGACAAAAAAAAGAAAC 50
P._takakuwai_site_12__________      TTTGGGATAATCTCTCACATCATTAGACAAGAAAGACAAAAAAAAGAAAC 50
P._takakuwai_site_18__________      TTTGGGATAATCTCCCACATCATTAGACAAGAAAGACAAAAAAAAGAAAC 50
P._sugitai_site_19____________      TTTGGAATAATCTCCCACATCATTAGGCAAGAAAGACAAAAAAAAGAAAC 50
P._sugitai_site_21____________      TTTGGGATAATCTCCCACATCATTAGACAAGAAAGACAAAAAAAAGAAAC 50
P._urushiyamai_site_29________      TTTGGAATAATTTCCCACATCATTAGACAAGAAAGACAAAAAAAAGAAAC 50
P._urushiyamai_site_30________      TTTGGGATAATTTCCCACATCATTAGACAAGAAAGACAAAAAAAAGAAAC 50
P._urushiyamai_site_24________      TTTGGGATAATTTCCCACATCATTAGACAAGAAAGACAAAAAAAAGAAAC 50
P._kawadai_site_10__________        TTTGGGATAATCTCCCACATTATTAGACAAGAAAGACAAAAAAAAGAAAC 50
P._delicatulus_site_2_________      TTTGGGATAATCTCCCACATTATTAGACAAGAAAGACAAAAAAAAGAAAC 50
P._delicatulus_site_6_________      TTTGGGATAATCTCCCACATTATTAGACAAGAAAGACAAAAAAAAGAAAC 50
P._delicatulus_site_20________      TTTGGGATAATCTCCCACATTATTAGACAAGAAAGACAAAAAAAAGAAAC 50
P._delicatulus_site_25________      TTTGGAATAATCTCCCATATTATTAGACAAGAAAGACAAAAAAAAGAAAC 50
P._delicatulus_site_28________      TTTGGAATAATCTCCCATATTATTAGACAAGAAAGACAAAAAAAAGAAAC 50
P._kawadai_site_6___________        TTTGGGATAATCTCTCACATTATTAGACAAGAAAGACAAAAAAAAGAAAC 50
P._kawadai_site_7_____________      TTTGGAATAATCTCTCACATTATTAGACAAGAAAGACAAAAAAAAGAAAC 50
P._kawadai_site_8_____________      TTTGGGATGATCTCTCACATTATTAGACAAGAAAGACAAAAAAAAGAAAC 50
```

```
P._delicatulus_site_26________       TTTGGGATAATCTCCCACATCATTAGACAAGAAAGACAAAAAAAAGAAAC 50
P._delicatulus_site_27________       TTTGGGATAATCTCCCACATCATTAGACAAGAAAGACAAAAAAAAGAAAC 50
P._takakuwai_site_11__________       TTTGGGATAATTTCCCACATCATTAGACAAGAAAGACAAAAAAAAGAAAC 50
P._oregonensis_sanmaeto_______       TTCGGAATAATTTCTCATATTATTAGGCAAGAAAGTATAAAAAAAGAAAC 50
P._hongwonpyoi_birosa_________       TTTGGGATAATTTCCCATATTATTAGACAAGAAAGAATAAAAAAAGAAAC 50
P._hongwonpyoi_jengoge________       TTTGGGATAATTTCCCATATTATTAGACAAGAAAGAATAAAAAAAGAAAC 50
P._hongwonpyoi_deogyusan______       TTTGGCATAATCTCCCATATTATTAGACAAGAAAGGATAAAAAAAGAAAC 50
P._hongwonpyoi_hangeryeong____       TTTGGTATAATTTCCCATATTATTAGACAAGAAAGAATAAAAAAAGAAAC 50
P._hongwonpyoi_nogodan________       TTTGGTATAATTTCCCATATTATTAGACAAGAAAGGAGGAAAAAAGAAAC 50
P._hongwonpyoi_jeoksangsan____       TTTGGGATAATCTCCCATATTATTAGACAAGAAAGTATAAAAAAAAGAAC 50
                                     ** ** ** ** ** ** ** ***** ** ** **    ******  ***

P._acuticollis_site_5_________       ATTTGGAACTCTTGGAATAATTTATGCTATAATAGCAATTGGATTATTAG 100
P._takakuwai_site_6_________         ATTTGGAACTCTTGGAATAATTTATGCTATGATAGCAATTGGATTATTAG 100
P._takakuwai_site_7___________       ATTTGGAACTCTTGGAATAATTTATGCTATAATAGCAATTGGATTATTAG 100
P._takakuwai_site_8___________       ATTTGGAACTCTTGGAATAATTTATGCTATAATAGCAATTGGATTATTAG 100
P._albisomni_site_9___________       ATTTGGAACTCTTGGAATAATTTATGCTATGATAGCAATTGGATTATTAG 100
P._albisomni_site_3___________       ATTTGGAACTCTTGGAATAATTTATGCTATGATAGCAATTGGATTATTAG 100
P._albisomni_site_1___________       ATTTGGAACTCTTGGAATAATTTATGCTATGATAGCAATTGGATTATTAG 100
P._albisomni_site_4___________       ATTTGGAACTCTTGGAATAATTTATGCTATAATAGCAATTGGATTATTAG 100
P._takakuwai_site_10__________       ATTTGGAACTCTTGGAATAATTTATGCTATAATAGCAATTGGATTATTAG 100
P._acuticollis_site_2_________       ATTTGGAACTCTTGGAATAATTTATGCTATAATAGCAATTGGATTATTAG 100
P._viridicuprus_site_15_______       ATTTGGAACTCTTGGAATAATTTATGCTATAATAGCAATTGGATTATTAG 100
P._viridicuprus_site_16_______       ATTTGGAACTCTTGGAATAATTTATGCTATAATAGCAATTGGATTATTAG 100
P._sue_site_22_____________          ATTTGGAACTCTTGGAATAATCTATGCTATAATAGCAATTGGATTATTAG 100
P._viridicuprus_site_23_______       ATTTGGAACTCTTGGAATAATTTATGCTATAATAGCAATTGGATTATTAG 100
P._viridicuprus_site_25_______       ATTTGGGACTCTTGGAATAATTTATGCTATAATAGCAATTGGATTATTAG 100
P._viridicuprus_site_17_______       ATTTGGAACTCTTGGAATAATTTATGCTATAATAGCAATTGGGTTATTAG 100
P._takakuwai_site_14__________       ATTTGGAACCCTAGGAATAATTTATGCCATAATAGCAATTGGACTATTAG 100
P._akitaorum_site_13__________       ATTTGGAACCCTAGGAATAATTTATGCCATAATAGCAATTGGACTATTAG 100
P._takakuwai_site_12__________       ATTTGGAACCCTAGGAATAATTTATGCCATAATAGCAATTGGACTATTAG 100
P._takakuwai_site_18__________       ATTTGGAACCCTAGGAATAATTTATGCCATAATAGCAATTGGACTATTAG 100
P._sugitai_site_19____________       ATTTGGAACCCTAGGAATAATTTATGCAATAATAGCAATTGGACTATTAG 100
P._sugitai_site_21____________       ATTTGGAACCCTAGGAATAATTTATGCCATAATAGCAATTGGTCTATTAG 100
P._urushiyamai_site_29________       ATTTGGGACCCTAGGAATAATTTATGCTATAATAGCAATTGGGCTATTGG 100
P._urushiyamai_site_30________       ATTTGGGACCCTAGGAATAATTTATGCTATAATAGCAATTGGGCTATTGG 100
```

```
P._urushiyamai_site_24________       ATTTGGGACCCTAGGAATAATTTATGCTATAATAGCAATTGGGCTATTGG 100
P._kawadai_site_10__________         ATTTGGAACCCTAGGAATAATTTATGCTATAATAGCAATTGGACTATTAG 100
P._delicatulus_site_2_________       ATTTGGAACCCTAGGAATAATCTATGCTATAATAGCAATTGGGCTATTAG 100
P._delicatulus_site_6_________       ATTTGGAACCCTAGGAATAATCTATGCTATAATAGCAATTGGACTATTAG 100
P._delicatulus_site_20________       ATTTGGAACCCTAGGAATAATTTATGCTATAATAGCAATTGGACTATTAG 100
P._delicatulus_site_25________       ATTTGGAACCCTAGGAATAATTTATGCTATAATAGCAATTGGACTATTAG 100
P._delicatulus_site_28________       ATTTGGAACCCTAGGAATAATTTATGCTATAATAGCAATTGGACTATTAG 100
P._kawadai_site_6___________         ATTTGGAACCCTAGGAATAATTTATGCTATAATAGCAATTGGACTATTAG 100
P._kawadai_site_7_____________       ATTTGGAACCCTAGGGATAATTTATGCTATAATAGCAATTGGACTATTAG 100
P._kawadai_site_8_____________       ATTTGGAACCCTAGGAATAATTTATGCTATAATAGCAATTGGACTATTAG 100
P._delicatulus_site_26________       ATTTGGAACCCTAGGAATAATTTATGCTATAATAGCAATTGGACTATTAG 100
P._delicatulus_site_27________       ATTTGGAACCCTAGGAATAATTTATGCTATAATAGCAATTGGACTATTAG 100
P._takakuwai_site_11__________       ATTTGGAACCCTTGGAATAATTTATGCTATAATAGCAATTGGACTATTAG 100
P._oregonensis_sanmaeto_______       ATTTGGAACACTAGGTATAATTTATGCTATACTAGCAATTGGTTTACTAG 100
P._hongwonpyoi_birosa_________       ATTTGGGACCCTGGGAATAATTTATGCAATAATAGCAATTGGATTATTAG 100
P._hongwonpyoi_jengoge________       ATTTGGGACCCTGGGAATAATTTATGCAATAATAGCAATTGGATTATTAG 100
P._hongwonpyoi_deogyusan______       ATTTGGGACCCTAGGGATAATTTATGCAATAATAGCAATCGGATTATTAG 100
P._hongwonpyoi_hangeryeong____       ATTTGGAACTCTAGGAATAATTTATGCAATAATAGCAATTGGGCTATTAG 100
P._hongwonpyoi_nogodan________       ATTTGGAACTCTGGGAATAATTTATGCAATAATAGCAATTGGATTACTAG 100
P._hongwonpyoi_jeoksangsan____       ATTCGGAACTCTAGGAATAATTTATGCAATAATAGCAATTGGATTATTAG 100
                                     *** ** ** ** ** ***** ***** **  ******* **  ** * *

P._acuticollis_site_5_________       GATTCATTGTTTGAGCACATCATATATTTACAGTAGGAATAGATGTAGAC 150
P._takakuwai_site_6_________         GATTCATTGTTTGAGCACATCATATATTTACAGTAGGAATAGATGTAGAC 150
P._takakuwai_site_7___________       GATTCATTGTTTGAGCACATCATATATTTACAGTAGGAATAGATGTAGAC 150
P._takakuwai_site_8___________       GATTCATTGTTTGAGCACATCATATATTTACAGTAGGAATAGATGTTGAC 150
P._albisomni_site_9___________       GATTCATTGTTTGAGCACATCATATATTTACAGTAGGAATAGATGTAGAC 150
P._albisomni_site_3___________       GATTCATTGTTTGAGCACATCATATATTTACAGTAGGAATAGATGTAGAC 150
P._albisomni_site_1___________       GATTCATTGTTTGAGCACACCATATATTTACAGTAGGAATAGATGTAGAC 150
P._albisomni_site_4___________       GATTCATTGTTTGAGCACATCATATATTTACAGTAGGAATAGATGTAGAC 150
P._takakuwai_site_10__________       GATTCATTGTTTGAGCACATCATATATTTACAGTAGGAATAGATGTAGAC 150
P._acuticollis_site_2_________       GATTCATTGTTTGAGCACATCATATATTTACAGTAGGAATAGATGTAGAT 150
P._viridicuprus_site_15_______       GATTTATTGTTTGAGCACATCATATGTTTACAGTAGGAATAGATGTAGAT 150
P._viridicuprus_site_16_______       GATTTATTGTTTGAGCACATCATATGTTTACAGTAGGAATAGATGTAGAT 150
P._sue_site_22_____________          GATTTATTGTTTGAGCACATCATATGTTTACAGTAGGAATAGATGTAGAT 150
P._viridicuprus_site_23_______       GATTTATTGTTTGAGCACATCATATATTTACAGTAGGAATAGATGTAGAT 150
```

```
P._viridicuprus_site_25_______      GATTTATTGTTTGAGCACATCATATATTTACAGTAGGAATAGATGTAGAT 150
P._viridicuprus_site_17_______      GATTTATTGTTTGAGCACATCATATATTTACAGTAGGAATAGATGTAGAT 150
P._takakuwai_site_14__________      GATTTATTGTTTGAGCACACCATATATTTACAGTAGGAATAGATGTAGAT 150
P._akitaorum_site_13__________      GATTTATTGTTTGAGCACACCATATATTTACAGTAGGAATAGATGTAGAT 150
P._takakuwai_site_12__________      GATTTATTGTTTGAGCACACCATATATTTACAGTAGGAATAGATGTAGAT 150
P._takakuwai_site_18__________      GATTTATTGTTTGAGCACACCATATATTTACAGTAGGAATAGATGTAGAT 150
P._sugitai_site_19____________      GATTTATTGTTTGAGCACACCATATATTTACAGTAGGAATAGATGTAGAT 150
P._sugitai_site_21____________      GATTTATTGTTTGAGCACACCATATATTTACAGTAGGAATAGATGTAGAT 150
P._urushiyamai_site_29________      GATTTATTGTTTGAGCACACCATATATTCACAGTGGGAATAGACGTAGAT 150
P._urushiyamai_site_30________      GATTTATTGTTTGAGCACACCATATATTCACAGTGGGAATAGACGTAGAT 150
P._urushiyamai_site_24________      GATTTATTGTTTGAGCACACCATATATTCACAGTGGGGATAGACGTAGAT 150
P._kawadai_site_10__________        GATTTATTGTTTGAGCACACCACATATTCACAGTAGGAATAGATGTAGAT 150
P._delicatulus_site_2_________      GATTTATTGTTTGAGCACACCATATATTCACAGTAGGAATAGATGTAGAT 150
P._delicatulus_site_6_________      GATTTATTGTTTGAGCACACCATATATTCACAGTAGGAATAGATGTAGAT 150
P._delicatulus_site_20________      GATTCATTGTTTGAGCACACCATATATTCACAGTAGGAATAGATGTAGAT 150
P._delicatulus_site_25________      GATTTATTGTTTGAGCACACCATATATTCACAGTAGGAATAGATGTAGAT 150
P._delicatulus_site_28________      GATTTATTGTTTGAGCACACCATATATTCACAGTAGGAATAGATGTAGAT 150
P._kawadai_site_6___________        GATTTATTGTTTGAGCACACCATATATTCACAGTAGGAATAGATGTAGAT 150
P._kawadai_site_7_____________      GATTTATTGTTTGAGCACACCATATATTCACAGTAGGAATAGATGTAGAT 150
P._kawadai_site_8_____________      GATTTATTGTTTGAGCACACCATATATTCACAGTAGGAATAGATGTAGAT 150
P._delicatulus_site_26________      GATTTATTGTTTGAGCACACCATATATTCACAGTAGGAATAGATGTAGAT 150
P._delicatulus_site_27________      GATTTATTGTTTGAGCACACCATATATTCACAGTAGGAATAGATGTAGAT 150
P._takakuwai_site_11__________      GATTTATTGTTTGAGCACACCATATATTCACAGTAGGGATAGATGTAGAT 150
P._oregonensis_sanmaeto_______      GATTTATTGTTTGAGCTCACCATATGTTTACAGTAGGAATAGATGTGGAT 150
P._hongwonpyoi_birosa_________      GATTTATTGTCTGAGCTCACCATATATTTACAGTAGGAATAGATGTTGAT 150
P._hongwonpyoi_jengoge________      GATTTATTGTCTGAGCTCACCATATATTTACAGTAGGAATAGATGTTGAT 150
P._hongwonpyoi_deogyusan______      GGTTTATTGTCTGAGCCCACCATATATTTACAGTAGGGATAGATGTCGAT 150
P._hongwonpyoi_hangeryeong____      GATTTATTGTCTGAGCTCACCATATATTTACAGTAGGAATAGATGTTGAT 150
P._hongwonpyoi_nogodan________      GATTTATTGTTTGAGCTCACCATATATTTACAGTGGGAATAGATGTTGAT 150
P._hongwonpyoi_jeoksangsan____      GATTTATCGTTTGAGCTCACCATATATTTACAGTGGGAATAGATGTCGAT 150
                                    * ** ** ** ***** ** ** ** ** ***** ** ***** ** **

P._acuticollis_site_5_________      ACACGAGCCTACTTTACATCTGCCACAATAATCATTGCAGTTCCTACAGG 200
P._takakuwai_site_6__________       ACACGAGCCTACTTTACATCTGCCACAATAATCATTGCAGTTCCTACAGG 200
P._takakuwai_site_7___________      ACACGAGCCTACTTTACATCTGCCACAATAATCATTGCAGTTCCTACAGG 200
P._takakuwai_site_8___________      ACACGAGCCTACTTTACATCTGCGGTTCCTACAGG 200
```

```
P._albisomni_site_9___________      ACACGAGCCTACTTTACATCTGCCACAATAATCATTGCAGTTCCCACAGG 200
P._albisomni_site_3___________      ACACGAGCCTACTTTACATCTGCCACAATAATCATTGCAGTTCCCACAGG 200
P._albisomni_site_1___________      ACACGAGCCTACTTTACATCTGCCACAATAATCATTGCAGTTCCCACAGG 200
P._albisomni_site_4___________      ACACGAGCCTACTTCACATCTGCCACAATAATCATTGCAGTTCCCACAGG 200
P._takakuwai_site_10__________      ACACGAGCCTACTTTACATCTGCCACAATAATCATTGCAGTTCCCACAGG 200
P._acuticollis_site_2_________      ACACGAGCCTACTTTACATCTGCCACAATAATCATTGCAGTTCCCACAGG 200
P._viridicuprus_site_15_______      ACACGAGCCTATTTTACATCTGCCACAATAATTATTGCAGTTCCTACGGG 200
P._viridicuprus_site_16_______      ACACGAGCCTATTTTACATCTGCCACAATAATTATTGCAGTTCCTACGGG 200
P._sue_site_22_____________         ACACGAGCCTATTTTACATCTGCCACAATAATTATTGCAGTTCCTACGGG 200
P._viridicuprus_site_23_______      ACACGGGCCTATTTTACATCTGCCACAATAATTATTGCAGTTCCTACAGG 200
P._viridicuprus_site_25_______      ACACGGGCCTATTTTACATCTGCCACAATAATTATTGCAGTTCCTACAGG 200
P._viridicuprus_site_17_______      ACACGGGCCTATTTTACATCTGCCACAATAATTATTGCAGTTCCTACAGG 200
P._takakuwai_site_14__________      ACACGAGCATATTTTACATCTGCTACAATAATTATTGCAGTTCCGACAGG 200
P._akitaorum_site_13__________      ACACGAGCATATTTTACATCTGCTACAATAATTATTGCAGTTCCGACAGG 200
P._takakuwai_site_12__________      ACACGGGCATATTTTACATCTGCTACAATAATTATTGCAGTTCCGACAGG 200
P._takakuwai_site_18__________      ACACGAGCATATTTTACATCTGCTACAATAATTATTGCAGTTCCGACAGG 200
P._sugitai_site_19____________      ACACGAGCATATTTTACATCTGCTACAATAATTATTGCAGTTCCGACAGG 200
P._sugitai_site_21____________      ACACGAGCATATTTTACATCTGCTACAATAATTATTGCAGTTCCGACAGG 200
P._urushiyamai_site_29________      ACACGAGCATACTTTACATCTGCTACAATAATTATTGCAGTTCCGACAGG 200
P._urushiyamai_site_30________      ACACGAGCATACTTTACATCTGCTACAATAATTATTGCAGTTCCGACAGG 200
P._urushiyamai_site_24________      ACACGAGCATACTTTACATCTGCTACAATAATTATTGCAGTTCCGACAGG 200
P._kawadai_site_10__________        ACACGAGCATATTTTACATCTGCTACTATAATTATTGCAGTTCCGACAGG 200
P._delicatulus_site_2_________      ACACGAGCATATTTTACATCTGCTACAATAATTATTGCAGTTCCAACAGG 200
P._delicatulus_site_6_________      ACACGAGCATATTTTACATCTGCTACAATAATTATTGCAGTTCCAACAGG 200
P._delicatulus_site_20________      ACACGGGCATATTTTACATCTGCTACAATAATTATTGCAGTTCCAACAGG 200
P._delicatulus_site_25________      ACACGAGCATATTTTACATCTGCTACAATAATTATTGCAGTTCCAACAGG 200
P._delicatulus_site_28________      ACACGAGCATATTTTACATCTGCTACAATAATTATTGCAGTTCCAACAGG 200
P._kawadai_site_6___________        ACACGAGCATATTTTACATCTGCTACAATAATTATTGCAGTTCCGACAGG 200
P._kawadai_site_7_____________      ACACGAGCATATTTTACATCTGCTACAATAATTATTGCAGTTCCGACAGG 200
P._kawadai_site_8_____________      ACACGAGCATATTTTACATCTGCTACAATAATTATTGCAGTTCCGACAGG 200
P._delicatulus_site_26________      ACACGAGCATATTTTACATCTGCCACAATAATTATTGCAGTTCCTACAGG 200
P._delicatulus_site_27________      ACACGAGCATATTTTACATCTGCCACAATAATTATTGCAGTTCCTACAGG 200
P._takakuwai_site_11__________      ACACGAGCATATTTTACATCTGCTACAATAATTATTGCAGTTCCAACAGG 200
P._oregonensis_sanmaeto_______      ACACGAGCTTATTTTACATCAGCAACTATAATTATTGCTGTACCAACAGG 200
P._hongwonpyoi_birosa_________      ACCCGAGCTTACTTTACGTCCGCTACTATAATTATTGCAGTACCTACAGG 200
P._hongwonpyoi_jengoge________      ACCCGAGCTTACTTTACGTCCGCTACTATAATTATTGCAGTACCTACAGG 200
```

```
P._hongwonpyoi_deogyusan______      ACCCGAGCTTATTTTACATCCGCTACTATAATTATTGCAGTACCCACTGG 200
P._hongwonpyoi_hangeryeong____      ACCCGGGCTTATTTTACATCCGCTACTATAATTATTGCAGTACCTACAGG 200
P._hongwonpyoi_nogodan________      ACCCGAGCTTATTTTACGTCCGCTACTATAATTATTGCAGTACCTACAGG 200
P._hongwonpyoi_jeoksangsan____      ACCCGAGCTTATTTTACGTCCGCTACTATAATTATTGCAGTCCCTACAGG 200
                                    ** ** ** ** ** ** ** ** ** ***** ***** ** ** ** **

P._acuticollis_site_5_________      AATTAAAATTTTCAGATGATTGGCTACACTTCACGGAACACAAATTAATT 250
P._takakuwai_site_6__________       AATTAAAATTTTCAGATGATTGGCTACACTTCACGGAACACAAATTAATT 250
P._takakuwai_site_7___________      AATTAAAATTTTCAGATGATTGGCTACACTTCACGGAACACAAATTAATT 250
P._takakuwai_site_8___________      AATTAAAATTTTCAGATGATTGGCTACACTTCACGGAACACAAATTAATT 250
P._albisomni_site_9___________      AATTAAAATTTTCAGATGATTAGCTACACTTCACGGAACACAAATTAATT 250
P._albisomni_site_3___________      AATTAAAATTTTCAGATGATTAGCTACACTTCACGGAACACAAATTAATT 250
P._albisomni_site_1___________      AATTAAAATTTTCAGATGATTAGCTACACTTCACGGAACACAAATTAATT 250
P._albisomni_site_4___________      AATTAAGATTTTCAGATGATTAGCTACACTCCACGGGACACAAATTAATT 250
P._takakuwai_site_10__________      AATTAAAATTTTCAGATGATTAGCTACACTTCACGGAACACAAATTAATT 250
P._acuticollis_site_2_________      AATTAAGATTTTCAGATGATTAGCTACACTCCACGGAACACAAATTAATT 250
P._viridicuprus_site_15_______      AATTAAAATTTTTAGATGATTAGCTACACTTCACGGAACACAAATTAATT 250
P._viridicuprus_site_16_______      AATTAAAATTTTTAGATGATTAGCTACACTTCACGGAACACAAATTAATT 250
P._sue_site_22_____________         AATTAAAATTTTTAGATGATTAGCTACACTTCACGGAACACAAATTAATT 250
P._viridicuprus_site_23_______      AATTAAAATTTTTAGATGATTAGCTACACTTCACGGAACACAAATTAATT 250
P._viridicuprus_site_25_______      AATTAAAATTTTTAGATGATTAGCTACACTTCACGGAACACAAATTAATT 250
P._viridicuprus_site_17_______      AATTAAAATTTTTAGATGATTAGCTACACTTCACGGAACACAAATTAATT 250
P._takakuwai_site_14__________      AATCAAAATTTTCAGATGATTAGCTACACTACACGGAACACAAATTAATT 250
P._akitaorum_site_13__________      AATCAAAATTTTCAGATGATTAGCTACACTACACGGAACACAAATTAATT 250
P._takakuwai_site_12__________      AATTAAAATTTTCAGATGATTAGCTACACTACACGGAACACAAATTAATT 250
P._takakuwai_site_18__________      AATCAAAATTTTCAGATGATTAGCTACACTACATGGAACACAAATTAATT 250
P._sugitai_site_19____________      AATCAAAATTTTCAGATGATTAGCTACACTACACGGAACACAAATTAATT 250
P._sugitai_site_21____________      AATCAAAATTTTCAGATGATTAGCTACACTCCACGGAACTCAAATTAATT 250
P._urushiyamai_site_29________      AATCAAAGTTTTCAGATGATTAGCTACGCTACACGGAACACAAATTAATT 250
P._urushiyamai_site_30________      AATCAAAGTTTTCAGATGATTAGCTACGCTACACGGAACACAAATTAATT 250
P._urushiyamai_site_24________      AATCAAAGTTTTCAGATGATTAGCTACTCTACACGGAACACAAATTAATT 250
P._kawadai_site_10__________        AATCAAAATTTTCAGATGATTGGCTACACTACACGGAACACAAATTAATT 250
P._delicatulus_site_2_________      AATCAAAATTTTCAGATGATTAGCTACACTACACGGAACACAAATTAATT 250
P._delicatulus_site_6_________      AATCAAAATTTTCAGATGATTAGCTACACTACACGGAACACAAATTAATT 250
P._delicatulus_site_20________      AATCAAAATTTTCAGATGATTAGCTACACTACACGGAACACAAATTAATT 250
P._delicatulus_site_25________      AATCAAAATTTTCAGATGATTAGCTACGCTTCACGGAACACAAATTAATT 250
```

```
P._delicatulus_site_28________      AATCAAAATTTTCAGATGATTAGCTACGCTTCACGGAACACAAATTAATT 250
P._kawadai_site_6___________        AATCAAAATTTTCAGATGATTAGCTACACTACACGGAACACAAATTAATT 250
P._kawadai_site_7_____________      AATCAAAATTTTCAGATGATTAGCTACACTACACGGAACACAAATTAATT 250
P._kawadai_site_8_____________      AATCAAAATTTTCAGATGATTAGCTACACTACACGGAACACAAATTAATT 250
P._delicatulus_site_26________      AATCAAAATTTTCAGATGATTAGCTACACTACACGGAACACAAATTAATT 250
P._delicatulus_site_27________      AATCAAAATTTTCAGATGATTAGCTACACTACACGGAACACAAATTAATT 250
P._takakuwai_site_11__________      AATCAAAATTTTTAGATGATTAGCTACACTACACGGAACACAAATTAATT 250
P._oregonensis_sanmaeto_______      AATTAAAATTTTTAGTTGATTAGCCACCCTCCATGGAACACAAATTAACT 250
P._hongwonpyoi_birosa_________      AATCAAAATTTTTAGATGATTAGCCACTCTTCATGGAACACAAATTAATT 250
P._hongwonpyoi_jengoge________      AATCAAAATTTTTAGATGATTAGCCACTCTTCATGGAACACAAATTAATT 250
P._hongwonpyoi_deogyusan______      AATCAAAATTTTTAGGTGATTAGCCACCCTTCATGGAACACAAATTAATT 250
P._hongwonpyoi_hangeryeong____      AATCAAAATTTTTAGATGATTAGCCACTCTCCATGGAACACAAATTAATT 250
P._hongwonpyoi_nogodan________      AATCAAAATTTTTAGATGATTAGCTACTCTTCATGGAACACAAATTAATT 250
P._hongwonpyoi_jeoksangsan____      AATCAAAATTTTTAGATGATTAGCCACTCTTCATGGAACACAAATTAATT 250
                                    *** **  **** ** ***** ** ** ** ** ** ** ******** *

P._acuticollis_site_5_________      ATTCACCTTCAATAATTTGAGCAATTGGATTCGTATTTTTATTTACAGTA 300
P._takakuwai_site_6__________       ATTCACCTTCAATAATTTGAGCAATTGGATTCGTATTTTTATTTACAGTA 300
P._takakuwai_site_7___________      ATTCACCTTCAATAATTTGGGCAATTGGGTTCGTATTTTTATTTACAGTA 300
P._takakuwai_site_8___________      ATTCACCTTCAATAATTTGAGCAATTGGGTTTGTATTTTTATTTACAGTA 300
P._albisomni_site_9___________      ATTCACCTTCAATAATTTGAGCAATTGGATTCGTATTCTTATTTACAGTA 300
P._albisomni_site_3___________      ATTCACCTTCAATAATTTGAGCAATTGGATTCGTATTCTTATTTACAGTA 300
P._albisomni_site_1___________      ATTCACCTTCAATAATTTGAGCAATTGGATTCGTATTCTTATTTACAGTA 300
P._albisomni_site_4___________      ATTCACCTTCAATAATTTGAGCAATTGGATTCGTATTTTTATTTACAGTA 300
P._takakuwai_site_10__________      ATTCACCTTCAATAATTTGAGCAATTGGATTCGTATTTTTATTTACAGTA 300
P._acuticollis_site_2_________      ACTCACCTTCAATAATTTGAGCAATTGGATTCGTATTTTTATTTACAGTA 300
P._viridicuprus_site_15_______      ATTCACCTTCAATAATTTGAGCAATTGGATTTGTATTCTTATTCACAGTA 300
P._viridicuprus_site_16_______      ATTCACCTTCAATAATCTGAGCAATTGGATTTGTATTCTTATTCACAGTA 300
P._sue_site_22_____________         ACTCACCCTCAATAATTTGAGCAATTGGATTTGTATTCTTATTCACAGTA 300
P._viridicuprus_site_23_______      ACTCACCTTCAATAATTTGAGCAATTGGATTTGTATTTTTATTTACAGTA 300
P._viridicuprus_site_25_______      ACTCACCTTCAATAATTTGAGCAATTGGATTTGTATTTTTATTTACAGTA 300
P._viridicuprus_site_17_______      ATTCACCTTCAATAATTTGAGCAATTGGATTTGTATTTTTATTCACAGTA 300
P._takakuwai_site_14__________      ATTCACCTTCTATAATTTGAGCAATTGGATTTGTATTTTTATTTACAGTA 300
P._akitaorum_site_13__________      ATTCACCTTCTATAATTTGAGCAATTGGATTTGTATTTTTATTTACAGTA 300
P._takakuwai_site_12__________      ATTCACCTTCTATAATTTGAGCAATTGGATTTGTATTTTTATTTACAGTA 300
P._takakuwai_site_18__________      ATTCACCTTCTATAATTTGAGCAATTGGATTTGTATTTTTATTTACAGTA 300
```

```
P._sugitai_site_19___________           ATTCACCTTCTATAATTTGAGCAATTGGATTTGTATTTTTATTTACAGTA 300
P._sugitai_site_21___________           ATTCGCCTTCTATAATTTGAGCAATTGGATTTGTATTTTTATTTACAGTA 300
P._urushiyamai_site_29_______           ATTCACCTTCTATAATTTGAGCAATTGGATTTGTATTTTTATTTACAGTA 300
P._urushiyamai_site_30_______           ATTCACCTTCTATAATTTGAGCAATTGGATTTGTATTTTTATTTACAGTA 300
P._urushiyamai_site_24_______           ATTCACCTTCTATAATTTGAGCAATTGGATTTGTATTTTTATTTACAGTA 300
P._kawadai_site_10_________             ATTCACCTTCTATAATTTGAGCAATTGGATTTGTATTTTTATTTACAGTA 300
P._delicatulus_site_2________           ATTCACCTTCTATAATTTGAGCAATTGGATTTGTATTTTTATTTACAGTA 300
P._delicatulus_site_6________           ATTCACCTTCTATAATTTGAGCAATTGGGTTTGTATTTTTATTTACAGTA 300
P._delicatulus_site_20_______           ATTCACCTTCTATAATTTGAGCAATTGGGTTTGTATTTTTATTTACAGTA 300
P._delicatulus_site_25_______           ATTCACCTTCTATAATTTGAGCAATTGGGTTTGTATTTTTATTTACAGTA 300
P._delicatulus_site_28_______           ATTCACCTTCTATAATTTGAGCAATTGGGTTTGTATTTTTATTTACAGTA 300
P._kawadai_site_6___________            ATTCACCTTCTATAATTTGAGCAATTGGATTTGTATTTTTATTTACAGTA 300
P._kawadai_site_7_____________          ATTCACCTTCTATAATTTGAGCAATTGGATTTGTATTTTTATTTACAGTA 300
P._kawadai_site_8_____________          ATTCACCTTCTATAATTTGAGCAATTGGATTTGTATTTTTATTTACAGTA 300
P._delicatulus_site_26_______           ATTCACCTTCTATAATTTGAGCAATTGGATTTGTATTTTTATTTACAGTA 300
P._delicatulus_site_27_______           ATTCACCTTCTATAATTTGAGCAATTGGATTTGTATTTTTATTTACAGTA 300
P._takakuwai_site_11_________           ATTCACCTTCCATAATTTGAGCAATTGGGTTTGTATTTTTATTTACAGTA 300
P._oregonensis_sanmaeto_______          ATTCCCCATCAATAATTTGAGCAATTGGTTTTGTATTTTTATTTACAGTA 300
P._hongwonpyoi_birosa_________          ATTCACCCTCAATAATTTGAACAATTGGATTCGTATTCTTATTTACAGTT 300
P._hongwonpyoi_jengoge________          ATTCACCCTCAATAATTTGAACAATTGGATTCGTATTCTTATTTACAGTT 300
P._hongwonpyoi_deogyusan______          ACTCACCGTCAATAATTTGAGCAATCGGATTCGTATTCTTATTTACAGTT 300
P._hongwonpyoi_hangeryeong____          ACTCACCTTCAATAATTTGAGCAATTGGATTCGTATTCTTATTTACAGTT 300
P._hongwonpyoi_nogodan________          ACTCACCCTCAATAATTTGAGCAATTGGATTCGTATTCTTATTTACAGTT 300
P._hongwonpyoi_jeoksangsan____          ACTCACCTTCAATAGTTTGAGCAATTGGATTCGTATTCCTATTTACAGTA 300
                                        * ** ** ** *** * **  **** ** ** *****  **** *****

P._acuticollis_site_5________           GGGGGATTAACTGGAGTTGTATTAGCTAACTCCTCAATTGATATTATTCT 350
P._takakuwai_site_6_________            GGGGGATTAACTGGAGTTGTATTAGCTAACTCCTCAATTGATATTATTCT 350
P._takakuwai_site_7__________           GGGGGATTAACTGGAGTTGTATTAGCTAACTCCTCAATTGATATTATTCT 350
P._takakuwai_site_8__________           GGGGGATTAACTGGAGTTGTATTAGCTAACTCCTCAATTGATATTATTCT 350
P._albisomni_site_9__________           GGGGGATTAACTGGAGTTGTATTAGCTAACTCCTCAATTGATATTATTCT 350
P._albisomni_site_3__________           GGGGGATTAACTGGAGTTGTATTAGCTAACTCCTCAATTGATATTATTCT 350
P._albisomni_site_1__________           GGGGGATTAACTGGAGTAGTATTAGCTAACTCCTCAATTGATATTATTCT 350
P._albisomni_site_4__________           GGGGGATTAACTGGAGTAGTATTAGCTAACTCCTCAATTGATATTATTCT 350
P._takakuwai_site_10_________           GGAGGATTAACTGGAGTCGTATTAGCTAACTCCTCAATTGATATTATTCT 350
P._acuticollis_site_2________           GGAGGATTAACTGGAGTTGTATTAGCTAACTCCTCAATTGATATTATTCT 350
```

```
P._viridicuprus_site_15_______         GGGGGATTAACAGGTGTAGTATTAGCTAATTCCTCAATTGATATTATTCT 350
P._viridicuprus_site_16_______         GGGGGATTAACAGGTGTAGTATTAGCTAATTCCTCAATTGATATTATTCT 350
P._sue_site_22_____________            GGGGGATTAACAGGTGTGGTATTAGCTAATTCCTCAATTGATATTATTCT 350
P._viridicuprus_site_23_______         GGGGGATTAACAGGTGTAGTATTAGCTAATTCCTCAATTGATATTATTCT 350
P._viridicuprus_site_25_______         GGGGGATTAACAGGTGTAGTATTAGCTAATTCCTCAATTGATATTATTCT 350
P._viridicuprus_site_17_______         GGGGGATTAACAGGTGTAGTATTAGCTAATTCCTCAATTGATATTATTCT 350
P._takakuwai_site_14__________         GGGGGATTAACAGGAGTAGTTTTAGCTAATTCCTCAATCGATATTATCCT 350
P._akitaorum_site_13__________         GGGGGATTAACAGGAGTAGTTTTAGCTAATTCCTCAATTGATATTATCCT 350
P._takakuwai_site_12__________         GGGGGATTAACAGGAGTAGTTTTAGCTAATTCCTCAATTGATATTATCCT 350
P._takakuwai_site_18__________         GGGGGATTAACAGGAGTAGTTTTAGCTAATTCCTCAATTGATATTATCCT 350
P._sugitai_site_19____________         GGGGGATTAACAGGAGTAGTTTTAGCTAATTCCTCAATTGATATTATCCT 350
P._sugitai_site_21____________         GGGGGATTAACAGGAGTAGTTTTAGCTAATTCCTCAATTGATATTATCCT 350
P._urushiyamai_site_29________         GGGGGATTAACAGGAGTAGTTTTAGCTAATTCCTCAATTGATATTATCCT 350
P._urushiyamai_site_30________         GGGGGATTAACAGGAGTAGTTTTAGCTAATTCCTCAATTGATATTATCCT 350
P._urushiyamai_site_24________         GGGGGATTAACAGGAGTAGTTTTAGCTAATTCCTCAATTGATATTATCCT 350
P._kawadai_site_10__________           GGGGGATTAACAGGAGTAGTTTTAGCTAATTCCTCAATTGATATTATCCT 350
P._delicatulus_site_2_________         GGAGGATTAACAGGAGTAGTTTTAGCTAATTCCTCAATTGATATTATTCT 350
P._delicatulus_site_6_________         GGAGGATTAACAGGAGTAGTTTTAGCTAATTCCTCAATTGATATTATTCT 350
P._delicatulus_site_20________         GGGGGATTAACAGGAGTAGTTTTAGCTAATTCCTCAATTGATATTATTCT 350
P._delicatulus_site_25________         GGAGGATTAACAGGAGTAGTTTTAGCTAATTCCTCAATTGATATTATTCT 350
P._delicatulus_site_28________         GGAGGATTAACAGGAGTAGTTTTAGCTAATTCCTCAATTGATATTATTCT 350
P._kawadai_site_6___________           GGGGGATTAACAGGGGTAGTTTTAGCTAATTCCTCAATTGATATTATTCT 350
P._kawadai_site_7_____________         GGGGGATTAACAGGGGTAGTTTTAGCTAATTCCTCAATTGATATTATTCT 350
P._kawadai_site_8_____________         GGGGGATTAACAGGGGTAGTTTTAGCTAATTCCTCAATTGATATTATTCT 350
P._delicatulus_site_26________         GGGGGATTAACAGGAGTAGTTTTAGCTAATTCTTCAATTGATATTATCCT 350
P._delicatulus_site_27________         GGGGGATTAACAGGAGTAGTTTTAGCTAATTCCTCTATTGATATTATCCT 350
P._takakuwai_site_11__________         GGGGGATTAACAGGAGTAGTTTTAGCTAATTCCTCAATTGATATCATCCT 350
P._oregonensis_sanmaeto_______         GGAGGATTAACAGGAGTAGTCCTAGCAAATTCATCAATTGATATTATTTT 350
P._hongwonpyoi_birosa_________         GGGGGACTGACAGGAGTAGTATTAGCAAACTCCTCAATTGACATTATCCT 350
P._hongwonpyoi_jengoge________         GGGGGACTGACAGGGGTGGTATTAGCCAACTCCTCAATTGACATTATCCT 350
P._hongwonpyoi_deogyusan______         GGGGGATTGACAGGTGTAGTATTAGCAAACTCCTCAATTGACATTATCCT 350
P._hongwonpyoi_hangeryeong____         GGGGGACTGACGGGGGTCGTACTAGCAAATTCCTCAATTGACATTATCCT 350
P._hongwonpyoi_nogodan________         GGAGGTTTAACAGGGGTAGTATTAGCAAATTCCTCAATTGACATTATCCT 350
P._hongwonpyoi_jeoksangsan____         GGGGGGCTGACAGGGGTAGTATTAGCAAATTCCTCAATTGACATTATCCT 350
                                       ** **  * ** ** ** **  **** ** ** ** ** ** ** **  *
```

```
P._acuticollis_site_5_________       ACATGATACCTATTACGTAGTAGCCCACTTCCATTATGTTTTATCAATAG 400
P._takakuwai_site_6__________        ACATGATACCTATTACGTAGTAGCCCACTTCCATTATGTTTTATCAATAG 400
P._takakuwai_site_7___________       ACATGATACCTATTACGTAGTAGCCCACTTCCATTATGTTTTATCAATAG 400
P._takakuwai_site_8___________       ACATGATACCTACTACGTAGTAGCCCACTTCCATTATGTTTTATCAATAG 400
P._albisomni_site_9___________       ACATGATACCTATTACGTAGTAGCCCACTTCCATTATGTTTTATCAATAG 400
P._albisomni_site_3___________       ACATGATACCTATTACGTAGTAGCCCACTTCCATTATGTTTTATCAATAG 400
P._albisomni_site_1___________       ACATGATACCTATTACGTAGTAGCCCACTTCCATTATGTTTTATCAATAG 400
P._albisomni_site_4___________       ACATGATACCTATTACGTAGTAGCCCACTTCCATTATGTTTTATCAATAG 400
P._takakuwai_site_10__________       ACATGATACCTATTATGTAGTAGCTCACTTCCATTATGTTTTATCAATAG 400
P._acuticollis_site_2_________       GCATGATACCTATTATGTAGTAGCCCACTTCCATTATGTCTTATCAATAG 400
P._viridicuprus_site_15_______       CCACGATACTTATTACGTAGTAGCCCACTTCCATTATGTTTTATCAATAG 400
P._viridicuprus_site_16_______       CCACGATACTTATTACGTAGTAGCCCACTTCCATTATGTTTTATCAATAG 400
P._sue_site_22_____________          CCACGATACTTATTACGTAGTAGCCCACTTCCATTATGTTTTATCAATAG 400
P._viridicuprus_site_23_______       CCACGATACTTATTACGTAGTAGCCCACTTCCATTATGTTTTATCAATAG 400
P._viridicuprus_site_25_______       CCACGATACTTATTACGTAGTAGCCCACTTCCATTATGTTTTATCAATAG 400
P._viridicuprus_site_17_______       CCACGATACCTATTACGTAGTAGCCCACTTCCATTATGTTTTATCAATAG 400
P._takakuwai_site_14__________       TCATGACACTTACTATGTAGTAGCCCACTTCCATTATGTATTATCGATAG 400
P._akitaorum_site_13__________       TCATGACACTTACTATGTAGTAGCCCACTTCCATTATGTATTATCGATAG 400
P._takakuwai_site_12__________       TCATGACACTTACTATGTAGTAGCCCACTTCCATTATGTATTATCGATAG 400
P._takakuwai_site_18__________       TCATGACACTTACTACGTAGTAGCCCACTTCCATTATGTATTATCGATAG 400
P._sugitai_site_19____________       TCATGACACTTATTATGTAGTAGCCCACTTTCATTATGTATTATCGATAG 400
P._sugitai_site_21____________       TCATGACACTTACTATGTAGTAGCCCACTTCCATTATGTATTATCAATAG 400
P._urushiyamai_site_29________       TCATGACACTTACTATGTAGTAGCCCACTTCCATTATGTATTATCAATAG 400
P._urushiyamai_site_30________       TCATGACACTTACTATGTAGTAGCCCACTTCCATTATGTATTATCAATAG 400
P._urushiyamai_site_24________       TCATGACACTTACTATGTAGTAGCCCACTTCCATTATGTATTATCAATAG 400
P._kawadai_site_10__________         TCATGACACTTACTATGTAGTAGCCCACTTCCATTATGTACTATCAATAG 400
P._delicatulus_site_2_________       TCATGACACTTACTATGTAGTAGCCCACTTCCATTATGTATTATCAATAG 400
P._delicatulus_site_6_________       TCATGACACTTACTATGTAGTAGCCCACTTCCATTATGTATTATCAATAG 400
P._delicatulus_site_20________       TCATGACACTTATTATGTAGTAGCCCACTTCCATTATGTATTATCAATAG 400
P._delicatulus_site_25________       TCATGACACTTACTATGTAGTAGCCCACTTCCATTATGTGTTATCAATAG 400
P._delicatulus_site_28________       TCATGACACTTACTATGTAGTAGCCCACTTCCATTATGTGTTATCAATAG 400
P._kawadai_site_6___________         TCATGACACTTACTACGTAGTAGCCCACTTCCATTATGTATTATCAATAG 400
P._kawadai_site_7_____________       TCATGACACTTACTACGTAGTAGCCCACTTCCATTATGTATTATCAATAG 400
P._kawadai_site_8_____________       TCATGACACTTACTACGTAGTAGCCCACTTCCATTATGTATTATCAATAG 400
P._delicatulus_site_26________       TCATGACACTTACTATGTAGTAGCTCACTTCCATTATGTATTATCAATAG 400
P._delicatulus_site_27________       TCATGACACTTACTATGTAGTAGCTCACTTCCATTATGTGTTATCAATAG 400
```

```
P._takakuwai_site_11__________       TCATGATACTTACTATGTAGTAGCCCACTTCCATTATGTATTATCGATAG 400
P._oregonensis_sanmaeto_______       ACATGACACTTATTATGTAGTAGCTCACTTTCATTATGTTTTATCAATAG 400
P._hongwonpyoi_birosa_________       CCATGACACATATTATGTAGTGGCCCATTTTCACTACGTTTTATCAATAG 400
P._hongwonpyoi_jengoge________       CCATGACACATATTATGTAGTGGCCCATTTTCACTACGTTTTATCAATAG 400
P._hongwonpyoi_deogyusan______       CCACGATACATATTATGTAGTGGCCCATTTTCACTACGTTTTATCAATAG 400
P._hongwonpyoi_hangeryeong____       CCATGATACATATTATGTAGTGGCCCATTTTCATTACGTTTTATCAATAG 400
P._hongwonpyoi_nogodan________       TCATGACACATATTATGTAGTGGCCCATTTTCACTACGTTTTATCTATAG 400
P._hongwonpyoi_jeoksangsan____       CCATGACACATACTATGTAGTAGCCCATTTTCACTACGTTTTATCTATGG 400
                                      ** ** ** ** ** ***** ** ** ** ** ** **  **** ** *

P._acuticollis_site_5_________       GTGCTGTATTTGCTATCATAGCAGGATTTGTTCATTGATACCCTTTATTT 450
P._takakuwai_site_6__________        GTGCTGTATTTGCTATCATAGCAGGATTTGTTCATTGATACCCTTTATTT 450
P._takakuwai_site_7___________       GTGCTGTATTTGCTATCATAGCAGGATTTGTTCATTGATACCCTTTATTT 450
P._takakuwai_site_8___________       GTGCTGTATTTGCTATCATAGCAGGATTTGTTCATTGATACCCTTTATTT 450
P._albisomni_site_9___________       GTGCTGTATTTGCTATCATAGCAGGATTTGTTCATTGATACCCTTTATTT 450
P._albisomni_site_3___________       GTGCTGTATTTGCTATCATAGCAGGATTTGTTCATTGATACCCTTTATTT 450
P._albisomni_site_1___________       GTGCTGTATTTGCTATCATAGCAGGATTTGTTCATTGATACCCTTTATTT 450
P._albisomni_site_4___________       GTGCTGTATTTGCTATTATAGCAGGATTTGTTCATTGATACCCTTTATTT 450
P._takakuwai_site_10__________       GTGCTGTATTTGCTATCATAGCAGGATTTGTTCATTGATACCCTTTATTT 450
P._acuticollis_site_2_________       GCGCTGTATTTGCTATTATAGCAGGATTTGTTCATTGATACCCTTTATTT 450
P._viridicuprus_site_15_______       GAGCCGTATTTGCTATTATAGCAGGATTTGTCCATTGATACCCATTATTT 450
P._viridicuprus_site_16_______       GAGCCGTATTTGCTATTATAGCAGGATTTGTCCATTGATACCCATTATTT 450
P._sue_site_22_____________          GAGCCGTATTTGCTATTATAGCAGGATTTGTCCATTGATATCCATTATTT 450
P._viridicuprus_site_23_______       GAGCTGTATTTGCTATTATAGCAGGATTTGTCCATTGATACCCATTATTT 450
P._viridicuprus_site_25_______       GAGCTGTATTTGCTATTATAGCAGGATTTGTCCATTGATACCCATTATTT 450
P._viridicuprus_site_17_______       GAGCTGTATTTGCTATTATAGCGGGATTTGTCCATTGATACCCATTATTT 450
P._takakuwai_site_14__________       GAGCTGTGTTCGCTATTATAGCGGGATTTGTCCATTGATACCCTTTATTT 450
P._akitaorum_site_13__________       GAGCTGTGTTCGCTATTATAGCGGGATTTGTCCATTGATACCCTTTATTT 450
P._takakuwai_site_12__________       GAGCTGTGTTCGCTATTATAGCAGGATTTGTCCATTGATACCCTTTATTT 450
P._takakuwai_site_18__________       GAGCTGTGTTCGCTATTATAGCAGGATTTGTCCATTGATACCCTTTATTT 450
P._sugitai_site_19____________       GAGCTGTATTCGCTATTATAGCAGGATTTGTCCATTGATACCCTTTATTT 450
P._sugitai_site_21____________       GAGCTGTATTCGCTATTATAGCAGGATTTGTCCATTGATACCCTTTATTT 450
P._urushiyamai_site_29________       GAGCCGTGTTCGCTATTATAGCAGGATTTGTCCATTGATACCCTTTATTT 450
P._urushiyamai_site_30________       GAGCCGTGTTCGCTATTATAGCAGGATTTGTCCATTGATACCCTTTATTT 450
P._urushiyamai_site_24________       GAGCCGTGTTCGCTATTATAGCAGGATTTGTCCATTGATACCCTTTATTT 450
P._kawadai_site_10__________         GAGCTGTGTTCGCTATTATAGCAGGATTTGTCCATTGGTACCCTTTATTC 450
```

```
P._delicatulus_site_2________        GGGCTGTGTTCGCTATTATAGCAGGATTTGTCCATTGATACCCTCTATTT 450
P._delicatulus_site_6________        GGGCTGTATTCGCTATTATAGCAGGATTTGTACATTGATACCCTCTATTT 450
P._delicatulus_site_20_______        GGGCTGTGTTTGCTATTATAGCAGGATTTGTCCATTGATACCCTCTATTT 450
P._delicatulus_site_25_______        GGGCTGTGTTCGCTATTATAGCAGGATTTGTCCATTGATACCCTCTATTT 450
P._delicatulus_site_28_______        GGGCTGTGTTCGCTATTATAGCAGGATTTGTCCATTGATACCCTCTATTT 450
P._kawadai_site_6__________          GGGCTGTATTCGCTATTATAGCAGGATTTGTCCATTGATACCCTCTATTT 450
P._kawadai_site_7____________        GGGCTGTATTCGCTATTATAGCAGGATTTGTCCATTGATACCCTCTATTT 450
P._kawadai_site_8____________        GGGCTGTATTCGCTATTATAGCAGGATTTGTCCATTGATATCCTCTATTT 450
P._delicatulus_site_26_______        GGGCTGTGTTCGCTATTATAGCAGGATTTGTCCATTGATACCCTTTATTT 450
P._delicatulus_site_27_______        GGGCTGTGTTCGCTATTATAGCAGGATTTGTCCATTGATACCCTTTATTT 450
P._takakuwai_site_11_________        GGGCTGTGTTCGCTATTATAGCAGGATTTGTCCATTGATATCCTTTATTT 450
P._oregonensis_sanmaeto______        GGGCTGTATTTGCAATTATAGCTGGATTTGTTCATTGATACCCCTTATTT 450
P._hongwonpyoi_birosa________        GGGCAGTATTTGCCATCATGGCAGGATTTGTACATTGATACCCTTTATTT 450
P._hongwonpyoi_jengoge_______        GGGCAGTATTTGCCATCATGGCAGGATTTGTACATTGATACCCTTTATTT 450
P._hongwonpyoi_deogyusan_____        GAGCGGTGTTTGCCATCATAGCAGGATTTGTACATTGATACCCTTTATTT 450
P._hongwonpyoi_hangeryeong___        GAGCAGTATTCGCCATCATAGCGGGATTTGTACATTGATACCCTTTATTT 450
P._hongwonpyoi_nogodan_______        GAGCAGTATTTGCCATCATAGCAGGATTTGTACATTGATATCCTTTATTT 450
P._hongwonpyoi_jeoksangsan___        GGGCGGTATTTGCCATCATAGCAGGATTTGTACATTGATACCCTTTATTT 450
                                     * ** ** ** ** ** ** ** ******** ***** ** **  ****

P._acuticollis_site_5________        ACAGGCCTTTCCTTAAATAATAAATACTTAAAAATTCAATTTCTAGTAAT 500
P._takakuwai_site_6_________         ACAGGCCTTTCCTTAAATAATAAATACTTAAAAATTCAATTTCTAGTAAT 500
P._takakuwai_site_7__________        ACAGGCCTTTCCTTAAATAATAAATACTTAAAAATTCAATTTCTAGTAAT 500
P._takakuwai_site_8__________        ACAGGCCTTTCCTTAAATAATAAATACTTAAAAATTCAATTTCTAGTAAT 500
P._albisomni_site_9__________        ACAGGACTTTCCTTAAATAATAAATACTTAAAAATTCAATTTATAGTAAT 500
P._albisomni_site_3__________        ACAGGACTTTCCTTAAATAATAAATACTTAAAAATTCAATTTCTAGTAAT 500
P._albisomni_site_1__________        ACAGGACTTTCCTTAAATAATAAATACTTAAAAATTCAATTTATAGTAAT 500
P._albisomni_site_4__________        ACAGGCCTTTCCTTAAATAATAAGTACTTAAAAATTCAATTTCTAGTAAT 500
P._takakuwai_site_10_________        ACAGGGCTTTCCTTAAATAATAAATACTTAAAAATTCAATTTCTAGTAAT 500
P._acuticollis_site_2________        ACAGGCCTTTCCTTAAATAATAAGTACTTAAAAATTCAATTTCTAGTTAT 500
P._viridicuprus_site_15______        ACAGGTCTTTCCTTAAATAATAAATATCTAAAAATTCAGTTCCTAGTTAT 500
P._viridicuprus_site_16______        ACAGGTCTCTCCTTAAATAATAAATACCTAAAAATTCAGTTCCTAGTTAT 500
P._sue_site_22____________           ACAGGTCTTTCCTTAAATAATAAATATCTAAAAATTCAGTTCCTAGTTAT 500
P._viridicuprus_site_23______        ACAGGTCTTTCCTTAAATAATAAATATCTAAAAATTCAGTTCCTAGTTAT 500
P._viridicuprus_site_25______        ACAGGTCTTTCCTTAAATAATAAATATCTAAAAATTCAGTTCCTAGTTAT 500
P._viridicuprus_site_17______        ACAGGTCTTTCCTTAAATAATAAATATCTAAAAATTCAGTTCCTAGTTAT 500
```

```
P._takakuwai_site_14__________      ACAGGACTATCCTTAAACAATAAATACTTAAAAATTCAATTTCTAGTGAT 500
P._akitaorum_site_13__________      ACAGGACTATCCTTAAACAATAAATACTTAAAAATTCAATTTCTAGTAAT 500
P._takakuwai_site_12__________      ACAGGACTATCCTTAAACAATAAATACTTAAAAGTTCAATTTCTAGTAAT 500
P._takakuwai_site_18__________      ACAGGGCTATCCTTAAACAATAAATACTTAAAAATTCAATTTCTAGTAAT 500
P._sugitai_site_19____________      ACAGGGCTATCCTTAAACAATAAATACTTAAAAATTCAATTTCTAGTAAT 500
P._sugitai_site_21____________      ACAGGGCTATCCTTAAACAATAAATACTTAAAAATTCAATTTCTAGTAAT 500
P._urushiyamai_site_29________      ACAGGATTATCATTAAACAATAAATACTTAAAGATTCAATTTCTAGTAAT 500
P._urushiyamai_site_30________      ACAGGATTATCATTAAACAATAAATACTTAAAGATTCAATTTCTAGTAAT 500
P._urushiyamai_site_24________      ACAGGATTATCATTAAACAATAAATACTTAAAGATTCAATTTCTAGTAAT 500
P._kawadai_site_10__________        ACAGGACTATCCTTAAACAATAAATACTTAAAAATTCAATTCCTAGTAAT 500
P._delicatulus_site_2_________      ACAGGGTTATCCTTAAACAATAAATACTTAAAAATTCAATTTCTAGTAAT 500
P._delicatulus_site_6_________      ACAGGGTTATCCTTAAACAATAAATACTTAAAAATTCAATTTCTAGTAAT 500
P._delicatulus_site_20________      ACAGGGCTATCCTTAAACAATAAATACTTAAAAATTCAATTTCTAGTAAT 500
P._delicatulus_site_25________      ACAGGGCTATCCTTAAACAATAAATACTTAAAAATTCAATTTCTAGTAAT 500
P._delicatulus_site_28________      ACAGGACTATCCTTAAACAATAAATACTTAAAAATTCAATTTCTAGTAAT 500
P._kawadai_site_6___________        ACAGGATTATCCTTAAACAATAAATACTTAAAAATTCAATTTCTAGTAAT 500
P._kawadai_site_7_____________      ACAGGATTATCCTTAAACAATAAATACTTAAAAATTCAATTTCTAGTAAT 500
P._kawadai_site_8_____________      ACAGGATTATCCTTAAACAACAAATACTTAAAAATTCAATTTCTAGTAAT 500
P._delicatulus_site_26________      ACAGGATTATCCTTAAACAATAAATACTTAAAAATTCAATTTCTAGTAAT 500
P._delicatulus_site_27________      ACAGGATTATCCTTAAACAATAAATACTTAAAAATTCAATTTCTAGTAAT 500
P._takakuwai_site_11__________      ACAGGGCTATCCTTAAACAATAAATACTTAAAAATTCAATTTCTAGTTAT 500
P._oregonensis_sanmaeto_______      ACAGGACTATCTCTTAATAATAAATACCTAAAAATTCAATTTTTAGTTAT 500
P._hongwonpyoi_birosa_________      ACAGGACTATCTTTAAATAACAAATATTTAAAAATTCAATTTCTAGTTAT 500
P._hongwonpyoi_jengoge________      ACAGGACTATCTTTAAATAACAAATATTTAAAAATTCAGTTTCTAGTTAT 500
P._hongwonpyoi_deogyusan______      ACAGGATTATCTTTAAATAACAAATATTTAAAAATTCAATTTCTAGTCAT 500
P._hongwonpyoi_hangeryeong____      ACAGGGTTATCTTTAAATAACAAATATTTAAAAATTCAATTTCTAGTTAT 500
P._hongwonpyoi_nogodan________      ACAGGACTATTTTTAAATAGTAAGTATTTAAAAATTCAATTTCTAGTCAT 500
P._hongwonpyoi_jeoksangsan____      ACAGGGCTATCTTTAAATAACAAATATTTAAAAATTCAATTTCTAGTCAT 500
                                    *****  * *   * ** *  ** **  ****  **** **  **** **

P._acuticollis_site_5_________      ATTCATTGGGGTAAATATAACTTTTTTCCCACAACATTTTCTGGGATTAA 550
P._takakuwai_site_6__________       ATTCATTGGGGTAAATATAACTTTTTTCCCACAACATTTTCTGGGATTAA 550
P._takakuwai_site_7___________      ATTCATTGGAGTAAATATAACTTTTTTCCCACAACATTTTCTAGGATTAA 550
P._takakuwai_site_8___________      ATTCATTGGGGTAAATATAACTTTTTTCCCACAACATTTTCTAGGATTAA 550
P._albisomni_site_9___________      ATTCATTGGAGTAAATATAACTTTTTTCCCGCAACATTTTCTAGGATTAA 550
P._albisomni_site_3___________      ATTCATTGGGGTAAATATAACCTTTTTCCCGCAACATTTTCTGGGATTAA 550
```

```
P._albisomni_site_1___________      ATTCATTGGGGTAAATATAACTTTTTTCCCTCAACATTTTCTAGGATTAA 550
P._albisomni_site_4___________      ATTCATTGGAGTAAATATAACTTTTTTCCCACAACATTTTCTGGGATTAA 550
P._takakuwai_site_10__________      GTTCATTGGGGTAAATATAACTTTTTTCCCACAACATTTTCTGGGATTAA 550
P._acuticollis_site_2_________      ATTCATTGGGGTGAATATAACTTTTTTCCCACAACATTTTCTGGGATTAA 550
P._viridicuprus_site_15_______      ATTTATTGGGGTAAATATAACATTTTTCCCTCAACATTTTCTTGGATTAA 550
P._viridicuprus_site_16_______      ATTTATTGGGGTAAATATAACATTTTTCCCTCAACATTTTCTTGGGTTAA 550
P._sue_site_22_____________         ATTTATTGGGGTAAATATAACATTTTTCCCTCAACATTTTCTTGGATTAA 550
P._viridicuprus_site_23_______      ATTTATTGGGGTAAATATAACATTTTTCCCCCAACATTTTCTTGGATTAA 550
P._viridicuprus_site_25_______      ATTTATTGGGGTAAATATAACATTTTTCCCCCAACATTTTCTTGGGTTAA 550
P._viridicuprus_site_17_______      ATTTATTGGGGTAAATATAACATTTTTCCCTCAACATTTTCTTGGATTAA 550
P._takakuwai_site_14__________      ATTTATTGGAGTAAACATAACATTTTTCCCCCAACATTTTCTTGGATTAA 550
P._akitaorum_site_13__________      ATTTATTGGAGTAAACATAACATTTTTCCCTCAACATTTTCTTGGATTAA 550
P._takakuwai_site_12__________      ATTTATTGGAGTAAACATAACATTTTTCCCTCAACATTTTCTTGGATTAA 550
P._takakuwai_site_18__________      ATTTATTGGAGTAAACATAACATTTTTCCCTCAACATTTCCTTGGATTAA 550
P._sugitai_site_19____________      ATTTATTGGAGTAAACATAACATTTTTCCCTCAACATTTCCTTGGATTAA 550
P._sugitai_site_21____________      ATTTATTGGAGTAAACATAACATTTTTCCCTCAACATTTCCTTGGATTAA 550
P._urushiyamai_site_29________      ATTTATTGGAGTAAATATAACATTTTTCCCTCAACATTTCCTTGGGTTAA 550
P._urushiyamai_site_30________      ATTTATTGGAGTAAATATAACATTTTTCCCTCAACATTTCCTTGGATTAA 550
P._urushiyamai_site_24________      ATTTATTGGAGTAAATATAACATTTTTCCCTCAACATTTCCTTGGATTAA 550
P._kawadai_site_10__________        ATTTATTGGGGTAAACTTAACATTTTTCCCTCAACATTTCCTTGGATTAA 550
P._delicatulus_site_2_________      ATTTATTGGAGTAAACATAACATTTTTCCCTCAACACTTCCTTGGATTAA 550
P._delicatulus_site_6_________      ATTTATTGGAGTAAACATAACATTTTTCCCCCAACACTTCCTTGGATTAA 550
P._delicatulus_site_20________      ATTTATTGGAGTAAACATAACATTTTTCCCTCAACACTTCCTTGGATTAA 550
P._delicatulus_site_25________      ATTTATTGGAGTAAACATAACATTTTTCCCTCAACACTTCCTTGGATTAA 550
P._delicatulus_site_28________      ATTTATTGGAGTAAACATAACATTTTTCCCTCAACACTTCCTTGGTTTAA 550
P._kawadai_site_6___________        ATTTATTGGAGTAAACATAACATTTTTCCCTCAACACTTCCTTGGATTAA 550
P._kawadai_site_7_____________      ATTTATTGGAGTAAACATAACATTTTTCCCTCAACACTTCCTTGGATTAA 550
P._kawadai_site_8_____________      ATTTATTGGAGTAAACATAACATTTTTCCCCCAACACTTCCTTGGATTAA 550
P._delicatulus_site_26________      ATTTATTGGAGTAAACATAACATTTTTCCCTCAACATTTCCTTGGATTAA 550
P._delicatulus_site_27________      ATTTATTGGAGTAAACATAACATTTTTCCCTCAACATTTCCTTGGATTAA 550
P._takakuwai_site_11__________      ATTTATTGGGGTAAATATAACATTTTTTCCTCAACATTTCCTTGGATTAA 550
P._oregonensis_sanmaeto_______      ATTTATTGGAGTAAACATAACATTTTTCCCTCAACATTTTCTAGGGCTAA 550
P._hongwonpyoi_birosa_________      ATTTATTGGAGTAAACATAACATTCTTCCCACAACATTTCCTGGGATTAA 550
P._hongwonpyoi_jengoge________      ATTTATTGGAGTAAACATAACATTCTTCCCACAACATTTCCTGGGATTAA 550
P._hongwonpyoi_deogyusan______      ATTTATTGGAGTAAATATAACATTCTTTCCGCAGCATTTCCTGGGGTTAA 550
P._hongwonpyoi_hangeryeong____      ATTTATTGGAGTAAACATAACGTTCTTTCCACAACATTTCCTGGGGTTAA 550
```

```
P._hongwonpyoi_nogodan________        ATTTATTGGAGTAAACATAACGTTCTTTCCACAACATTTCCTGGGATTAA 550
P._hongwonpyoi_jeoksangsan____        ATTTATTGGAGTAAATATAACATTCTTTCCACAACATTTCCTGGGGTTAA 550
                                       ** ***** ** **  **** ** ** ** ** ** ** ** **  ***

P._acuticollis_site_5_________        GGGGTATACCTCGACGTTATTCTGATTATCCGGATGCTTACACGGCTTGA 600
P._takakuwai_site_6__________         GGGGTATACCTCGACGTTATTCTGATTATCCGGATGCTTACACGGCTTGA 600
P._takakuwai_site_7___________        GGGGTATACCTCGACGTTATTCTGATTATCCGGATGCTTACACGGCTTGA 600
P._takakuwai_site_8___________        GGGGTATACCTCGACGTTATTCTGATTATCCGGATGCTTACACGGCTTGA 600
P._albisomni_site_9___________        GGGGCATACCTCGACGTTATTCTGATTATCCGGATGCTTACACGGCTTGA 600
P._albisomni_site_3___________        GGGGTATACCTCGACGTTATTCTGATTATCCGGATGCTTACACGGCTTGA 600
P._albisomni_site_1___________        GGGGCATACCTCGACGTTATTCTGATTATCCGGATGCTTACACGGCTTGA 600
P._albisomni_site_4___________        GAGGTATACCTCGACGTTATTCTGATTATCCGGATGCTTATACGGCTTGA 600
P._takakuwai_site_10__________        GAGGTATGCCTCGACGTTATTCTGATTATCCAGATGCTTACACAGCTTGA 600
P._acuticollis_site_2_________        GGGGTATGCCTCGACGTTATTCTGATTATCCTGAAGCTTACACGGCTTGA 600
P._viridicuprus_site_15_______        GAGGTATACCTCGACGTTATTCTGACTACCCAGATGCTTACACAGCTTGA 600
P._viridicuprus_site_16_______        GAGGTATACCTCGACGTTATTCTGACTATCCAGATGCTTACACAGCTTGA 600
P._sue_site_22_____________           GAGGTATACCTCGACGTTATTCTGACTATCCAGATGCTTACACAGCTTGA 600
P._viridicuprus_site_23_______        GAGGTATACCTCGACGTTATTCTGACTATCCAGATGCTTACACAGCTTGA 600
P._viridicuprus_site_25_______        GAGGTATACCTCGACGTTATTCTGACTATCCAGATGCTTACACAGCTTGA 600
P._viridicuprus_site_17_______        GAGGTATACCTCGACGTTATTCTGACTATCCAGATGCTTATACAGCTTGA 600
P._takakuwai_site_14__________        GAGGTATACCTCGACGATATTCTGATTATCCAGACGCCTATACAGCTTGA 600
P._akitaorum_site_13__________        GAGGTATACCTCGACGATATTCTGATTATCCAGACGCCTATACAGCTTGA 600
P._takakuwai_site_12__________        GAGGTATACCTCGACGATATTCTGATTATCCAGACGCCTATACAGCTTGA 600
P._takakuwai_site_18__________        GAGGTATACCTCGACGATATTCTGATTATCCGGACGCTTACACAGCTTGA 600
P._sugitai_site_19____________        GAGGTATACCTCGACGATATTCTGATTATCCAGACGCCTATACAGCTTGA 600
P._sugitai_site_21____________        GGGGTATACCTCGACGATATTCCGATTATCCAGACGCCTATACAGCTTGA 600
P._urushiyamai_site_29________        GAGGTATACCTCGACGATATTCTGATTATCCAGATGCCTATACAGCTTGA 600
P._urushiyamai_site_30________        GAGGTATACCTCGACGGTATTCTGATTATCCAGATGCCTATACAGCTTGA 600
P._urushiyamai_site_24________        GAGGTATACCTCGACGGTATTCTGATTATCCAGATGCCTATACAGCTTGA 600
P._kawadai_site_10__________          GAGGTATACCTCGACGATATTCTGATTATCCAGATGCCTATACAGCTTGA 600
P._delicatulus_site_2_________        GAGGTATACCTCGACGATATTCTGATTATCCAGACGCCTATACAGCTTGA 600
P._delicatulus_site_6_________        GAGGCATACCTCGACGATATTCTGATTATCCAGATGCCTATACAGCTTGA 600
P._delicatulus_site_20________        GAGGTATACCTCGACGATATTCTGATTACCCAGACGCCTATACAGCTTGA 600
P._delicatulus_site_25________        GAGGTATACCTCGACGATATTCTGATTATCCAGACGCCTACACAGCTTGA 600
P._delicatulus_site_28________        GAGGTATACCTCGACGATATTCTGATTATCCAGACGCCTACACAGCTTGA 600
P._kawadai_site_6___________          GAGGAATACCTCGACGATATTCTGATTATCCAGACGCCTATACAGCTTGA 600
```

```
P._kawadai_site_7_____________        GAGGAATACCTCGACGATATTCTGATTATCCAGACGCCTATACAGCTTGA 600
P._kawadai_site_8_____________        GGGGAATACCTCGACGATATTCTGATTATCCGGACGCCTATACAGCTTGA 600
P._delicatulus_site_26________        GAGGTATACCTCGACGATATTCAGATTACCCAGACGCCTATACAGCTTGA 600
P._delicatulus_site_27________        GAGGCATACCTCGACGATATTCAGATTACCCAGACGCCTATACAGCTTGA 600
P._takakuwai_site_11__________        GAGGTATACCTCGACGATATTCTGATTATCCAGACGCCTATACAGCTTGA 600
P._oregonensis_sanmaeto_______        GAGGAATACCTCGACGTTATTCTGATTATCCAGATGCTTATACAGCTTGA 600
P._hongwonpyoi_birosa_________        GAGGTATACCTCGACGTTACTCTGATTACCCAGATGCCTACACAGCTTGA 600
P._hongwonpyoi_jengoge________        GAGGTATACCTCGACGTTACTCTGATTACCCAGATGCCTACACAGCTTGA 600
P._hongwonpyoi_deogyusan______        GAGGAATACCTCGACGTTACTCTGATTACCCAGATGCCTACACCGCTTGA 600
P._hongwonpyoi_hangeryeong____        GAGGAATGCCCCGACGTTACTCTGATTACCCGGATGCCTACACAGCCTGA 600
P._hongwonpyoi_nogodan________        GAGGGATACCTCGACGTTACTCTGATTATCCAGATGCCTACACAGCCTGA 600
P._hongwonpyoi_jeoksangsan____        GAGGCATACCTCGACGTTATTCTGATTACCCAGATGCCTACACAGCTTGA 600
                                      * ** ** ** ***** ** ** ** ** ** ** ** ** ** ** ***

P._acuticollis_site_5_________        AACATTATTTCATCTATTGGATCTTTAATCTCATTTGTAAGAATTTTAAT 650
P._takakuwai_site_6_________          AACATTATTTCATCTATTGGATCTTTAATCTCATTTGTAAGAATCTTAAT 650
P._takakuwai_site_7___________        AACATTATTTCATCTATTGGATCTTTAATCTCATTTGTAAGAATTTTAAT 650
P._takakuwai_site_8___________        AACATTATTTCATCTATTGGATCTTTAATCTCATTTGTAAGAATTTTAAT 650
P._albisomni_site_9___________        AACATTATTTCATCTATTGGATCTTTAATCTCATTTGTAAGAATTTTAAT 650
P._albisomni_site_3___________        AACATTATTTCATCTATTGGATCTTTAATCTCATTTGTAAGAATTTTAAT 650
P._albisomni_site_1___________        AACATTATTTCATCTATTGGATCTTTAATCTCATTTGTAAGAATTTTAAT 650
P._albisomni_site_4___________        AACATTATTTCATCTATTGGATCTTTAATCTCATTTGTAAGAATTTTAAT 650
P._takakuwai_site_10__________        AACATTATTTCATCTATTGGATCTTTAATTTCATTTGTAAGAATTTTAAT 650
P._acuticollis_site_2_________        AACATTATTTCATCTATTGGATCTTTAATCTCATTTGTAAGAATTTTAAT 650
P._viridicuprus_site_15_______        AATATTATTTCATCTATTGGATCCTTGATTTCATTTGTAAGAATTTTAAT 650
P._viridicuprus_site_16_______        AATATTATTTCATCTATTGGATCTTTGATTTCATTTGTAAGAATTTTAAT 650
P._sue_site_22_____________           AATATTATTTCATCTATTGGATCTTTAATTTCATTCGTAAGAATTTTGAT 650
P._viridicuprus_site_23_______        AATGTTATTTCATCTATTGGATCTTTAATTTCATTCGTAAGAATTTTAAT 650
P._viridicuprus_site_25_______        AATGTTATTTCATCTATTGGATCTTTAATTTCATTCGTAAGAATTTTAAT 650
P._viridicuprus_site_17_______        AATATTATTTCATCTATTGGATCTTTAATTTCATTCGTAAGAATTTTAAT 650
P._takakuwai_site_14__________        AATATTATTTCATCTATTGGATCTCTAATTTCATTCGTAAGAATTTTAAT 650
P._akitaorum_site_13__________        AATATTATTTCATCTATTGGATCTCTAATTTCATTCGTAAGAATTTTAAT 650
P._takakuwai_site_12__________        AATATTATTTCATCTATTGGATCTCTAATTTCATTCGTAAGAATTTTAAT 650
P._takakuwai_site_18__________        AATATTATTTCATCTATTGGATCTCTAATTTCATTCGTAAGAATTTTAAT 650
P._sugitai_site_19____________        AATATTATTTCATCTATTGGATCTCTAATTTCATTCGTAAGAATTTTAAT 650
P._sugitai_site_21____________        AATATTATTTCATCTATTGGATCTCTAATTTCATTCGTAAGAATTTTAAT 650
```

```
P._urushiyamai_site_29________       AATATTATTTCATCTATTGGATCCCTAATTTCATTCGTAAGAATTTTAAT 650
P._urushiyamai_site_30________       AATATTATTTCATCTATTGGATCCCTAATTTCATTCGTAAGAATTTTAAT 650
P._urushiyamai_site_24________       AATATTATTTCATCTATTGGATCTCTAATTTCATTTGTAAGAATTTTAAT 650
P._kawadai_site_10__________         AATATTATCTCATCTATTGGGTCTTTAATTTCATTCGTAAGAATTTTAAT 650
P._delicatulus_site_2_________       AATATTATTTCATCTATTGGATCTCTAATCTCATTCGTAAGAATTTTAAT 650
P._delicatulus_site_6_________       AATATTATTTCATCTATTGGATCTCTAATCTCATTCGTAAGAATTTTAAT 650
P._delicatulus_site_20________       AATATTATTTCATCTATTGGATCTCTAATCTCATTCGTAAGAATTTTAAT 650
P._delicatulus_site_25________       AATATTATTTCATCTATTGGATCTCTAATCTCATTCGTAAGAATCTTAAT 650
P._delicatulus_site_28________       AATATTATTTCATCTATTGGATCTCTAATCTCATTCGTAAGAATCTTAAT 650
P._kawadai_site_6___________         AATGTTATTTCATCTATTGGATCTCTAATCTCATTCGTAAGAATTTTAAT 650
P._kawadai_site_7_____________       AATGTTATTTCATCTATTGGATCTCTAATCTCATTCGTAAGAATTTTAAT 650
P._kawadai_site_8_____________       AATGTTATTTCATCTATTGGATCTCTAATCTCATTCGTAAGAATTTTAAT 650
P._delicatulus_site_26________       AATATCATTTCATCTATTGGATCTCTAATTTCATTCGTAAGAATTTTAAT 650
P._delicatulus_site_27________       AATATCATTTCATCTATTGGATCTCTAATTTCATTCGTAAGAATTTTAAT 650
P._takakuwai_site_11__________       AATATCATTTCATCTATTGGATCTCTAATTTCGTTCGTAAGAATTTTAAT 650
P._oregonensis_sanmaeto_______       AATATTATTTCATCAATCGGATCGTTAATTTCATTTGTAAGAATTCTAAT 650
P._hongwonpyoi_birosa_________       AATATTATTTCATCAATCGGGTCTTTAATTTCATTCGTAAGAATTTTAAT 650
P._hongwonpyoi_jengoge________       AATATTATTTCATCAATCGGGTCTTTAATTTCATTCGTAAGAATTTTAAT 650
P._hongwonpyoi_deogyusan______       AATATTATTTCATCAATTGGGTCTTTAATTTCATTTGTAAGAATTTTAAT 650
P._hongwonpyoi_hangeryeong____       AATATTATTTCATCAATTGGGTCTTTAATTTCATTCGTAAGAATTTTAAT 650
P._hongwonpyoi_nogodan________       AATATTATTTCATCAATTGGGTCTTTAATTTCATTCGTAAGAATTTTAAT 650
P._hongwonpyoi_jeoksangsan____       AATATTATTTCATCAATTGGGTCTTTAATTTCATTCGTAAGAATTTTAAT 650
                                     **  * ** ***** ** ** **  * ** ** ** ********  * **

P._acuticollis_site_5_________       ATTTATATTTATTATTTGAGAAAGATTTTCCTCGATTCGAAAAAGAATCT 700
P._takakuwai_site_6__________        ATTTATATTTATTATTTGAGAAAGATTTTCCTCGATTCGAAAAAGAATCT 700
P._takakuwai_site_7___________       ATTTATATTTATTATTTGAGAAAGATTTTCCTCGATTCGAAAAAGAATCT 700
P._takakuwai_site_8___________       ATTTATATTTATTATTTGAGAAAGATTCTCCTCGATTCGAAAAAGAATCT 700
P._albisomni_site_9___________       ATTTATATTTATTATTTGAGAAAGATTTTCCTCGATTCGAAAAAGAATCT 700
P._albisomni_site_3___________       ATTTATATTTATTATTTGAGAAAGATTTTCCTCGATTCGAAAAAGAATCT 700
P._albisomni_site_1___________       ACTTATATTTATTATTTGAGAAAGATTTTCCTCGATTCGAAAAAGAATCT 700
P._albisomni_site_4___________       ATTTATATTTATTATTTGAGAAAGATTTTCCTCGATTCGAAAAAGAATCT 700
P._takakuwai_site_10__________       ATTTATATTTATTATTTGAGAAAGATTTTCCTCGATTCGAAAAAGAATCT 700
P._acuticollis_site_2_________       ATTTATATTTATTATCTGAGAAAGATTTTCCTCAATTCGAAAAAGAATTT 700
P._viridicuprus_site_15_______       ATTTATATTCATTATTTGAGAAAGATTTTCCTCAATTCGAAAAAGAATTT 700
P._viridicuprus_site_16_______       ATTTATATTCATTATTTGAGAAAGATTTTCCTCAATTCGAAAAAGAATTT 700
```

```
P._sue_site_22______________         ATTTATATTCATTATTTGAGAAAGATTTTCCTCAATTCGAAAAAGAATTT 700
P._viridicuprus_site_23_______       ATTTATATTCATTATTTGAGAAAGATTTTCCTCAATTCGAAAAAGAATTT 700
P._viridicuprus_site_25_______       ATTTATATTCATTATTTGAGAAAGATTTTCCTCAATTCGAAAAAGAATTT 700
P._viridicuprus_site_17_______       ATTTATATTCATTATTTGAGAAAGATTTTCCTCAATTCGAAAAAGAATTT 700
P._takakuwai_site_14__________       ATTTATATTTATTATTTGAGAAAGATTCTCTTCAATTCGAAAAAGAATTT 700
P._akitaorum_site_13__________       ATTTATATTTATTATTTGAGAAAGATTCTCTTCAATTCGAAAAAGAATTT 700
P._takakuwai_site_12__________       ATTTATATTTATTATTTGAGAAAGATTCTCTTCAATTCGAAAAAGAATTT 700
P._takakuwai_site_18__________       ATTTATATTTATTATTTGAGAAAGATTCTCTTCAATTCGAAAAAGAATTT 700
P._sugitai_site_19____________       ATTTATATTTATTATTTGAGAAAGATTCTCTTCAATTCGAAAAAGAATTT 700
P._sugitai_site_21____________       ATTTATATTTATTATTTGAGAAAGGTTCTCTTCAATTCGAAAAAGAATTT 700
P._urushiyamai_site_29________       ATTTATATTTATTATTTGAGAAAGATTCTCCTCAATTCGAAAAAGAATTT 700
P._urushiyamai_site_30________       ATTTATATTTATTATTTGAGAAAGATTCTCCTCAATTCGAAAAAGAATTT 700
P._urushiyamai_site_24________       ATTTATATTTATTATTTGAGAAAGATTCTCCTCAATTCGAAAAAGAATTT 700
P._kawadai_site_10__________         ATTTATATTTATTATTTGAGAAAGATTCTCTTCAATTCGAAAAAGAATTT 700
P._delicatulus_site_2_________       ATTTGTATTTATTATTTGAGAAAGATTCTCTTCAATTCGAAAAAGAATTT 700
P._delicatulus_site_6_________       ATTTGTATTTATTATTTGAGAAAGATTCTCTTCAATTCGAAAAAGAATTT 700
P._delicatulus_site_20________       ATTTGTATTTATTATTTGAGAAAGATTCTCTTCAATTCGAAAAAGAATTT 700
P._delicatulus_site_25________       ATTTGTATTTATTATTTGAGAAAGATTCTCTTCAATTCGAAAAAGAATTT 700
P._delicatulus_site_28________       ATTTGTATTTATTATTTGAGAAAGATTCTCTTCAATTCGAAAAAGAATTT 700
P._kawadai_site_6___________         ATTTGTATTCATTATTTGAGAAAGATTCTCTTCAATCCGAAAAAGAATTT 700
P._kawadai_site_7_____________       ATTTGTATTCATTATTTGAGAAAGATTCTCTTCAATTCGAAAAAGAATTT 700
P._kawadai_site_8_____________       ATTTGTATTCATTATTTGAGAGAGATTCTCTTCAATTCGAAAAAGAATTT 700
P._delicatulus_site_26________       ATTTGTATTTATTATTTGAGAAAGATTCTCTTCAATTCGAAAAAGAATTT 700
P._delicatulus_site_27________       ATTTGTATTTATTATTTGAGAAAGATTCTCTTCAATTCGAAAAAGAATTT 700
P._takakuwai_site_11__________       ATTTATATTTATTATTTGAGAAAGATTCTCTTCAATTCGAAAAAGAATTT 700
P._oregonensis_sanmaeto_______       ATTTATATTCATTATTTGAGAAAGATTTTCATCAATACGAAAAAGAATTT 700
P._hongwonpyoi_birosa_________       ATTTTTATTTATTATTTGAGAGAGATTCTCTTCAATTCGAAAAAGAATTT 700
P._hongwonpyoi_jengoge________       ATTTTTATTTATTATTTGAGAGAGATTCTCTTCAATTCGAAAAAGAATTT 700
P._hongwonpyoi_deogyusan______       ATTTTTATTTATCATTTGAGAAAGATTTTCTTCAATTCGAAAAAGAATTT 700
P._hongwonpyoi_hangeryeong____       ATTTTTATTTATTATTTGAGAAAGATTTTCTTCAATTCGAAAAAGAATTT 700
P._hongwonpyoi_nogodan________       ATTTTTATTTATTATTTGAGAAAGATTTTCTTCAATTCGAAAAAGAATTT 700
P._hongwonpyoi_jeoksangsan____       ATTTTTATTTATTATTTGAGAAAGATTTTCTTCAATTCGAAAAAGAATTT 700
                                     * ** **** ** ** ***** ** ** ** ** ** *********** *

P._acuticollis_site_5_________       CTACATACAGAATAACATCATCAATTGAATGATTACAAAAAATACCACCT 750
P._takakuwai_site_6_________         CTACATACAGAATAACATCATCAATTGAATGATTACAAAAAATACCACCT 750
```

P._takakuwai_site_7__________	CTACATATAGAATAACATCATCAATTGAATGATTACAAAAAATACCACCT	750
P._takakuwai_site_8__________	CTACATACAGAATAACATCATCAATTGAATGATTACAAAAAATACCACCT	750
P._albisomni_site_9__________	CTACATACAGAATAACATCATCAATTGAATGATTACAAAAAATACCACCT	750
P._albisomni_site_3__________	CTACATACAGAATAACATCATCAATTGAATGATTACAAAAAATACCACCT	750
P._albisomni_site_1__________	CTACATACAGAATAACATCATCAATTGAATGATTACAAAAAATACCACCT	750
P._albisomni_site_4__________	CTACATATAGAATAACATCATCAATTGAATGATTACAAAAAATACCACCT	750
P._takakuwai_site_10_________	CTACATACAGAATAACATCATCAATTGAATGATTACAAAAAATACCACCT	750
P._acuticollis_site_2________	CTACATATAGAATAACATCATCAATTGAATGATTACAAAAAATACCACCT	750
P._viridicuprus_site_15_______	CTACTTATAGAATAACATCATCAATTGAATGATTACAAAAAATACCACCT	750
P._viridicuprus_site_16_______	CTACTTATAGAATAACATCATCAATTGAATGATTACAAAAAATACCGCCT	750
P._sue_site_22____________	CTACCTATAGAATAACATCATCAATTGAATGATTACAAAAAATACCACCT	750
P._viridicuprus_site_23_______	CTACTTATAGAATAACATCATCAATTGAATGATTACAAAAAATACCACCT	750
P._viridicuprus_site_25_______	CTACTTATAGAATAACATCATCAATTGAATGATTACAAAAAATACCACCT	750
P._viridicuprus_site_17_______	CTACTTATAGAATAACATCATCAATTGAATGATTACAAAAAATACCACCT	750
P._takakuwai_site_14_________	CCACTTACAGAATAACATCGTCAATTGAGTGATTACAAAAAATACCACCT	750
P._akitaorum_site_13_________	CCACTTACAGAATAACATCGTCAATTGAGTGATTACAAAAAATACCACCT	750
P._takakuwai_site_12_________	CCACTTACAGAATAACATCGTCAATTGAGTGATTACAAAAAATACCACCT	750
P._takakuwai_site_18_________	CCACTTACAGAATAACATCGTCAATTGAGTGATTACAAAAAATACCACCT	750
P._sugitai_site_19___________	CCACTTACAGAATAACATCGTCAATTGAGTGATTACAAAAAATACCACCT	750
P._sugitai_site_21___________	CCACTTACAGAATAACATCGTCAATTGAATGATTACAAAAAATACCACCT	750
P._urushiyamai_site_29________	CCACTTACAGAATAACATCATCAATTGAGTGATTACAAAAAATACCACCT	750
P._urushiyamai_site_30________	CCACTTACAGAATAACATCATCAATTGAGTGATTACAAAAAATACCACCT	750
P._urushiyamai_site_24________	CCACTTACAGAATAACATCATCAATTGAGTGATTACAAAAAATACCACCT	750
P._kawadai_site_10_________	CCACTTACAGAATAACATCATCAATTGAGTGATTACAAAAAATACCACCT	750
P._delicatulus_site_2_________	CCACTTACAGAATAACATCATCAATTGAGTGATTACAAAAAATACCACCT	750
P._delicatulus_site_6_________	CCACTTACAGAATAACATCATCAATTGAGTGATTACAAAAAATACCACCT	750
P._delicatulus_site_20________	CCACTTACAGAATAACATCATCAATTGAGTGATTCCAAAAAATACCACCT	750
P._delicatulus_site_25________	CCACTTACAGAATAACATCATCAATTGAATGATTACAAAAAATACCACCT	750
P._delicatulus_site_28________	CCACTTACAGAATAACATCATCAATTGAATGATTACAAAAAATACCACCT	750
P._kawadai_site_6__________	CCACTTATAGAATAACATCATCAATTGAATGATTACAAAAAATACCACCT	750
P._kawadai_site_7____________	CCACTTACAGAATAACATCATCAATTGAATGATTACAAAAAATACCACCT	750
P._kawadai_site_8____________	CCACTTACAGAATGACATCATCAATTGAATGATTACAAAAAATACCACCT	750
P._delicatulus_site_26________	CCACTTACAGAATAACATCATCAATTGAATGATTACAAAAAATACCACCT	750
P._delicatulus_site_27________	CCACTTACAGAATAACATCATCAATTGAATGATTACAAAAAATACCACCT	750
P._takakuwai_site_11_________	CCACTTATAGAATAACATCATCAATTGAATGATTACAAAAAATACCACCC	750
P._oregonensis_sanmaeto_______	CTACTTATAGAATAACATCATCAATTGAGTGATTACAAAAAATACCTCCT	750

```
P._hongwonpyoi_birosa_________        CAACATTTAGAATAACGTCATCAATCGAGTGATTACAAAAAATACCACCA 750
P._hongwonpyoi_jengoge________        CAACATTTAGAATAACGTCATCAATCGAGTGATTACAAAAAATACCACCA 750
P._hongwonpyoi_deogyusan______        CAACATTCAGAATAACGTCATCAATCGAGTGATTACAAAAAATACCACCA 750
P._hongwonpyoi_hangeryeong____        CAACATTTAGAATAACATCATCAATCGAATGATTACAAAAAATACCGCCA 750
P._hongwonpyoi_nogodan________        CAACATTTAGAATAACATCATCAATTGAGTGATTACAAAAAATACCACCA 750
P._hongwonpyoi_jeoksangsan____        CAACACTCAGAATAACGTCATCAATCGAGTGATTACAAAAAATACCACCA 750
                                      * **    ***** ** ** ***** ** ***** *********** **

P._acuticollis_site_5_________        GCA 753
P._takakuwai_site_6__________         GCA 753
P._takakuwai_site_7___________        GCA 753
P._takakuwai_site_8___________        GCA 753
P._albisomni_site_9___________        GCA 753
P._albisomni_site_3___________        GCA 753
P._albisomni_site_1___________        GCA 753
P._albisomni_site_4___________        GCA 753
P._takakuwai_site_10__________        GCA 753
P._acuticollis_site_2_________        GCA 753
P._viridicuprus_site_15_______        GCA 753
P._viridicuprus_site_16_______        GCA 753
P._sue_site_22_____________           GCA 753
P._viridicuprus_site_23_______        GCA 753
P._viridicuprus_site_25_______        GCA 753
P._viridicuprus_site_17_______        GCA 753
P._takakuwai_site_14__________        GCA 753
P._akitaorum_site_13__________        GCA 753
P._takakuwai_site_12__________        GCA 753
P._takakuwai_site_18__________        GCA 753
P._sugitai_site_19____________        GCA 753
P._sugitai_site_21____________        GCA 753
P._urushiyamai_site_29________        GCA 753
P._urushiyamai_site_30________        GCA 753
P._urushiyamai_site_24________        GCA 753
P._kawadai_site_10__________          GCA 753
P._delicatulus_site_2_________        GCA 753
P._delicatulus_site_6_________        GCA 753
```

```
P._delicatulus_site_20________      GCA 753
P._delicatulus_site_25________      GCA 753
P._delicatulus_site_28________      GCA 753
P._kawadai_site_6___________        GCA 753
P._kawadai_site_7_____________      GCA 753
P._kawadai_site_8_____________      GCA 753
P._delicatulus_site_26________      GCA 753
P._delicatulus_site_27________      GCA 753
P._takakuwai_site_11__________      GCA 753
P._oregonensis_sanmaeto_______      GCA 753
P._hongwonpyoi_birosa_________      GCA 753
P._hongwonpyoi_jengoge________      GCA 753
P._hongwonpyoi_deogyusan______      GCA 753
P._hongwonpyoi_hangeryeong____      GCA 753
P._hongwonpyoi_nogodan________      GCA 753
P._hongwonpyoi_jeoksangsan____      GCA 753
                                    ***
```
